# Genetic factors for short life span associated with evolution of the loss of flight ability

**DOI:** 10.1002/ece3.6342

**Published:** 2020-05-29

**Authors:** Atsushi Ikemoto, Daiki X. Sato, Takashi Makino, Masakado Kawata

**Affiliations:** ^1^ Graduate School of Life Sciences Tohoku University Sendai Japan

**Keywords:** evolution of flying, *IGF2BP2*, longevity, maximum life span, metabolism

## Abstract

Acquisition or loss of flying ability is evolutionarily linked with maximum life span (MLS) in mammals and birds. Although ecological factors, such as extrinsic mortality, may lead to either shortened or extended life spans through natural selection, MLS is influenced by complex molecular and metabolic processes, and the genetic changes associated with flying ability that have led to either a longer or shorter MLS are unknown. Here, we examine the parallel evolution of flight in mammals and birds and investigate positively selected genes at branches where either the acquisition (in little brown bats and large flying foxes) or loss (in Adélie penguins, emperor penguins, common ostriches, emus, great spotted kiwis, little spotted kiwis, okarito brown kiwis, greater rheas, lesser rheas, and cassowaries) of flight abilities occurred. Although we found no shared genes under selection among all the branches of interest, 7 genes were found to be positively selected in 2 of the branches. Among the 7 genes, only *IGF2BP2* is known to affect both life span and energy expenditure. The positively selected mutations detected in *IGF2BP2* likely affected the functionality of the encoded protein. *IGF2BP2*, which has been reported to simultaneously prolong life span and increase energy expenditure, could be responsible for the evolution of shortened MLS associated with the loss of flying ability.

## Graphical abstract

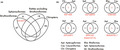

## 1 INTRODUCTION

Maximum life span (MLS) is a fundamental life‐history trait related to the rate of aging and senescence in animals. It has been proposed that species with lower extrinsic mortalities have longer life spans because they can invest in long‐term survival (Ricklefs, 2010; Stearns, 1992; Williams, 1957). Extrinsic mortality is generally determined by ecological factors, such as climate and predation risk, and may drive shortened or extended life spans through natural selection. However, MLS is influenced by complex molecular and metabolic processes such as mitochondrial homeostasis (Katsyuba et al., 2018), the production of reactive oxygen species (ROS) (Barja, 2004), and telomere shortening (Monaghan & Haussmann, 2006). Adaptive changes in life span through natural selection could be constrained by these complex intrinsic factors.

Mitochondria of aerobic animals produce ROS (Barja, 2004), which can damage lipids, proteins, and nucleic acids (Hekimi, Lapointe, & Wen, 2011). A low rate of mitochondrial ROS generation reportedly leads to long life spans in both long‐lived and calorie‐restricted animals because of low levels of both oxidative stress and accumulation of mutations in somatic mitochondrial DNA (Barja, 2004). Because animals with higher metabolic rates produce more ROS, a causal relationship between metabolic rate and life span can be expected. Additionally, a positive relationship between body mass and life span is pervasive in vertebrates (Cohen, 2018; de Magalhães, Costa, & Church, 2007). Because metabolic rates per mass are lower with increasing body mass, animals with smaller body masses could suffer more from ROS, and their life spans would be correspondingly shorter.

However, flight ability significantly affects MLS and aging rates in both mammals and birds regardless of body mass (Healy et al., 2014; Ricklefs, 2010). Flight typically requires higher rates of energy consumption and generates more ROS than other types of locomotion, such as walking or swimming (Maina, 2000). However, a prolonged life span often evolved with the acquisition of flight ability, suggesting that there is no simple relationship between metabolism and life span (Austad & Fischer, 1991). In vertebrates, the evolution of flight occurred in parallel in both bat and bird clades. By contrast, the loss of flight occurred many times in birds. Examples include the ancestors of penguins and some paleognaths and rails (Harshman et al., 2008; Sackton et al., 2019). Along with flight acquisition or loss, MLS can also become longer or shorter, respectively. A longer MLS in volant species might be explained by ecological selective pressures; because volant organisms can more easily escape predators, reproduction later in life could have evolved as extrinsic mortality decreased (Healy et al., 2014; Pomeroy, 1990). It is therefore expected that genetic changes affecting MLS could have occurred in line with the evolution of flight ability due to natural selection. However, it is unclear which genetic changes caused the longer MLSs and higher energy expenditures associated with the acquisition of flight ability, even though volant species would be expected to have higher metabolic rates and therefore suffer more from the toxic effects of ROS. We hypothesized that genes affecting a longer/shorter MLS and higher/lower energy expenditure could be favored by natural selection associated with the acquisition or loss of flight ability, and that these genes may have evolved to suppress or enhance the generation of ROS and aging by changing energy expenditure rates.

Recently, genetic factors underlying differences in aging and life spans among species have been explored through comparative genomics (Gorbunova, Seluanov, Zhang, Gladyshev, & Vijg, 2014). Among rodents, the naked mole rat and the blind mole rat have extremely long life spans (Azpurua & Seluanov, 2013; Buffenstein, 2005; Ruby, Smith, & Buffenstein, 2018). Genes related to cancer and stress resistance could contribute to the evolution of long life spans (Gorbunova et al., 2014). In particular, high‐molecular‐mass hyaluronan plays a crucial role in both cancer resistance through cell‐contact inhibition and longevity through stress resistance (Gorbunova et al., 2014; Seluanov et al., 2009; Tian et al., 2013). The gray squirrel also has a long life span, and its cells do not secrete high‐molecular‐mass hyaluronan but may have unknown mechanisms for cell cycle control, and they show extremely high telomerase activity (Gorbunova et al., 2014). Bats are species in which flying ability evolved that also enjoy a longer MLS (de Magalhães et al., 2007). A genome analysis of an exceptionally long‐lived bat, *Myotis brandtii*, revealed that growth hormone receptors and insulin‐like growth factor 1 (IGF‐1) receptors may have contributed to the evolution of their long life spans (Seim et al., 2013). These studies indicated that the genes associated with the evolution of a long MLS could be species‐ or lineage‐specific and that genes contributing to the evolution of a long MLS associated with flying ability may also be lineage‐specific. Previous studies identified several candidate genes involved in the flight ability of some flightless bird groups. Although most of the candidate genes did not overlap among the lineages (Burga et al., 2017; Campagna, McCracken, & Lovette, 2019; Clarke, 2019; Sackton et al., 2019), a few genes associated with energy metabolism have been reported to be commonly selected in numerous bird species that are either flightless or weak fliers (Pan et al., 2019), suggesting the presence of commonly selected genes when flight loss has occurred in birds.

To shed light on the parallel evolution of either longer or shorter MLSs with that of either acquisition or loss of flight ability, it is necessary to examine candidate genes that have been subject to positive selection in multiple lineages of birds that have lost their flying ability in addition to those of mammals that have evolved the ability to fly. The purpose of our research was to detect positively selected genes at the branches where the acquisition or loss of flying ability occurred, using whole‐genome‐sequenced mammals and birds, and to explore the genes responsible for shorter or longer MLSs associated with the evolution of the loss or acquisition of flying ability. We chose two bats, the large flying fox (*Pteropus vampyrus*) and the little brown bat (*Myotis lucifugus*), as volant species with a longer MLS in mammals; and two penguins, the Adélie penguin (*Pygoscelis adeliae*) and the emperor penguin (*Aptenodytes forsteri*), and eight ratites, the common ostrich (*Struthio camelus*), the emu (*Dromaius novaehollandiae*), the great spotted kiwi (*Apteryx haastii*), the little spotted kiwi (*Apteryx owenii*), the okarito brown kiwi (*Apteryx rowi*), the greater rhea (*Rhea americana*), the lesser rhea (*Rhea pennata*), and the cassowary (*Casuarius casuarius*), as nonvolant birds with a shorter MLS. We then searched for positively selected genes among the different lineages to identify genes that evolved in parallel and are related to both flying and life span. Genes detected among multiple branches where changes in flying ability had occurred are discussed for their possible roles in energy expenditure and alteration in life spans.

## 2 MATERIALS AND METHODS

### 2.1 Acquisition of ortholog datasets

Mammals and birds are characterized by homeothermism and include both volant and nonvolant species. We used avian species with genomes that had been fully sequenced with high coverage and excluded species with poor flying ability (such as chickens and turkeys) or those that rarely fly (such as the tinamou or hoatzin). For mammals, the genome sequences of the cat (*Felis catus*), cow (*Bos taurus*), dog (*Canis lupus familiaris*), elephant (*Loxodonta africana*), horse (*Equus caballus*), human being (*Homo sapiens*), marmoset (*Callithrix jacchus*), mouse (*Mus musculus*), opossum (*Monodelphis domestica*), pig (*Sus scrofa*), platypus (*Ornithorhynchus anatinus*), rabbit (*Oryctolagus cuniculus*), rat (*Rattus norvegicus*), and sheep (*Ovis aries*) were selected as nonvolant species, and the large flying fox (*Pteropus vampyrus*) and little brown bat (*Myotis lucifugus*) were selected as volant species. All genome sequences were obtained from Ensembl 98 (Yates et al., 2016). For birds, the Adélie penguin (*Pygoscelis adeliae*), emperor penguin (*Aptenodytes forsteri*), common ostrich (*Struthio camelus*), emu (*Dromaius novaehollandiae*), great spotted kiwi (*Apteryx haastii*), little spotted kiwi (*Apteryx owenii*), okarito brown kiwi (*Apteryx rowi*), greater rhea (*Rhea americana*), lesser rhea (*Rhea pennata*), and cassowary (*Casuarius casuarius*) were selected as nonvolant species, and the American crow (*Corvus brachyrhynchos*), Anna's hummingbird (*Calypte anna*), bald eagle (*Haliaeetus leucocephalus*), mallard (*Anas platyrhynchos*), budgerigar (*Melopsittacus undulatus*), chimney swift (*Chaetura pelagica*), common cuckoo (*Cuculus canorus*), crested ibis (*Nipponia nippon*), downy woodpecker (*Picoides pubescens*), golden‐collared manakin (*Manacus vitellinus*), killdeer (*Charadrius vociferus*), little egret (*Egretta garzetta*), medium ground finch (*Geospiza fortis*), peregrine falcon (*Falco peregrinus*), pigeon (*Columba livia*), and zebra finch (*Taeniopygia guttata*) were selected as volant species. Genomes of the zebra finch, emu, and the three kiwis were obtained from Ensembl 98 (Yates et al., 2016), and greater rhea, lesser rhea, and cassowary genomes were obtained from a previous study (Sackton et al., 2019). The other birds’ genomes were obtained from GigaDB, as reported in Zhang et al. (2014). The reference values of high/low coverage were 5 or 50 in Ensembl or GigaDB, respectively. The large flying fox genome was used as a representative ancestor of large flying foxes and little brown bats, although coverage was lower than 5. Brandt's bat (*Myotis brandtii*), reportedly the longest‐living bat, and the flightless brown kiwi (*Apteryx australis*) were not included in our analysis as their assembled genome sequences included many gaps and a sufficient number of orthologous genes was not available. Analyses for mammals and birds were conducted separately. We performed reciprocal basic local alignment search tool (BLAST) searches and obtained the highest score pairs of one‐to‐one orthologs using the BLASTp algorithm in National Center for Biotechnology Information (NCBI) BLAST v.2.6.0+ (Camacho et al., 2009) for protein‐coding regions of each species (Table S1). Reciprocal BLAST searches were run against reference species: humans for mammals and zebra finches for birds. For genes that had the same BLAST score, the longest transcript was chosen as the representative sequence. We used one‐to‐one orthologous gene sets with full sequence information for the comparative species sets in mammals and birds, respectively (Figure S1).

### 2.2 Alignment and gene tree construction

**FIGURE 1:**
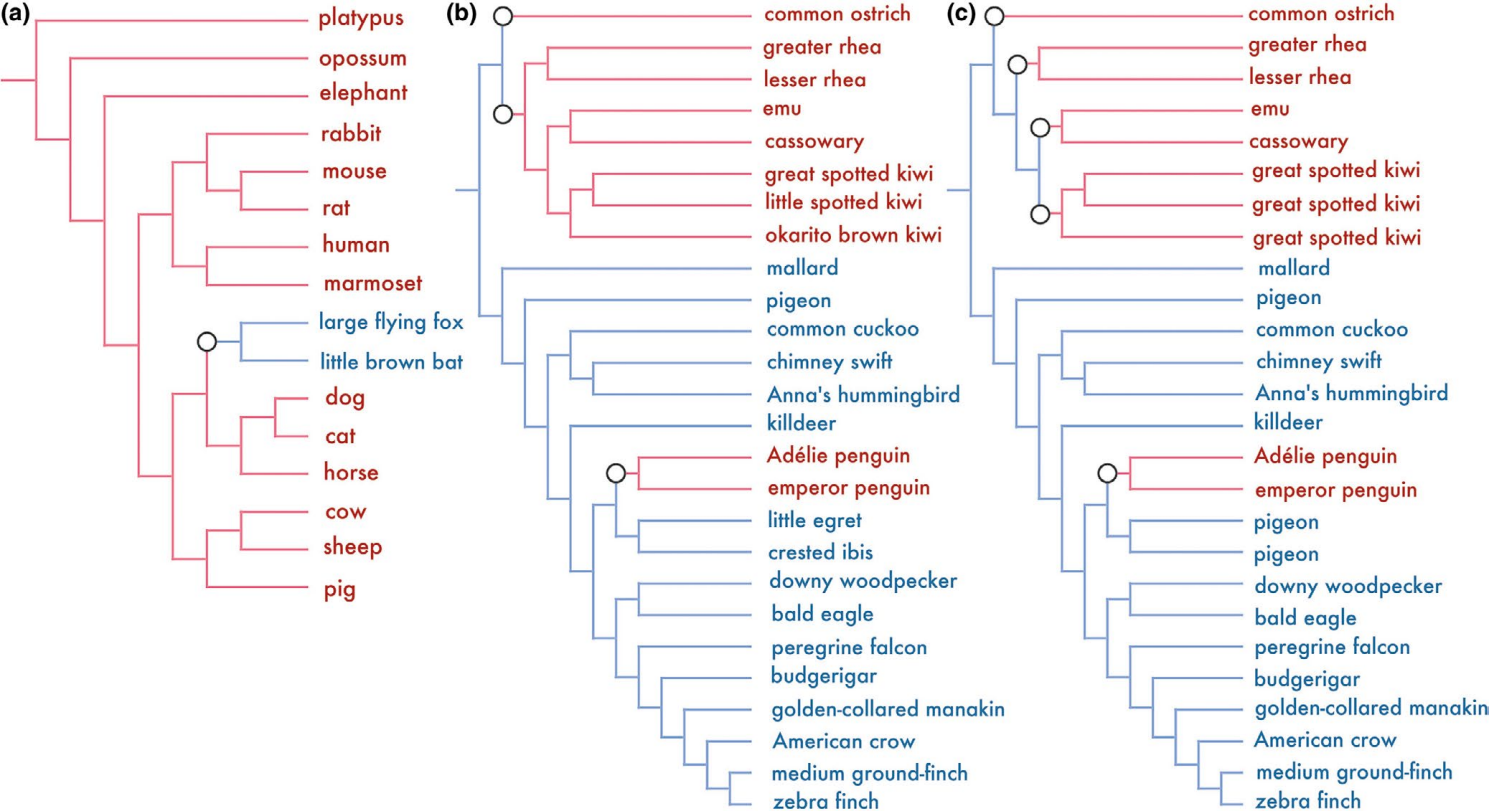
Species tree topologies used for detection of positive selection. The branches for volant and nonvolant taxa are displayed in blue and red, respectively. The branches of interest in positive selection analyses are displayed with gray circles. (a) Mammalian species tree topology reported in Ensembl 98 (Yates et al., 2016). (b) Avian species tree topology with the 2‐times flight‐loss hypothesis in ratites based on the previous studies (Jarvis et al., 2014; Sackton et al., 2019). (c) Avian species tree topology with the 4‐times flight‐loss hypothesis in ratites based on previous studies (Jarvis et al., 2014; Sackton et al., 2019)

Orthologs were aligned using PRANK v.170427 (Loytynoja & Goldman, 2005), which has been recommended for the detection of positive selection (Fletcher & Yang, 2010) due to its low false‐positive rates (Table S1). Initially, we used gene trees to detect positively selected genes to avoid false‐positive results due to incomplete lineage sorting (Mendes & Hahn, 2016; Wu, Kostyun, Hahn, & Moyle, 2018). We also detected positive selection with species trees independent of the analyses with gene trees, because short sequences and convergence may have made the analyses unreliable. Gene trees were generated using RAxML v.8.2.11 (Stamatakis, 2014), with the GTR + GAMMA substitution model and the codon positions' partition. The aligned sequences used for construction of gene trees were trimmed using trimAl v1.4.rev22 (Capella‐Gutiérrez, Silla‐Martínez, & Gabaldón, 2009), to ensure that more than 80% of species had the sequence for a given codon (Table S1). We obtained a mammalian species tree from Ensembl 98 (Yates et al., 2016) and an avian species tree from previous studies (Jarvis et al., 2014; Sackton et al., 2019) (Figure 1).

### 2.3 Detection of positively selected genes at the acquisition or loss of flying ability

We estimated positively selected genes using branch‐site model in PAML v.4.9i (Yang, 2007) and aBSREL in Hyphy v.2.3.7 (Smith et al., 2015), based on aligned orthologous sequences, for which the codons including either ambiguous characters or gaps in two or more species were removed (Table S1). We used gene trees and species trees without branch lengths and root information. Based on a previous study (Sackton et al., 2019), we adopted two hypotheses regardless where flightless evolutions occurred in the ratites: (a) a 2‐times hypothesis involving the ancestor of Struthioniformes (ostrich) and the ancestor of emu, kiwis, rheas, and cassowary; and (b) a 4‐times hypothesis, with the ancestor of Struthioniformes, Rheiformes (rheas), Apterygiformes (kiwis), and Casuariiformes (emu and cassowary), both apart from the lineage of penguins. We detected positive selection at 3 or 5 branches at which the loss of flying ability had occurred in birds, in addition to a branch where the acquisition of flying ability occurred in mammals (the last common ancestor of the large flying fox and little brown bat) (Figure 1). We performed positive selection analysis for genes that had a sufficient number of nucleotides to estimate gene trees and the level of positive selection after trimming. Taking into account the reliability of tree topology, we excluded genes with trees exhibiting multiphyletic origins for Chiroptera, Sphenisciformes (penguins), or ratites, excluding Struthioniformes for the 2‐times hypothesis, and those showing multiphyletic origins for Chiroptera, Sphenisciformes, Rheiformes, Apterygiformes, or Casuariiformes in the 4‐times hypothesis, respectively (Figure S1). The PAML analysis was performed independently, assuming positive selection to have occurred at each branch of interest. We used Bonferroni corrections to adjust the threshold of the *p* values by the number of tests performed for each ortholog (once in mammals, and 3 or 5 times under 2‐ or 4‐times hypothesis, respectively, in birds). We focused on genes detected via both calculations with a gene tree and species tree, and the genes detected by both PAML and aBSREL analyses were considered candidates under positive selection. To evaluate parallel and/or convergent evolution between avian and mammalian species, we used one‐to‐one ortholog information between humans (the reference species in mammals) and zebra finches (the reference species in birds) inferred by BLASTp (Camacho et al., 2009) (Table S1). Finally, a Bayes empirical Bayes method (Yang, Wong, & Nielsen, 2005) was used to estimate focal amino acids under positive selection in the given candidate genes.

### 2.4 Estimating the impact of amino acid substitutions

To detect genes through the filtering steps above, we calculated the impact of amino acid substitutions detected by the branch‐site model in PAML, using Provean v.1.1.5 (Choi, Sims, Murphy, Miller, & Chan, 2012), with NCBI nr protein database numbers 00 to 78, NCBI BLAST v.2.4.0+ (Camacho et al., 2009), CD‐HIT version 4.7 (Fu, Niu, Zhu, Wu, & Li, 2012; Li & Godzik, 2006), and SIFT v.6.2.1 (Kumar, Henikoff, & Ng, 2009), with UniProt reference 90 and NCBI BLAST v.2.6.0+ (Camacho et al., 2009) (Table S1). In this step, we used the sequences from humans and zebra finches as the query for mammals and birds, respectively. We determined whether positively selected sites belonged to protein domains using Prosite (Sigrist et al., 2013) and a reference species sequence.

### 2.5 Enrichment analysis for detected genes

For the genes detected in each clade, we analyzed enriched gene ontology (GO) terms and pathways using the over‐representation test with Fisher's exact test and the Benjamini–Hochberg procedure in PANTHER version 14.1 (Mi et al., 2019; Thomas, 2003). Orthologous genes of reference species to the given genes under positive selection were utilized for this analysis. Because zebra finch Ensembl gene IDs were not available for the PANTHER software, we converted them to chicken Ensembl gene IDs using chicken protein sequences from Ensembl 98 and BLASTp (Camacho et al., 2009; Yates et al., 2016).

## 3 RESULTS

### 3.1 Orthologs and gene trees of mammals and birds

The reciprocal BLAST search produced 7,010 and 6,801 orthologs for mammals and birds, respectively. In mammals, Chiroptera formed a monophyletic taxon in 5,182 out of 7,010 obtained gene trees. Sphenisciformes and ratites, excluding the ostrich, formed a monophyletic taxon in 4,445 gene trees, and Sphenisciformes, Rheiformes, Apterygiformes, and Casuariiformes formed a monophyletic taxon in 5,745 out of 6,801 obtained trees for birds (Figure S1).

### 3.2 Positively selected genes at the branches of interest

**FIGURE 2:**
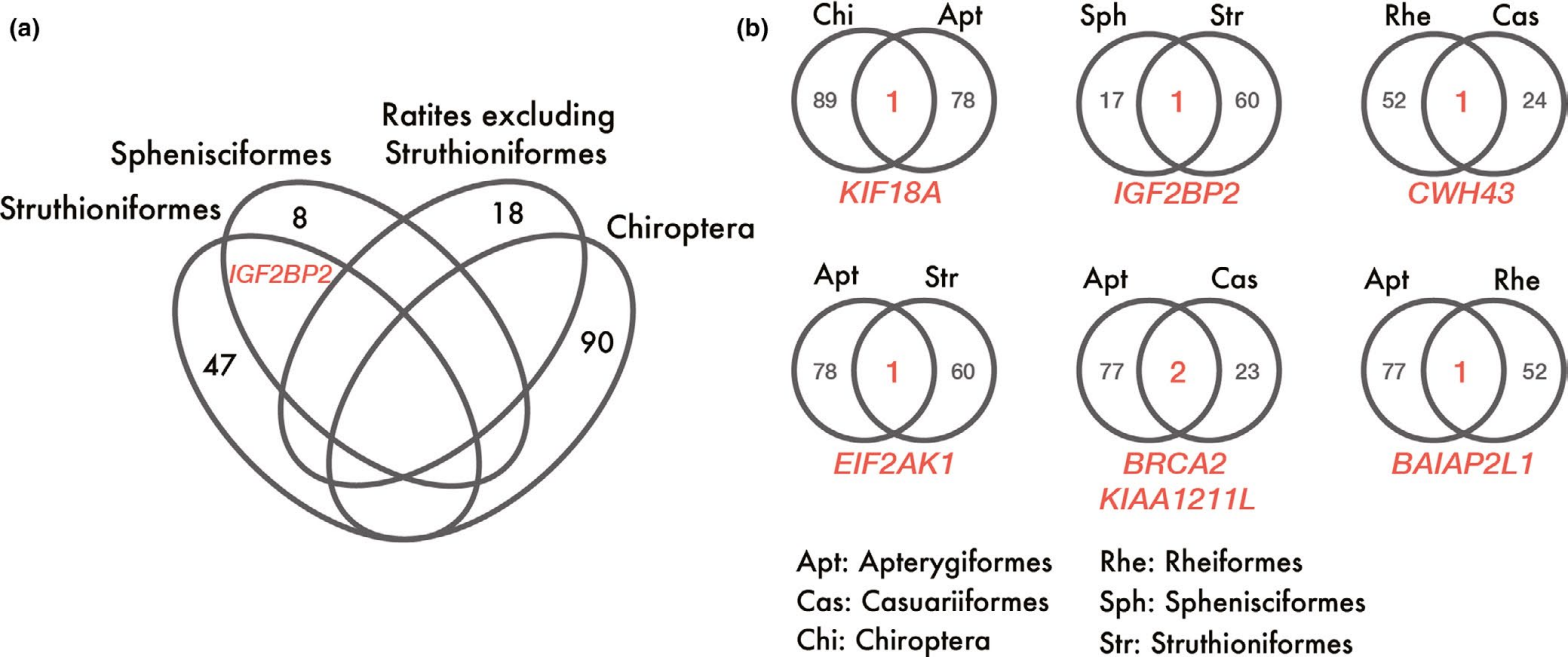
Positively selected genes at the branches of interest. The Venn diagram indicates the number of positively selected genes detected. (a) Results of the analysis assuming the 2‐times hypothesis. Only *IGF2BP2* was commonly detected at more than two branches. (b) Results of the analysis assuming the 4‐times hypothesis. Seven genes were commonly detected at two branches

The branch‐site model in PAML and aBSREL in Hyphy estimated 90 genes in Chiroptera were positively selected at the branches where flight acquisition occurred. In the analysis assuming the 2‐times hypothesis, we detected 9 genes in Sphenisciformes, 48 in Struthioniformes, and 18 in the ancestor of ratites without Struthioniformes. In the analysis based on the 4‐times hypothesis, 18 genes in Sphenisciformes, 61 in Struthioniformes, 53 in Rheiformes, 79 in Apterygiformes, and 25 in Casuariiformes were putatively under positive selection (Figure S2 and Tables S2–S10). Although we did not detect a common gene in all branches, 7 genes were positively selected in the 2 branches (Figure 2, Figures S3–S18 and Tables S2–S10). These 7 genes contained positively selected amino acid sites with empirical Bayes posterior probabilities >0.5, calculated by the branch‐site model in PAML (Table 1 and Tables S11–S16).

**TABLE 1:** Positively selected amino acid substitutions in *IGF2BP2* detected by the branch‐site model in PAML and their estimated impact on protein function. Values that were >0.95 in BEB, less than −2.5 in Provean, and <0.05 in SIFT are presented in bold; these amino acid changes could either have a remarkable impact on protein function (Provean and SIFT) or have been positively selected (PAML)

Branches in interest	Selected sites	Amino acids of zebra finch	Amino acids of target species	BEB probabilities when using gene tree	BEB probabilities when using species tree	Proven scores	SIFT scores
Sphenisciformes	346	P	R	0.911	0.915	**–4.990**	**0.01**
Struthioniformes	384	I	V	0.792	0.783	–0.864	0.15
	385	A	S	**0.997**	**0.997**	**–2.572**	**0.01**
	386	P	R	**0.985**	**0.987**	**–7.900**	0.09
	388	E	S	**0.998**	**0.998**	**–4.341**	**0.00**
	491	T	V	0.365	0.524	−1.582	0.30

### 3.3 Impacts of amino acid substitutions

**FIGURE 3:**
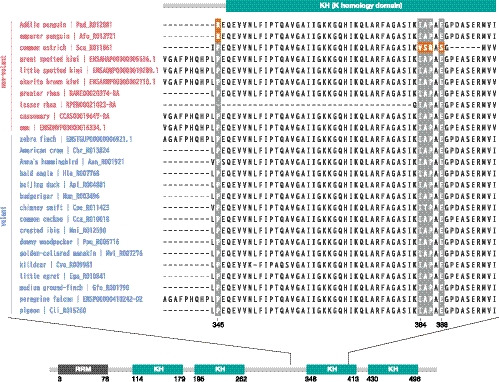
Domains in *IGF2BP2* and multiple alignments with the information of positively selected sites. Amino acid sites detected by PAML are colored orange in species of interest (foreground) and gray in other species (background). RRM is an RNA recognition motif, and KHs are K homology domains. The positions are based on the sequence of the zebra finch

For positively selected sites with Bayes empirical Bayes posterior probabilities greater than 0.5, the impact of amino acid substitutions was calculated. For *IGF2BP2*, *CWH43*, *EIF2AK1*, *KIAA1211L*, *BRCA2,* and *KIF18A*, both software packages determined some amino acid substitutions as remarkable changes that may have affected protein function (Table 1 and Tables S11–S16). Moreover, the positively selected sites in *IGF2BP2* either belong to or are located close to the third K homology domain, a RNA‐binding motif (Figure 3).

### 3.4 Enrichment analysis of genes detected in each clade

We analyzed the enriched GO terms and pathways for genes detected in Chiroptera, Sphenisciformes, Struthioniformes, ratites excluding Struthioniformes, Apterygiformes, Rheiformes, and Casuariiformes based on the 2‐ or 4‐times hypothesis. There were no enriched GO terms or pathways after correction (Tables S17–S52).

## 4 DISCUSSION

Our positive selection analyses detected 6 common genes in flightless groups and 1 gene in both volant and nonvolant groups (Figure 2). The detected genes shared among Chiroptera and avian branches where flight loss occurred may have played important roles in the evolution of both acquisition and loss of flying abilities. On the other hand, the detected genes shared only among avian branches could have played crucial roles only in the loss of flying abilities. In our study, few candidate genes were shared between the groups evolving with either acquisition or loss of flight, and there was no common gene shared among more than 2 groups (Figure 2). This suggests that a common gene may not necessarily have been selected when either the acquisition or loss of flight occurred during evolution.

Among the 7 genes we detected, *CWH43*, *EIF2AK1*, *BAIAP2L1*, and *BRCA2* have been reported to have functions related to life span and/or aging. For example, *CWH43* could affect chronological life spans in yeast (Fabrizio et al., 2010) or ovarian senescence in mice (Schneider et al., 2017). However, *CWH43* in the greater and lesser rhea may be paralogous as these species have many selected amino acids sites, making it difficult to conclude that *CWH43* in Rheiformes evolved under positive selection. Expression levels of *EIF2AK1* and *BAIAP2L1* were associated with biological age (Lin et al., 2019) or age (Dimitrakopoulou, Vrahatis, & Bezerianos, 2015), respectively. Mutant mice of *Brca2* had reduced life spans, possibly because of impaired DNA repair function, resulting in early onset of cancer and sepsis (Donoho et al., 2003). *BAIAP2L1* may also be related to energy expenditure given its upregulation during weight loss in humans (Larrouy et al., 2008). No clear evidence that these genes cause changes in both life span and energy expenditure has been reported to date, but *IGF2BP2* is known to relate to and affect both life span and energy expenditure (Dai et al., 2015).

*IGF2BP2*, detected in Sphenisciformes and Struthioniformes, belongs to a family of mRNA‐binding proteins that bind to mRNAs of other genes and regulate RNA processing (Yisraeli, 2005). Although *IGF2* genes were previously assumed to act only during developmental stages (Nielsen et al., 1999), expression of *IGF2BP2*, a member of the *IGF2* family, has been reported in adult organisms (Dai et al., 2011). *IGF2BP2* was detected in a genome‐wide association study targeting type 2 diabetes in humans (Saxena et al., 2007; Scott et al., 2007; Zeggini et al., 2007), and attention has recently been paid to this gene. In a previous study, *Igf2bp2* knockout mice simultaneously exhibited several phenotypes, such as higher energy expenditures, longer life spans, and smaller body size compared with wild mice, and it has been suggested that the expression of *Ucp1*, an uncoupling protein negatively regulated by IGF2BP2, could play a key role in energy expenditure (Dai et al., 2015). In addition, it has been suggested that uncoupling proteins suppress the generation of ROS and aging (Mookerjee, Divakaruni, Jastroch, & Brand, 2010). It is therefore likely that IGF2BP2 is associated with both energy expenditure and life span (Figure S19). This is consistent with the association between flying ability and longevity in that IGF2BP2 can simultaneously trigger conflicting phenomena: increased energy expenditure and a prolonged life span. However, *IGF2BP2* was only detected in birds, and birds have lost *UCP1* and *UCP2* (Emre et al., 2007). Instead, birds have *UCP3*, which acts in mitochondria (Emre et al., 2007). Because IGF2BP2 regulates the translation of genes in mitochondria (Dai et al., 2015), it is expected that genes acting in mitochondria, such as *UCP3*, are suppressed by the activation of IGF2BP2, and accordingly, an increase in energy expenditure and the prolongation of life span may co‐occur. *IGF2BP2* was not detected to be positively selected at the branch of Chiroptera, possibly due to the very different regulatory pathways among species.

Genome analysis of the long‐lived Brandt's bat (*Myotis brandtii*) (Seim et al., 2013) indicated that unique sequence changes in growth hormones and insulin‐like growth factor 1 (IGF‐1) receptors may be associated with the species’ exceptionally long life span. Although the present study could not detect *IGF‐1*, *IGF‐1* is a paralog of *IGF‐2* that encoded the ligand of IGF2BP2. A pathway involving IGF‐1, also called the IGF‐1 axis, is associated with life spans in nematodes, fruit flies, and mice, and its function is evolutionarily highly conserved (Longo & Finch, 2003). In addition, IGF2BP2 reportedly promotes the translation of *Igf1r* in mice (Li et al., 2012), which is also related to the IGF‐1 axis and life span. The final transcriptional product of the IGF‐1 axis is FOXO, which is known to affect life spans associated with the regulation of stress and DNA repair (Ziv & Hu, 2011). Moreover, IGFs may be involved in energy consumption, as they are associated with glucose metabolism. In nematodes, *daf‐2*, an orthologous gene of *Igf1r*, knockout individuals exhibited prolonged life spans (Kenyon, Chang, Gensch, Rudner, & Tabtiang, 1993). *Igf1r* hetero knockout mice also exhibited prolonged life spans (Holzenberger et al., 2003) although *Igf1r* homo knockout was lethal. These studies show that IGF1R negatively regulates life span (Figure S19). It is therefore safe to assume that activated IGF2BP2 in Sphenisciformes and Struthioniformes increases *IGF1R* protein, leading to shortened life spans. Seim *et al*. found specific amino acid substitutions of *IGF1R*, which probably affects protein function, in a subset of Chiroptera, including the little brown bat, which was used in the current study, and Brandt's bat. This suggests that common pathways rather than common genes have evolved associated with life span and the acquisition or loss of flying ability in both mammals and birds. Additionally, it is assumed that the evolution of longer life spans occurred several times in Chiroptera, as positive selection of *IGF1R* was not detected at the ancestral branch of the large flying fox and little brown bat. Still, whether the amount of *IGF1R* protein is correlated with life span, and how mutations in *IGF2BP2* affect IGF1R warrant further study to reveal the detailed evolutionary mechanisms.

The current study revealed that *IGF2BP2* was positively selected when the loss of flight occurred independently in two taxa of birds. Given that *IGF2BP2* is associated with both energy expenditure and life span, and IGF1R also affects both life span and energy consumption in some bats, evolutionary changes in these genes may suppress the generation of ROS and aging by energy expenditure mechanisms such as uncoupling proteins. This also suggests that life span can been shortened and/or prolonged as a pleiotropic effect of the decrease and/or increase in energy expenditure associated with flight.

Detecting *IGF2BP2* under positive selection in multiple nonvolant avian species may support our hypothesis that a gene affecting both a shorter MLS and lower energy expenditure could be favored by natural selection associated with the loss of flight ability. Five other positively selected genes also have some relevant functions, suggesting roles in co‐evolution of flight ability and life spans. However, our analyses found no other genes were shared among multiple taxa, which suggests that different genes were involved in changing MLS and energy expenditure independently associated with flight ability in many other cases. Because we focused on coding regions and used amino acid sites existing in almost all the analyzed mammals or birds, further study is needed to determine extent to which our hypothesis can be supported.

## CONFLICT OF INTEREST

None declared.

## AUTHOR CONTRIBUTION

**Atsushi Ikemoto:** Formal analysis (equal); Visualization (equal); Writing‐original draft (equal); Writing‐review & editing (equal). **Daiki X. Sato:** Formal analysis (supporting); Writing‐original draft (supporting); Writing‐review & editing (supporting). **Takashi Makino:** Formal analysis (supporting); Writing‐review & editing (supporting). **Masakado Kawata:** Conceptualization (equal); Supervision (lead); Writing‐original draft (equal); Writing‐review & editing (lead). 

## Supporting information

Fig S1‐S19

Table S1‐S52

## Data Availability

Nucleotide and protein sequences used for this study were downloaded from Ensembl 98, GigaDB, and a previous study (Sackton et al., 2019). All the other data are included in Supplementary files.
