# Supplementary material for: Genetic factors for short life span associated with evolution of the loss of flight ability
Source: Ecol Evol. 2020 May 29;10(12):6020–9. doi: 10.1002/ece3.6342 (PMC7319159; doi:10.1002/ece3.6342)
Supplement: Supplementary file 1 — Fig S1‐S19 [file ECE3-10-6020-s001.docx]

Figure S1. The flowchart of detecting positively selected genes.

Figure S2. The number of detected genes using either the branch-site model in PAML or aBSREL in Hyphy. (a) 2-times hypothesis. (b) 4-times hypothesis.

Figure S3. *IGF2BP2* protein sequence alignment of avian species. Amino acid substitutions detected by PAML are colored orange in species of interest (foreground) and gray in other species (background). Amino acid positions are based on the alignment.

Figure S4. *KIF18A* protein sequence alignment of mammalian species. Amino acid substitutions detected by PAML are colored orange in species of interest (foreground) and gray in other species (background). Amino acid positions are based on the alignment.

Figure S5. *KIF18A* protein sequence alignment of avian species. Amino acid substitutions detected by PAML are colored orange in species of interest (foreground) and gray in other species (background). Amino acid positions are based on the alignment.

Figure S6. *CWH43* protein sequence alignment of avian species. Amino acid substitutions detected by PAML are colored orange in species of interest (foreground) and gray in other species (background). Amino acid positions are based on the alignment.

Figure S7. *EIF2AK1* protein sequence alignment of avian species. Amino acid substitutions detected by PAML are colored orange in species of interest (foreground) and gray in other species (background). Amino acid positions are based on the alignment.

Figure S8. *BRCA2* protein sequence alignment of avian species. Amino acid substitutions detected by PAML are colored orange in species of interest (foreground) and gray in other species (background). Amino acid positions are based on the alignment.

Figure S9. *KIAA1211L* protein sequence alignment of avian species. Amino acid substitutions detected by PAML are colored orange in species of interest (foreground) and gray in other species (background). Amino acid positions are based on the alignment.

Figure S10. *BAIAP2L1* protein sequence alignment of avian species. Amino acid substitutions detected by PAML are colored orange in species of interest (foreground) and gray in other species (background). Amino acid positions are based on the alignment.

Figure S11. The unrooted gene tree of avian *IGF2BP2*. Values on each node indicate bootstrap values. Branches considered to be volant taxa and non-volant taxa are displayed in blue and red, respectively. Branches of interest in positive selection analyses are displayed using gray outlined circles. (a) Branches of interest in 2-times hypothesis. (b) Branches of interest in 4-times hypothesis.

Figure S12. The unrooted gene tree of mammalian *KIF18A*. Values on each node indicate bootstrap values. Branches considered to be volant taxa and non-volant taxa are displayed in blue and red, respectively. Branches of interest in positive selection analyses are displayed using gray outlined circles.

Figure S13. The unrooted gene tree of avian *KIF18A*. Values on each node indicate bootstrap values. Branches considered to be volant taxa and non-volant taxa are displayed in blue and red, respectively. Branches of interest in positive selection analyses are displayed using gray outlined circles.

Figure S14. The unrooted gene tree of avian *CWH43*. Branches considered to be volant taxa and non-volant taxa are displayed in blue and red, respectively. (a) The unrooted tree with branch lengths and bootstrap values. Values on each node indicate bootstrap values. (b) The unrooted tree without branch lengths and bootstrap values. Branches of interest in positive selection analyses are displayed using gray outlined circles.

Figure S15. The unrooted gene tree of avian *EIF2AK1*. Values on each node indicate bootstrap values. Branches considered to be volant taxa and non-volant taxa are displayed in blue and red, respectively. Branches of interest in positive selection analyses are displayed using gray outlined circles.

Figure S16. The unrooted gene tree of avian *BRCA2*. Values on each node indicate bootstrap values. Branches considered to be volant taxa and non-volant taxa are displayed in blue and red, respectively. Branches of interest in positive selection analyses are displayed using gray outlined circles.

Figure S17. The unrooted gene tree of avian *KIAA1211L*. Values on each node indicate bootstrap values. Branches considered to be volant taxa and non-volant taxa are displayed in blue and red, respectively. Branches of interest in positive selection analyses are displayed using gray outlined circles.

Figure S18. The unrooted gene tree of avian *BAIAP2L1*. Values on each node indicate bootstrap values. Branches considered to be volant taxa and non-volant taxa are displayed in blue and red, respectively. Branches of interest in positive selection analyses are displayed using gray outlined circles.

Figure S19. The expected pathways of *Igf2bp2* contributing to energy expenditure and longevity.
