# Supplementary material for: Genetic factors for short life span associated with evolution of the loss of flight ability
Source: Ecol Evol. 2020 May 29;10(12):6020–9. doi: 10.1002/ece3.6342 (PMC7319159; doi:10.1002/ece3.6342)
Supplement: Supplementary file 2 — Table S1‐S52 [file ECE3-10-6020-s002.docx]

Table S1. Software options used in the analyses.

| Software | Options |
| --- | --- |
| BLASTp | -evalue 1e-15 |
| PRANK | -codon -seed=12345 |
| RAxML (best tree search) | -p 12345 -m GTRGAMMA -N 100 |
| RAxML (bootstrap) | -p 12345 -x 12345 -m GTRGAMMA -N 1000 |
| trimAl | -gt 0.8 |
| codeml (alternative model) | model=2, NSsites=2, fix_omega=0, omega=1, runmode=0, seqtype=1, CodonFreq=2, clock=0, icode=0, fix_kappa=0, kappa=2, fix_alpha=1, alpha=.0, Malpha=0, ncatG=4, fix_blength=0, cleandata=0 |
| codeml (null model) | model=2, NSsites=2, fix_omega=1, omega=1, runmode=0, seqtype=1, CodonFreq=2, clock=0, icode=0, fix_kappa=0, kappa=2, fix_alpha=1, alpha=.0, Malpha=0, ncatG=4, fix_blength=0, cleandata=0 |
| aBSREL | with default parameters |
| PROVEAN | with default parameters |
| SIFT | with default parameters |

Table S2. Positively selected genes at the branch for Chiroptera.

| Gene name | Ensembl ID | Branch-site model in PAML  (gene tree) | | Branch-site model in PAML  (species tree) | | aBSREL (gene tree) | | aBSREL (species tree) | |
| --- | --- | --- | --- | --- | --- | --- | --- | --- | --- |
|  |  | dN/dS | P-value | dN/dS | P-value | dN/dS | P-value | dN/dS | P-value |
| *DBF4* | ENSG00000006634 | 139.38228 | 0.007855168 | 135.04913 | 0.007783017 | 36.72646571 | 0.031285 | 34.35117812 | 0.032520941 |
| *HIVEP2* | ENSG00000010818 | 107.06562 | 0.01666271 | 105.78389 | 0.01753505 | 16.99142076 | 0.016188969 | 19.49654351 | 0.041482087 |
| *ABCC2* | ENSG00000023839 | 35.77276 | 0.0111906 | 31.9088 | 0.01211148 | 13.63788616 | 0.000109162 | 14.81532685 | 4.11E-05 |
| *CDH1* | ENSG00000039068 | 999 | 0.000161091 | 999 | 0.000126492 | 285.7223068 | 0.005684802 | 148.1409385 | 0.001099247 |
| *LSG1* | ENSG00000041802 | 201.1572 | 0.001784545 | 179.56671 | 0.004118292 | 324.4767248 | 0.004212792 | 239.1857017 | 0.012412356 |
| *TFE3* | ENSG00000068323 | 65.93488 | 0.0137268 | 59.82524 | 0.01636803 | 48.81697773 | 0.021469694 | 30.14040969 | 0.034037253 |
| *NEDD4* | ENSG00000069869 | 97.3123 | 0.01176509 | 90.47487 | 0.01196597 | 212.0097435 | 0.017889536 | 179.4755219 | 0.017750282 |
| *ZFYVE26* | ENSG00000072121 | 999 | 0.01268984 | 999 | 0.01748241 | 6472.943039 | 0.006457228 | 6601.277949 | 0.008102541 |
| *LRP2* | ENSG00000081479 | 9.31283 | 0.000131242 | 9.38684 | 0.000207466 | 8.596485999 | 1.09E-09 | 7.41747256 | 5.34E-09 |
| *ERC1* | ENSG00000082805 | 999 | 0.000710641 | 999 | 0.000383697 | 10000 | 0.001259901 | 10000 | 0.000302842 |
| *FAM234B* | ENSG00000084444 | 999 | 0.01867401 | 999 | 0.02355356 | 10000 | 0.024147526 | 10000 | 0.031677692 |
| *FAT2* | ENSG00000086570 | 11.89979 | 0.007421696 | 12.05623 | 0.005554381 | 3.888339558 | 0.047337604 | 3.956193779 | 0.031803584 |
| *UIMC1* | ENSG00000087206 | 999 | 0.02027649 | 999 | 0.02022294 | 475.2122714 | 0.030350094 | 433.9681257 | 0.030050912 |
| *SIGLEC1* | ENSG00000088827 | 105.75977 | 1.08E-05 | 117.74148 | 5.68E-05 | 27.94026125 | 0.002237694 | 30.23671255 | 0.003438172 |
| *DZANK1* | ENSG00000089091 | 66.29156 | 0.0186569 | 65.80386 | 0.01917547 | 17.68567086 | 0.013341648 | 17.75626831 | 0.014033085 |
| *SLC26A3* | ENSG00000091138 | 23.55157 | 0.002156572 | 27.155 | 0.000968958 | 28.93335162 | 0.002893621 | 29.36073147 | 6.15E-05 |
| *RAPGEF4* | ENSG00000091428 | 161.20449 | 0.006099573 | 159.9349 | 0.006016761 | 45.0168495 | 0.027960443 | 40.70147972 | 0.0273967 |
| *PLA2G3* | ENSG00000100078 | 208.36715 | 0.007666708 | 147.05787 | 0.01122388 | 44.35639921 | 0.012658117 | 52.63673588 | 0.015959981 |
| *GTPBP1* | ENSG00000100226 | 62.26498 | 0.01349824 | 68.28438 | 0.0115883 | 745.5356022 | 0.030233901 | 391.9036665 | 0.010228625 |
| *IKBKB* | ENSG00000104365 | 211.78907 | 0.005376372 | 208.44463 | 0.005417922 | 163.2288002 | 0.030637155 | 212.6311507 | 0.031541671 |
| *GARS* | ENSG00000106105 | 32.92992 | 0.02775705 | 36.37539 | 0.02427504 | 23.47484035 | 0.042035047 | 25.3324533 | 0.038910461 |
| *BLVRA* | ENSG00000106605 | 87.57246 | 0.001976374 | 78.27099 | 0.002473104 | 85.75790445 | 0.000664772 | 62.02906016 | 0.000902449 |
| *SUSD1* | ENSG00000106868 | 999 | 0.0314068 | 999 | 0.04168511 | 1999.035959 | 0.010662316 | 2341.447052 | 0.010252823 |
| *TASOR2* | ENSG00000108021 | 87.43505 | 0.002373774 | 83.61212 | 0.003168468 | 17.75346787 | 0.000997297 | 18.19095889 | 0.001433736 |
| *KRT23* | ENSG00000108244 | 238.12682 | 0.008387463 | 238.12725 | 0.008387463 | 26.52235298 | 0.031321888 | 25.42065688 | 0.031429418 |
| *CSF3* | ENSG00000108342 | 999 | 0.004180201 | 999 | 0.003373594 | 475.8825361 | 0.010080023 | 260.5939923 | 0.013356033 |
| *PAK1IP1* | ENSG00000111845 | 448.02715 | 0.001029941 | 217.80746 | 0.002210399 | 815.2118514 | 0.000824423 | 403.4112283 | 0.001105711 |
| *IL17A* | ENSG00000112115 | 699.40999 | 0.00366898 | 117.0333 | 0.006144614 | 1097.980424 | 0.006224285 | 270.989265 | 0.021293115 |
| *ZNF451* | ENSG00000112200 | 230.66549 | 0.002620006 | 233.3623 | 0.002562358 | 50.33963642 | 0.026644228 | 50.57256855 | 0.026726591 |
| *ZBTB24* | ENSG00000112365 | 999 | 0.001974293 | 23.25172 | 0.008233769 | 144.259372 | 0.000996609 | 10.45043586 | 0.024231121 |
| *EHHADH* | ENSG00000113790 | 80.46913 | 0.00176361 | 79.12881 | 0.001828134 | 48.29156813 | 0.001616803 | 47.95191524 | 0.001503769 |
| *NCL* | ENSG00000115053 | 999 | 0.002690603 | 999 | 0.003017177 | 10000 | 0.00658678 | 10000 | 0.007251717 |
| *SWT1* | ENSG00000116668 | 63.24501 | 0.006769876 | 47.56482 | 0.02807943 | 49.53807168 | 0.000933531 | 24.59752024 | 0.002945813 |
| *AMPD1* | ENSG00000116748 | 15.77335 | 0.01852358 | 16.37143 | 0.0145955 | 10.63545544 | 0.04497485 | 23.49006454 | 0.00529879 |
| *ELOVL4* | ENSG00000118402 | 93.5506 | 0.01482556 | 95.85456 | 0.01439001 | 178.6820494 | 0.0063092 | 118.5839778 | 0.018057665 |
| *MED28* | ENSG00000118579 | 466.04975 | 0.000346063 | 999 | 0.000477603 | 111.6428886 | 0.002130392 | 77.67091324 | 0.003473738 |
| *KIF18A* | ENSG00000121621 | 63.03101 | 0.009950851 | 73.98181 | 0.01094298 | 24.28984559 | 0.014600655 | 17.97509959 | 0.022482241 |
| *KHDRBS1* | ENSG00000121774 | 999 | 0.000926337 | 999 | 0.001544949 | 10000 | 0.00062987 | 3350.026837 | 0.001214075 |
| *POLK* | ENSG00000122008 | 163.74551 | 0.000359675 | 192.27511 | 0.000282271 | 194.8531788 | 0.00371852 | 164.5005693 | 0.002339696 |
| *C4BPA* | ENSG00000123838 | 32.58521 | 0.008817536 | 27.33931 | 0.009830237 | 5.980714972 | 0.040737986 | 6.795474489 | 0.011297854 |
| *WRNIP1* | ENSG00000124535 | 129.76485 | 0.01304346 | 112.85056 | 0.01602173 | 61.81481756 | 0.028016116 | 54.05000013 | 0.031603652 |
| *SLC10A2* | ENSG00000125255 | 36.28841 | 0.02223937 | 36.51666 | 0.02191196 | 20.79406464 | 0.018230338 | 24.44821833 | 0.018714532 |
| *LRRN4* | ENSG00000125872 | 100.75679 | 0.02200123 | 69.23962 | 0.02429703 | 10000 | 0.002517693 | 56.74143747 | 0.04511161 |
| *TSPAN8* | ENSG00000127324 | 999 | 0.01595343 | 76.09065 | 0.01235589 | 10000000000 | 0.031286064 | 10000000000 | 0.018804649 |
| *METTL16* | ENSG00000127804 | 999 | 0.003320847 | 999 | 0.003140155 | 10000 | 0.003753069 | 10000 | 0.003723158 |
| *NECTIN2* | ENSG00000130202 | 29.54688 | 0.00066807 | 37.15313 | 0.000430783 | 17.08285226 | 0.001711726 | 377.6468515 | 0.000207087 |
| *COX4I1* | ENSG00000131143 | 131.49771 | 0.01813315 | 76.92694 | 0.02392477 | 214.5091266 | 0.009103762 | 117.329388 | 0.011930555 |
| *IL13RA1* | ENSG00000131724 | 50.16418 | 0.000951867 | 53.15778 | 0.000722604 | 28.74470747 | 0.000178643 | 28.88078081 | 0.000189667 |
| *CD36* | ENSG00000135218 | 14.55284 | 0.01698851 | 15.35802 | 0.02025853 | 62.98201139 | 0.000565448 | 62.88484923 | 0.000321218 |
| *APTX* | ENSG00000137074 | 861.30581 | 0.01436999 | 999 | 0.005305451 | 10000 | 0.00343181 | 10000 | 0.003499634 |
| *THBS1* | ENSG00000137801 | 80.02183 | 0.001802652 | 68.72687 | 0.002418275 | 30.33302331 | 0.015187524 | 28.53634242 | 0.024674254 |
| *SPPL2A* | ENSG00000138600 | 999 | 0.01709586 | 999 | 0.01627306 | 53.95589221 | 0.036549431 | 59.89545499 | 0.038135686 |
| *ENPEP* | ENSG00000138792 | 37.28287 | 0.02356895 | 39.73236 | 0.01792279 | 46.72565345 | 0.013073103 | 54.94391698 | 0.006873077 |
| *CHST4* | ENSG00000140835 | 138.96496 | 2.68E-06 | 134.00286 | 6.97E-06 | 112.1306392 | 9.31E-05 | 99.241138 | 0.000434016 |
| *TOR1AIP1* | ENSG00000143337 | 39.83149 | 0.008077909 | 40.59284 | 0.008074544 | 11.75110275 | 0.006201591 | 11.33170859 | 0.006818527 |
| *FANCD2* | ENSG00000144554 | 8.23515 | 0.0150576 | 8.88759 | 0.01323492 | 8.710599336 | 0.013324598 | 8.486679429 | 0.017203071 |
| *LYAR* | ENSG00000145220 | 356.7221 | 0.000537776 | 360.08313 | 0.000528996 | 10000 | 0.003332931 | 10000 | 0.003754558 |
| *ALAD* | ENSG00000148218 | 999 | 1.84E-09 | 999 | 1.61E-09 | 10000 | 4.63E-10 | 10000 | 3.40E-10 |
| *HSD17B12* | ENSG00000149084 | 138.27089 | 0.01319961 | 167.58335 | 0.002560092 | 5861.251146 | 0.00500217 | 584.6918129 | 0.002622575 |
| *TKFC* | ENSG00000149476 | 88.76571 | 0.005085518 | 57.72292 | 0.009793626 | 113.2396855 | 0.001240594 | 57.09513267 | 0.003448399 |
| *RAD9B* | ENSG00000151164 | 573.51151 | 0.008418777 | 999 | 0.002135749 | 10000 | 0.002246256 | 10000 | 0.002096637 |
| *EDNRA* | ENSG00000151617 | 999 | 0.000776774 | 999 | 0.000785101 | 10000 | 0.041045531 | 10000 | 0.041006863 |
| *FBXL18* | ENSG00000155034 | 328.34845 | 0.003174 | 998.99996 | 0.006450439 | 825.408641 | 0.026384992 | 577.3346896 | 0.023924352 |
| *OTOA* | ENSG00000155719 | 998.99962 | 2.26E-06 | 302.5601 | 1.82E-06 | 3037.58207 | 0.006630121 | 1790.649365 | 0.005660503 |
| *FRRS1* | ENSG00000156869 | 139.04097 | 0.000248858 | 170.74513 | 4.04E-05 | 21.5683482 | 0.015485551 | 17.88266296 | 0.004801939 |
| *PTPDC1* | ENSG00000158079 | 998.99992 | 0.007325266 | 999 | 0.007999331 | 4947.092615 | 0.020540095 | 5332.134258 | 0.020244542 |
| *XDH* | ENSG00000158125 | 166.70823 | 0.005218734 | 161.13416 | 0.005350197 | 186.232057 | 0.029171313 | 162.5704264 | 0.034333508 |
| *ADPGK* | ENSG00000159322 | 60.85149 | 0.005530263 | 63.83741 | 0.003666266 | 68.93885466 | 0.004929478 | 51.97611427 | 0.001415248 |
| *BDH1* | ENSG00000161267 | 999 | 0.004352612 | 767.3132 | 0.004182127 | 10000 | 0.001843593 | 10000 | 0.001876534 |
| *IL20* | ENSG00000162891 | 999 | 0.01373132 | 999 | 0.01377337 | 1235.614496 | 0.027504682 | 1259.204866 | 0.027593648 |
| *TXLNB* | ENSG00000164440 | 104.54564 | 0.001883611 | 150.99755 | 0.000703369 | 43.95152129 | 0.004771982 | 69.76428322 | 0.001045786 |
| *INTS8* | ENSG00000164941 | 999 | 0.000277916 | 999 | 0.000277349 | 1166.730292 | 0.001068411 | 1202.015229 | 0.001068153 |
| *CCDC68* | ENSG00000166510 | 64.2973 | 0.02565648 | 49.55501 | 0.02959687 | 30.50836519 | 0.024002292 | 34.96488809 | 0.025362648 |
| *ATF6B* | ENSG00000168468 | 999 | 2.70E-06 | 999 | 5.29E-06 | 983.4066024 | 7.49E-06 | 10000 | 2.35E-05 |
| *FOS* | ENSG00000170345 | 349.88946 | 0.003900343 | 270.89914 | 0.005054056 | 218.0793893 | 0.01172958 | 280.5099531 | 0.013216142 |
| *FGG* | ENSG00000171557 | 81.32581 | 0.01985219 | 77.92909 | 0.02204287 | 51.55223858 | 0.006185461 | 51.41413827 | 0.006963712 |
| *MAB21L3* | ENSG00000173212 | 50.00656 | 0.02098586 | 40.77702 | 0.00513228 | 242.6575136 | 0.034471011 | 116.9847607 | 0.031702653 |
| *SNX31* | ENSG00000174226 | 35.60875 | 0.03625037 | 39.79565 | 0.0320435 | 18.38400296 | 0.045191093 | 19.65939735 | 0.043305301 |
| *ETV4* | ENSG00000175832 | 39.14516 | 0.03821241 | 38.83991 | 0.03736042 | 49.44851631 | 0.041023007 | 55.00559571 | 0.037424067 |
| *C3orf38* | ENSG00000179021 | 795.62884 | 0.00042664 | 828.82219 | 0.001324147 | 749.3280807 | 0.00086755 | 669.3832013 | 0.003092159 |
| *CIITA* | ENSG00000179583 | 999 | 0.004377067 | 999 | 0.004121002 | 6.06617698 | 0.025828708 | 5.326166032 | 0.035302001 |
| *CCDC30* | ENSG00000186409 | 66.28814 | 0.001533656 | 63.59648 | 0.001254711 | 15.51527134 | 0.009832699 | 18.39205658 | 0.00368264 |
| *MAP3K5* | ENSG00000197442 | 30.44879 | 0.009501169 | 29.9407 | 0.009988469 | 21.04586634 | 0.01338845 | 20.83397163 | 0.016207243 |
| *SFI1* | ENSG00000198089 | 104.66706 | 0.02283018 | 73.22475 | 0.03294675 | 75.14655981 | 0.000581387 | 39.87288497 | 0.00219674 |
| *DCLRE1A* | ENSG00000198924 | 70.93647 | 0.02561206 | 69.38497 | 0.02576719 | 27.19655404 | 0.027866728 | 26.8828508 | 0.029460889 |
| *FBXO48* | ENSG00000204923 | 999 | 0.01465098 | 999 | 0.01509985 | 10000000000 | 0.015422727 | 10000000000 | 0.015411097 |
| *IZUMO3* | ENSG00000205442 | 144.91015 | 0.00408329 | 126.17142 | 0.004363347 | 89.95749341 | 0.011890457 | 86.9948211 | 0.012193742 |
| *YAE1* | ENSG00000241127 | 999 | 0.004111984 | 999 | 0.004078825 | 10000 | 0.007475543 | 10000 | 0.004660489 |
| *CFAP206* | ENSG00000272514 | 77.00553 | 0.03175809 | 94.5333 | 0.02354062 | 120.4853164 | 0.03865224 | 254.5524238 | 0.044303132 |
| *MYO19* | ENSG00000278259 | 52.68453 | 0.03391187 | 52.45617 | 0.03385175 | 26.6328386 | 0.031525755 | 29.57984659 | 0.022123885 |

Table S3. Positively selected genes at the branch of Sphenisciformes in the 2-times hypothesis. Threshold of P values is 0.05/3 calculated by a Bonferroni correction. Genes that are annotated as novel in zebra finches but with a particular name in chickens or humans are displayed with brackets.

| Gene name | Ensembl ID | Branch-site model in PAML  (gene tree) | | Branch-site model in PAML  (species tree) | | aBSREL (gene tree) | | aBSREL (species tree) | |
| --- | --- | --- | --- | --- | --- | --- | --- | --- | --- |
|  |  | dN/dS | P-value | dN/dS | P-value | dN/dS | P-value | dN/dS | P-value |
| *PDK4* | ENSTGUG00000001585 | 28.97492 | 0.003568075 | 31.78239 | 0.002655243 | 25.20259509 | 0.004948234 | 38.91526162 | 0.002888977 |
| *LARP7* | ENSTGUG00000001804 | 87.18571 | 0.005526814 | 66.4933 | 0.01004138 | 57.25048837 | 0.000941264 | 33.59773114 | 0.002595621 |
| *CDC14A* | ENSTGUG00000005078 | 554.57111 | 0.000630379 | 545.98594 | 0.000629029 | 555.0567143 | 0.00068238 | 399.2641479 | 0.000719734 |
| *RBP2* | ENSTGUG00000005160 | 198.92141 | 0.007482285 | 207.35714 | 0.00721137 | 10000 | 0.003813847 | 10000 | 0.00378218 |
| *IGF2BP2* (chicken) | ENSTGUG00000006719 | 999 | 0.000301673 | 999 | 0.000366824 | 3333.111126 | 0.000640146 | 10000 | 0.002096287 |
| *PWP1* (chicken) | ENSTGUG00000010969 | 52.15934 | 0.001499007 | 52.66198 | 0.001513204 | 53.42776803 | 0.004926389 | 51.47878522 | 0.005045164 |
| *CPNE3* | ENSTGUG00000011769 | 290.68724 | 8.12E-06 | 260.74967 | 9.68E-06 | 96.84731321 | 0.00028117 | 82.56975263 | 0.000387707 |
| *ITGB1BP1* | ENSTGUG00000013015 | 999 | 0.005539224 | 999 | 0.005177033 | 344.1631552 | 0.000183618 | 253.585693 | 0.001373825 |
| *PRRC2C* | ENSTGUG00000017290 | 999 | 0.000945926 | 999 | 0.001073114 | 1121.028793 | 0.004513974 | 900.4218994 | 0.006952097 |

Table S4. Positively selected genes at the branch of Struthioniformes in the 2-times hypothesis. Threshold of P values is 0.05/3 calculated by a Bonferroni correction. Genes that are annotated as novel in zebra finches but with a particular name in chickens or humans are displayed with brackets.

| Gene name | Ensembl ID | Branch-site model in PAML  (gene tree) | | Branch-site model in PAML  (species tree) | | aBSREL (gene tree) | | aBSREL (species tree) | |
| --- | --- | --- | --- | --- | --- | --- | --- | --- | --- |
|  |  | dN/dS | P-value | dN/dS | P-value | dN/dS | P-value | dN/dS | P-value |
| *MUSK* | ENSTGUG00000001301 | 460.56506 | 0.000758193 | 442.23954 | 0.000556827 | 190.0393237 | 0.009815107 | 208.805251 | 0.007400299 |
| *NCDN* | ENSTGUG00000001681 | 998.99989 | 0.000175637 | 998.99996 | 0.000213972 | 426.1889582 | 0.001439789 | 487.984306 | 0.001263154 |
| *MAP3K3* | ENSTGUG00000001958 | 999 | 8.00E-05 | 998.99984 | 8.36E-05 | 5331.986677 | 0.000310566 | 10000 | 0.000313741 |
| *DDOST* (chicken) | ENSTGUG00000002085 | 999 | 0.000474363 | 998.99994 | 0.000509255 | 10000 | 0.000828312 | 10000 | 0.000974675 |
| *CD40LG* | ENSTGUG00000002108 | 250.56581 | 0.003046627 | 256.24044 | 0.002917493 | 1999.2 | 0.003683946 | 1999.2 | 0.003335402 |
| *GPCPD1* | ENSTGUG00000002182 | 999 | 1.76E-05 | 999 | 0.000188523 | 10000 | 0.002256895 | 5332.216786 | 0.001639884 |
| *NUDT5* | ENSTGUG00000002198 | 373.1439 | 0.000148521 | 575.97739 | 0.000188163 | 391.9103976 | 0.00375264 | 850.8012062 | 0.000828544 |
| *SCIN* (chicken) | ENSTGUG00000002439 | 329.72795 | 0.000908971 | 329.17553 | 0.000912317 | 309.7178544 | 0.000645833 | 341.9949826 | 0.00073109 |
| *SPTLC3* (chicken) | ENSTGUG00000002471 | 999 | 0.000841984 | 999 | 0.002968615 | 10000 | 1.22E-05 | 10000 | 1.24E-05 |
| *KAZN* | ENSTGUG00000002511 | 999 | 1.20E-05 | 999 | 3.04E-05 | 10000 | 1.02E-06 | 10000 | 1.32E-06 |
| *SLC26A11* | ENSTGUG00000003079 | 440.20669 | 2.44E-06 | 437.4213 | 2.29E-06 | 286.4264056 | 0.000104393 | 300.247432 | 0.00012516 |
| *SNAPC3* | ENSTGUG00000004548 | 26.58276 | 0.004516685 | 25.48539 | 0.005005053 | 33.09847904 | 0.005504981 | 32.12841013 | 0.00595646 |
| *DOCK3* (chicken) | ENSTGUG00000004762 | 104.57534 | 0.0134926 | 101.52202 | 0.01481577 | 3333.111126 | 0.001266607 | 258.9192153 | 0.001378504 |
| *SMARCA2* | ENSTGUG00000005460 | 6.59799 | 0.002489093 | 6.56563 | 0.002408748 | 13.16672022 | 0.004919199 | 12.24488468 | 0.004431649 |
| *DPYD* | ENSTGUG00000005495 | 999 | 0.000142387 | 998.99993 | 0.000163767 | 5332.455896 | 0.000199747 | 10000 | 0.000251695 |
| *ELMO2* | ENSTGUG00000005693 | 95.40273 | 0.000120738 | 91.28523 | 0.000146527 | 304.6947552 | 7.88E-05 | 405.9925128 | 6.91E-05 |
| *IRAK4* (chicken) | ENSTGUG00000005932 | 248.31612 | 0.01370827 | 251.40083 | 0.01184991 | 10000 | 0.002126687 | 10000 | 0.002083337 |
| *DIPK2B* | ENSTGUG00000006067 | 82.58606 | 0.00128121 | 84.00917 | 0.000699175 | 25.99631113 | 0.001426607 | 36.25462437 | 0.000791987 |
| *BTBD8* | ENSTGUG00000006099 | 137.30961 | 0.002060274 | 136.53223 | 0.002168626 | 87.13695325 | 0.005000154 | 81.33663181 | 0.005190134 |
| *SLX4IP* | ENSTGUG00000006123 | 12.48968 | 0.01219667 | 12.40231 | 0.01171588 | 21.00175576 | 0.009131347 | 18.99887857 | 0.009248406 |
| *RPAP3* | ENSTGUG00000006201 | 119.26129 | 1.27E-05 | 118.92651 | 1.24E-05 | 1999.2 | 1.03E-05 | 2242.491328 | 1.06E-05 |
| novel gene | ENSTGUG00000006673 | 21.04896 | 0.01320205 | 20.11481 | 0.01529684 | 34.94118459 | 0.006696937 | 29.55266007 | 0.009588588 |
| *IGF2BP2* (chicken) | ENSTGUG00000006719 | 168.72502 | 1.85E-07 | 200.9953 | 3.73E-08 | 152.9577516 | 8.75E-08 | 172.8184382 | 1.17E-08 |
| *CEP63* | ENSTGUG00000006904 | 32.90321 | 0.009513194 | 32.31456 | 0.008019815 | 8.669374041 | 0.007666627 | 9.22046408 | 0.005690253 |
| *SECISBP2L* (chicken) | ENSTGUG00000007252 | 493.52773 | 0.00028203 | 493.17868 | 0.000287034 | 10000 | 0.003123064 | 10000 | 0.003209741 |
| *ANAPC1* | ENSTGUG00000007360 | 40.36253 | 0.000387679 | 39.86006 | 0.000354946 | 32.4599262 | 0.005650757 | 33.28487112 | 0.003784263 |
| *EVPL* (human) | ENSTGUG00000008114 | 23.83324 | 0.000404276 | 24.32775 | 0.000378495 | 111.3771753 | 0.002487448 | 102.1017392 | 0.002515378 |
| *GORASP2* | ENSTGUG00000008120 | 451.06198 | 0.000653176 | 464.07525 | 0.000573354 | 54.53076162 | 0.009127696 | 53.08421634 | 0.009140271 |
| *TLR7* | ENSTGUG00000008250 | 999 | 0.000959509 | 999 | 0.000977846 | 20.95817706 | 0.005757915 | 21.04367555 | 0.004227274 |
| *DNER* | ENSTGUG00000008311 | 223.9697 | 4.07E-05 | 230.05269 | 3.81E-05 | 379.4672454 | 0.000325733 | 374.7514981 | 0.000208497 |
| *EIF2AK1* | ENSTGUG00000008807 | 14.82571 | 0.00137728 | 14.27412 | 0.001833311 | 8.11523478 | 0.006455302 | 8.03272535 | 0.007358291 |
| *ERP44* | ENSTGUG00000008812 | 999 | 0.001259126 | 998.9999 | 0.001262037 | 10000 | 0.001091368 | 10000 | 0.001027958 |
| *DLG1* (chicken) | ENSTGUG00000008892 | 24.77764 | 0.004075133 | 25.01647 | 0.00409756 | 41.43766242 | 0.009280965 | 42.66578409 | 0.009195395 |
| *CCNE1* | ENSTGUG00000008979 | 173.62541 | 0.000471901 | 172.95127 | 0.000475176 | 151.4542267 | 0.000408868 | 156.6320102 | 0.000398136 |
| *FASTKD2* | ENSTGUG00000010004 | 866.58199 | 0.001417054 | 998.99916 | 0.001468184 | 10000 | 0.002422984 | 10000 | 0.002522173 |
| *WBP1L* | ENSTGUG00000010216 | 12.41726 | 0.01314017 | 14.03455 | 0.01261386 | 236.8295877 | 0.001309437 | 202.9681858 | 0.001433952 |
| *SENP5* | ENSTGUG00000010432 | 999 | 7.80E-05 | 999 | 9.28E-05 | 10000 | 6.48E-05 | 10000 | 9.83E-05 |
| *TMCC1* | ENSTGUG00000010564 | 169.87422 | 0.002052993 | 163.40393 | 0.001959742 | 783.9152983 | 0.000602587 | 1061.084576 | 0.000450118 |
| *CKAP5* (chicken) | ENSTGUG00000010577 | 487.00096 | 0.001614057 | 392.53079 | 0.00139176 | 3333.111126 | 0.007919062 | 48.0185917 | 0.002000773 |
| *GTF3C3* | ENSTGUG00000010631 | 999 | 2.30E-05 | 565.03755 | 2.25E-05 | 355.7869753 | 0.000659966 | 383.3385362 | 0.000634295 |
| *DSC1* (human) | ENSTGUG00000010651 | 9.20415 | 0.001187621 | 8.98741 | 0.001076461 | 23.59213936 | 0.000704963 | 23.80426766 | 0.000767007 |
| *AMN1* | ENSTGUG00000011480 | 171.64863 | 0.002290963 | 160.525 | 0.002414418 | 188.2230939 | 0.008808777 | 121.5044239 | 0.008840593 |
| *RALGAPA1* | ENSTGUG00000011986 | 52.59028 | 0.000110097 | 52.61302 | 0.000110235 | 71.32763243 | 0.000172107 | 72.94034714 | 0.00015017 |
| *PPM1A* | ENSTGUG00000012997 | 180.04549 | 0.003248963 | 174.98547 | 0.003312013 | 177.2019263 | 0.002450579 | 162.0195445 | 0.002524145 |
| *CD4* (chicken) | ENSTGUG00000013304 | 24.05838 | 0.003207488 | 23.49339 | 0.003154307 | 7.696572084 | 0.00601781 | 7.533112485 | 0.005893301 |
| *ATP1A1* | ENSTGUG00000013386 | 47.75243 | 0.001823426 | 45.98139 | 0.001891435 | 22.43380323 | 0.001340261 | 26.27030752 | 0.002946844 |
| *PPME1* | ENSTGUG00000013448 | 999 | 0.01272609 | 999 | 0.0124006 | 421.3796625 | 0.0083701 | 399.2641479 | 0.008043297 |
| *SAMSN1* | ENSTGUG00000013543 | 200.83339 | 0.00066974 | 194.14835 | 0.000704132 | 10000 | 0.000339442 | 10000 | 0.00042189 |

Table S5. Positively selected genes at the branch of ratites excluding Struthioniformes in the 2-times hypothesis. Threshold of P values is 0.05/3 calculated by a Bonferroni correction. Genes that are annotated as novel in zebra finches but with a particular name in chickens or humans are displayed with brackets.

| Gene name | Ensembl ID | Branch-site model in PAML  (gene tree) | | Branch-site model in PAML  (species tree) | | aBSREL (gene tree) | | aBSREL (species tree) | |
| --- | --- | --- | --- | --- | --- | --- | --- | --- | --- |
|  |  | dN/dS | P-value | dN/dS | P-value | dN/dS | P-value | dN/dS | P-value |
| *CSTF2* | ENSTGUG00000002253 | 235.83147 | 0.003043459 | 281.02581 | 0.002573382 | 188.6709504 | 0.008202981 | 295.2572624 | 0.003290404 |
| *BTD* (chicken) | ENSTGUG00000003052 | 96.66445 | 0.005852942 | 103.72158 | 0.003844963 | 21.24183647 | 0.007427226 | 22.9348095 | 0.006546268 |
| *METTL7A* | ENSTGUG00000003382 | 999 | 0.001283511 | 999 | 0.001240226 | 813.4855473 | 0.009021527 | 697.5845032 | 0.00832009 |
| *SLC39A8* | ENSTGUG00000003585 | 999 | 0.001955091 | 999 | 0.001966169 | 3430.917055 | 0.003296231 | 3290.003203 | 0.003373813 |
| *TBCK* | ENSTGUG00000003881 | 406.24312 | 1.22E-05 | 399.57658 | 1.46E-05 | 247.6755275 | 3.95E-07 | 245.5306707 | 4.29E-07 |
| *PTPRC* (chicken) | ENSTGUG00000004389 | 236.24673 | 0.00369528 | 177.48293 | 0.004759931 | 10000 | 0.003000147 | 303.9058321 | 0.004709624 |
| *WDR59* | ENSTGUG00000006347 | 999 | 3.39E-06 | 999 | 2.38E-06 | 1574.81697 | 2.71E-07 | 1542.483344 | 3.00E-07 |
| *LONRF1* | ENSTGUG00000007379 | 999 | 1.24E-05 | 999 | 1.31E-05 | 10000 | 5.16E-07 | 10000 | 5.36E-07 |
| *FAH* | ENSTGUG00000008046 | 402.99786 | 0.001585286 | 443.30939 | 0.00153296 | 205.3972817 | 0.007168646 | 245.8673238 | 0.006258319 |
| *SP3* | ENSTGUG00000008690 | 999 | 0.00013187 | 999 | 0.000132934 | 7499.882992 | 0.000443331 | 10000 | 0.000464862 |
| *N4BP2* | ENSTGUG00000008721 | 261.51822 | 3.98E-06 | 250.72126 | 8.97E-07 | 134.241256 | 0.000422772 | 123.3761508 | 0.000533134 |
| *NFE2L2* | ENSTGUG00000009026 | 999 | 0.01223184 | 999 | 0.01399617 | 676.0480323 | 0.002653884 | 927.8759677 | 0.002586902 |
| *EIF2B5* | ENSTGUG00000010097 | 475.01989 | 0.003150234 | 195.06103 | 0.005445744 | 654.697297 | 0.000824007 | 329.535491 | 0.001341541 |
| *RAG1* | ENSTGUG00000010147 | 580.90087 | 0.000684484 | 563.6835 | 0.000890931 | 324.4212256 | 0.00169417 | 357.5409278 | 0.001896987 |
| *CSNK1E* (chicken) | ENSTGUG00000010414 | 999 | 0.000283299 | 999 | 0.000138227 | 10000 | 0.000700395 | 10000 | 0.00035611 |
| *TAF4B* | ENSTGUG00000010600 | 352.62435 | 0.000925512 | 352.97576 | 0.000920999 | 106.977685 | 0.001746888 | 100.798676 | 0.001728106 |
| *PARPBP* | ENSTGUG00000011294 | 791.84018 | 0.004952359 | 780.08515 | 0.004836244 | 1990.989755 | 0.000896504 | 2068.013315 | 0.002797083 |
| *MRPL39* (chicken) | ENSTGUG00000013553 | 270.97234 | 0.003584391 | 287.7145 | 0.003143288 | 220.5016155 | 0.000922971 | 179.202767 | 0.000959074 |

Table S6. Positively selected genes at the branch of Sphenisciformes in the 4-times hypothesis. Threshold of P values is 0.01 calculated by a Bonferroni correction. Genes that are annotated as novel in zebra finches but with a particular name in chickens or humans are displayed with brackets.

| Gene name | Ensembl ID | Branch-site model in PAML  (gene tree) | | Branch-site model in PAML  (species tree) | | aBSREL (gene tree) | | aBSREL (species tree) | |
| --- | --- | --- | --- | --- | --- | --- | --- | --- | --- |
|  |  | dN/dS | P-value | dN/dS | P-value | dN/dS | P-value | dN/dS | P-value |
| *PDK4* | ENSTGUG00000001585 | 28.97412 | 0.003568075 | 31.78278 | 0.002655243 | 25.20259509 | 0.004948234 | 38.91526162 | 0.002890128 |
| *C1GALT1* | ENSTGUG00000001666 | 274.80299 | 0.002626787 | 205.68559 | 0.003797024 | 186.2242619 | 0.003760164 | 139.6605043 | 0.005181303 |
| *LARP7* | ENSTGUG00000001804 | 87.18809 | 0.005526814 | 66.49611 | 0.01004138 | 57.25048837 | 0.000941264 | 33.59773114 | 0.002595621 |
| *RSBN1L* | ENSTGUG00000002712 | 244.41266 | 0.001786906 | 243.03714 | 0.001805547 | 354.7731274 | 0.006178306 | 262.4758108 | 0.007255721 |
| *MARC1* (chicken) | ENSTGUG00000002713 | 999 | 0.005720514 | 999 | 0.007131382 | 10000 | 0.003158938 | 10000 | 0.004544299 |
| *CDC14A* | ENSTGUG00000005078 | 554.11011 | 0.000630378 | 545.62518 | 0.00062903 | 555.0567143 | 0.00068238 | 399.2641479 | 0.000719734 |
| *RBP2* | ENSTGUG00000005160 | 198.92168 | 0.007482285 | 207.35381 | 0.00721137 | 10000 | 0.003813847 | 10000 | 0.00378218 |
| *RGR* (chicken) | ENSTGUG00000005992 | 999 | 0.000333342 | 999 | 0.000455819 | 595.1355265 | 0.001737398 | 308.3195019 | 0.002667626 |
| *IGF2BP2* (chicken) | ENSTGUG00000006719 | 999 | 0.000301672 | 999 | 0.000366824 | 3333.111126 | 0.000640146 | 10000 | 0.002096287 |
| *TMEM100* | ENSTGUG00000009511 | 423.13121 | 0.000764614 | 410.34876 | 0.00078675 | 214.9331456 | 0.001838999 | 233.9510774 | 0.001425016 |
| *C2orf49* | ENSTGUG00000009750 | 999 | 0.01472973 | 998.99988 | 0.01548767 | 10000 | 0.007244345 | 10000 | 0.005902651 |
| *PWP1* (chicken) | ENSTGUG00000010969 | 52.15818 | 0.001499007 | 52.66871 | 0.001513204 | 53.42776803 | 0.004926389 | 51.47878522 | 0.005045164 |
| *C10orf88* (chicken) | ENSTGUG00000011339 | 543.43047 | 0.000370412 | 254.08692 | 0.000357079 | 10000 | 5.72E-05 | 269.0172842 | 8.77E-05 |
| *CPNE3* | ENSTGUG00000011769 | 290.64922 | 8.12E-06 | 260.72606 | 9.68E-06 | 96.84731321 | 0.00028117 | 82.56975263 | 0.000387667 |
| *NR2E1* | ENSTGUG00000012147 | 399.93162 | 0.000570051 | 261.68768 | 0.000892607 | 401.0482018 | 0.001260244 | 261.2738796 | 0.002405711 |
| *SLC30A8* (chicken) | ENSTGUG00000012324 | 998.99993 | 0.00989257 | 998.99992 | 0.0123285 | 2967.995364 | 0.002201199 | 1353.874585 | 0.0064882 |
| *HAS2* (chicken) | ENSTGUG00000012411 | 999 | 8.76E-05 | 999 | 0.000128822 | 10000 | 0.000240488 | 10000 | 0.000373688 |
| *PRRC2C* | ENSTGUG00000017290 | 999 | 0.000945926 | 999 | 0.001090199 | 1121.028793 | 0.004328738 | 900.4218994 | 0.006952172 |

Table S7. Positively selected genes at the branch of Struthioniformes in the 4-times hypothesis. Threshold of P values is 0.01 calculated by a Bonferroni correction. Genes that are annotated as novel in zebra finches but with a particular name in chickens or humans are displayed with brackets.

| Gene name | Ensembl ID | Branch-site model in PAML  (gene tree) | | Branch-site model in PAML  (species tree) | | aBSREL (gene tree) | | aBSREL (species tree) | |
| --- | --- | --- | --- | --- | --- | --- | --- | --- | --- |
|  |  | dN/dS | P-value | dN/dS | P-value | dN/dS | P-value | dN/dS | P-value |
| *MUSK* | ENSTGUG00000001301 | 460.66419 | 0.000758193 | 442.28639 | 0.000556827 | 190.0393237 | 0.009815107 | 208.805251 | 0.007400299 |
| *TRIM36* | ENSTGUG00000001340 | 669.45926 | 0.000219831 | 923.65482 | 8.69E-05 | 3333.111126 | 0.002010073 | 3635.16359 | 0.000483786 |
| *IFT74* | ENSTGUG00000001371 | 64.36256 | 0.000396328 | 71.68289 | 0.000193857 | 15.39499483 | 0.007129339 | 17.15435559 | 0.002654159 |
| *PLXNA2* | ENSTGUG00000001511 | 999 | 1.33E-06 | 998.99949 | 4.00E-07 | 350.8871105 | 7.06E-05 | 539.4490078 | 1.15E-05 |
| *NCDN* | ENSTGUG00000001681 | 998.99996 | 0.000175637 | 998.99994 | 0.000213972 | 426.1889582 | 0.001428122 | 487.984306 | 0.001263162 |
| *MAP3K3* | ENSTGUG00000001958 | 998.99997 | 8.00E-05 | 998.99925 | 8.36E-05 | 5331.986677 | 0.000310566 | 10000 | 0.000313741 |
| *DDOST* (chicken) | ENSTGUG00000002085 | 999 | 0.000474363 | 998.99989 | 0.000509255 | 10000 | 0.000828312 | 10000 | 0.000974675 |
| *CD40LG* | ENSTGUG00000002108 | 265.50328 | 0.003046838 | 239.4366 | 0.002917838 | 1999.2 | 0.003683946 | 1999.2 | 0.003335402 |
| *GPCPD1* | ENSTGUG00000002182 | 999 | 0.00029588 | 999 | 0.000188523 | 10000 | 0.00229345 | 5332.216786 | 0.0016398 |
| *SCIN* (chicken) | ENSTGUG00000002439 | 329.23856 | 0.000908971 | 329.35728 | 0.000912317 | 309.7178544 | 0.000645833 | 341.9949826 | 0.00073109 |
| *SPTLC3* (chicken) | ENSTGUG00000002471 | 998.99989 | 0.000841984 | 998.99998 | 0.002968615 | 10000 | 1.22E-05 | 10000 | 1.24E-05 |
| *CNP* | ENSTGUG00000002478 | 14.98141 | 0.000784943 | 14.02946 | 0.00076851 | 10000 | 0.004397301 | 5355.454251 | 0.003509802 |
| *KAZN* | ENSTGUG00000002511 | 999 | 1.20E-05 | 999 | 3.04E-05 | 10000 | 1.02E-06 | 10000 | 1.32E-06 |
| *EHMT1* (chicken) | ENSTGUG00000002819 | 23.50472 | 0.001885901 | 22.80788 | 0.001997008 | 39.0032464 | 0.007244551 | 39.78399141 | 0.007374449 |
| *SLC26A11* | ENSTGUG00000003079 | 440.18701 | 2.44E-06 | 437.38086 | 2.29E-06 | 286.4264056 | 0.000104393 | 300.247432 | 0.00012516 |
| *ATP1B4* (chicken) | ENSTGUG00000003461 | 111.64608 | 1.83E-07 | 110.39344 | 2.89E-07 | 35.42972925 | 0.000110655 | 36.17329188 | 0.000156926 |
| *ITGB5* | ENSTGUG00000004062 | 279.85338 | 0.00331113 | 503.77783 | 0.002547314 | 261.6108703 | 0.007537578 | 777.3917423 | 0.001566032 |
| *SNAPC3* | ENSTGUG00000004548 | 26.58224 | 0.004516685 | 25.48496 | 0.005005053 | 33.09847904 | 0.005504981 | 32.12841013 | 0.00595646 |
| *DOCK3* (chicken) | ENSTGUG00000004762 | 104.52255 | 0.0134924 | 101.68779 | 0.0148155 | 3333.111126 | 0.001266607 | 258.9192153 | 0.001378504 |
| *SMARCA2* | ENSTGUG00000005460 | 6.60079 | 0.002489093 | 6.56539 | 0.002408748 | 13.16672022 | 0.004919199 | 12.24488468 | 0.004431649 |
| *DPYD* | ENSTGUG00000005495 | 999 | 0.000142387 | 999 | 0.000163767 | 5332.455896 | 0.000199746 | 10000 | 0.000251697 |
| *ELMO2* | ENSTGUG00000005693 | 95.45512 | 0.000120738 | 91.26923 | 0.000146527 | 304.6947552 | 7.88E-05 | 405.9925128 | 6.91E-05 |
| *IRAK4* (chicken) | ENSTGUG00000005932 | 248.21921 | 0.01370827 | 251.75271 | 0.01184991 | 10000 | 0.002126687 | 10000 | 0.002083337 |
| *DIPK2B* | ENSTGUG00000006067 | 82.59248 | 0.00128121 | 84.01227 | 0.000699175 | 25.99631113 | 0.001426607 | 36.25462437 | 0.000791987 |
| *BTBD8* | ENSTGUG00000006099 | 137.30043 | 0.002060272 | 136.50537 | 0.002168624 | 87.13695325 | 0.005000154 | 81.33663181 | 0.005190134 |
| *SLX4IP* | ENSTGUG00000006123 | 12.48962 | 0.01219667 | 12.40231 | 0.01171588 | 21.00175576 | 0.009131347 | 18.99887857 | 0.009248406 |
| *RPAP3* | ENSTGUG00000006201 | 119.28397 | 1.27E-05 | 118.93341 | 1.24E-05 | 1999.2 | 1.03E-05 | 2242.491328 | 1.06E-05 |
| *TRAPPC11* | ENSTGUG00000006590 | 98.61192 | 6.01E-05 | 104.78796 | 4.98E-05 | 120.8818757 | 0.000276828 | 143.5685641 | 0.000258277 |
| novel gene | ENSTGUG00000006673 | 21.04888 | 0.01320205 | 20.11341 | 0.0152976 | 34.94118459 | 0.006696937 | 29.55266007 | 0.009588588 |
| *IGF2BP2* (chicken) | ENSTGUG00000006719 | 168.71343 | 1.85E-07 | 200.99626 | 3.73E-08 | 152.9577516 | 8.75E-08 | 172.8184382 | 1.17E-08 |
| *CEP63* | ENSTGUG00000006904 | 32.90358 | 0.009513194 | 32.31491 | 0.008019815 | 8.669374041 | 0.007666627 | 9.22046408 | 0.005690253 |
| *SECISBP2L* (chicken) | ENSTGUG00000007252 | 493.87032 | 0.00028203 | 494.42396 | 0.000287034 | 10000 | 0.003123064 | 10000 | 0.003209741 |
| *ANAPC1* | ENSTGUG00000007360 | 40.37028 | 0.000387679 | 39.85263 | 0.000354955 | 32.4599262 | 0.005650757 | 33.28487112 | 0.003784263 |
| *MARCH8* | ENSTGUG00000007859 | 301.54341 | 3.37E-07 | 274.97168 | 3.41E-07 | 7494.379487 | 1.73E-06 | 10000 | 1.40E-06 |
| *EVPL* (human) | ENSTGUG00000008114 | 23.83416 | 0.000404258 | 24.31446 | 0.000378512 | 111.3771753 | 0.002487448 | 102.1017392 | 0.002515379 |
| *GORASP2* | ENSTGUG00000008120 | 450.83461 | 0.000653176 | 463.8597 | 0.000573354 | 54.53076162 | 0.009127696 | 53.08421634 | 0.009140271 |
| *NYAP2* | ENSTGUG00000008129 | 999 | 0.008975175 | 999 | 0.009169569 | 10000 | 0.004342434 | 10000 | 0.003317592 |
| *TLR7* | ENSTGUG00000008250 | 999 | 0.001054658 | 999 | 0.000977845 | 20.95817706 | 0.005757915 | 21.04367555 | 0.004227274 |
| *DNER* | ENSTGUG00000008311 | 223.8834 | 4.07E-05 | 230.10723 | 3.81E-05 | 379.4672454 | 0.000325733 | 374.7514981 | 0.000208497 |
| *EIF2AK1* | ENSTGUG00000008807 | 14.82524 | 0.00137728 | 14.27439 | 0.001833311 | 8.11523478 | 0.006455302 | 8.03272535 | 0.007358291 |
| *ERP44* | ENSTGUG00000008812 | 999 | 0.001259126 | 998.99992 | 0.001262037 | 10000 | 0.001091368 | 10000 | 0.001027958 |
| *DLG1* (chicken) | ENSTGUG00000008892 | 24.7759 | 0.004075133 | 25.01843 | 0.00409756 | 41.43766242 | 0.009280965 | 42.66578409 | 0.009195395 |
| *CCNE1* | ENSTGUG00000008979 | 173.62213 | 0.000471901 | 172.95149 | 0.000475176 | 151.4542267 | 0.000408868 | 156.6320102 | 0.000398136 |
| *SLC7A10* (chicken) | ENSTGUG00000009432 | 999 | 6.78E-05 | 999 | 4.34E-05 | 333.8551157 | 0.001207268 | 401.0194984 | 0.000909803 |
| *LSM14A* | ENSTGUG00000009535 | 465.27051 | 2.61E-06 | 999 | 6.12E-07 | 200.7330656 | 1.35E-05 | 338.7304724 | 3.39E-06 |
| *WBP1L* | ENSTGUG00000010216 | 12.41716 | 0.01314017 | 14.03447 | 0.01261386 | 236.8295877 | 0.001309437 | 202.9681858 | 0.001433952 |
| *SENP5* | ENSTGUG00000010432 | 999 | 7.80E-05 | 999 | 9.28E-05 | 10000 | 6.48E-05 | 10000 | 9.83E-05 |
| *TMCC1* | ENSTGUG00000010564 | 169.88859 | 0.002052993 | 163.35435 | 0.001959742 | 783.9152983 | 0.000602617 | 1061.084576 | 0.000450118 |
| *CKAP5* (chicken) | ENSTGUG00000010577 | 487.17007 | 0.001614059 | 392.07057 | 0.001391759 | 3333.111126 | 0.007919062 | 48.0185917 | 0.002000773 |
| *GTF3C3* | ENSTGUG00000010631 | 998.99999 | 2.30E-05 | 556.92026 | 2.25E-05 | 355.7869753 | 0.000659966 | 383.3385362 | 0.000634295 |
| *DSC1* (human) | ENSTGUG00000010651 | 9.20411 | 0.001187621 | 8.98774 | 0.001076461 | 23.59213936 | 0.000704963 | 23.80426766 | 0.000767007 |
| *EIF2A* | ENSTGUG00000011386 | 998.99996 | 0.000135029 | 685.09734 | 0.000100431 | 611.1993992 | 0.004560491 | 372.7175755 | 0.003534236 |
| *AMN1* | ENSTGUG00000011480 | 171.64857 | 0.002290963 | 160.53061 | 0.002414418 | 188.2230939 | 0.008808777 | 121.5044239 | 0.008840593 |
| *RALGAPA1* | ENSTGUG00000011986 | 52.58415 | 0.0001101 | 52.59581 | 0.000110232 | 71.32763243 | 0.000172107 | 72.94034714 | 0.00015017 |
| *VASH1* | ENSTGUG00000012260 | 440.1713 | 6.10E-08 | 481.09927 | 4.25E-08 | 10000 | 4.97E-08 | 10000 | 1.73E-08 |
| *PPM1A* | ENSTGUG00000012997 | 180.03701 | 0.003248963 | 174.99718 | 0.003312013 | 177.2019263 | 0.002450579 | 162.0195445 | 0.002524145 |
| *CD4* (chicken) | ENSTGUG00000013304 | 24.05834 | 0.003207488 | 23.4938 | 0.003154307 | 7.696572084 | 0.00601781 | 7.533112485 | 0.005893301 |
| *ATP1A1* | ENSTGUG00000013386 | 47.74826 | 0.001823426 | 45.96317 | 0.001891437 | 22.43380323 | 0.001340261 | 26.27030752 | 0.002946844 |
| *PPME1* | ENSTGUG00000013448 | 999 | 0.01272609 | 999 | 0.01240057 | 421.3796625 | 0.0083701 | 399.2641479 | 0.008043297 |
| *SAMSN1* | ENSTGUG00000013543 | 200.82587 | 0.00066974 | 194.18634 | 0.000704132 | 10000 | 0.000339442 | 10000 | 0.00042189 |
| *SERINC2* | ENSTGUG00000014321 | 37.09728 | 3.92E-06 | 44.33676 | 3.05E-06 | 31.08794582 | 0.000497869 | 43.49175298 | 0.000513853 |

Table S8. Positively selected genes at the branch of Apterygiformes in the 4-times hypothesis. Threshold of P values is 0.01 calculated by a Bonferroni correction. Genes that are annotated as novel in zebra finches but with a particular name in chickens or humans are displayed with brackets.

| Gene name | Ensembl ID | Branch-site model in PAML  (gene tree) | | Branch-site model in PAML  (species tree) | | aBSREL (gene tree) | | aBSREL (species tree) | |
| --- | --- | --- | --- | --- | --- | --- | --- | --- | --- |
|  |  | dN/dS | P-value | dN/dS | P-value | dN/dS | P-value | dN/dS | P-value |
| *UBLCP1* | ENSTGUG00000000578 | 999 | 5.99E-06 | 432.25783 | 7.36E-06 | 10000 | 1.21E-05 | 10000 | 1.20E-05 |
| *ADGRV1* | ENSTGUG00000000694 | 152.92796 | 8.43E-05 | 144.13624 | 5.76E-05 | 200.6018341 | 3.67E-06 | 166.6422537 | 3.01E-06 |
| *EPB41* | ENSTGUG00000000745 | 999 | 0.000377223 | 999 | 0.000390806 | 10000 | 0.00270838 | 10000 | 0.002631581 |
| *PTAFR* | ENSTGUG00000000879 | 129.8402 | 1.01E-06 | 127.72032 | 1.03E-06 | 73.8994719 | 0.000109783 | 74.86644326 | 0.000127933 |
| *RHCE* (chicken) | ENSTGUG00000001107 | 37.1575 | 0.000127038 | 31.875 | 0.000236454 | 10.5942649 | 0.003357989 | 9.408350747 | 0.004645089 |
| *HSD17B4* | ENSTGUG00000001154 | 814.51455 | 0.003468371 | 810.81178 | 0.00354346 | 10000 | 0.007585372 | 10000 | 0.007341221 |
| *ARHGAP21* | ENSTGUG00000001167 | 8.76434 | 0.00817578 | 8.68766 | 0.008257553 | 34.83922381 | 0.006125109 | 31.2114916 | 0.006165102 |
| *ITGA8* | ENSTGUG00000001333 | 55.60945 | 5.96E-05 | 50.06903 | 0.000134842 | 87.85589609 | 0.000361319 | 73.39723107 | 0.001127195 |
| *P4HA2* | ENSTGUG00000001347 | 998.99995 | 2.66E-06 | 998.99993 | 2.64E-06 | 10000 | 8.99E-05 | 4946.946145 | 0.000101566 |
| *PDZD2* (chicken) | ENSTGUG00000001901 | 639.8928 | 0.000546787 | 584.81586 | 0.000695662 | 325.9027029 | 0.002809658 | 283.925693 | 0.003283664 |
| *PRDM5* | ENSTGUG00000001920 | 92.33638 | 0.002118377 | 92.92092 | 0.002053004 | 391.5265705 | 2.32E-05 | 386.926416 | 2.08E-05 |
| *F13A1* | ENSTGUG00000002276 | 64.72572 | 0.009731538 | 64.79473 | 0.01012465 | 114.6900284 | 0.003148861 | 108.8362567 | 0.003476543 |
| *C1D* | ENSTGUG00000002367 | 999 | 2.20E-05 | 999 | 2.18E-05 | 295.8917897 | 0.000298527 | 339.6894016 | 0.000306999 |
| *BICRAL* | ENSTGUG00000003367 | 154.71119 | 0.000235169 | 152.41175 | 0.000231797 | 386.1641027 | 0.004459265 | 435.9630705 | 0.004291666 |
| *FTCD* | ENSTGUG00000003422 | 999 | 1.08E-05 | 998.99999 | 1.16E-05 | 10000 | 0.000569466 | 10000 | 0.00066524 |
| *LAMP2* (chicken) | ENSTGUG00000003483 | 580.26804 | 9.18E-05 | 999 | 5.84E-12 | 10000 | 0.000639966 | 4946.86995 | 2.30E-12 |
| *SMARCA1* (chicken) | ENSTGUG00000003899 | 162.52482 | 0.001215496 | 159.58733 | 0.001223637 | 83.71405009 | 0.006383339 | 81.64400384 | 0.00599586 |
| *NEO1* | ENSTGUG00000004132 | 170.08256 | 0.000946382 | 135.3946 | 0.001057994 | 769.2898483 | 0.001322118 | 637.4101121 | 0.001605018 |
| *ACBD3* | ENSTGUG00000004214 | 663.98694 | 5.93E-05 | 641.39094 | 6.26E-05 | 10000 | 7.52E-05 | 10000 | 9.21E-05 |
| *ORAI2* | ENSTGUG00000004467 | 999 | 0.000610688 | 999 | 0.000571141 | 10000 | 0.000228376 | 10000 | 0.000185064 |
| *ANAPC16* | ENSTGUG00000004474 | 999 | 0.002882339 | 999 | 0.000176577 | 10000 | 0.002889085 | 10000 | 6.32E-05 |
| *FREM1* | ENSTGUG00000004583 | 139.58372 | 0.00019052 | 133.4017 | 0.000188697 | 89.90326463 | 0.000421595 | 103.0155125 | 0.000334864 |
| *KIF18A* | ENSTGUG00000004750 | 235.77524 | 0.01504909 | 232.91684 | 0.01522667 | 610.1935797 | 0.004184452 | 685.7837485 | 0.004255457 |
| *ERN1* (chicken) | ENSTGUG00000004752 | 999 | 9.20E-05 | 998.99986 | 8.56E-05 | 1466.344745 | 0.000221075 | 10000 | 0.000211488 |
| *KIAA1143* | ENSTGUG00000004815 | 998.99987 | 1.36E-06 | 999 | 1.26E-06 | 262.0081044 | 7.35E-05 | 238.6334011 | 7.13E-05 |
| *OLFM3* | ENSTGUG00000005012 | 999 | 0.000480734 | 999 | 0.000483189 | 10000 | 0.001101896 | 10000 | 0.001077931 |
| *UTP14C* (human) | ENSTGUG00000005130 | 999 | 0.000234849 | 999 | 0.000222682 | 10000 | 0.000772917 | 10000 | 0.000743493 |
| *ADA* | ENSTGUG00000005206 | 69.87627 | 0.004508237 | 52.80712 | 0.006232137 | 78.61129133 | 0.001203603 | 55.87246308 | 0.00188541 |
| *PALMD* | ENSTGUG00000005423 | 499.94192 | 6.73E-05 | 486.29828 | 8.61E-05 | 114.6483125 | 0.000360508 | 107.9943863 | 0.000484427 |
| *ABCC6* | ENSTGUG00000005678 | 26.22151 | 6.24E-05 | 25.69434 | 6.23E-05 | 10000 | 6.50E-05 | 7499.458435 | 6.35E-05 |
| *CBR4* | ENSTGUG00000006085 | 62.72646 | 0.001538935 | 59.05532 | 0.002043941 | 26.57265619 | 0.005207078 | 22.88031271 | 0.006276241 |
| *SETD1A* (chicken) | ENSTGUG00000006093 | 256.72633 | 0.003554882 | 250.12308 | 0.00380346 | 10000 | 0.00073304 | 10000 | 0.000744879 |
| *RFWD3* | ENSTGUG00000006323 | 999 | 0.000649472 | 999 | 0.000359166 | 10000 | 0.002758322 | 10000 | 0.000256265 |
| *ITGB6* | ENSTGUG00000006539 | 151.20944 | 3.89E-18 | 146.71718 | 5.67E-18 | 207.6377865 | 2.78E-16 | 213.1929856 | 2.78E-16 |
| *GRM2* | ENSTGUG00000006595 | 445.76131 | 6.34E-05 | 465.34099 | 5.89E-05 | 264.3790937 | 0.000280477 | 298.6323773 | 0.000346242 |
| *USP4* | ENSTGUG00000006633 | 500.13928 | 0.000804111 | 497.12885 | 0.000775676 | 805.2091333 | 0.002830809 | 772.6822446 | 0.002921679 |
| *DTX4* | ENSTGUG00000007144 | 48.03397 | 0.006526769 | 48.64448 | 0.006211882 | 55.25687703 | 0.008970677 | 60.97303942 | 0.008243368 |
| *ADGRD2* | ENSTGUG00000007153 | 999 | 0.000192874 | 999 | 0.000194219 | 10000 | 0.002818272 | 10000 | 0.002786101 |
| *ADAMTSL3* | ENSTGUG00000007800 | 532.31983 | 4.25E-05 | 556.4239 | 4.72E-05 | 93.72225617 | 0.00054787 | 13.75128783 | 0.000789324 |
| *FAS* (chicken) | ENSTGUG00000008313 | 103.53389 | 0.006862545 | 46.23673 | 0.004722854 | 369.7940808 | 0.002838723 | 551.6454477 | 1.45E-05 |
| *PDZK1IP1* | ENSTGUG00000008532 | 999 | 5.44E-08 | 999 | 8.36E-07 | 10000 | 4.26E-07 | 4946.90289 | 3.25E-05 |
| *BAIAP2L1* | ENSTGUG00000008652 | 999 | 2.81E-09 | 999 | 3.16E-09 | 10000 | 8.49E-08 | 10000 | 1.04E-07 |
| novel gene | ENSTGUG00000008659 | 204.46223 | 0.01113751 | 213.18625 | 0.01053847 | 10000 | 0.00424913 | 10000 | 0.004444726 |
| *EIF2AK1* | ENSTGUG00000008807 | 30.26574 | 0.001048818 | 26.07748 | 0.0052107 | 10000 | 0.001109287 | 10000 | 0.001961714 |
| *SHCBP1* | ENSTGUG00000008933 | 999 | 0.000209507 | 999 | 0.000207479 | 10000 | 0.003525081 | 10000 | 0.003530519 |
| *KLHL5* | ENSTGUG00000008989 | 999 | 1.29E-05 | 999 | 1.14E-05 | 10000 | 8.03E-08 | 10000 | 1.39E-07 |
| *TBC1D1* | ENSTGUG00000009056 | 131.65937 | 0.000853802 | 107.90179 | 0.000957014 | 127.5396078 | 0.00747589 | 99.74557117 | 0.008267633 |
| novel gene | ENSTGUG00000009269 | 281.73472 | 0.00094787 | 333.16357 | 0.000378953 | 543.540887 | 0.000901215 | 541.9834446 | 0.000625904 |
| *C3orf14* | ENSTGUG00000009334 | 999 | 2.41E-06 | 999 | 1.37E-06 | 447.3453426 | 3.01E-07 | 538.5550918 | 1.73E-08 |
| *CYB5RL* (human) | ENSTGUG00000009400 | 89.20171 | 1.43E-05 | 84.92882 | 1.65E-05 | 84.17157879 | 0.000202038 | 81.98916634 | 0.00022641 |
| *GPATCH1* | ENSTGUG00000009401 | 225.82852 | 2.22E-05 | 188.69052 | 4.65E-05 | 293.0515038 | 0.000105142 | 266.9357848 | 0.000235695 |
| *PSMD1* | ENSTGUG00000009697 | 999 | 3.70E-05 | 999 | 3.60E-05 | 10000 | 9.81E-05 | 10000 | 8.80E-05 |
| *MYOM1* | ENSTGUG00000010031 | 201.21021 | 1.03E-08 | 200.59786 | 1.21E-08 | 71.32672619 | 1.69E-06 | 93.04578964 | 3.52E-06 |
| *KIAA1211L* | ENSTGUG00000010153 | 982.50453 | 0.000952843 | 998.9999 | 0.000815142 | 17.28732304 | 0.002726916 | 10000 | 0.001111883 |
| *C2orf69* | ENSTGUG00000010479 | 70.88567 | 1.34E-09 | 66.29067 | 1.49E-09 | 64.64520885 | 3.01E-08 | 55.27720305 | 3.19E-08 |
| *LAMA3* (chicken) | ENSTGUG00000010481 | 999 | 3.70E-19 | 999 | 4.28E-20 | 10000 | 0 | 10000 | 0 |
| novel gene | ENSTGUG00000010550 | 132.05937 | 9.05E-05 | 134.18382 | 9.06E-05 | 10000 | 9.41E-05 | 10000 | 0.000128949 |
| *GLS* | ENSTGUG00000010807 | 632.43856 | 0.000697772 | 629.05525 | 0.000374517 | 3333.086529 | 6.09E-05 | 3332.938277 | 0.000102528 |
| *NOX3* | ENSTGUG00000011124 | 55.00236 | 0.00244895 | 54.0568 | 0.00276597 | 21.62339113 | 0.002658596 | 21.29345485 | 0.002881938 |
| *VEPH1* | ENSTGUG00000011225 | 998.99995 | 0.008837506 | 998.99993 | 0.005743166 | 10000 | 0.00584547 | 10000 | 0.006013593 |
| *IYD* | ENSTGUG00000011323 | 998.99979 | 0.000177065 | 999 | 0.000389614 | 850.6744677 | 2.09E-05 | 720.2735895 | 3.69E-05 |
| *PCNX1* | ENSTGUG00000011329 | 583.00128 | 0.000150251 | 570.02184 | 0.000151672 | 10000 | 0.000378014 | 10000 | 0.000494756 |
| *ACAT2* | ENSTGUG00000011404 | 293.13544 | 6.47E-08 | 266.72699 | 8.23E-08 | 10000 | 1.14E-06 | 10000 | 2.23E-06 |
| *BRCA2* | ENSTGUG00000011763 | 33.20636 | 6.04E-05 | 32.83501 | 6.29E-05 | 35.94639271 | 4.97E-05 | 34.75661985 | 4.71E-05 |
| novel gene | ENSTGUG00000011921 | 39.11616 | 5.65E-09 | 38.7225 | 5.91E-09 | 216.84471 | 9.98E-08 | 222.2009095 | 1.05E-07 |
| *RPF2* | ENSTGUG00000012034 | 128.20896 | 7.53E-05 | 126.59372 | 7.19E-05 | 355.5801701 | 0.000131487 | 288.0508774 | 0.000155602 |
| *STYK1* | ENSTGUG00000012052 | 249.54152 | 1.06E-12 | 213.09033 | 3.11E-12 | 10000 | 1.29E-12 | 10000 | 1.20E-11 |
| *RCBTB2* | ENSTGUG00000012201 | 65.23348 | 0.002227075 | 64.04474 | 0.002217189 | 10000 | 0.000829821 | 10000 | 0.000857496 |
| *PTPRO* | ENSTGUG00000012409 | 999 | 0.006411562 | 999 | 0.006469619 | 1743.224589 | 0.005870318 | 1989.560643 | 0.006493781 |
| *DIAPH3* (chicken) | ENSTGUG00000012460 | 999 | 3.08E-05 | 999 | 2.94E-05 | 10000 | 0.000391839 | 5332.041579 | 0.00040705 |
| *CD109* | ENSTGUG00000012659 | 999 | 2.61E-05 | 999 | 2.60E-05 | 4947.70504 | 9.36E-05 | 4947.722738 | 8.95E-05 |
| *DYNC1H1* | ENSTGUG00000012793 | 999 | 8.22E-07 | 999 | 8.20E-07 | 389.2944784 | 4.61E-05 | 390.0555773 | 4.45E-05 |
| *NOX4* | ENSTGUG00000012893 | 999 | 0.00298029 | 999 | 0.003826564 | 10000 | 0.003047975 | 10000 | 0.002978302 |
| *MFSD2B* | ENSTGUG00000013188 | 410.08838 | 0.000212556 | 408.34841 | 0.000212439 | 402.6698935 | 0.00194213 | 356.1859594 | 0.001880571 |
| *F5* | ENSTGUG00000013426 | 448.36551 | 0.000356467 | 447.24978 | 0.0003255 | 10000 | 0.001126793 | 10000 | 0.001047622 |
| *SOD1* | ENSTGUG00000013463 | 115.93461 | 0.005433879 | 117.90334 | 0.00537464 | 10000 | 0.003914083 | 10000 | 0.004411495 |
| *CEP97* (human) | ENSTGUG00000013617 | 98.5617 | 0.01385723 | 97.92363 | 0.0040359 | 16.65344973 | 0.002138585 | 19.07883695 | 0.001084141 |
| *STARD10* (human) | ENSTGUG00000015143 | 160.52899 | 0.000635031 | 164.63624 | 0.000233361 | 152.6865722 | 0.001035803 | 160.281055 | 0.000710361 |
| *CCDC125* | ENSTGUG00000017531 | 999 | 4.12E-05 | 999 | 4.26E-05 | 10000 | 4.98E-06 | 10000 | 4.78E-06 |

Table S9. Positively selected genes at the branch of Rheiformes in the 4-times hypothesis. Threshold of P values is 0.01 calculated by a Bonferroni correction. Genes that are annotated as novel in zebra finches but with a particular name in chickens or humans are displayed with brackets.

| Gene name | Ensembl ID | Branch-site model in PAML  (gene tree) | | Branch-site model in PAML  (species tree) | | aBSREL (gene tree) | | aBSREL (species tree) | |
| --- | --- | --- | --- | --- | --- | --- | --- | --- | --- |
|  |  | dN/dS | P-value | dN/dS | P-value | dN/dS | P-value | dN/dS | P-value |
| *CPAMD8* | ENSTGUG00000000470 | 6.81117 | 4.57E-07 | 8.30053 | 1.05E-07 | 17.73844403 | 1.39E-07 | 18.86440803 | 3.99E-08 |
| *LARP4B* (chicken) | ENSTGUG00000000811 | 999 | 0.001713094 | 999 | 0.00175337 | 745.0234954 | 0.007160394 | 809.3133199 | 0.006820422 |
| *SLC16A4* | ENSTGUG00000000891 | 13.87921 | 0.005886001 | 8.30331 | 0.01536828 | 21.59611256 | 0.003359215 | 11.83529718 | 0.008791899 |
| *WDTC1* | ENSTGUG00000000973 | 87.13968 | 0.002704338 | 94.04547 | 0.002314277 | 69.84471184 | 0.007355762 | 77.52823828 | 0.005891134 |
| *BMI1* (chicken) | ENSTGUG00000001203 | 999 | 4.15E-05 | 998.99985 | 5.36E-05 | 386.0691736 | 9.16E-05 | 343.4189254 | 0.000116146 |
| *PALM2-AKAP2* | ENSTGUG00000001233 | 25.79267 | 1.40E-12 | 18.0192 | 4.50E-10 | 24.70403852 | 1.47E-09 | 15.20634779 | 5.20E-06 |
| *C7* | ENSTGUG00000002197 | 22.6313 | 0.004872084 | 21.45253 | 0.006431467 | 5.88416313 | 0.002322636 | 5.513107345 | 0.003350414 |
| *GRM3* | ENSTGUG00000002399 | 999 | 0.001726929 | 999 | 0.001717061 | 3333.111126 | 0.008943633 | 3333.111126 | 0.008524472 |
| *DHX29* | ENSTGUG00000002464 | 27.69934 | 1.33E-11 | 21.87092 | 2.29E-09 | 26.66113199 | 7.48E-09 | 14.96614769 | 1.08E-06 |
| *RC3H1* (chicken) | ENSTGUG00000004090 | 13.91397 | 2.09E-05 | 15.16561 | 1.24E-05 | 21.19242739 | 3.72E-06 | 22.65935819 | 2.77E-06 |
| *MGRN1* | ENSTGUG00000004130 | 24.02036 | 0.000137931 | 23.98558 | 0.000190957 | 10000 | 3.30E-05 | 3333.111126 | 3.68E-05 |
| *GAN* | ENSTGUG00000004495 | 998.99988 | 0.000993005 | 998.99989 | 0.001803729 | 3333.111215 | 0.000775033 | 10000 | 0.002175879 |
| *MORC3* | ENSTGUG00000004803 | 45.5369 | 0.000151139 | 45.24615 | 0.000130359 | 10.75385293 | 0.00356021 | 10000 | 0.00233756 |
| *RHOT1* | ENSTGUG00000004857 | 736.46922 | 0.000211474 | 999 | 0.00021891 | 10000 | 7.10E-05 | 10000 | 7.67E-05 |
| *PLA2G10* (chicken) | ENSTGUG00000004955 | 999 | 0.002043656 | 999 | 0.000174116 | 10000 | 0.000160754 | 10000 | 0.000221837 |
| *HNF4A* (chicken) | ENSTGUG00000005271 | 268.83092 | 0.000572828 | 254.25173 | 0.000636204 | 198.6656047 | 0.000502133 | 206.5002357 | 0.000951131 |
| *PPP1R3A* (chicken) | ENSTGUG00000005399 | 15.22865 | 0.001527842 | 13.44148 | 0.002508186 | 5.302508567 | 0.005125844 | 5.608183565 | 0.008197877 |
| *C12orf65* | ENSTGUG00000005440 | 998.99989 | 0.003339066 | 999 | 0.003322185 | 10000 | 0.009067758 | 3333.111126 | 0.008430056 |
| *AVPR2* (chicken) | ENSTGUG00000005614 | 511.26921 | 0.005975891 | 493.17894 | 0.006119279 | 10000 | 0.000750226 | 10000 | 0.000652001 |
| *NAT10* (chicken) | ENSTGUG00000005638 | 263.02169 | 0.00519525 | 266.31674 | 0.005520168 | 3332.893657 | 0.002966521 | 10000 | 0.002894635 |
| *RAB3IL1* | ENSTGUG00000005913 | 998.99931 | 0.01023518 | 998.9994 | 0.008078333 | 10000 | 0.001934824 | 10000 | 0.001283403 |
| *HMGCR* | ENSTGUG00000006083 | 57.82512 | 0.000552769 | 56.73586 | 0.000587818 | 325.8626857 | 0.003088686 | 314.0318787 | 0.003518332 |
| *NFATC3* | ENSTGUG00000007006 | 2.66859 | 0.008503778 | 45.93428 | 3.93E-07 | 9.555161549 | 0.000464336 | 10000 | 4.84E-06 |
| *REPS2* | ENSTGUG00000007806 | 106.92617 | 9.28E-06 | 105.39284 | 1.06E-05 | 111.5786932 | 5.78E-05 | 116.5107582 | 5.60E-05 |
| *CWH43* | ENSTGUG00000007956 | 36.80637 | 1.78E-12 | 55.73805 | 5.91E-12 | 10000 | 8.17E-07 | 10000 | 1.17E-06 |
| *AKR1A1* | ENSTGUG00000007993 | 999 | 0.001968898 | 999 | 0.000413517 | 10000 | 0.000743892 | 10000 | 0.001018299 |
| *ERCC6* | ENSTGUG00000008216 | 41.79069 | 2.41E-19 | 33.3556 | 8.63E-18 | 3333.111037 | 1.49E-05 | 663.1637281 | 2.79E-05 |
| *TNKS2* (chicken) | ENSTGUG00000008510 | 14.29412 | 0.00040474 | 18.19376 | 1.04E-06 | 24.71859268 | 2.99E-08 | 28.8265317 | 8.49E-11 |
| *SOX6* | ENSTGUG00000008522 | 999 | 0.001290371 | 999 | 0.001273517 | 10000 | 0.003678021 | 10000 | 0.003680028 |
| *ELK3* | ENSTGUG00000008619 | 179.223 | 5.22E-07 | 168.8576 | 7.75E-07 | 10000 | 3.32E-06 | 255.256175 | 6.67E-06 |
| *BAIAP2L1* | ENSTGUG00000008652 | 554.12617 | 9.58E-05 | 998.99998 | 9.15E-05 | 117.3517903 | 0.002514599 | 117.3348478 | 0.002585804 |
| *PLOD2* | ENSTGUG00000008767 | 6.1345 | 7.17E-06 | 6.76744 | 1.10E-05 | 14.22227199 | 7.63E-06 | 13.78991243 | 6.77E-06 |
| *SCYL2* | ENSTGUG00000008908 | 31.91075 | 0.000260803 | 57.56434 | 4.43E-07 | 434.0166552 | 4.76E-05 | 35.54981521 | 1.35E-06 |
| *POLR3E* | ENSTGUG00000008986 | 69.19492 | 0.000389216 | 159.23941 | 9.79E-05 | 59.46903566 | 0.002556539 | 99.84828368 | 0.001594538 |
| *IL21R* | ENSTGUG00000009519 | 15.57305 | 1.35E-07 | 17.70385 | 2.90E-08 | 9971.785211 | 0.000114146 | 10000 | 5.65E-05 |
| *C8A* | ENSTGUG00000009598 | 32.07289 | 0.000486831 | 47.42396 | 0.000669741 | 8.78481111 | 0.004986203 | 8.877690389 | 0.004800034 |
| *ARL6IP5* | ENSTGUG00000009784 | 263.70262 | 0.002473758 | 278.76876 | 0.002568221 | 10000 | 0.006815577 | 10000 | 0.005559309 |
| *CARF* | ENSTGUG00000010076 | 28.66189 | 2.21E-08 | 24.86358 | 1.43E-10 | 25.81858182 | 0.000112937 | 28.54299122 | 1.59E-07 |
| *SORCS2* | ENSTGUG00000010129 | 6.53958 | 0.00016891 | 6.95658 | 0.00010042 | 6.172444261 | 0.002257708 | 6.210927784 | 0.001598658 |
| *LIPT1* | ENSTGUG00000010145 | 508.13941 | 0.000323383 | 511.75948 | 0.000321683 | 10000 | 0.006470249 | 3333.111126 | 0.006853313 |
| *GPR137B* | ENSTGUG00000010376 | 999 | 0.000175788 | 999 | 0.000199668 | 10000 | 0.001937754 | 10000 | 0.002353776 |
| *RPAP1* | ENSTGUG00000010870 | 10.35569 | 2.25E-10 | 10.47638 | 1.74E-10 | 8.068409357 | 2.42E-05 | 8.043289638 | 2.58E-05 |
| *TYRO3* | ENSTGUG00000010896 | 9.50779 | 7.72E-07 | 13.69079 | 2.01E-08 | 10.66222518 | 5.62E-06 | 16.37804763 | 8.88E-08 |
| *WDR75* | ENSTGUG00000010903 | 15.76308 | 0.000285655 | 15.50062 | 0.000281564 | 7.093282812 | 0.000519805 | 6.879083151 | 0.000607207 |
| *ADGRG6* | ENSTGUG00000010957 | 10.02471 | 0.000575802 | 17.63503 | 7.71E-08 | 24.37276835 | 1.61E-07 | 23.12935142 | 1.09E-09 |
| *SERAC1* | ENSTGUG00000011073 | 8.5929 | 2.15E-06 | 9.00549 | 4.42E-05 | 662.7244492 | 0.000106925 | 768.8054654 | 0.001103194 |
| *TRPA1* (chicken) | ENSTGUG00000011503 | 42.19113 | 0.001542581 | 42.96409 | 0.001455347 | 38.68501494 | 0.002938827 | 41.46702237 | 0.003832484 |
| *RDH12* (human) | ENSTGUG00000011523 | 998.99991 | 1.80E-06 | 999 | 1.75E-06 | 472.2010458 | 7.12E-06 | 479.3622872 | 7.01E-06 |
| *MKRN1* | ENSTGUG00000011669 | 304.84965 | 0.001381865 | 282.61035 | 0.001538229 | 347.0979679 | 0.002678304 | 304.2058021 | 0.003435727 |
| *SLC37A3* | ENSTGUG00000011676 | 3.16964 | 0.01290429 | 3.38022 | 0.01117251 | 4.191957291 | 0.003332425 | 4.339945348 | 0.002530367 |
| *SLC35A1* | ENSTGUG00000012459 | 998.99992 | 1.06E-08 | 998.99982 | 1.07E-08 | 202.0912334 | 3.05E-07 | 209.9893413 | 2.69E-07 |
| *CEP162* | ENSTGUG00000012509 | 81.28136 | 3.20E-28 | 81.92539 | 4.97E-28 | 10000 | 9.34E-08 | 10000 | 5.36E-08 |
| *SOUL* (chicken) | ENSTGUG00000013230 | 92.96164 | 0.009747273 | 78.48203 | 0.01120722 | 77.83951828 | 0.009708674 | 68.23112469 | 0.009670292 |

Table S10. Positively selected genes at the branch of Casuariiformes in the 4-times hypothesis. Threshold of P values is 0.01 calculated by a Bonferroni correction. Genes that are annotated as novel in zebra finches but with a particular name in chickens or humans are displayed with brackets.

| Gene name | Ensembl ID | Branch-site model in PAML  (gene tree) | | Branch-site model in PAML  (species tree) | | aBSREL (gene tree) | | aBSREL (species tree) | |
| --- | --- | --- | --- | --- | --- | --- | --- | --- | --- |
|  |  | dN/dS | P-value | dN/dS | P-value | dN/dS | P-value | dN/dS | P-value |
| *TCF3* | ENSTGUG00000000208 | 397.78621 | 3.77E-05 | 138.36826 | 0.000349578 | 10000 | 0.000291902 | 10000 | 0.002008524 |
| *VIPR1* (chicken) | ENSTGUG00000000285 | 179.25499 | 0.006170194 | 174.97337 | 0.006214387 | 238.7296883 | 0.004225777 | 251.8755793 | 0.003910572 |
| *MIOS* | ENSTGUG00000001673 | 126.83943 | 0.002028494 | 122.08971 | 0.002085999 | 84.56760467 | 0.003737891 | 74.66019633 | 0.004326813 |
| *KIF6* | ENSTGUG00000002485 | 340.45648 | 0.01255903 | 343.73353 | 0.01225693 | 10000 | 0.000851903 | 10000 | 0.000809752 |
| *EIF4H* | ENSTGUG00000003901 | 591.66279 | 0.001072694 | 581.51627 | 0.001077366 | 373.912328 | 0.004480995 | 346.745501 | 0.004859488 |
| *SCAMP2* | ENSTGUG00000003927 | 124.38966 | 0.003499187 | 102.99127 | 0.00506026 | 64.09958713 | 0.009426818 | 64.41397839 | 0.00908854 |
| *SFMBT1* | ENSTGUG00000005471 | 999 | 0.000575835 | 999 | 0.000609954 | 10000 | 0.008310062 | 10000 | 0.008761033 |
| *GMPR* | ENSTGUG00000005990 | 999 | 0.00016027 | 999 | 0.000223865 | 10000 | 0.000581185 | 10000 | 0.00059605 |
| *AQP9* | ENSTGUG00000006153 | 55.75775 | 0.005887014 | 59.05816 | 0.004736058 | 32.35429261 | 0.004880809 | 36.45090107 | 0.004108138 |
| *LY75* (chicken) | ENSTGUG00000006460 | 23.61024 | 0.0022514 | 22.35839 | 0.002879224 | 10.19782073 | 0.000221776 | 9.575132713 | 0.000265505 |
| *CWH43* | ENSTGUG00000007956 | 999 | 0.00050304 | 999 | 0.002219317 | 10000 | 0.000847125 | 10000 | 0.002051005 |
| *CCDC127* | ENSTGUG00000008296 | 209.51133 | 0.003192487 | 220.74897 | 0.002137828 | 194.0308236 | 0.002669963 | 220.0930158 | 0.002929839 |
| *EXOC3* | ENSTGUG00000008684 | 999 | 0.000640184 | 999 | 0.000710362 | 1999.2 | 0.003408125 | 10000 | 0.004268734 |
| *RELL1* | ENSTGUG00000009141 | 281.35855 | 0.01430264 | 256.48096 | 0.01532409 | 371.0095162 | 0.004930981 | 424.4306561 | 0.004910005 |
| *MGP* | ENSTGUG00000009234 | 18.04989 | 0.00066417 | 12.88854 | 0.00195103 | 33.72119787 | 0.000313151 | 25.20804861 | 0.002509383 |
| *ABCC5* | ENSTGUG00000010048 | 56.20166 | 0.005472383 | 55.7144 | 0.005638877 | 30.79413031 | 0.00284087 | 29.5645195 | 0.002912386 |
| *KIAA1211L* | ENSTGUG00000010153 | 27.56348 | 0.000754246 | 24.32855 | 0.001392442 | 15.98574917 | 0.001206105 | 10.6455039 | 0.007885616 |
| *PDGFB* | ENSTGUG00000010265 | 487.68045 | 0.00175573 | 480.19951 | 0.002139094 | 10000 | 0.00151192 | 10000 | 0.001806146 |
| *IRAK2* (chicken) | ENSTGUG00000010449 | 26.20674 | 0.005417292 | 25.80777 | 0.005363272 | 12.11016176 | 0.0089901 | 12.0502699 | 0.008829544 |
| *ARHGAP11B* (chicken) | ENSTGUG00000011736 | 999 | 1.34E-17 | 999 | 1.10E-16 | 1316.85405 | 4.16E-15 | 1277.603239 | 6.77E-15 |
| *BRCA2* | ENSTGUG00000011763 | 385.35253 | 4.25E-07 | 384.79762 | 4.09E-07 | 10000 | 1.60E-05 | 10000 | 1.35E-05 |
| *COL10A1* (human) | ENSTGUG00000011952 | 243.90047 | 2.73E-05 | 236.05251 | 2.80E-05 | 193.4795638 | 7.95E-05 | 187.9125677 | 8.86E-05 |
| *LRCH1* | ENSTGUG00000012267 | 634.39469 | 0.000100541 | 611.0136 | 0.000114875 | 10000 | 0.001111361 | 1307.258838 | 0.001365361 |
| *GALC* | ENSTGUG00000012397 | 21.04211 | 0.003585956 | 18.89965 | 0.003853453 | 11.4838545 | 0.004580317 | 10.31471244 | 0.005792899 |
| *PANX3* (chicken) | ENSTGUG00000014860 | 220.17641 | 0.000851217 | 191.95676 | 0.001142871 | 219.9062727 | 0.006734463 | 192.528856 | 0.007124986 |

Table S11. The positively selected amino acid substitutions in *KIF18A* detected by the branch-site model in PAML and their estimated impact on protein function. Values that were > 0.95 in BEB, <−2.5 in Provean, and < 0.05 in SIFT are presented in bold; these amino acid changes could either have a remarkable impact on protein function (Provean and SIFT) or have been positively selected (PAML). The positions of amino acids and reference amino acids are shown in human or zebra finch for Chiroptera or Apterygiformes, respectively.

| Branches in interest | Selected sites | Amino acids of zebra finch | Amino acids of target species | BEB probabilities when using gene tree | BEB probabilities when using species tree | Proven scores | SIFT scores |
| --- | --- | --- | --- | --- | --- | --- | --- |
| Chiroptera | 49 | E | Q | 0.836 | 0.791 | **-2.559** | 0.09 |
|  | 338 | F | S | **0.987** | **0.980** | **-3.778** | 1.00 |
|  | 365 | N | H | 0.843 | 0.800 | **-2.917** | **0.03** |
|  | 495 | E | Q | 0.786 | 0.735 | -0.728 | 0.93 |
|  | 635 | L | F | 0.736 | 0.682 | -2.373 | 0.09 |
|  | 642 | G | A | 0.819 | 0.772 | -0.035 | 0.79 |
|  |  |  |  |  |  |  |  |
| Apterygiformes | 75 | F | L | 0.759 | 0.760 | **-5.827** | **0.00** |
|  | 405 | H | R | 0.553 | 0.538 | -0.627 | 0.63 |
|  | 562 | D | D | 0.620 | 0.625 | 0.000 | 0.81 |
|  | 620 | E | E | 0.723 | 0.726 | 0.000 | 1.00 |
|  | 639 | P | S | 0.754 | 0.755 | **-3.602** | 0.76 |
|  | 706 | L | F | 0.747 | 0.749 | -1.906 | 0.23 |
|  | 761 | D | A | 0.765 | 0.766 | **-3.532** | 0.08 |
|  | 785 | T | L | 0.697 | 0.699 | -1.732 | 0.08 |

Table S12. The positively selected amino acid substitutions in *CWH43* detected by the branch-site model in PAML and their estimated impact on protein function. Values that were > 0.95 in BEB, <−2.5 in Provean, and < 0.05 in SIFT are presented in bold; these amino acid changes could either have a remarkable impact on protein function (Provean and SIFT) or have been positively selected (PAML).

| Branches in interest | Selected sites | Amino acids of zebra finch | Amino acids of target species | BEB probabilities when using gene tree | BEB probabilities when using species tree | Proven scores | SIFT scores |
| --- | --- | --- | --- | --- | --- | --- | --- |
| Casuariiformes | 86 | A | T (emu) | 0.501 | 0.524 | **-3.627** | **0.02** |
|  |  |  | – (cassowary) | 0.501 | 0.524 | NA | NA |
|  | 98 | A | V | 0.475 | 0.501 | 0.853 | 0.39 |
|  |  |  | – (cassowary) | 0.475 | 0.501 | NA | NA |
|  | 109 | V | A | 0.504 | 0.526 | **-2.976** | 0.51 |
|  |  |  | – (cassowary) | 0.504 | 0.526 | NA | NA |
|  | 331 | V | A | 0.501 | 0.525 | 0.568 | 0.58 |
|  |  |  | – (cassowary) | 0.501 | 0.525 | NA | NA |
|  | 640 | M | V | 0.613 | 0.631 | -2.088 | 0.09 |
|  | 696 | K | V | **0.983** | **0.984** | **-5.044** | **0.00** |
|  |  |  |  |  |  |  |  |
| Rheiformes | 28 | M | L | 0.741 | 0.744 | **-2.719** | 0.07 |
|  | 29 | I | V | 0.617 | 0.621 | -0.831 | 0.09 |
|  | 30 | Y | L | **0.970** | **0.970** | **-6.019** | **0.01** |
|  | 31 | Y | F | 0.823 | 0.825 | **-2.644** | 0.78 |
|  | 32 | F | G | **0.999** | **0.999** | **-7.407** | **0.00** |
|  | 33 | P | R | 0.828 | 0.828 | **-7.840** | **0.00** |
|  | 34 | L | W | **0.993** | **0.993** | **-5.280** | **0.00** |
|  | 35 | Q | L | 0.883 | 0.886 | **-5.435** | **0.00** |
|  | 37 | L | T | **0.981** | **0.982** | **-4.341** | **0.00** |
|  | 38 | A | S | **0.992** | **0.993** | 0.242 | 0.07 |
|  | 39 | L | I | 0.921 | 0.921 | -1.328 | 0.85 |
|  | 40 | T | S | 0.842 | 0.863 | **-2.577** | 0.38 |
|  | 51 | S | D | **0.997** | **0.997** | **-3.191** | **0.00** |
|  | 52 | P | W | **0.993** | **0.993** | **-9.624** | **0.00** |
|  | 53 | I | L | 0.574 | 0.579 | -1.269 | 0.27 |
|  | 54 | F | W | 0.944 | 0.949 | **-2.762** | **0.00** |
|  | 64 | A | S (greater rhea) | 0.783 | 0.800 | -1.829 | 0.11 |
|  |  |  | G (lesser rhea) | 0.783 | 0.800 | **-2.722** | **0.00** |
|  | 65 | N | S | **0.999** | **0.999** | **-3.239** | 0.09 |
|  | 66 | N | S | **0.999** | **0.999** | -1.440 | 0.27 |
|  | 67 | E | R | 0.601 | 0.609 | 1.150 | 0.42 |
|  | 74 | R | G | 0.662 | 0.663 | **-6.042** | **0.00** |
|  | 75 | L | A | **0.990** | **0.990** | **-3.028** | 0.17 |
|  | 76 | T | S | 0.873 | 0.881 | -1.101 | **0.01** |
|  | 77 | T | F | 0.912 | 0.926 | **-3.880** | **0.01** |
|  | 80 | N | R | 0.762 | 0.770 | -1.899 | **0.01** |
|  | 82 | A | V | 0.756 | 0.760 | **-3.877** | 0.08 |
|  | 83 | S | L | 0.520 | 0.527 | **-5.897** | **0.00** |
|  | 86 | A | Q | **0.995** | **0.996** | **-4.777** | **0.00** |
|  | 87 | P | W | **0.993** | **0.993** | **-9.839** | **0.00** |
|  | 89 | A | K | **0.997** | **0.997** | **-3.925** | **0.01** |
|  | 90 | S | F | 0.611 | 0.621 | -1.844 | 0.73 |
|  | 94 | L | I | 0.807 | 0.816 | 0.251 | 0.45 |
|  | 95 | I | M | 0.581 | 0.589 | -1.652 | 0.10 |
|  | 97 | A | L | **0.992** | **0.992** | **-4.741** | **0.00** |
|  | 99 | G | A | 0.860 | 0.861 | **-5.891** | **0.00** |
|  | 100 | V | S | **0.993** | **0.993** | **-5.134** | **0.03** |
|  | 101 | S | C | 0.763 | 0.763 | **-4.109** | 0.07 |
|  | 106 | V | G | 0.936 | 0.936 | **-6.592** | **0.00** |
|  | 107 | Q | R | **0.975** | **0.974** | **-3.266** | 0.35 |
|  | 108 | T | S | 0.936 | 0.933 | -0.769 | 0.21 |
|  | 109 | V | H | **0.998** | **0.998** | **-6.202** | **0.00** |
|  | 111 | W | F | **0.983** | **0.984** | **-7.923** | 0.09 |
|  | 112 | W | L | **0.961** | **0.961** | **-12.720** | **0.01** |
|  | 113 | S | G | **0.999** | **0.999** | **-3.576** | **0.01** |
|  | 115 | N | Q | **0.975** | **0.978** | **-2.733** | **0.01** |
|  | 117 | L | V | 0.822 | 0.828 | -2.023 | 0.35 |
|  | 118 | Q | G | **0.997** | **0.997** | **-6.380** | **0.01** |
|  | 122 | R | H | **0.998** | **0.998** | **-4.068** | 0.11 |
|  | 124 | W | Q | **0.968** | **0.969** | **-12.259** | **0.00** |
|  | 125 | G | A | 0.824 | 0.825 | **-5.656** | **0.02** |
|  | 126 | F | Q | **0.998** | **0.998** | **-7.631** | **0.01** |
|  | 132 | M | H (greater rhea) | **0.989** | **0.991** | **-4.506** | **0.00** |
|  |  |  | R (lesser rhea) | **0.989** | **0.991** | **-3.697** | **0.01** |
|  | 133 | L | M | 0.904 | **0.953** | -1.754 | 0.05 |
|  | 134 | L | G | **0.999** | **0.999** | **-5.717** | **0.00** |
|  | 137 | R | F | **0.995** | **0.995** | **-7.538** | **0.00** |
|  | 138 | I | C | **0.987** | **0.989** | **-4.625** | **0.00** |
|  | 140 | Y | D | 0.843 | 0.844 | **-8.460** | **0.00** |
|  | 141 | T | W | **0.996** | **0.996** | **-6.229** | **0.00** |
|  | 142 | S | L | **0.955** | **0.954** | **-5.574** | **0.00** |
|  | 144 | N | D | 0.595 | 0.598 | **-4.252** | **0.04** |
|  | 146 | V | C | **0.996** | **0.996** | **-3.803** | **0.02** |
|  | 147 | W | F | **0.983** | **0.984** | **-9.438** | **0.01** |
|  | 148 | S | H | **0.988** | **0.988** | **-2.963** | 0.30 |
|  | 199 | G | P | **0.993** | **0.993** | **-6.493** | **0.00** |
|  | 201 | A | L | **0.992** | **0.993** | **-4.298** | **0.00** |
|  | 202 | F | P | 0.913 | 0.912 | **-8.481** | **0.00** |
|  | 212 | I | H | **0.999** | **0.998** | **-5.593** | **0.00** |
|  | 213 | F | L | 0.499 | 0.505 | **-5.314** | **0.03** |
|  | 214 | G | R | 0.784 | 0.784 | **-7.085** | **0.00** |
|  | 215 | E | L | **0.994** | **0.994** | **-7.087** | **0.00** |
|  | 218 | L | F | 0.763 | 0.733 | **-3.314** | **0.00** |
|  | 219 | I | I | 0.515 | 0.527 | 0.000 | 0.40 |
|  | 220 | S | Y | 0.784 | 0.842 | **-4.334** | **0.01** |
|  | 240 | T | A (greater rhea) | 0.947 | 0.949 | 1.031 | 1.00 |
|  |  |  | X (lesser rhea) | 0.947 | 0.949 | NA | NA |
|  | 241 | V | L | 0.925 | 0.925 | -2.138 | **0.01** |
|  | 244 | G | Q | 0.862 | 0.860 | **-3.695** | **0.03** |
|  | 245 | L | A | **0.982** | **0.982** | **-4.364** | **0.00** |
|  | 270 | L | L | 0.675 | 0.694 | 0.000 | 0.62 |
|  | 271 | A | L | 0.891 | 0.905 | **-3.855** | 0.12 |
|  | 273 | S | V | **0.956** | **0.962** | -1.110 | 0.13 |
|  | 274 | A | C | **0.966** | **0.970** | **-2.712** | 0.14 |
|  | 277 | L | S | **0.996** | **0.996** | **-4.670** | **0.00** |
|  | 278 | Y | W | **0.978** | **0.978** | **-4.149** | **0.00** |
|  | 280 | H | V | **0.984** | **0.984** | **-3.480** | 0.18 |
|  | 290 | I | S | **0.974** | **0.978** | **-4.548** | **0.00** |
|  | 294 | F | C | 0.830 | 0.831 | **-7.209** | **0.00** |
|  | 295 | T | I | 0.580 | 0.585 | **-5.816** | 0.17 |
|  | 296 | M | L | 0.767 | 0.769 | -1.349 | 1.00 |
|  | 297 | C | G | 0.679 | 0.693 | -0.935 | **0.03** |
|  | 298 | V | K | 0.944 | **0.951** | **-3.837** | **0.01** |
|  | 299 | W | L | 0.759 | 0.762 | **-12.559** | **0.00** |
|  | 301 | Q | M | **0.955** | **0.958** | **-3.825** | **0.03** |
|  | 302 | L | F | 0.794 | 0.795 | -2.155 | 0.09 |
|  | 303 | A | L | **0.996** | **0.995** | 2.330 | 1.00 |
|  | 304 | G | N | **0.990** | **0.991** | **-4.132** | 0.19 |
|  | 307 | V | F | 0.566 | 0.577 | **-2.958** | 0.18 |
|  | 308 | S | V | **0.996** | **0.996** | **-4.621** | 0.10 |
|  | 311 | H | Y | 0.485 | 0.507 | -2.464 | 0.30 |
|  | 312 | P | L | 0.811 | 0.813 | **-8.883** | **0.00** |
|  | 315 | A | F | **0.981** | **0.981** | **-3.212** | **0.00** |
|  | 317 | S | V | 0.735 | 0.766 | -1.754 | 0.20 |
|  | 318 | T | R | **0.993** | **0.994** | **-4.142** | **0.03** |
|  | 319 | A | T | 0.584 | 0.586 | **-3.223** | 0.09 |
|  | 320 | M | F | **0.982** | **0.982** | **-4.255** | **0.01** |
|  | 328 | F | T | **0.995** | **0.995** | **-6.821** | **0.00** |
|  | 331 | V | Q | **0.995** | **0.995** | **-2.944** | **0.00** |
|  | 339 | V | D | 0.944 | 0.943 | **-6.826** | **0.00** |
|  | 342 | G | K | **0.965** | **0.965** | **-7.832** | **0.00** |
|  | 344 | Y | L | **0.987** | **0.987** | **-6.927** | **0.09** |
|  | 346 | R | L | **0.985** | **0.985** | **-6.875** | **0.00** |
|  | 347 | E | S | **0.999** | **0.999** | **-4.898** | **0.00** |
|  | 348 | R | K | 0.912 | 0.928 | -1.758 | 0.16 |
|  | 351 | L | Y | **0.973** | **0.973** | -1.232 | 0.34 |
|  | 352 | L | I | 0.913 | 0.912 | -1.513 | 0.09 |
|  | 354 | G | N | **0.994** | **0.994** | **-4.301** | **0.01** |
|  | 355 | F | Y | 0.762 | 0.762 | -0.478 | 0.79 |
|  | 356 | I | V | 0.836 | 0.839 | -0.020 | 1.00 |
|  | 358 | L | K | **0.992** | **0.992** | **-3.212** | **0.01** |
|  | 360 | I | T | 0.762 | 0.763 | **-3.425** | **0.03** |
|  | 362 | V | R | 0.753 | 0.686 | **-2.923** | 0.12 |
|  | 363 | D | S | 0.665 | 0.674 | -0.139 | 0.85 |
|  | 364 | F | W | **0.999** | **0.999** | -1.427 | **0.01** |
|  | 365 | L | G | **0.998** | **0.998** | **-4.671** | 0.06 |
|  | 366 | T | K | 0.684 | 0.693 | -1.579 | 0.38 |
|  | 368 | P | F | 0.516 | 0.528 | **-4.899** | **0.00** |
|  | 369 | K | Q | 0.862 | 0.862 | -1.637 | 0.19 |
|  | 371 | D | E | 0.626 | 0.631 | -1.000 | 0.24 |
|  | 372 | L | T | **0.974** | **0.973** | **-2.748** | 0.19 |
|  | 374 | S | L | **0.954** | **0.955** | 0.338 | 0.67 |
|  | 377 | E | D | 0.534 | 0.557 | -1.285 | 0.33 |
|  | 378 | V | L | 0.799 | 0.802 | -1.058 | 0.35 |
|  | 379 | K | H | **0.989** | **0.989** | **-3.633** | 0.08 |
|  | 380 | S | M | **0.985** | **0.985** | -2.279 | 0.11 |
|  | 381 | N | T | 0.846 | 0.851 | -0.645 | 0.34 |
|  | 382 | Q | M | **0.996** | **0.996** | -1.212 | **0.02** |
|  | 387 | K | E | 0.532 | 0.551 | -1.803 | 0.27 |
|  | 389 | S | D | 0.843 | 0.863 | **-2.627** | **0.03** |
|  | 391 | N | D | 0.646 | 0.646 | -1.135 | 0.12 |
|  | 392 | C | F | 0.653 | 0.670 | **2.954** | 0.40 |
|  | 393 | I | T | 0.761 | 0.763 | -2.435 | **0.02** |
|  | 394 | K | Q | 0.865 | 0.865 | **-2.631** | 0.05 |
|  | 395 | L | I | 0.891 | 0.896 | -1.238 | 0.18 |
|  | 399 | L | H | **0.952** | **0.952** | **-6.080** | **0.01** |
|  | 400 | F | Y | 0.629 | 0.652 | -1.522 | **0.01** |
|  | 401 | V | Q | **0.999** | **0.999** | **-5.122** | **0.00** |
|  | 403 | V | I | 0.541 | 0.557 | -0.535 | 0.29 |
|  | 404 | G | Q | **0.998** | **0.998** | **-6.835** | **0.00** |
|  | 407 | G | N | **0.990** | **0.991** | **-5.169** | **0.00** |
|  | 409 | G | P | **0.997** | **0.997** | **-6.206** | 0.07 |
|  | 410 | L | I | 0.921 | 0.921 | -1.447 | 0.10 |
|  | 411 | R | T | **0.997** | **0.997** | **-5.219** | **0.00** |
|  | 412 | Y | S | 0.849 | 0.850 | **-4.089** | 0.19 |
|  | 413 | K | S | **0.998** | **0.998** | **-3.450** | 0.17 |
|  | 414 | T | H | 0.867 | 0.864 | **-3.699** | 0.11 |
|  | 415 | Y | L | **0.982** | **0.983** | **-6.282** | 0.17 |
|  | 418 | K | R | 0.603 | 0.611 | -2.366 | 0.12 |
|  | 422 | G | H | **0.998** | **0.998** | **-2.752** | 0.11 |
|  | 423 | V | L | 0.898 | 0.903 | -1.089 | 0.29 |
|  | 425 | K | S | **0.999** | **0.999** | -0.264 | 0.46 |
|  | 428 | F | L | 0.802 | 0.802 | -1.333 | 0.06 |
|  | 430 | A | T | 0.503 | 0.508 | **-3.448** | **0.04** |
|  | 441 | N | S | **0.999** | **0.999** | **-4.735** | **0.00** |
|  | 442 | E | Q | 0.849 | 0.853 | -1.856 | 0.19 |
|  | 445 | S | L | 0.818 | 0.833 | **-4.665** | **0.02** |
|  | 446 | N | T | 0.747 | 0.751 | -2.427 | **0.01** |
|  | 455 | N | T | 0.746 | 0.751 | **-3.807** | **0.04** |
|  | 456 | Q | L | 0.918 | 0.922 | **-5.827** | **0.01** |
|  | 458 | E | E | 0.633 | 0.636 | 0.000 | 0.06 |
|  | 459 | A | N | **0.998** | **0.998** | **-5.274** | **0.00** |
|  | 460 | D | S | **0.997** | **0.997** | **-5.282** | **0.03** |
|  | 461 | F | R | **0.999** | **0.999** | **-7.210** | **0.00** |
|  | 462 | I | L | 0.924 | 0.923 | -1.757 | **0.04** |
|  | 463 | T | K | **0.970** | **0.969** | **-4.913** | 0.06 |
|  | 464 | I | L | **0.971** | **0.969** | -1.226 | 0.89 |
|  | 466 | E | Q | 0.863 | 0.863 | **-2.641** | **0.01** |
|  | 484 | G | C | **0.966** | **0.966** | **-7.633** | **0.00** |
|  | 513 | V | F | **0.952** | **0.951** | **-3.807** | **0.00** |
|  | 514 | K | W | **0.996** | **0.996** | **-5.652** | **0.00** |
|  | 515 | S | D | **0.993** | **0.993** | **-3.405** | **0.00** |
|  | 517 | H | S | **0.997** | **0.997** | **-7.619** | **0.00** |
|  | 518 | H | L | 0.853 | 0.855 | **-9.294** | 0.10 |
|  | 519 | L | Q | **0.965** | **0.965** | **-4.990** | **0.00** |
|  | 522 | S | D | **0.993** | **0.993** | **-3.405** | **0.00** |
|  | 525 | G | S | 0.880 | 0.880 | **-5.157** | **0.00** |
|  | 528 | A | K | **0.978** | **0.978** | **-4.181** | **0.00** |
|  | 529 | P | M | **0.996** | **0.996** | **-7.585** | **0.00** |
|  | 531 | I | M | 0.591 | 0.596 | -2.462 | **0.00** |
|  | 533 | L | R | 0.610 | 0.635 | **-3.562** | **0.02** |
|  | 564 | A | L | **0.996** | **0.995** | -2.123 | 0.63 |
|  | 565 | V | T | **0.969** | **0.968** | -2.326 | 0.09 |
|  | 566 | S | V | 0.522 | 0.537 | **-3.588** | **0.00** |
|  | 567 | N | Q | 0.586 | 0.604 | -0.740 | 0.17 |
|  | 568 | L | I | 0.922 | 0.927 | -1.069 | 0.28 |
|  | 621 | I | V | 0.578 | 0.715 | -0.714 | 0.10 |
|  | 622 | R | I | 0.908 | 0.908 | **-6.027** | **0.01** |
|  | 625 | Y | W | **0.978** | **0.978** | **-3.945** | **0.00** |
|  | 627 | R | M | **0.998** | **0.998** | **-4.847** | **0.00** |
|  | 628 | I | P | **0.995** | **0.995** | **-5.716** | **0.00** |
|  | 629 | S | A | 0.937 | 0.937 | -2.239 | **0.00** |
|  | 631 | A | L | **0.985** | **0.985** | **-3.777** | **0.00** |
|  | 632 | G | M | **0.996** | **0.996** | **-2.917** | **0.01** |
|  | 641 | A | Q | **0.999** | **0.999** | **-4.077** | **0.00** |
|  | 643 | F | S | 0.591 | 0.595 | **-6.317** | **0.00** |
|  | 645 | I | W | **0.999** | **0.999** | **-5.781** | **0.00** |
|  | 663 | S | S | **0.989** | **0.990** | 0.000 | 0.53 |
|  | 664 | Q | L | **0.959** | **0.959** | **-3.445** | 0.05 |
|  | 665 | V | I | 0.577 | 0.583 | -0.710 | 0.17 |
|  | 673 | P | A | 0.832 | 0.833 | **-4.801** | 0.06 |
|  | 696 | K | A | 0.575 | 0.851 | **-4.352** | **0.00** |
|  | 697 | Y | S (greater rhea) | 0.634 | 0.653 | **-6.550** | **0.00** |
|  |  |  | L (lesser rhea) | 0.634 | 0.653 | **-5.861** | **0.00** |
|  | 698 | F | L | 0.795 | 0.782 | **-4.362** | **0.00** |
|  | 699 | K | R | 0.639 | 0.639 | -0.371 | **0.00** |

Table S13. The positively selected amino acid substitutions in *EIF2AK1* detected by the branch-site model in PAML and their estimated impact on protein function. Values that were > 0.95 in BEB, <−2.5 in Provean, and < 0.05 in SIFT are presented in bold; these amino acid changes could either have a remarkable impact on protein function (Provean and SIFT) or have been positively selected (PAML).

| Branches in interest | Selected sites | Amino acids of zebra finch | Amino acids of target species | BEB probabilities when using gene tree | BEB probabilities when using species tree | Proven scores | SIFT scores |
| --- | --- | --- | --- | --- | --- | --- | --- |
| Struthioniformes | 18 | V | L | 0.636 | 0.631 | -0.516 | 0.33 |
|  | 64 | L | F | 0.592 | 0.587 | **-3.645** | **0.01** |
|  | 153 | F | S | 0.618 | 0.613 | **-5.324** | 0.09 |
|  | 198 | V | I | 0.534 | 0.529 | -0.900 | **0.01** |
|  | 200 | T | L | 0.672 | 0.642 | -0.664 | 0.26 |
|  | 203 | P | K | **0.978** | **0.977** | **-3.946** | 0.06 |
|  | 204 | K | A | **0.981** | **0.980** | -1.918 | 0.44 |
|  | 299 | T | H | 0.500 | 0.493 | -1.078 | 0.19 |
|  | 369 | E | G | 0.543 | 0.540 | **-2.629** | 0.09 |
|  | 440 | Q | R | 0.922 | 0.918 | -0.628 | 0.63 |
|  | 525 | V | I | 0.501 | 0.499 | 0.170 | 1.00 |
|  |  |  |  |  |  |  |  |
| Apterygiformes | 16 | K | R | **0.980** | 0.890 | -0.630 | 0.37 |
|  | 19 | K | T | 0.734 | 0.717 | 2.110 | 1.00 |
|  | 24 | I | L | 0.950 | 0.943 | -1.705 | **0.02** |
|  | 31 | V | A | 0.662 | 0.644 | **-3.105** | 1.00 |
|  | 44 | N | S | 0.715 | 0.696 | **-3.264** | **0.03** |
|  | 47 | H | R | 0.719 | 0.702 | 0.321 | 1.00 |
|  | 183 | Q | E | 0.721 | 0.703 | **-2.809** | **0.01** |
|  | 232 | E | Q | 0.511 | 0.496 | -1.983 | 0.13 |
|  | 270 | N | S | 0.645 | 0.624 | -0.229 | 0.51 |
|  | 368 | S | R | 0.705 | 0.688 | -0.919 | 0.50 |
|  | 433 | L | H | 0.733 | 0.715 | **-4.912** | **0.01** |
|  | 561 | K | R | 0.710 | 0.690 | **-2.611** | 0.23 |
|  | 582 | - | N | 0.771 | 0.852 | NA | NA |

Table S14. The positively selected amino acid substitutions in *BRCA2* detected by the branch-site model in PAML and their estimated impact on protein function. Values that were > 0.95 in BEB, <−2.5 in Provean, and < 0.05 in SIFT are presented in bold; these amino acid changes could either have a remarkable impact on protein function (Provean and SIFT) or have been positively selected (PAML).

| Branches in interest | Selected sites | Amino acids of zebra finch | Amino acids of target species | BEB probabilities when using gene tree | BEB probabilities when using species tree | Proven scores | SIFT scores |
| --- | --- | --- | --- | --- | --- | --- | --- |
| Casuariiformes | 1290 | E | T | 0.672 | 0.703 | **-4.729** | **0.03** |
|  |  |  |  |  |  |  |  |
| Apterygiformes | 231 | L | S | 0.637 | 0.676 | **-3.374** | 0.09 |
|  | 232 | H | R | 0.704 | 0.741 | **-4.683** | 0.37 |
|  | 1098 | E | Q | 0.511 | 0.513 | -1.477 | 0.22 |
|  | 1195 | T | V | 0.906 | 0.917 | **-3.556** | 0.15 |
|  | 1801 | D | R | 0.501 | 0.529 | -1.301 | 0.14 |
|  | 1810 | Q | E | 0.500 | 0.505 | -2.062 | 0.11 |
|  | 2424 | P | L | 0.500 | 0.503 | **-9.046** | **0.01** |
|  | 2471 | S | A | 0.913 | 0.924 | **-2.649** | **0.02** |
|  | 2787 | A | V | 0.512 | 0.513 | -2.128 | 0.11 |

Table S15. The positively selected amino acid substitutions in *KIAA1211L* detected by the branch-site model in PAML and their estimated impact on protein function. Values that were > 0.95 in BEB, <−2.5 in Provean, and < 0.05 in SIFT are presented in bold; these amino acid changes could either have a remarkable impact on protein function (Provean and SIFT) or have been positively selected (PAML).

| Branches in interest | Selected sites | Amino acids of zebra finch | Amino acids of target species | BEB probabilities when using gene tree | BEB probabilities when using species tree | Proven scores | SIFT scores |
| --- | --- | --- | --- | --- | --- | --- | --- |
| Casuariiformes | 68 | K | A | 0.628 | 0.536 | -0.430 | 0.47 |
|  | 78 | S | Y | 0.905 | 0.866 | **-2.716** | 0.31 |
|  | 80 | K | R | 0.784 | 0.710 | -1.386 | 0.10 |
|  | 126 | S | S | 0.546 | 0.458 | 0.000 | 1.00 |
|  | 131 | S | N | 0.757 | 0.679 | 1.884 | 1.00 |
|  | 156 | G | E | 0.585 | 0.492 | **-5.118** | 0.39 |
|  | 168 | N | G | **0.993** | **0.990** | **-3.288** | 0.06 |
|  | 196 | E | R | **0.992** | **0.988** | **-3.804** | **0.00** |
|  | 205 | S | F | 0.789 | 0.725 | **-5.300** | **0.00** |
|  | 223 | I | L | 0.563 | 0.471 | -0.220 | 0.26 |
|  | 226 | P | L | 0.735 | 0.660 | **-6.392** | 0.13 |
|  |  |  |  |  |  |  |  |
| Apterygiformes | 71 | D | G | 0.749 | 0.794 | **-4.120** | 0.06 |
|  | 135 | V | I | 0.488 | 0.502 | -0.790 | 0.15 |
|  | 270 | D | T | 0.948 | 0.950 | -1.144 | 0.30 |
|  | 277 | P | L | 0.570 | 0.584 | **-8.535** | **0.01** |

Table S16. The positively selected amino acid substitutions in *BAIAP2L1* detected by the branch-site model in PAML and their estimated impact on protein function. Values that were > 0.95 in BEB, <−2.5 in Provean, and < 0.05 in SIFT are presented in bold; these amino acid changes could either have a remarkable impact on protein function (Provean and SIFT) or have been positively selected (PAML).

| Branches in interest | Selected sites | Amino acids of zebra finch | Amino acids of target species | BEB probabilities when using gene tree | BEB probabilities when using species tree | Proven scores | SIFT scores |
| --- | --- | --- | --- | --- | --- | --- | --- |
| Rheiformes | 247 | R | S | 0.517 | 0.384 | **-3.258** | 0.65 |
|  |  |  |  |  |  |  |  |
| Apterygiformes | 214 | C | V | **0.997** | **0.997** | **-3.147** | 0.23 |
|  | 215 | A | S | **1.000** | **1.000** | -1.094 | **0.04** |
|  | 223 | P | H | 0.835 | 0.833 | **-6.703** | **0.01** |

Table S17. GO analysis in Chiroptera using the PANTHER GO-Slim Biological Process. P values are calculated by Fisher’s exact test and FDR values are calculated by the Benjamini–Hochberg procedure.

| GO term | Number of genes in reference | Number of genes in query | P-values | FDR values |
| --- | --- | --- | --- | --- |
| very long-chain fatty acid metabolic process (GO:0000038) | 1 | 1 | 3.33E-02 | 1.00E+00 |
| positive regulation of DNA replication (GO:0045740) | 2 | 1 | 4.95E-02 | 1.00E+00 |
| purine ribonucleoside monophosphate biosynthetic process (GO:0009168) | 3 | 1 | 6.54E-02 | 1.00E+00 |
| purine nucleoside monophosphate biosynthetic process (GO:0009127) | 3 | 1 | 6.54E-02 | 1.00E+00 |
| response to external biotic stimulus (GO:0043207) | 3 | 1 | 6.54E-02 | 1.00E+00 |
| mitochondrial electron transport, cytochrome c to oxygen (GO:0006123) | 3 | 1 | 6.54E-02 | 1.00E+00 |
| defense response to bacterium (GO:0042742) | 3 | 1 | 6.54E-02 | 1.00E+00 |
| response to other organism (GO:0051707) | 3 | 1 | 6.54E-02 | 1.00E+00 |
| heme biosynthetic process (GO:0006783) | 3 | 1 | 6.54E-02 | 1.00E+00 |
| bile acid and bile salt transport (GO:0015721) | 3 | 1 | 6.54E-02 | 1.00E+00 |
| rRNA base methylation (GO:0070475) | 3 | 1 | 6.54E-02 | 1.00E+00 |
| glycosaminoglycan biosynthetic process (GO:0006024) | 3 | 1 | 6.54E-02 | 1.00E+00 |
| response to bacterium (GO:0009617) | 3 | 1 | 6.54E-02 | 1.00E+00 |
| membrane protein proteolysis (GO:0033619) | 3 | 1 | 6.54E-02 | 1.00E+00 |
| cellular response to tumor necrosis factor (GO:0071356) | 3 | 1 | 6.54E-02 | 1.00E+00 |
| aminoglycan biosynthetic process (GO:0006023) | 4 | 1 | 8.11E-02 | 1.00E+00 |
| DNA synthesis involved in DNA repair (GO:0000731) | 4 | 1 | 8.11E-02 | 1.00E+00 |
| response to biotic stimulus (GO:0009607) | 4 | 1 | 8.11E-02 | 1.00E+00 |
| cellular response to radiation (GO:0071478) | 4 | 1 | 8.11E-02 | 1.00E+00 |
| translesion synthesis (GO:0019985) | 4 | 1 | 8.11E-02 | 1.00E+00 |
| regulation of DNA repair (GO:0006282) | 8 | 2 | 1.15E-02 | 1.00E+00 |
| regulation of ion transmembrane transporter activity (GO:0032412) | 4 | 1 | 8.11E-02 | 1.00E+00 |
| protein K63-linked deubiquitination (GO:0070536) | 5 | 1 | 9.65E-02 | 1.00E+00 |
| monocarboxylic acid transport (GO:0015718) | 5 | 1 | 9.65E-02 | 1.00E+00 |
| regulation of sodium ion transport (GO:0002028) | 5 | 1 | 9.65E-02 | 1.00E+00 |
| response to ionizing radiation (GO:0010212) | 5 | 1 | 9.65E-02 | 1.00E+00 |
| regulation of cell division (GO:0051302) | 5 | 1 | 9.65E-02 | 1.00E+00 |
| regulation of dendrite morphogenesis (GO:0048814) | 5 | 1 | 9.65E-02 | 1.00E+00 |
| dendrite morphogenesis (GO:0048813) | 5 | 1 | 9.65E-02 | 1.00E+00 |
| regulation of cytokinesis (GO:0032465) | 5 | 1 | 9.65E-02 | 1.00E+00 |
| sphingolipid biosynthetic process (GO:0030148) | 6 | 1 | 1.12E-01 | 1.00E+00 |
| intra-S DNA damage checkpoint (GO:0031573) | 6 | 1 | 1.12E-01 | 1.00E+00 |
| membrane lipid biosynthetic process (GO:0046467) | 6 | 1 | 1.12E-01 | 1.00E+00 |
| activation of MAPKK activity (GO:0000186) | 6 | 1 | 1.12E-01 | 1.00E+00 |
| regulation of RNA splicing (GO:0043484) | 6 | 1 | 1.12E-01 | 1.00E+00 |
| rRNA methylation (GO:0031167) | 6 | 1 | 1.12E-01 | 1.00E+00 |
| positive regulation of RNA metabolic process (GO:0051254) | 6 | 1 | 1.12E-01 | 1.00E+00 |
| carboxylic acid transport (GO:0046942) | 6 | 1 | 1.12E-01 | 1.00E+00 |
| organic acid transport (GO:0015849) | 6 | 1 | 1.12E-01 | 1.00E+00 |
| double-strand break repair (GO:0006302) | 19 | 3 | 5.57E-03 | 1.00E+00 |
| nucleoside monophosphate biosynthetic process (GO:0009124) | 7 | 1 | 1.27E-01 | 1.00E+00 |
| pigment metabolic process (GO:0042440) | 7 | 1 | 1.27E-01 | 1.00E+00 |
| pigment biosynthetic process (GO:0046148) | 7 | 1 | 1.27E-01 | 1.00E+00 |
| positive regulation of response to DNA damage stimulus (GO:2001022) | 7 | 1 | 1.27E-01 | 1.00E+00 |
| RNA methylation (GO:0001510) | 7 | 1 | 1.27E-01 | 1.00E+00 |
| steroid metabolic process (GO:0008202) | 7 | 1 | 1.27E-01 | 1.00E+00 |
| SCF-dependent proteasomal ubiquitin-dependent protein catabolic process (GO:0031146) | 14 | 2 | 2.86E-02 | 1.00E+00 |
| protein depolymerization (GO:0051261) | 7 | 1 | 1.27E-01 | 1.00E+00 |
| microtubule depolymerization (GO:0007019) | 7 | 1 | 1.27E-01 | 1.00E+00 |
| positive regulation of macromolecule metabolic process (GO:0010604) | 15 | 2 | 3.21E-02 | 1.00E+00 |
| regulation of metal ion transport (GO:0010959) | 8 | 1 | 1.41E-01 | 1.00E+00 |
| telomere capping (GO:0016233) | 8 | 1 | 1.41E-01 | 1.00E+00 |
| histone modification (GO:0016570) | 8 | 1 | 1.41E-01 | 1.00E+00 |
| cellular carbohydrate metabolic process (GO:0044262) | 8 | 1 | 1.41E-01 | 1.00E+00 |
| interstrand cross-link repair (GO:0036297) | 8 | 1 | 1.41E-01 | 1.00E+00 |
| cellular protein complex disassembly (GO:0043624) | 8 | 1 | 1.41E-01 | 1.00E+00 |
| vesicle-mediated transport to the plasma membrane (GO:0098876) | 9 | 1 | 1.56E-01 | 1.00E+00 |
| nucleoside monophosphate metabolic process (GO:0009123) | 9 | 1 | 1.56E-01 | 1.00E+00 |
| positive regulation of gene expression (GO:0010628) | 9 | 1 | 1.56E-01 | 1.00E+00 |
| positive regulation of protein catabolic process (GO:0045732) | 9 | 1 | 1.56E-01 | 1.00E+00 |
| positive regulation of protein serine/threonine kinase activity (GO:0071902) | 9 | 1 | 1.56E-01 | 1.00E+00 |
| mitochondrial ATP synthesis coupled electron transport (GO:0042775) | 9 | 1 | 1.56E-01 | 1.00E+00 |
| ATP synthesis coupled electron transport (GO:0042773) | 9 | 1 | 1.56E-01 | 1.00E+00 |
| negative regulation of transport (GO:0051051) | 9 | 1 | 1.56E-01 | 1.00E+00 |
| regulation of cation transmembrane transport (GO:1904062) | 9 | 1 | 1.56E-01 | 1.00E+00 |
| regulation of ion transmembrane transport (GO:0034765) | 9 | 1 | 1.56E-01 | 1.00E+00 |
| inflammatory response (GO:0006954) | 9 | 1 | 1.56E-01 | 1.00E+00 |
| positive regulation of catabolic process (GO:0009896) | 9 | 1 | 1.56E-01 | 1.00E+00 |
| regulation of response to DNA damage stimulus (GO:2001020) | 10 | 1 | 1.70E-01 | 1.00E+00 |
| positive regulation of NF-kappaB transcription factor activity (GO:0051092) | 10 | 1 | 1.70E-01 | 1.00E+00 |
| endosomal transport (GO:0016197) | 10 | 1 | 1.70E-01 | 1.00E+00 |
| cell division (GO:0051301) | 20 | 2 | 5.17E-02 | 1.00E+00 |
| positive regulation of nucleobase-containing compound metabolic process (GO:0045935) | 10 | 1 | 1.70E-01 | 1.00E+00 |
| ATP synthesis coupled proton transport (GO:0015986) | 10 | 1 | 1.70E-01 | 1.00E+00 |
| energy coupled proton transport, down electrochemical gradient (GO:0015985) | 10 | 1 | 1.70E-01 | 1.00E+00 |
| mitotic DNA damage checkpoint (GO:0044773) | 10 | 1 | 1.70E-01 | 1.00E+00 |
| adherens junction organization (GO:0034332) | 10 | 1 | 1.70E-01 | 1.00E+00 |
| membrane fission (GO:0090148) | 10 | 1 | 1.70E-01 | 1.00E+00 |
| cytokinesis (GO:0000910) | 20 | 2 | 5.17E-02 | 1.00E+00 |
| negative regulation of molecular function (GO:0044092) | 10 | 1 | 1.70E-01 | 1.00E+00 |
| peptide metabolic process (GO:0006518) | 10 | 1 | 1.70E-01 | 1.00E+00 |
| proton transmembrane transport (GO:1902600) | 10 | 1 | 1.70E-01 | 1.00E+00 |
| cell-matrix adhesion (GO:0007160) | 21 | 2 | 5.60E-02 | 1.00E+00 |
| oxidative phosphorylation (GO:0006119) | 11 | 1 | 1.84E-01 | 1.00E+00 |
| cell-substrate adhesion (GO:0031589) | 22 | 2 | 6.05E-02 | 1.00E+00 |
| fatty acid catabolic process (GO:0009062) | 11 | 1 | 1.84E-01 | 1.00E+00 |
| cellular catabolic process (GO:0044248) | 22 | 2 | 6.05E-02 | 1.00E+00 |
| positive regulation of macromolecule biosynthetic process (GO:0010557) | 11 | 1 | 1.84E-01 | 1.00E+00 |
| I-kappaB kinase/NF-kappaB signaling (GO:0007249) | 12 | 1 | 1.98E-01 | 1.00E+00 |
| macromolecule methylation (GO:0043414) | 12 | 1 | 1.98E-01 | 1.00E+00 |
| methylation (GO:0032259) | 12 | 1 | 1.98E-01 | 1.00E+00 |
| ATP metabolic process (GO:0046034) | 12 | 1 | 1.98E-01 | 1.00E+00 |
| fatty acid biosynthetic process (GO:0006633) | 12 | 1 | 1.98E-01 | 1.00E+00 |
| G2/M transition of mitotic cell cycle (GO:0000086) | 12 | 1 | 1.98E-01 | 1.00E+00 |
| regulation of macromolecule metabolic process (GO:0060255) | 36 | 3 | 2.70E-02 | 1.00E+00 |
| cell-cell junction assembly (GO:0007043) | 12 | 1 | 1.98E-01 | 1.00E+00 |
| regulation of DNA metabolic process (GO:0051052) | 25 | 2 | 7.45E-02 | 1.00E+00 |
| purine ribonucleotide biosynthetic process (GO:0009152) | 13 | 1 | 2.11E-01 | 1.00E+00 |
| peptidyl-serine phosphorylation (GO:0018105) | 13 | 1 | 2.11E-01 | 1.00E+00 |
| DNA repair (GO:0006281) | 80 | 6 | 3.01E-03 | 1.00E+00 |
| spermatogenesis (GO:0007283) | 14 | 1 | 2.24E-01 | 1.00E+00 |
| sulfur compound metabolic process (GO:0006790) | 14 | 1 | 2.24E-01 | 1.00E+00 |
| small molecule catabolic process (GO:0044282) | 14 | 1 | 2.24E-01 | 1.00E+00 |
| macromolecule biosynthetic process (GO:0009059) | 14 | 1 | 2.24E-01 | 1.00E+00 |
| positive regulation of protein kinase activity (GO:0045860) | 14 | 1 | 2.24E-01 | 1.00E+00 |
| cell junction organization (GO:0034330) | 14 | 1 | 2.24E-01 | 1.00E+00 |
| activation of protein kinase activity (GO:0032147) | 14 | 1 | 2.24E-01 | 1.00E+00 |
| positive regulation of biosynthetic process (GO:0009891) | 14 | 1 | 2.24E-01 | 1.00E+00 |
| cellular response to DNA damage stimulus (GO:0006974) | 101 | 7 | 2.08E-03 | 1.00E+00 |
| protein-containing complex disassembly (GO:0032984) | 15 | 1 | 2.37E-01 | 1.00E+00 |
| male gamete generation (GO:0048232) | 15 | 1 | 2.37E-01 | 1.00E+00 |
| cellular component disassembly (GO:0022411) | 15 | 1 | 2.37E-01 | 1.00E+00 |
| defense response (GO:0006952) | 15 | 1 | 2.37E-01 | 1.00E+00 |
| regulation of ion transport (GO:0043269) | 16 | 1 | 2.50E-01 | 1.00E+00 |
| purine ribonucleotide metabolic process (GO:0009150) | 16 | 1 | 2.50E-01 | 1.00E+00 |
| vesicle targeting, trans-Golgi to periciliary membrane compartment (GO:0097712) | 16 | 1 | 2.50E-01 | 1.00E+00 |
| ciliary basal body-plasma membrane docking (GO:0097711) | 16 | 1 | 2.50E-01 | 1.00E+00 |
| regulation of protein serine/threonine kinase activity (GO:0071900) | 16 | 1 | 2.50E-01 | 1.00E+00 |
| positive regulation of DNA-binding transcription factor activity (GO:0051091) | 16 | 1 | 2.50E-01 | 1.00E+00 |
| cell-cell adhesion via plasma-membrane adhesion molecules (GO:0098742) | 16 | 1 | 2.50E-01 | 1.00E+00 |
| DNA damage checkpoint (GO:0000077) | 16 | 1 | 2.50E-01 | 1.00E+00 |
| cilium assembly (GO:0060271) | 16 | 1 | 2.50E-01 | 1.00E+00 |
| ribonucleotide biosynthetic process (GO:0009260) | 16 | 1 | 2.50E-01 | 1.00E+00 |
| ciliary transition zone assembly (GO:1905349) | 16 | 1 | 2.50E-01 | 1.00E+00 |
| regulation of cell morphogenesis involved in differentiation (GO:0010769) | 16 | 1 | 2.50E-01 | 1.00E+00 |
| intraciliary transport involved in cilium assembly (GO:0035735) | 16 | 1 | 2.50E-01 | 1.00E+00 |
| axoneme assembly (GO:0035082) | 16 | 1 | 2.50E-01 | 1.00E+00 |
| proteasome-mediated ubiquitin-dependent protein catabolic process (GO:0043161) | 50 | 3 | 5.86E-02 | 1.00E+00 |
| cytokine-mediated signaling pathway (GO:0019221) | 17 | 1 | 2.63E-01 | 1.00E+00 |
| response to radiation (GO:0009314) | 17 | 1 | 2.63E-01 | 1.00E+00 |
| telomere maintenance (GO:0000723) | 17 | 1 | 2.63E-01 | 1.00E+00 |
| ribonucleotide metabolic process (GO:0009259) | 17 | 1 | 2.63E-01 | 1.00E+00 |
| positive regulation of metabolic process (GO:0009893) | 17 | 1 | 2.63E-01 | 1.00E+00 |
| proteasomal protein catabolic process (GO:0010498) | 53 | 3 | 6.69E-02 | 1.00E+00 |
| embryo development (GO:0009790) | 18 | 1 | 2.75E-01 | 1.00E+00 |
| oxoacid metabolic process (GO:0043436) | 18 | 1 | 2.75E-01 | 1.00E+00 |
| response to abiotic stimulus (GO:0009628) | 18 | 1 | 2.75E-01 | 1.00E+00 |
| regulation of cellular response to stress (GO:0080135) | 18 | 1 | 2.75E-01 | 1.00E+00 |
| movement of cell or subcellular component (GO:0006928) | 18 | 1 | 2.75E-01 | 1.00E+00 |
| anatomical structure homeostasis (GO:0060249) | 18 | 1 | 2.75E-01 | 1.00E+00 |
| cell morphogenesis (GO:0000902) | 18 | 1 | 2.75E-01 | 1.00E+00 |
| cellular response to cytokine stimulus (GO:0071345) | 18 | 1 | 2.75E-01 | 1.00E+00 |
| regulation of cell morphogenesis (GO:0022604) | 18 | 1 | 2.75E-01 | 1.00E+00 |
| protein polyubiquitination (GO:0000209) | 19 | 1 | 2.87E-01 | 1.00E+00 |
| regulation of response to stress (GO:0080134) | 19 | 1 | 2.87E-01 | 1.00E+00 |
| regulation of protein kinase activity (GO:0045859) | 19 | 1 | 2.87E-01 | 1.00E+00 |
| cellular response to stress (GO:0033554) | 134 | 7 | 9.02E-03 | 1.00E+00 |
| gamete generation (GO:0007276) | 20 | 1 | 2.99E-01 | 1.00E+00 |
| regulation of protein phosphorylation (GO:0001932) | 20 | 1 | 2.99E-01 | 1.00E+00 |
| DNA biosynthetic process (GO:0071897) | 40 | 2 | 1.56E-01 | 1.00E+00 |
| regulation of gene expression (GO:0010468) | 20 | 1 | 2.99E-01 | 1.00E+00 |
| ribose phosphate metabolic process (GO:0019693) | 20 | 1 | 2.99E-01 | 1.00E+00 |
| organonitrogen compound catabolic process (GO:1901565) | 20 | 1 | 2.99E-01 | 1.00E+00 |
| cellular biosynthetic process (GO:0044249) | 81 | 4 | 5.36E-02 | 1.00E+00 |
| organic substance catabolic process (GO:1901575) | 61 | 3 | 9.15E-02 | 1.00E+00 |
| organic acid metabolic process (GO:0006082) | 21 | 1 | 3.11E-01 | 1.00E+00 |
| tRNA metabolic process (GO:0006399) | 21 | 1 | 3.11E-01 | 1.00E+00 |
| regulation of mRNA processing (GO:0050684) | 22 | 1 | 3.23E-01 | 1.00E+00 |
| fatty acid metabolic process (GO:0006631) | 22 | 1 | 3.23E-01 | 1.00E+00 |
| mitotic sister chromatid segregation (GO:0000070) | 22 | 1 | 3.23E-01 | 1.00E+00 |
| response to cytokine (GO:0034097) | 22 | 1 | 3.23E-01 | 1.00E+00 |
| sexual reproduction (GO:0019953) | 22 | 1 | 3.23E-01 | 1.00E+00 |
| regulation of nucleobase-containing compound metabolic process (GO:0019219) | 69 | 3 | 1.19E-01 | 1.00E+00 |
| protein localization to cilium (GO:0061512) | 23 | 1 | 3.34E-01 | 1.00E+00 |
| alcohol metabolic process (GO:0006066) | 23 | 1 | 3.34E-01 | 1.00E+00 |
| inorganic cation transmembrane transport (GO:0098662) | 23 | 1 | 3.34E-01 | 1.00E+00 |
| sensory perception (GO:0007600) | 23 | 1 | 3.34E-01 | 1.00E+00 |
| biosynthetic process (GO:0009058) | 93 | 4 | 7.90E-02 | 1.00E+00 |
| ion transmembrane transport (GO:0034220) | 24 | 1 | 3.45E-01 | 1.00E+00 |
| protein deubiquitination (GO:0016579) | 24 | 1 | 3.45E-01 | 1.00E+00 |
| regulation of mRNA metabolic process (GO:1903311) | 24 | 1 | 3.45E-01 | 1.00E+00 |
| regulation of transport (GO:0051049) | 24 | 1 | 3.45E-01 | 1.00E+00 |
| inorganic ion transmembrane transport (GO:0098660) | 24 | 1 | 3.45E-01 | 1.00E+00 |
| regulation of catabolic process (GO:0009894) | 24 | 1 | 3.45E-01 | 1.00E+00 |
| positive regulation of transcription by RNA polymerase II (GO:0045944) | 73 | 3 | 1.34E-01 | 1.00E+00 |
| cell adhesion (GO:0007155) | 99 | 4 | 9.37E-02 | 1.00E+00 |
| biological adhesion (GO:0022610) | 99 | 4 | 9.37E-02 | 1.00E+00 |
| monosaccharide metabolic process (GO:0005996) | 25 | 1 | 3.56E-01 | 1.00E+00 |
| proteolysis (GO:0006508) | 50 | 2 | 2.17E-01 | 1.00E+00 |
| DNA replication (GO:0006260) | 52 | 2 | 2.29E-01 | 1.00E+00 |
| catabolic process (GO:0009056) | 80 | 3 | 1.62E-01 | 1.00E+00 |
| cilium organization (GO:0044782) | 27 | 1 | 3.78E-01 | 1.00E+00 |
| microtubule-based movement (GO:0007018) | 27 | 1 | 3.78E-01 | 1.00E+00 |
| DNA metabolic process (GO:0006259) | 54 | 2 | 2.41E-01 | 1.00E+00 |
| regulation of phosphorylation (GO:0042325) | 55 | 2 | 2.48E-01 | 1.00E+00 |
| nucleotide biosynthetic process (GO:0009165) | 28 | 1 | 3.88E-01 | 1.00E+00 |
| vesicle fusion to plasma membrane (GO:0099500) | 28 | 1 | 3.88E-01 | 1.00E+00 |
| drug metabolic process (GO:0017144) | 28 | 1 | 3.88E-01 | 1.00E+00 |
| protein modification by small protein removal (GO:0070646) | 28 | 1 | 3.88E-01 | 1.00E+00 |
| mitotic cell cycle phase transition (GO:0044772) | 28 | 1 | 3.88E-01 | 1.00E+00 |
| cellular macromolecule biosynthetic process (GO:0034645) | 56 | 2 | 2.54E-01 | 1.00E+00 |
| organonitrogen compound metabolic process (GO:1901564) | 57 | 2 | 2.60E-01 | 1.00E+00 |
| translation (GO:0006412) | 86 | 3 | 1.87E-01 | 1.00E+00 |
| regulation of RNA metabolic process (GO:0051252) | 29 | 1 | 3.99E-01 | 1.00E+00 |
| mitotic nuclear division (GO:0140014) | 87 | 3 | 1.91E-01 | 1.00E+00 |
| proteolysis involved in cellular protein catabolic process (GO:0051603) | 87 | 3 | 1.91E-01 | 1.00E+00 |
| cellular protein catabolic process (GO:0044257) | 88 | 3 | 1.96E-01 | 1.00E+00 |
| regulation of cellular metabolic process (GO:0031323) | 148 | 5 | 1.13E-01 | 1.00E+00 |
| mitotic cell cycle (GO:0000278) | 89 | 3 | 2.00E-01 | 1.00E+00 |
| mitotic cell cycle process (GO:1903047) | 89 | 3 | 2.00E-01 | 1.00E+00 |
| mRNA splicing, via spliceosome (GO:0000398) | 61 | 2 | 2.85E-01 | 1.00E+00 |
| RNA splicing, via transesterification reactions with bulged adenosine as nucleophile (GO:0000377) | 61 | 2 | 2.85E-01 | 1.00E+00 |
| positive regulation of protein phosphorylation (GO:0001934) | 31 | 1 | 4.19E-01 | 1.00E+00 |
| plasma membrane fusion (GO:0045026) | 31 | 1 | 4.19E-01 | 1.00E+00 |
| RNA splicing, via transesterification reactions (GO:0000375) | 62 | 2 | 2.91E-01 | 1.00E+00 |
| cell-cell adhesion (GO:0098609) | 31 | 1 | 4.19E-01 | 1.00E+00 |
| organic substance transport (GO:0071702) | 31 | 1 | 4.19E-01 | 1.00E+00 |
| protein catabolic process (GO:0030163) | 94 | 3 | 2.22E-01 | 1.00E+00 |
| macromolecule catabolic process (GO:0009057) | 96 | 3 | 2.31E-01 | 1.00E+00 |
| positive regulation of phosphorylation (GO:0042327) | 32 | 1 | 4.29E-01 | 1.00E+00 |
| lipid metabolic process (GO:0006629) | 32 | 1 | 4.29E-01 | 1.00E+00 |
| negative regulation of biological process (GO:0048519) | 32 | 1 | 4.29E-01 | 1.00E+00 |
| DNA-dependent DNA replication (GO:0006261) | 32 | 1 | 4.29E-01 | 1.00E+00 |
| regulation of phosphorus metabolic process (GO:0051174) | 65 | 2 | 3.10E-01 | 1.00E+00 |
| regulation of phosphate metabolic process (GO:0019220) | 65 | 2 | 3.10E-01 | 1.00E+00 |
| RNA splicing (GO:0008380) | 65 | 2 | 3.10E-01 | 1.00E+00 |
| carboxylic acid catabolic process (GO:0046395) | 33 | 1 | 4.38E-01 | 1.00E+00 |
| organic acid catabolic process (GO:0016054) | 33 | 1 | 4.38E-01 | 1.00E+00 |
| protein metabolic process (GO:0019538) | 70 | 2 | 3.41E-01 | 1.00E+00 |
| cell morphogenesis involved in neuron differentiation (GO:0048667) | 35 | 1 | 4.57E-01 | 1.00E+00 |
| small molecule metabolic process (GO:0044281) | 72 | 2 | 3.53E-01 | 1.00E+00 |
| negative regulation of transcription by RNA polymerase II (GO:0000122) | 36 | 1 | 4.66E-01 | 1.00E+00 |
| membrane fusion (GO:0061025) | 36 | 1 | 4.66E-01 | 1.00E+00 |
| cellular amino acid metabolic process (GO:0006520) | 36 | 1 | 4.66E-01 | 1.00E+00 |
| cellular component morphogenesis (GO:0032989) | 37 | 1 | 4.75E-01 | 1.00E+00 |
| anatomical structure morphogenesis (GO:0009653) | 37 | 1 | 4.75E-01 | 1.00E+00 |
| positive regulation of molecular function (GO:0044093) | 37 | 1 | 4.75E-01 | 1.00E+00 |
| vesicle-mediated transport (GO:0016192) | 149 | 4 | 3.27E-01 | 1.00E+00 |
| formation of translation initiation ternary complex (GO:0001677) | 75 | 2 | 3.72E-01 | 1.00E+00 |
| translational termination (GO:0006415) | 75 | 2 | 3.72E-01 | 1.00E+00 |
| translational elongation (GO:0006414) | 75 | 2 | 3.72E-01 | 1.00E+00 |
| intracellular protein transport (GO:0006886) | 188 | 5 | 2.58E-01 | 1.00E+00 |
| cellular response to stimulus (GO:0051716) | 505 | 13 | 1.45E-01 | 1.00E+00 |
| MAPK cascade (GO:0000165) | 39 | 1 | 4.93E-01 | 1.00E+00 |
| nucleotide metabolic process (GO:0009117) | 40 | 1 | 5.01E-01 | 1.00E+00 |
| regulation of metabolic process (GO:0019222) | 403 | 10 | 2.27E-01 | 1.00E+00 |
| metabolic process (GO:0008152) | 1208 | 29 | 4.24E-02 | 1.00E+00 |
| regulation of cell cycle (GO:0051726) | 42 | 1 | 5.18E-01 | 1.00E+00 |
| protein phosphorylation (GO:0006468) | 84 | 2 | 6.56E-01 | 1.00E+00 |
| RNA processing (GO:0006396) | 84 | 2 | 6.56E-01 | 1.00E+00 |
| membrane organization (GO:0061024) | 84 | 2 | 6.56E-01 | 1.00E+00 |
| regulation of molecular function (GO:0065009) | 85 | 2 | 6.58E-01 | 1.00E+00 |
| primary metabolic process (GO:0044238) | 128 | 3 | 4.84E-01 | 1.00E+00 |
| macromolecule metabolic process (GO:0043170) | 749 | 17 | 2.22E-01 | 1.00E+00 |
| nucleoside phosphate metabolic process (GO:0006753) | 45 | 1 | 5.42E-01 | 1.00E+00 |
| cell cycle (GO:0007049) | 183 | 4 | 5.56E-01 | 1.00E+00 |
| signal transduction by protein phosphorylation (GO:0023014) | 46 | 1 | 5.50E-01 | 1.00E+00 |
| neuron development (GO:0048666) | 46 | 1 | 5.50E-01 | 1.00E+00 |
| G-protein coupled receptor signaling pathway (GO:0007186) | 46 | 1 | 5.50E-01 | 1.00E+00 |
| cellular protein localization (GO:0034613) | 235 | 5 | 6.01E-01 | 1.00E+00 |
| cellular macromolecule localization (GO:0070727) | 237 | 5 | 6.03E-01 | 1.00E+00 |
| nucleobase-containing small molecule metabolic process (GO:0055086) | 49 | 1 | 5.72E-01 | 1.00E+00 |
| gene expression (GO:0010467) | 542 | 11 | 4.87E-01 | 1.00E+00 |
| cellular metabolic process (GO:0044237) | 458 | 9 | 5.74E-01 | 1.00E+00 |
| protein localization to organelle (GO:0033365) | 51 | 1 | 5.87E-01 | 1.00E+00 |
| response to stress (GO:0006950) | 51 | 1 | 5.87E-01 | 1.00E+00 |
| organic substance metabolic process (GO:0071704) | 1020 | 20 | 5.00E-01 | 1.00E+00 |
| intracellular transport (GO:0046907) | 51 | 1 | 5.87E-01 | 1.00E+00 |
| cell surface receptor signaling pathway (GO:0007166) | 154 | 3 | 7.47E-01 | 1.00E+00 |
| neuron differentiation (GO:0030182) | 52 | 1 | 5.94E-01 | 1.00E+00 |
| nucleic acid metabolic process (GO:0090304) | 157 | 3 | 7.51E-01 | 1.00E+00 |
| response to stimulus (GO:0050896) | 211 | 4 | 7.83E-01 | 1.00E+00 |
| regulation of biological process (GO:0050789) | 692 | 13 | 6.39E-01 | 1.00E+00 |
| cellular localization (GO:0051641) | 268 | 5 | 8.07E-01 | 1.00E+00 |
| ncRNA metabolic process (GO:0034660) | 54 | 1 | 6.07E-01 | 1.00E+00 |
| generation of neurons (GO:0048699) | 55 | 1 | 6.14E-01 | 1.00E+00 |
| establishment of localization (GO:0051234) | 331 | 6 | 8.26E-01 | 1.00E+00 |
| transport (GO:0006810) | 331 | 6 | 8.26E-01 | 1.00E+00 |
| signal transduction (GO:0007165) | 396 | 7 | 8.40E-01 | 1.00E+00 |
| carbohydrate metabolic process (GO:0005975) | 57 | 1 | 1.00E+00 | 1.00E+00 |
| response to organic substance (GO:0010033) | 57 | 1 | 1.00E+00 | 1.00E+00 |
| ion transport (GO:0006811) | 57 | 1 | 1.00E+00 | 1.00E+00 |
| intracellular signal transduction (GO:0035556) | 174 | 3 | 1.00E+00 | 1.00E+00 |
| biological regulation (GO:0065007) | 813 | 14 | 1.00E+00 | 1.00E+00 |
| cellular process (GO:0009987) | 1589 | 27 | 1.00E+00 | 1.00E+00 |
| protein ubiquitination (GO:0016567) | 59 | 1 | 1.00E+00 | 1.00E+00 |
| regulation of localization (GO:0032879) | 59 | 1 | 1.00E+00 | 1.00E+00 |
| neurogenesis (GO:0022008) | 59 | 1 | 1.00E+00 | 1.00E+00 |
| transmembrane receptor protein tyrosine kinase signaling pathway (GO:0007169) | 60 | 1 | 1.00E+00 | 1.00E+00 |
| cellular protein modification process (GO:0006464) | 242 | 4 | 1.00E+00 | 1.00E+00 |
| regulation of transcription by RNA polymerase II (GO:0006357) | 182 | 3 | 1.00E+00 | 1.00E+00 |
| cell differentiation (GO:0030154) | 62 | 1 | 1.00E+00 | 1.00E+00 |
| cellular developmental process (GO:0048869) | 62 | 1 | 1.00E+00 | 1.00E+00 |
| regulation of response to stimulus (GO:0048583) | 64 | 1 | 1.00E+00 | 1.00E+00 |
| reproduction (GO:0000003) | 64 | 1 | 1.00E+00 | 1.00E+00 |
| protein modification by small protein conjugation or removal (GO:0070647) | 64 | 1 | 1.00E+00 | 1.00E+00 |
| microtubule-based process (GO:0007017) | 64 | 1 | 1.00E+00 | 1.00E+00 |
| protein modification by small protein conjugation (GO:0032446) | 64 | 1 | 1.00E+00 | 1.00E+00 |
| regulation of transcription, DNA-templated (GO:0006355) | 194 | 3 | 1.00E+00 | 1.00E+00 |
| regulation of nucleic acid-templated transcription (GO:1903506) | 194 | 3 | 1.00E+00 | 1.00E+00 |
| cellular protein metabolic process (GO:0044267) | 266 | 4 | 1.00E+00 | 1.00E+00 |
| cellular macromolecule metabolic process (GO:0044260) | 266 | 4 | 1.00E+00 | 1.00E+00 |
| transcription by RNA polymerase II (GO:0006366) | 266 | 4 | 1.00E+00 | 1.00E+00 |
| transcription, DNA-templated (GO:0006351) | 335 | 5 | 1.00E+00 | 1.00E+00 |
| Unclassified (UNCLASSIFIED) | 2447 | 36 | 2.38E-01 | 1.00E+00 |
| enzyme linked receptor protein signaling pathway (GO:0007167) | 68 | 1 | 1.00E+00 | 1.00E+00 |
| cellular lipid metabolic process (GO:0044255) | 69 | 1 | 1.00E+00 | 1.00E+00 |
| regulation of RNA biosynthetic process (GO:2001141) | 214 | 3 | 1.00E+00 | 1.00E+00 |
| cellular component organization or biogenesis (GO:0071840) | 72 | 1 | 1.00E+00 | 1.00E+00 |
| cellular component biogenesis (GO:0044085) | 72 | 1 | 1.00E+00 | 1.00E+00 |
| regulation of biosynthetic process (GO:0009889) | 219 | 3 | 1.00E+00 | 1.00E+00 |
| regulation of cellular biosynthetic process (GO:0031326) | 219 | 3 | 1.00E+00 | 1.00E+00 |
| multicellular organism development (GO:0007275) | 153 | 2 | 1.00E+00 | 1.00E+00 |
| cellular component organization (GO:0016043) | 461 | 6 | 7.04E-01 | 1.00E+00 |
| nervous system development (GO:0007399) | 77 | 1 | 1.00E+00 | 1.00E+00 |
| carbohydrate derivative metabolic process (GO:1901135) | 78 | 1 | 1.00E+00 | 1.00E+00 |
| immune system process (GO:0002376) | 78 | 1 | 1.00E+00 | 1.00E+00 |
| anatomical structure development (GO:0048856) | 78 | 1 | 1.00E+00 | 1.00E+00 |
| regulation of cellular component organization (GO:0051128) | 79 | 1 | 1.00E+00 | 1.00E+00 |
| localization (GO:0051179) | 563 | 7 | 4.89E-01 | 1.00E+00 |
| nervous system process (GO:0050877) | 85 | 1 | 1.00E+00 | 1.00E+00 |
| multicellular organismal process (GO:0032501) | 269 | 3 | 6.28E-01 | 1.00E+00 |
| regulation of cellular process (GO:0050794) | 269 | 3 | 6.28E-01 | 1.00E+00 |
| homeostatic process (GO:0042592) | 103 | 1 | 1.00E+00 | 1.00E+00 |
| RNA metabolic process (GO:0016070) | 105 | 1 | 1.00E+00 | 1.00E+00 |
| system process (GO:0003008) | 109 | 1 | 1.00E+00 | 1.00E+00 |
| developmental process (GO:0032502) | 110 | 1 | 1.00E+00 | 1.00E+00 |
| response to chemical (GO:0042221) | 110 | 1 | 1.00E+00 | 1.00E+00 |
| system development (GO:0048731) | 123 | 1 | 7.24E-01 | 1.00E+00 |
| regulation of biological quality (GO:0065008) | 176 | 1 | 3.71E-01 | 1.00E+00 |
| organelle organization (GO:0006996) | 232 | 1 | 1.86E-01 | 1.00E+00 |

Table S18. GO analysis in Chiroptera using PANTHER GO-Slim Cellular Component. P values are calculated by Fisher’s exact test and FDR values are calculated by the Benjamini–Hochberg procedure.

| GO term | Number of genes in reference | Number of genes in query | P-values | FDR values |
| --- | --- | --- | --- | --- |
| cytoplasmic side of membrane (GO:0098562) | 1 | 1 | 3.33E-02 | 1.00E+00 |
| side of membrane (GO:0098552) | 1 | 1 | 3.33E-02 | 1.00E+00 |
| Golgi-associated vesicle membrane (GO:0030660) | 2 | 1 | 4.95E-02 | 1.00E+00 |
| midbody (GO:0030496) | 2 | 1 | 4.95E-02 | 1.00E+00 |
| Golgi-associated vesicle (GO:0005798) | 3 | 1 | 6.54E-02 | 1.00E+00 |
| mitochondrial respiratory chain complex IV (GO:0005751) | 3 | 1 | 6.54E-02 | 1.00E+00 |
| spindle midzone (GO:0051233) | 4 | 1 | 8.11E-02 | 1.00E+00 |
| cytoplasmic microtubule (GO:0005881) | 4 | 1 | 8.11E-02 | 1.00E+00 |
| spindle microtubule (GO:0005876) | 5 | 1 | 9.65E-02 | 1.00E+00 |
| SCF ubiquitin ligase complex (GO:0019005) | 13 | 2 | 2.53E-02 | 1.00E+00 |
| integral component of endoplasmic reticulum membrane (GO:0030176) | 14 | 2 | 2.86E-02 | 1.00E+00 |
| mitotic spindle (GO:0072686) | 7 | 1 | 1.27E-01 | 1.00E+00 |
| serine/threonine protein kinase complex (GO:1902554) | 8 | 1 | 1.41E-01 | 1.00E+00 |
| collagen-containing extracellular matrix (GO:0062023) | 8 | 1 | 1.41E-01 | 1.00E+00 |
| early endosome (GO:0005769) | 9 | 1 | 1.56E-01 | 1.00E+00 |
| apical plasma membrane (GO:0016324) | 10 | 1 | 1.70E-01 | 1.00E+00 |
| mediator complex (GO:0016592) | 10 | 1 | 1.70E-01 | 1.00E+00 |
| mitochondrial respiratory chain (GO:0005746) | 10 | 1 | 1.70E-01 | 1.00E+00 |
| cullin-RING ubiquitin ligase complex (GO:0031461) | 21 | 2 | 5.60E-02 | 1.00E+00 |
| core mediator complex (GO:0070847) | 11 | 1 | 1.84E-01 | 1.00E+00 |
| protein kinase complex (GO:1902911) | 11 | 1 | 1.84E-01 | 1.00E+00 |
| transferase complex, transferring phosphorus-containing groups (GO:0061695) | 11 | 1 | 1.84E-01 | 1.00E+00 |
| condensed chromosome (GO:0000793) | 11 | 1 | 1.84E-01 | 1.00E+00 |
| apical part of cell (GO:0045177) | 11 | 1 | 1.84E-01 | 1.00E+00 |
| extrinsic component of plasma membrane (GO:0019897) | 12 | 1 | 1.98E-01 | 1.00E+00 |
| lysosomal membrane (GO:0005765) | 14 | 1 | 2.24E-01 | 1.00E+00 |
| condensed nuclear chromosome (GO:0000794) | 15 | 1 | 2.37E-01 | 1.00E+00 |
| nuclear chromosome, telomeric region (GO:0000784) | 15 | 1 | 2.37E-01 | 1.00E+00 |
| nuclear chromosome part (GO:0044454) | 34 | 2 | 1.22E-01 | 1.00E+00 |
| leaflet of membrane bilayer (GO:0097478) | 35 | 2 | 1.27E-01 | 1.00E+00 |
| transferase complex (GO:1990234) | 35 | 2 | 1.27E-01 | 1.00E+00 |
| histone methyltransferase complex (GO:0035097) | 18 | 1 | 2.75E-01 | 1.00E+00 |
| cell-cell adherens junction (GO:0005913) | 18 | 1 | 2.75E-01 | 1.00E+00 |
| ubiquitin ligase complex (GO:0000151) | 36 | 2 | 1.33E-01 | 1.00E+00 |
| cell surface (GO:0009986) | 57 | 3 | 7.88E-02 | 1.00E+00 |
| spindle (GO:0005819) | 20 | 1 | 2.99E-01 | 1.00E+00 |
| trans-Golgi network (GO:0005802) | 20 | 1 | 2.99E-01 | 1.00E+00 |
| nuclear chromosome (GO:0000228) | 43 | 2 | 1.74E-01 | 1.00E+00 |
| microtubule associated complex (GO:0005875) | 22 | 1 | 3.23E-01 | 1.00E+00 |
| DNA-directed RNA polymerase II, holoenzyme (GO:0016591) | 25 | 1 | 3.56E-01 | 1.00E+00 |
| external side of plasma membrane (GO:0009897) | 26 | 1 | 3.67E-01 | 1.00E+00 |
| cell-cell junction (GO:0005911) | 26 | 1 | 3.67E-01 | 1.00E+00 |
| lytic vacuole (GO:0000323) | 27 | 1 | 3.78E-01 | 1.00E+00 |
| lysosome (GO:0005764) | 27 | 1 | 3.78E-01 | 1.00E+00 |
| mitochondrial inner membrane (GO:0005743) | 28 | 1 | 3.88E-01 | 1.00E+00 |
| spliceosomal complex (GO:0005681) | 30 | 1 | 4.09E-01 | 1.00E+00 |
| cytosol (GO:0005829) | 199 | 6 | 1.58E-01 | 1.00E+00 |
| organelle inner membrane (GO:0019866) | 34 | 1 | 4.48E-01 | 1.00E+00 |
| nuclear DNA-directed RNA polymerase complex (GO:0055029) | 36 | 1 | 4.66E-01 | 1.00E+00 |
| cell junction (GO:0030054) | 36 | 1 | 4.66E-01 | 1.00E+00 |
| cytosolic part (GO:0044445) | 38 | 1 | 4.84E-01 | 1.00E+00 |
| nucleoplasm (GO:0005654) | 80 | 2 | 4.02E-01 | 1.00E+00 |
| organelle envelope (GO:0031967) | 41 | 1 | 5.10E-01 | 1.00E+00 |
| extracellular matrix (GO:0031012) | 41 | 1 | 5.10E-01 | 1.00E+00 |
| nuclear lumen (GO:0031981) | 170 | 4 | 5.38E-01 | 1.00E+00 |
| cilium (GO:0005929) | 43 | 1 | 5.26E-01 | 1.00E+00 |
| nucleoplasm part (GO:0044451) | 45 | 1 | 5.42E-01 | 1.00E+00 |
| Golgi subcompartment (GO:0098791) | 46 | 1 | 5.50E-01 | 1.00E+00 |
| microtubule (GO:0005874) | 48 | 1 | 5.65E-01 | 1.00E+00 |
| centrosome (GO:0005813) | 48 | 1 | 5.65E-01 | 1.00E+00 |
| catalytic complex (GO:1902494) | 98 | 2 | 6.85E-01 | 1.00E+00 |
| intracellular vesicle (GO:0097708) | 49 | 1 | 5.72E-01 | 1.00E+00 |
| cytoplasmic vesicle (GO:0031410) | 49 | 1 | 5.72E-01 | 1.00E+00 |
| plasma membrane protein complex (GO:0098797) | 50 | 1 | 5.80E-01 | 1.00E+00 |
| endosome (GO:0005768) | 51 | 1 | 5.87E-01 | 1.00E+00 |
| intraciliary transport particle (GO:0030990) | 52 | 1 | 5.94E-01 | 1.00E+00 |
| centriole (GO:0005814) | 53 | 1 | 6.01E-01 | 1.00E+00 |
| microtubule organizing center part (GO:0044450) | 54 | 1 | 6.07E-01 | 1.00E+00 |
| protein-containing complex (GO:0032991) | 488 | 9 | 7.16E-01 | 1.00E+00 |
| membrane part (GO:0044425) | 277 | 5 | 8.11E-01 | 1.00E+00 |
| membrane-bounded organelle (GO:0043227) | 169 | 3 | 7.65E-01 | 1.00E+00 |
| nuclear part (GO:0044428) | 282 | 5 | 8.14E-01 | 1.00E+00 |
| membrane (GO:0016020) | 283 | 5 | 8.14E-01 | 1.00E+00 |
| nucleus (GO:0005634) | 681 | 12 | 8.74E-01 | 1.00E+00 |
| vesicle (GO:0031982) | 57 | 1 | 1.00E+00 | 1.00E+00 |
| integral component of membrane (GO:0016021) | 231 | 4 | 1.00E+00 | 1.00E+00 |
| intrinsic component of membrane (GO:0031224) | 232 | 4 | 1.00E+00 | 1.00E+00 |
| Unclassified (UNCLASSIFIED) | 2678 | 46 | 1.00E+00 | 1.00E+00 |
| intracellular membrane-bounded organelle (GO:0043231) | 700 | 12 | 1.00E+00 | 1.00E+00 |
| intracellular organelle (GO:0043229) | 704 | 12 | 1.00E+00 | 1.00E+00 |
| microtubule cytoskeleton (GO:0015630) | 118 | 2 | 1.00E+00 | 1.00E+00 |
| organelle (GO:0043226) | 1137 | 19 | 1.00E+00 | 1.00E+00 |
| microtubule organizing center (GO:0005815) | 60 | 1 | 1.00E+00 | 1.00E+00 |
| cytoskeleton (GO:0005856) | 183 | 3 | 1.00E+00 | 1.00E+00 |
| actin cytoskeleton (GO:0015629) | 61 | 1 | 1.00E+00 | 1.00E+00 |
| cytoplasmic part (GO:0044444) | 637 | 10 | 1.00E+00 | 1.00E+00 |
| cell part (GO:0044464) | 1467 | 23 | 7.21E-01 | 1.00E+00 |
| receptor complex (GO:0043235) | 64 | 1 | 1.00E+00 | 1.00E+00 |
| cell (GO:0005623) | 1473 | 23 | 7.21E-01 | 1.00E+00 |
| cytoplasm (GO:0005737) | 1016 | 15 | 5.91E-01 | 1.00E+00 |
| intracellular non-membrane-bounded organelle (GO:0043232) | 342 | 5 | 1.00E+00 | 1.00E+00 |
| non-membrane-bounded organelle (GO:0043228) | 342 | 5 | 1.00E+00 | 1.00E+00 |
| intracellular (GO:0005622) | 1215 | 17 | 4.46E-01 | 1.00E+00 |
| chromosome (GO:0005694) | 143 | 2 | 1.00E+00 | 1.00E+00 |
| intracellular part (GO:0044424) | 1075 | 15 | 4.29E-01 | 1.00E+00 |
| plasma membrane part (GO:0044459) | 146 | 2 | 1.00E+00 | 1.00E+00 |
| organelle subcompartment (GO:0031984) | 76 | 1 | 1.00E+00 | 1.00E+00 |
| membrane protein complex (GO:0098796) | 78 | 1 | 1.00E+00 | 1.00E+00 |
| extracellular region (GO:0005576) | 165 | 2 | 1.00E+00 | 1.00E+00 |
| plasma membrane (GO:0005886) | 584 | 7 | 3.96E-01 | 1.00E+00 |
| cell periphery (GO:0071944) | 595 | 7 | 3.97E-01 | 1.00E+00 |
| vacuole (GO:0005773) | 340 | 4 | 6.61E-01 | 1.00E+00 |
| integral component of plasma membrane (GO:0005887) | 183 | 2 | 7.71E-01 | 1.00E+00 |
| nuclear chromatin (GO:0000790) | 96 | 1 | 1.00E+00 | 1.00E+00 |
| ribonucleoprotein complex (GO:1990904) | 97 | 1 | 1.00E+00 | 1.00E+00 |
| chromatin (GO:0000785) | 109 | 1 | 1.00E+00 | 1.00E+00 |
| plasma membrane region (GO:0098590) | 126 | 1 | 7.25E-01 | 1.00E+00 |
| chromosomal part (GO:0044427) | 131 | 1 | 7.27E-01 | 1.00E+00 |
| extracellular region part (GO:0044421) | 135 | 1 | 7.29E-01 | 1.00E+00 |
| endomembrane system (GO:0012505) | 223 | 1 | 1.85E-01 | 1.00E+00 |

Table S19. GO analysis in Chiroptera using PANTHER GO-Slim Molecular Function. P values are calculated by Fisher’s exact test and FDR values are calculated by the Benjamini–Hochberg procedure.

| GO term | Number of genes in reference | Number of genes in query | P-values | FDR values |
| --- | --- | --- | --- | --- |
| phosphatidylinositol-3-phosphate binding (GO:0032266) | 3 | 1 | 6.54E-02 | 1.00E+00 |
| activating transcription factor binding (GO:0033613) | 3 | 1 | 6.54E-02 | 1.00E+00 |
| deoxyribonuclease activity (GO:0004536) | 8 | 2 | 1.15E-02 | 1.00E+00 |
| 3'-5' exonuclease activity (GO:0008408) | 4 | 1 | 8.11E-02 | 1.00E+00 |
| cytochrome-c oxidase activity (GO:0004129) | 4 | 1 | 8.11E-02 | 1.00E+00 |
| mismatched DNA binding (GO:0030983) | 5 | 1 | 9.65E-02 | 1.00E+00 |
| 5'-3' exonuclease activity (GO:0008409) | 11 | 2 | 1.92E-02 | 1.00E+00 |
| zinc ion binding (GO:0008270) | 11 | 2 | 1.92E-02 | 1.00E+00 |
| polyubiquitin modification-dependent protein binding (GO:0031593) | 7 | 1 | 1.27E-01 | 1.00E+00 |
| RNA polymerase II core promoter sequence-specific DNA binding (GO:0000979) | 7 | 1 | 1.27E-01 | 1.00E+00 |
| double-stranded RNA binding (GO:0003725) | 7 | 1 | 1.27E-01 | 1.00E+00 |
| protein serine/threonine/tyrosine kinase activity (GO:0004712) | 7 | 1 | 1.27E-01 | 1.00E+00 |
| MAP kinase kinase activity (GO:0004708) | 7 | 1 | 1.27E-01 | 1.00E+00 |
| modification-dependent protein binding (GO:0140030) | 8 | 1 | 1.41E-01 | 1.00E+00 |
| protein kinase activator activity (GO:0030295) | 8 | 1 | 1.41E-01 | 1.00E+00 |
| transition metal ion binding (GO:0046914) | 17 | 2 | 3.95E-02 | 1.00E+00 |
| damaged DNA binding (GO:0003684) | 18 | 2 | 4.34E-02 | 1.00E+00 |
| exonuclease activity (GO:0004527) | 29 | 3 | 1.59E-02 | 1.00E+00 |
| DNA-directed DNA polymerase activity (GO:0003887) | 10 | 1 | 1.70E-01 | 1.00E+00 |
| phosphatidylinositol phosphate binding (GO:1901981) | 10 | 1 | 1.70E-01 | 1.00E+00 |
| ATP-dependent microtubule motor activity, plus-end-directed (GO:0008574) | 10 | 1 | 1.70E-01 | 1.00E+00 |
| dicarboxylic acid transmembrane transporter activity (GO:0005310) | 10 | 1 | 1.70E-01 | 1.00E+00 |
| monocarboxylic acid transmembrane transporter activity (GO:0008028) | 11 | 1 | 1.84E-01 | 1.00E+00 |
| phosphatidylinositol binding (GO:0035091) | 22 | 2 | 6.05E-02 | 1.00E+00 |
| exonuclease activity, active with either ribo- or deoxyribonucleic acids and producing 5'-phosphomonoesters (GO:0016796) | 22 | 2 | 6.05E-02 | 1.00E+00 |
| chloride transmembrane transporter activity (GO:0015108) | 12 | 1 | 1.98E-01 | 1.00E+00 |
| antiporter activity (GO:0015297) | 12 | 1 | 1.98E-01 | 1.00E+00 |
| sodium ion transmembrane transporter activity (GO:0015081) | 28 | 2 | 8.94E-02 | 1.00E+00 |
| hydro-lyase activity (GO:0016836) | 14 | 1 | 2.24E-01 | 1.00E+00 |
| carbon-oxygen lyase activity (GO:0016835) | 14 | 1 | 2.24E-01 | 1.00E+00 |
| growth factor binding (GO:0019838) | 15 | 1 | 2.37E-01 | 1.00E+00 |
| inorganic anion transmembrane transporter activity (GO:0015103) | 15 | 1 | 2.37E-01 | 1.00E+00 |
| monovalent inorganic cation transmembrane transporter activity (GO:0015077) | 46 | 3 | 4.83E-02 | 1.00E+00 |
| carboxylic acid transmembrane transporter activity (GO:0046943) | 32 | 2 | 1.11E-01 | 1.00E+00 |
| solute:cation symporter activity (GO:0015294) | 16 | 1 | 2.50E-01 | 1.00E+00 |
| enzyme activator activity (GO:0008047) | 17 | 1 | 2.63E-01 | 1.00E+00 |
| active transmembrane transporter activity (GO:0022804) | 17 | 1 | 2.63E-01 | 1.00E+00 |
| double-stranded DNA binding (GO:0003690) | 17 | 1 | 2.63E-01 | 1.00E+00 |
| secondary active transmembrane transporter activity (GO:0015291) | 17 | 1 | 2.63E-01 | 1.00E+00 |
| DNA polymerase activity (GO:0034061) | 18 | 1 | 2.75E-01 | 1.00E+00 |
| RNA methyltransferase activity (GO:0008173) | 18 | 1 | 2.75E-01 | 1.00E+00 |
| organic anion transmembrane transporter activity (GO:0008514) | 36 | 2 | 1.33E-01 | 1.00E+00 |
| anion transmembrane transporter activity (GO:0008509) | 36 | 2 | 1.33E-01 | 1.00E+00 |
| protein kinase regulator activity (GO:0019887) | 18 | 1 | 2.75E-01 | 1.00E+00 |
| protein homodimerization activity (GO:0042803) | 18 | 1 | 2.75E-01 | 1.00E+00 |
| nuclease activity (GO:0004518) | 54 | 3 | 6.98E-02 | 1.00E+00 |
| protein dimerization activity (GO:0046983) | 19 | 1 | 2.87E-01 | 1.00E+00 |
| single-stranded DNA binding (GO:0003697) | 19 | 1 | 2.87E-01 | 1.00E+00 |
| ubiquitin protein ligase activity (GO:0061630) | 39 | 2 | 1.50E-01 | 1.00E+00 |
| oxidoreductase activity, acting on the CH-OH group of donors, NAD or NADP as acceptor (GO:0016616) | 20 | 1 | 2.99E-01 | 1.00E+00 |
| proton transmembrane transporter activity (GO:0015078) | 20 | 1 | 2.99E-01 | 1.00E+00 |
| cadherin binding (GO:0045296) | 20 | 1 | 2.99E-01 | 1.00E+00 |
| MAP kinase activity (GO:0004707) | 20 | 1 | 2.99E-01 | 1.00E+00 |
| oxidoreductase activity, acting on CH-OH group of donors (GO:0016614) | 21 | 1 | 3.11E-01 | 1.00E+00 |
| metal ion binding (GO:0046872) | 63 | 3 | 9.82E-02 | 1.00E+00 |
| histone binding (GO:0042393) | 21 | 1 | 3.11E-01 | 1.00E+00 |
| kinase activity (GO:0016301) | 43 | 2 | 1.74E-01 | 1.00E+00 |
| hydrolase activity, acting on carbon-nitrogen (but not peptide) bonds (GO:0016810) | 22 | 1 | 3.23E-01 | 1.00E+00 |
| phospholipid binding (GO:0005543) | 47 | 2 | 1.98E-01 | 1.00E+00 |
| ubiquitin-protein transferase activity (GO:0004842) | 71 | 3 | 1.27E-01 | 1.00E+00 |
| motor activity (GO:0003774) | 24 | 1 | 3.45E-01 | 1.00E+00 |
| cell adhesion molecule binding (GO:0050839) | 24 | 1 | 3.45E-01 | 1.00E+00 |
| ATPase activity, coupled to transmembrane movement of substances (GO:0042626) | 25 | 1 | 3.56E-01 | 1.00E+00 |
| thiol-dependent ubiquitin-specific protease activity (GO:0004843) | 25 | 1 | 3.56E-01 | 1.00E+00 |
| thiol-dependent ubiquitinyl hydrolase activity (GO:0036459) | 25 | 1 | 3.56E-01 | 1.00E+00 |
| kinase regulator activity (GO:0019207) | 25 | 1 | 3.56E-01 | 1.00E+00 |
| lipid transporter activity (GO:0005319) | 25 | 1 | 3.56E-01 | 1.00E+00 |
| ATPase activity, coupled (GO:0042623) | 79 | 3 | 1.58E-01 | 1.00E+00 |
| cation binding (GO:0043169) | 80 | 3 | 1.62E-01 | 1.00E+00 |
| S-adenosylmethionine-dependent methyltransferase activity (GO:0008757) | 27 | 1 | 3.78E-01 | 1.00E+00 |
| proximal promoter sequence-specific DNA binding (GO:0000987) | 27 | 1 | 3.78E-01 | 1.00E+00 |
| RNA polymerase II proximal promoter sequence-specific DNA binding (GO:0000978) | 27 | 1 | 3.78E-01 | 1.00E+00 |
| DNA-dependent ATPase activity (GO:0008094) | 27 | 1 | 3.78E-01 | 1.00E+00 |
| ion binding (GO:0043167) | 138 | 5 | 9.11E-02 | 1.00E+00 |
| cytokine receptor activity (GO:0004896) | 28 | 1 | 3.88E-01 | 1.00E+00 |
| guanyl-nucleotide exchange factor activity (GO:0005085) | 29 | 1 | 3.99E-01 | 1.00E+00 |
| ligase activity, forming carbon-sulfur bonds (GO:0016877) | 30 | 1 | 4.09E-01 | 1.00E+00 |
| metallopeptidase activity (GO:0008237) | 30 | 1 | 4.09E-01 | 1.00E+00 |
| ATPase activity, coupled to movement of substances (GO:0043492) | 31 | 1 | 4.19E-01 | 1.00E+00 |
| cytokine binding (GO:0019955) | 31 | 1 | 4.19E-01 | 1.00E+00 |
| ubiquitin-like protein transferase activity (GO:0019787) | 94 | 3 | 2.22E-01 | 1.00E+00 |
| transcription regulatory region DNA binding (GO:0044212) | 94 | 3 | 2.22E-01 | 1.00E+00 |
| peptide binding (GO:0042277) | 33 | 1 | 4.38E-01 | 1.00E+00 |
| phosphoprotein phosphatase activity (GO:0004721) | 33 | 1 | 4.38E-01 | 1.00E+00 |
| enzyme regulator activity (GO:0030234) | 166 | 5 | 2.11E-01 | 1.00E+00 |
| inorganic cation transmembrane transporter activity (GO:0022890) | 101 | 3 | 2.53E-01 | 1.00E+00 |
| ATPase activity (GO:0016887) | 103 | 3 | 2.62E-01 | 1.00E+00 |
| ion transmembrane transporter activity (GO:0015075) | 139 | 4 | 3.04E-01 | 1.00E+00 |
| protein kinase binding (GO:0019901) | 35 | 1 | 4.57E-01 | 1.00E+00 |
| kinase binding (GO:0019900) | 35 | 1 | 4.57E-01 | 1.00E+00 |
| methyltransferase activity (GO:0008168) | 35 | 1 | 4.57E-01 | 1.00E+00 |
| nucleotidyltransferase activity (GO:0016779) | 35 | 1 | 4.57E-01 | 1.00E+00 |
| inorganic molecular entity transmembrane transporter activity (GO:0015318) | 35 | 1 | 4.57E-01 | 1.00E+00 |
| protein serine/threonine kinase activity (GO:0004674) | 106 | 3 | 4.31E-01 | 1.00E+00 |
| cation transmembrane transporter activity (GO:0008324) | 108 | 3 | 4.35E-01 | 1.00E+00 |
| anion binding (GO:0043168) | 76 | 2 | 3.78E-01 | 1.00E+00 |
| transferase activity, transferring acyl groups other than amino-acyl groups (GO:0016747) | 38 | 1 | 4.84E-01 | 1.00E+00 |
| transferase activity, transferring one-carbon groups (GO:0016741) | 38 | 1 | 4.84E-01 | 1.00E+00 |
| transcription regulatory region sequence-specific DNA binding (GO:0000976) | 77 | 2 | 3.84E-01 | 1.00E+00 |
| endopeptidase activity (GO:0004175) | 39 | 1 | 4.93E-01 | 1.00E+00 |
| amide binding (GO:0033218) | 39 | 1 | 4.93E-01 | 1.00E+00 |
| transferase activity, transferring phosphorus-containing groups (GO:0016772) | 247 | 6 | 3.18E-01 | 1.00E+00 |
| transferase activity (GO:0016740) | 494 | 12 | 2.01E-01 | 1.00E+00 |
| hydrolase activity, acting on ester bonds (GO:0016788) | 167 | 4 | 5.35E-01 | 1.00E+00 |
| RNA polymerase II regulatory region sequence-specific DNA binding (GO:0000977) | 42 | 1 | 5.18E-01 | 1.00E+00 |
| ligase activity (GO:0016874) | 85 | 2 | 6.58E-01 | 1.00E+00 |
| microtubule binding (GO:0008017) | 44 | 1 | 5.34E-01 | 1.00E+00 |
| molecular function regulator (GO:0098772) | 221 | 5 | 4.30E-01 | 1.00E+00 |
| catalytic activity (GO:0003824) | 1325 | 29 | 1.40E-01 | 1.00E+00 |
| calcium ion binding (GO:0005509) | 46 | 1 | 5.50E-01 | 1.00E+00 |
| transcription factor binding (GO:0008134) | 47 | 1 | 5.58E-01 | 1.00E+00 |
| DNA binding (GO:0003677) | 283 | 6 | 4.85E-01 | 1.00E+00 |
| hydrolase activity (GO:0016787) | 567 | 12 | 3.93E-01 | 1.00E+00 |
| phosphotransferase activity, alcohol group as acceptor (GO:0016773) | 190 | 4 | 5.67E-01 | 1.00E+00 |
| peptidase activity, acting on L-amino acid peptides (GO:0070011) | 144 | 3 | 7.37E-01 | 1.00E+00 |
| RNA binding (GO:0003723) | 193 | 4 | 5.73E-01 | 1.00E+00 |
| transmembrane transporter activity (GO:0022857) | 195 | 4 | 5.76E-01 | 1.00E+00 |
| ubiquitin-like protein-specific protease activity (GO:0019783) | 49 | 1 | 5.72E-01 | 1.00E+00 |
| transferase activity, transferring acyl groups (GO:0016746) | 49 | 1 | 5.72E-01 | 1.00E+00 |
| DNA-binding transcription factor activity (GO:0003700) | 197 | 4 | 5.80E-01 | 1.00E+00 |
| nucleoside-triphosphatase activity (GO:0017111) | 197 | 4 | 5.80E-01 | 1.00E+00 |
| pyrophosphatase activity (GO:0016462) | 201 | 4 | 7.77E-01 | 1.00E+00 |
| hydrolase activity, acting on acid anhydrides, in phosphorus-containing anhydrides (GO:0016818) | 201 | 4 | 7.77E-01 | 1.00E+00 |
| hydrolase activity, acting on acid anhydrides (GO:0016817) | 201 | 4 | 7.77E-01 | 1.00E+00 |
| peptidase activity (GO:0008233) | 152 | 3 | 7.45E-01 | 1.00E+00 |
| tubulin binding (GO:0015631) | 52 | 1 | 5.94E-01 | 1.00E+00 |
| enzyme binding (GO:0019899) | 160 | 3 | 7.54E-01 | 1.00E+00 |
| cytoskeletal protein binding (GO:0008092) | 108 | 2 | 7.08E-01 | 1.00E+00 |
| protein kinase activity (GO:0004672) | 167 | 3 | 7.63E-01 | 1.00E+00 |
| mRNA binding (GO:0003729) | 56 | 1 | 6.21E-01 | 1.00E+00 |
| binding (GO:0005488) | 1292 | 23 | 8.05E-01 | 1.00E+00 |
| RNA polymerase II transcription factor activity, sequence-specific DNA binding (GO:0000981) | 58 | 1 | 1.00E+00 | 1.00E+00 |
| phosphatase activity (GO:0016791) | 58 | 1 | 1.00E+00 | 1.00E+00 |
| transcription regulator activity (GO:0140110) | 235 | 4 | 1.00E+00 | 1.00E+00 |
| GTPase binding (GO:0051020) | 59 | 1 | 1.00E+00 | 1.00E+00 |
| protein binding (GO:0005515) | 655 | 11 | 1.00E+00 | 1.00E+00 |
| lyase activity (GO:0016829) | 60 | 1 | 1.00E+00 | 1.00E+00 |
| transporter activity (GO:0005215) | 244 | 4 | 1.00E+00 | 1.00E+00 |
| transcription coregulator activity (GO:0003712) | 61 | 1 | 1.00E+00 | 1.00E+00 |
| Unclassified (UNCLASSIFIED) | 2599 | 41 | 5.20E-01 | 1.00E+00 |
| nucleic acid binding (GO:0003676) | 509 | 8 | 1.00E+00 | 1.00E+00 |
| heterocyclic compound binding (GO:1901363) | 526 | 8 | 8.60E-01 | 1.00E+00 |
| cysteine-type peptidase activity (GO:0008234) | 66 | 1 | 1.00E+00 | 1.00E+00 |
| catalytic activity, acting on RNA (GO:0140098) | 68 | 1 | 1.00E+00 | 1.00E+00 |
| oxidoreductase activity (GO:0016491) | 150 | 2 | 1.00E+00 | 1.00E+00 |
| signaling receptor binding (GO:0005102) | 85 | 1 | 1.00E+00 | 1.00E+00 |
| phosphoric ester hydrolase activity (GO:0042578) | 89 | 1 | 1.00E+00 | 1.00E+00 |
| transmembrane signaling receptor activity (GO:0004888) | 110 | 1 | 1.00E+00 | 1.00E+00 |
| structural molecule activity (GO:0005198) | 117 | 1 | 7.24E-01 | 1.00E+00 |
| signaling receptor activity (GO:0038023) | 156 | 1 | 5.23E-01 | 1.00E+00 |
| molecular transducer activity (GO:0060089) | 179 | 1 | 3.72E-01 | 1.00E+00 |

Table S20. Pathway analysis using PANTHER Pathways in Chiroptera. P values are calculated by Fisher’s exact test and FDR values are calculated by the Benjamini–Hochberg procedure.

| Pathway | Number of genes in reference | Number of genes in query | P-values | FDR values |
| --- | --- | --- | --- | --- |
| Purine metabolism (P02769) | 3 | 1 | 6.54E-02 | 1.00E+00 |
| Adenine and hypoxanthine salvage pathway (P02723) | 4 | 1 | 8.11E-02 | 1.00E+00 |
| Heme biosynthesis (P02746) | 5 | 1 | 9.65E-02 | 1.00E+00 |
| Plasminogen activating cascade (P00050) | 6 | 1 | 1.12E-01 | 1.00E+00 |
| FAS signaling pathway (P00020) | 7 | 1 | 1.27E-01 | 1.00E+00 |
| Apoptosis signaling pathway (P00006) | 33 | 4 | 3.20E-03 | 4.86E-01 |
| p38 MAPK pathway (P05918) | 9 | 1 | 1.56E-01 | 1.00E+00 |
| B cell activation (P00010) | 20 | 2 | 5.17E-02 | 1.00E+00 |
| Interleukin signaling pathway (P00036) | 32 | 3 | 2.03E-02 | 1.00E+00 |
| Insulin/IGF pathway-mitogen activated protein kinase kinase/MAP kinase cascade (P00032) | 11 | 1 | 1.84E-01 | 1.00E+00 |
| T cell activation (P00053) | 23 | 2 | 6.50E-02 | 1.00E+00 |
| Nicotinic acetylcholine receptor signaling pathway (P00044) | 16 | 1 | 2.50E-01 | 1.00E+00 |
| Blood coagulation (P00011) | 17 | 1 | 2.63E-01 | 1.00E+00 |
| Alzheimer disease-presenilin pathway (P00004) | 34 | 2 | 1.22E-01 | 1.00E+00 |
| Cadherin signaling pathway (P00012) | 35 | 2 | 1.27E-01 | 1.00E+00 |
| Ubiquitin proteasome pathway (P00060) | 19 | 1 | 2.87E-01 | 1.00E+00 |
| Toll receptor signaling pathway (P00054) | 19 | 1 | 2.87E-01 | 1.00E+00 |
| PDGF signaling pathway (P00047) | 45 | 2 | 1.86E-01 | 1.00E+00 |
| CCKR signaling map (P06959) | 48 | 2 | 2.04E-01 | 1.00E+00 |
| p53 pathway (P00059) | 24 | 1 | 3.45E-01 | 1.00E+00 |
| Huntington disease (P00029) | 25 | 1 | 3.56E-01 | 1.00E+00 |
| Endothelin signaling pathway (P00019) | 26 | 1 | 3.67E-01 | 1.00E+00 |
| Gonadotropin-releasing hormone receptor pathway (P06664) | 53 | 2 | 2.35E-01 | 1.00E+00 |
| FGF signaling pathway (P00021) | 28 | 1 | 3.88E-01 | 1.00E+00 |
| Wnt signaling pathway (P00057) | 61 | 2 | 2.85E-01 | 1.00E+00 |
| EGF receptor signaling pathway (P00018) | 36 | 1 | 4.66E-01 | 1.00E+00 |
| Inflammation mediated by chemokine and cytokine signaling pathway (P00031) | 57 | 1 | 1.00E+00 | 1.00E+00 |
| Integrin signalling pathway (P00034) | 58 | 1 | 1.00E+00 | 1.00E+00 |
| Angiogenesis (P00005) | 60 | 1 | 1.00E+00 | 1.00E+00 |
| Unclassified (UNCLASSIFIED) | 4457 | 73 | 3.48E-01 | 1.00E+00 |

Table S21. GO analysis in Sphenisciformes in the 2-times hypothesis using PANTHER GO-Slim Biological Process. P values are calculated by Fisher’s exact test and FDR values are calculated by the Benjamini–Hochberg procedure.

| GO term | Number of genes in reference | Number of genes in query | P-values | FDR values |
| --- | --- | --- | --- | --- |
| regulation of exit from mitosis (GO:0007096) | 1 | 1 | 4.19E-03 | 1.00E+00 |
| cell cycle arrest (GO:0007050) | 2 | 1 | 6.28E-03 | 1.00E+00 |
| regulation of cell division (GO:0051302) | 3 | 1 | 8.36E-03 | 1.00E+00 |
| regulation of cytokinesis (GO:0032465) | 3 | 1 | 8.36E-03 | 1.00E+00 |
| nuclear transport (GO:0051169) | 6 | 1 | 1.46E-02 | 1.00E+00 |
| response to calcium ion (GO:0051592) | 7 | 1 | 1.67E-02 | 1.00E+00 |
| response to metal ion (GO:0010038) | 7 | 1 | 1.67E-02 | 1.00E+00 |
| membrane fission (GO:0090148) | 8 | 1 | 1.87E-02 | 1.00E+00 |
| regulation of mitotic nuclear division (GO:0007088) | 9 | 1 | 2.08E-02 | 1.00E+00 |
| response to inorganic substance (GO:0010035) | 9 | 1 | 2.08E-02 | 1.00E+00 |
| negative regulation of mitotic cell cycle (GO:0045930) | 12 | 1 | 2.69E-02 | 1.00E+00 |
| positive regulation of cell cycle (GO:0045787) | 14 | 1 | 3.10E-02 | 1.00E+00 |
| cellular amino acid catabolic process (GO:0009063) | 16 | 1 | 3.51E-02 | 1.00E+00 |
| negative regulation of cell cycle (GO:0045786) | 17 | 1 | 3.71E-02 | 1.00E+00 |
| cell cycle process (GO:0022402) | 17 | 1 | 3.71E-02 | 1.00E+00 |
| mitotic cell cycle phase transition (GO:0044772) | 17 | 1 | 3.71E-02 | 1.00E+00 |
| regulation of cell cycle process (GO:0010564) | 22 | 1 | 4.72E-02 | 1.00E+00 |
| cytokinesis (GO:0000910) | 22 | 1 | 4.72E-02 | 1.00E+00 |
| cell division (GO:0051301) | 23 | 1 | 4.92E-02 | 1.00E+00 |
| tRNA metabolic process (GO:0006399) | 25 | 1 | 5.32E-02 | 1.00E+00 |
| cellular response to chemical stimulus (GO:0070887) | 28 | 1 | 5.92E-02 | 1.00E+00 |
| negative regulation of cellular process (GO:0048523) | 31 | 1 | 6.52E-02 | 1.00E+00 |
| regulation of cell cycle (GO:0051726) | 33 | 1 | 6.91E-02 | 1.00E+00 |
| carboxylic acid catabolic process (GO:0046395) | 33 | 1 | 6.91E-02 | 1.00E+00 |
| organic acid catabolic process (GO:0016054) | 33 | 1 | 6.91E-02 | 1.00E+00 |
| intracellular transport (GO:0046907) | 33 | 1 | 6.91E-02 | 1.00E+00 |
| mRNA splicing, via spliceosome (GO:0000398) | 34 | 1 | 7.11E-02 | 1.00E+00 |
| RNA splicing, via transesterification reactions with bulged adenosine as nucleophile (GO:0000377) | 34 | 1 | 7.11E-02 | 1.00E+00 |
| RNA splicing, via transesterification reactions (GO:0000375) | 35 | 1 | 7.30E-02 | 1.00E+00 |
| microtubule cytoskeleton organization (GO:0000226) | 36 | 1 | 7.50E-02 | 1.00E+00 |
| RNA splicing (GO:0008380) | 36 | 1 | 7.50E-02 | 1.00E+00 |
| positive regulation of cellular process (GO:0048522) | 37 | 1 | 7.70E-02 | 1.00E+00 |
| transmembrane receptor protein tyrosine kinase signaling pathway (GO:0007169) | 42 | 1 | 8.67E-02 | 1.00E+00 |
| enzyme linked receptor protein signaling pathway (GO:0007167) | 51 | 1 | 1.04E-01 | 1.00E+00 |
| RNA processing (GO:0006396) | 52 | 1 | 1.06E-01 | 1.00E+00 |
| apoptotic process (GO:0006915) | 53 | 1 | 1.08E-01 | 1.00E+00 |
| ncRNA metabolic process (GO:0034660) | 53 | 1 | 1.08E-01 | 1.00E+00 |
| programmed cell death (GO:0012501) | 54 | 1 | 1.10E-01 | 1.00E+00 |
| microtubule-based process (GO:0007017) | 56 | 1 | 1.13E-01 | 1.00E+00 |
| cell death (GO:0008219) | 57 | 1 | 1.15E-01 | 1.00E+00 |
| organic substance catabolic process (GO:1901575) | 57 | 1 | 1.15E-01 | 1.00E+00 |
| membrane organization (GO:0061024) | 58 | 1 | 1.17E-01 | 1.00E+00 |
| positive regulation of biological process (GO:0048518) | 59 | 1 | 1.19E-01 | 1.00E+00 |
| mitotic nuclear division (GO:0140014) | 66 | 1 | 1.32E-01 | 1.00E+00 |
| mitotic cell cycle (GO:0000278) | 68 | 1 | 1.36E-01 | 1.00E+00 |
| mitotic cell cycle process (GO:1903047) | 68 | 1 | 1.36E-01 | 1.00E+00 |
| protein metabolic process (GO:0019538) | 68 | 1 | 1.36E-01 | 1.00E+00 |
| catabolic process (GO:0009056) | 72 | 1 | 1.43E-01 | 1.00E+00 |
| nervous system process (GO:0050877) | 88 | 1 | 1.72E-01 | 1.00E+00 |
| RNA metabolic process (GO:0016070) | 92 | 1 | 1.79E-01 | 1.00E+00 |
| response to chemical (GO:0042221) | 100 | 1 | 1.93E-01 | 1.00E+00 |
| signal transduction (GO:0007165) | 328 | 3 | 2.69E-02 | 1.00E+00 |
| system process (GO:0003008) | 110 | 1 | 2.10E-01 | 1.00E+00 |
| primary metabolic process (GO:0044238) | 118 | 1 | 2.24E-01 | 1.00E+00 |
| nucleic acid metabolic process (GO:0090304) | 131 | 1 | 2.45E-01 | 1.00E+00 |
| cell surface receptor signaling pathway (GO:0007166) | 131 | 1 | 2.45E-01 | 1.00E+00 |
| cellular response to stimulus (GO:0051716) | 435 | 3 | 5.55E-02 | 1.00E+00 |
| cell cycle (GO:0007049) | 156 | 1 | 2.85E-01 | 1.00E+00 |
| intracellular protein transport (GO:0006886) | 169 | 1 | 3.05E-01 | 1.00E+00 |
| response to stimulus (GO:0050896) | 180 | 1 | 3.22E-01 | 1.00E+00 |
| cellular protein localization (GO:0034613) | 207 | 1 | 3.61E-01 | 1.00E+00 |
| cellular macromolecule localization (GO:0070727) | 208 | 1 | 3.62E-01 | 1.00E+00 |
| regulation of cellular process (GO:0050794) | 211 | 1 | 3.66E-01 | 1.00E+00 |
| transcription by RNA polymerase II (GO:0006366) | 227 | 1 | 3.88E-01 | 1.00E+00 |
| cellular localization (GO:0051641) | 235 | 1 | 3.99E-01 | 1.00E+00 |
| multicellular organismal process (GO:0032501) | 236 | 1 | 4.00E-01 | 1.00E+00 |
| establishment of localization (GO:0051234) | 262 | 1 | 4.34E-01 | 1.00E+00 |
| transport (GO:0006810) | 262 | 1 | 4.34E-01 | 1.00E+00 |
| transcription, DNA-templated (GO:0006351) | 277 | 1 | 4.53E-01 | 1.00E+00 |
| macromolecule metabolic process (GO:0043170) | 615 | 2 | 6.26E-01 | 1.00E+00 |
| cellular process (GO:0009987) | 1339 | 4 | 4.74E-01 | 1.00E+00 |
| metabolic process (GO:0008152) | 1035 | 3 | 4.59E-01 | 1.00E+00 |
| cellular component organization (GO:0016043) | 363 | 1 | 5.50E-01 | 1.00E+00 |
| organic substance metabolic process (GO:0071704) | 866 | 2 | 1.00E+00 | 1.00E+00 |
| gene expression (GO:0010467) | 441 | 1 | 1.00E+00 | 1.00E+00 |
| localization (GO:0051179) | 457 | 1 | 1.00E+00 | 1.00E+00 |
| Unclassified (UNCLASSIFIED) | 2019 | 4 | 1.00E+00 | 1.00E+00 |
| regulation of biological process (GO:0050789) | 583 | 1 | 1.00E+00 | 1.00E+00 |
| biological regulation (GO:0065007) | 668 | 1 | 1.00E+00 | 1.00E+00 |

Table S22. GO analysis in Sphenisciformes in the 2-times hypothesis using PANTHER GO-Slim Cellular Component. P values are calculated by Fisher’s exact test and FDR values are calculated by the Benjamini–Hochberg procedure.

| GO term | Number of genes in reference | Number of genes in query | P-values | FDR values |
| --- | --- | --- | --- | --- |
| mitotic spindle (GO:0072686) | 3 | 1 | 8.36E-03 | 1.00E+00 |
| spindle pole (GO:0000922) | 5 | 1 | 1.25E-02 | 1.00E+00 |
| spindle (GO:0005819) | 10 | 1 | 2.28E-02 | 1.00E+00 |
| centrosome (GO:0005813) | 29 | 1 | 6.12E-02 | 1.00E+00 |
| microtubule organizing center part (GO:0044450) | 35 | 1 | 7.30E-02 | 1.00E+00 |
| centriole (GO:0005814) | 35 | 1 | 7.30E-02 | 1.00E+00 |
| nucleolus (GO:0005730) | 42 | 1 | 8.67E-02 | 1.00E+00 |
| microtubule organizing center (GO:0005815) | 44 | 1 | 9.05E-02 | 1.00E+00 |
| microtubule cytoskeleton (GO:0015630) | 93 | 1 | 1.81E-01 | 1.00E+00 |
| nuclear lumen (GO:0031981) | 144 | 1 | 2.66E-01 | 1.00E+00 |
| cytoskeleton (GO:0005856) | 154 | 1 | 2.82E-01 | 1.00E+00 |
| nuclear part (GO:0044428) | 227 | 1 | 3.88E-01 | 1.00E+00 |
| nucleus (GO:0005634) | 513 | 2 | 2.95E-01 | 1.00E+00 |
| intracellular membrane-bounded organelle (GO:0043231) | 527 | 2 | 3.06E-01 | 1.00E+00 |
| intracellular organelle (GO:0043229) | 528 | 2 | 3.07E-01 | 1.00E+00 |
| intracellular non-membrane-bounded organelle (GO:0043232) | 284 | 1 | 4.61E-01 | 1.00E+00 |
| non-membrane-bounded organelle (GO:0043228) | 284 | 1 | 4.61E-01 | 1.00E+00 |
| Unclassified (UNCLASSIFIED) | 2289 | 6 | 5.17E-01 | 1.00E+00 |
| organelle (GO:0043226) | 903 | 2 | 1.00E+00 | 1.00E+00 |
| plasma membrane (GO:0005886) | 483 | 1 | 1.00E+00 | 1.00E+00 |
| cell periphery (GO:0071944) | 493 | 1 | 1.00E+00 | 1.00E+00 |
| cell part (GO:0044464) | 1219 | 1 | 4.60E-01 | 1.00E+00 |
| cell (GO:0005623) | 1225 | 1 | 4.60E-01 | 1.00E+00 |

Table S23. GO analysis in Sphenisciformes in the 2-times hypothesis using PANTHER GO-Slim Molecular Function. P values are calculated by Fisher’s exact test and FDR values are calculated by the Benjamini–Hochberg procedure.

| GO term | Number of genes in reference | Number of genes in query | P-values | FDR values |
| --- | --- | --- | --- | --- |
| receptor tyrosine kinase binding (GO:0030971) | 2 | 1 | 6.28E-03 | 1.00E+00 |
| protein serine/threonine phosphatase activity (GO:0004722) | 12 | 1 | 2.69E-02 | 1.00E+00 |
| calcium-dependent phospholipid binding (GO:0005544) | 15 | 1 | 3.31E-02 | 1.00E+00 |
| phosphoprotein phosphatase activity (GO:0004721) | 31 | 1 | 6.52E-02 | 1.00E+00 |
| mRNA binding (GO:0003729) | 41 | 1 | 8.47E-02 | 1.00E+00 |
| phospholipid binding (GO:0005543) | 42 | 1 | 8.67E-02 | 1.00E+00 |
| phosphatase activity (GO:0016791) | 56 | 1 | 1.13E-01 | 1.00E+00 |
| anion binding (GO:0043168) | 67 | 1 | 1.34E-01 | 1.00E+00 |
| RNA binding (GO:0003723) | 135 | 2 | 3.14E-02 | 1.00E+00 |
| signaling receptor binding (GO:0005102) | 68 | 1 | 1.36E-01 | 1.00E+00 |
| phosphoric ester hydrolase activity (GO:0042578) | 82 | 1 | 1.61E-01 | 1.00E+00 |
| ion binding (GO:0043167) | 121 | 1 | 2.29E-01 | 1.00E+00 |
| protein kinase activity (GO:0004672) | 135 | 1 | 2.52E-01 | 1.00E+00 |
| hydrolase activity, acting on ester bonds (GO:0016788) | 146 | 1 | 2.69E-01 | 1.00E+00 |
| phosphotransferase activity, alcohol group as acceptor (GO:0016773) | 156 | 1 | 2.85E-01 | 1.00E+00 |
| nucleic acid binding (GO:0003676) | 406 | 2 | 2.08E-01 | 1.00E+00 |
| transferase activity, transferring phosphorus-containing groups (GO:0016772) | 205 | 1 | 3.58E-01 | 1.00E+00 |
| heterocyclic compound binding (GO:1901363) | 417 | 2 | 2.17E-01 | 1.00E+00 |
| protein binding (GO:0005515) | 563 | 2 | 3.36E-01 | 1.00E+00 |
| binding (GO:0005488) | 1109 | 3 | 7.03E-01 | 1.00E+00 |
| catalytic activity (GO:0003824) | 1153 | 3 | 7.09E-01 | 1.00E+00 |
| transferase activity (GO:0016740) | 451 | 1 | 1.00E+00 | 1.00E+00 |
| hydrolase activity (GO:0016787) | 490 | 1 | 1.00E+00 | 1.00E+00 |
| Unclassified (UNCLASSIFIED) | 2114 | 4 | 1.00E+00 | 1.00E+00 |

Table S24. Pathway analysis in Sphenisciformes in the 2-times hypothesis using PANTHER Pathways. P values are calculated by Fisher’s exact test and FDR values are calculated by the Benjamini–Hochberg procedure.

| Pathway | Number of genes in reference | Number of genes in query | P-values | FDR values |
| --- | --- | --- | --- | --- |
| Unclassified (UNCLASSIFIED) | 3681 | 9 | 6.23E-01 | 1.00E+00 |

Table S25. GO analysis in Struthioniformes in the 2-times hypothesis using PANTHER GO-Slim Biological Process. P values are calculated by Fisher’s exact test and FDR values are calculated by the Benjamini–Hochberg procedure.

| GO term | Number of genes in reference | Number of genes in query | P-values | FDR values |
| --- | --- | --- | --- | --- |
| protein demethylation (GO:0006482) | 1 | 1 | 2.20E-02 | 1.00E+00 |
| interleukin-6 production (GO:0032635) | 1 | 1 | 2.20E-02 | 1.00E+00 |
| regulation of cytokine biosynthetic process (GO:0042035) | 1 | 1 | 2.20E-02 | 1.00E+00 |
| protein dealkylation (GO:0008214) | 1 | 1 | 2.20E-02 | 1.00E+00 |
| positive regulation of protein metabolic process (GO:0051247) | 1 | 1 | 2.20E-02 | 1.00E+00 |
| positive regulation of Wnt signaling pathway (GO:0030177) | 2 | 1 | 3.29E-02 | 1.00E+00 |
| cellular nitrogen compound catabolic process (GO:0044270) | 2 | 1 | 3.29E-02 | 1.00E+00 |
| cytokine production (GO:0001816) | 2 | 1 | 3.29E-02 | 1.00E+00 |
| cation homeostasis (GO:0055080) | 2 | 1 | 3.29E-02 | 1.00E+00 |
| defense response to virus (GO:0051607) | 2 | 1 | 3.29E-02 | 1.00E+00 |
| positive regulation of canonical Wnt signaling pathway (GO:0090263) | 2 | 1 | 3.29E-02 | 1.00E+00 |
| cellular monovalent inorganic cation homeostasis (GO:0030004) | 2 | 1 | 3.29E-02 | 1.00E+00 |
| cellular cation homeostasis (GO:0030003) | 2 | 1 | 3.29E-02 | 1.00E+00 |
| centrosome duplication (GO:0051298) | 5 | 2 | 2.44E-03 | 1.00E+00 |
| glycerolipid catabolic process (GO:0046503) | 3 | 1 | 4.36E-02 | 1.00E+00 |
| T cell activation (GO:0042110) | 3 | 1 | 4.36E-02 | 1.00E+00 |
| regulation of translational initiation (GO:0006446) | 3 | 1 | 4.36E-02 | 1.00E+00 |
| protein-containing complex localization (GO:0031503) | 3 | 1 | 4.36E-02 | 1.00E+00 |
| positive regulation of NF-kappaB transcription factor activity (GO:0051092) | 6 | 2 | 3.23E-03 | 1.00E+00 |
| positive regulation of I-kappaB kinase/NF-kappaB signaling (GO:0043123) | 3 | 1 | 4.36E-02 | 1.00E+00 |
| regulation of I-kappaB kinase/NF-kappaB signaling (GO:0043122) | 3 | 1 | 4.36E-02 | 1.00E+00 |
| heterocycle catabolic process (GO:0046700) | 3 | 1 | 4.36E-02 | 1.00E+00 |
| immune effector process (GO:0002252) | 3 | 1 | 4.36E-02 | 1.00E+00 |
| positive regulation of ERK1 and ERK2 cascade (GO:0070374) | 3 | 1 | 4.36E-02 | 1.00E+00 |
| protein localization to synapse (GO:0035418) | 3 | 1 | 4.36E-02 | 1.00E+00 |
| pyrimidine nucleobase metabolic process (GO:0006206) | 3 | 1 | 4.36E-02 | 1.00E+00 |
| positive regulation of cellular biosynthetic process (GO:0031328) | 3 | 1 | 4.36E-02 | 1.00E+00 |
| establishment or maintenance of epithelial cell apical/basal polarity (GO:0045197) | 3 | 1 | 4.36E-02 | 1.00E+00 |
| receptor localization to synapse (GO:0097120) | 3 | 1 | 4.36E-02 | 1.00E+00 |
| nucleobase metabolic process (GO:0009112) | 4 | 1 | 5.42E-02 | 1.00E+00 |
| synapsis (GO:0007129) | 4 | 1 | 5.42E-02 | 1.00E+00 |
| transcription by RNA polymerase III (GO:0006383) | 4 | 1 | 5.42E-02 | 1.00E+00 |
| activation of immune response (GO:0002253) | 4 | 1 | 5.42E-02 | 1.00E+00 |
| toll-like receptor signaling pathway (GO:0002224) | 4 | 1 | 5.42E-02 | 1.00E+00 |
| activation of innate immune response (GO:0002218) | 4 | 1 | 5.42E-02 | 1.00E+00 |
| lymphocyte activation (GO:0046649) | 4 | 1 | 5.42E-02 | 1.00E+00 |
| protein K11-linked ubiquitination (GO:0070979) | 4 | 1 | 5.42E-02 | 1.00E+00 |
| cytokine-mediated signaling pathway (GO:0019221) | 9 | 2 | 6.20E-03 | 1.00E+00 |
| positive regulation of DNA-binding transcription factor activity (GO:0051091) | 9 | 2 | 6.20E-03 | 1.00E+00 |
| centrosome cycle (GO:0007098) | 9 | 2 | 6.20E-03 | 1.00E+00 |
| I-kappaB kinase/NF-kappaB signaling (GO:0007249) | 10 | 2 | 7.39E-03 | 1.00E+00 |
| leukocyte activation (GO:0045321) | 5 | 1 | 6.47E-02 | 1.00E+00 |
| homologous chromosome segregation (GO:0045143) | 5 | 1 | 6.47E-02 | 1.00E+00 |
| cellular response to cytokine stimulus (GO:0071345) | 10 | 2 | 7.39E-03 | 1.00E+00 |
| nuclear transport (GO:0051169) | 6 | 1 | 7.51E-02 | 1.00E+00 |
| innate immune response (GO:0045087) | 6 | 1 | 7.51E-02 | 1.00E+00 |
| organophosphate catabolic process (GO:0046434) | 6 | 1 | 7.51E-02 | 1.00E+00 |
| microtubule polymerization (GO:0046785) | 6 | 1 | 7.51E-02 | 1.00E+00 |
| wound healing (GO:0042060) | 6 | 1 | 7.51E-02 | 1.00E+00 |
| positive regulation of macromolecule biosynthetic process (GO:0010557) | 6 | 1 | 7.51E-02 | 1.00E+00 |
| mitotic sister chromatid separation (GO:0051306) | 6 | 1 | 7.51E-02 | 1.00E+00 |
| response to wounding (GO:0009611) | 6 | 1 | 7.51E-02 | 1.00E+00 |
| organic cyclic compound catabolic process (GO:1901361) | 6 | 1 | 7.51E-02 | 1.00E+00 |
| metaphase/anaphase transition of mitotic cell cycle (GO:0007091) | 6 | 1 | 7.51E-02 | 1.00E+00 |
| spindle organization (GO:0007051) | 6 | 1 | 7.51E-02 | 1.00E+00 |
| microtubule nucleation (GO:0007020) | 6 | 1 | 7.51E-02 | 1.00E+00 |
| histone modification (GO:0016570) | 7 | 1 | 8.53E-02 | 1.00E+00 |
| positive regulation of MAPK cascade (GO:0043410) | 7 | 1 | 8.53E-02 | 1.00E+00 |
| protein polymerization (GO:0051258) | 7 | 1 | 8.53E-02 | 1.00E+00 |
| cellular metal ion homeostasis (GO:0006875) | 7 | 1 | 8.53E-02 | 1.00E+00 |
| response to cytokine (GO:0034097) | 15 | 2 | 1.47E-02 | 1.00E+00 |
| regulation of cellular amide metabolic process (GO:0034248) | 16 | 2 | 1.64E-02 | 1.00E+00 |
| regulation of translation (GO:0006417) | 16 | 2 | 1.64E-02 | 1.00E+00 |
| heterocycle metabolic process (GO:0046483) | 8 | 1 | 9.55E-02 | 1.00E+00 |
| SCF-dependent proteasomal ubiquitin-dependent protein catabolic process (GO:0031146) | 8 | 1 | 9.55E-02 | 1.00E+00 |
| positive regulation of biosynthetic process (GO:0009891) | 8 | 1 | 9.55E-02 | 1.00E+00 |
| epithelial cell differentiation (GO:0030855) | 8 | 1 | 9.55E-02 | 1.00E+00 |
| establishment or maintenance of cell polarity (GO:0007163) | 17 | 2 | 1.82E-02 | 1.00E+00 |
| cell cycle process (GO:0022402) | 17 | 2 | 1.82E-02 | 1.00E+00 |
| regulation of mitotic nuclear division (GO:0007088) | 9 | 1 | 1.06E-01 | 1.00E+00 |
| positive regulation of metabolic process (GO:0009893) | 9 | 1 | 1.06E-01 | 1.00E+00 |
| proton transmembrane transport (GO:1902600) | 9 | 1 | 1.06E-01 | 1.00E+00 |
| epithelium development (GO:0060429) | 11 | 1 | 1.25E-01 | 1.00E+00 |
| canonical Wnt signaling pathway (GO:0060070) | 11 | 1 | 1.25E-01 | 1.00E+00 |
| protein localization to membrane (GO:0072657) | 11 | 1 | 1.25E-01 | 1.00E+00 |
| regulation of MAPK cascade (GO:0043408) | 11 | 1 | 1.25E-01 | 1.00E+00 |
| positive regulation of macromolecule metabolic process (GO:0010604) | 12 | 1 | 1.35E-01 | 1.00E+00 |
| cellular lipid catabolic process (GO:0044242) | 12 | 1 | 1.35E-01 | 1.00E+00 |
| meiosis I cell cycle phase (GO:0098764) | 13 | 1 | 1.45E-01 | 1.00E+00 |
| meiotic cell cycle phase (GO:0098762) | 13 | 1 | 1.45E-01 | 1.00E+00 |
| meiotic telophase I (GO:0007134) | 13 | 1 | 1.45E-01 | 1.00E+00 |
| biological phase (GO:0044848) | 13 | 1 | 1.45E-01 | 1.00E+00 |
| meiosis I (GO:0007127) | 13 | 1 | 1.45E-01 | 1.00E+00 |
| metal ion homeostasis (GO:0055065) | 13 | 1 | 1.45E-01 | 1.00E+00 |
| cell cycle phase (GO:0022403) | 13 | 1 | 1.45E-01 | 1.00E+00 |
| ribose phosphate metabolic process (GO:0019693) | 13 | 1 | 1.45E-01 | 1.00E+00 |
| positive regulation of multicellular organismal process (GO:0051240) | 13 | 1 | 1.45E-01 | 1.00E+00 |
| Golgi organization (GO:0007030) | 13 | 1 | 1.45E-01 | 1.00E+00 |
| positive regulation of molecular function (GO:0044093) | 26 | 2 | 3.78E-02 | 1.00E+00 |
| positive regulation of cell cycle (GO:0045787) | 14 | 1 | 1.54E-01 | 1.00E+00 |
| negative regulation of intracellular signal transduction (GO:1902532) | 14 | 1 | 1.54E-01 | 1.00E+00 |
| regulation of cyclin-dependent protein serine/threonine kinase activity (GO:0000079) | 14 | 1 | 1.54E-01 | 1.00E+00 |
| import into cell (GO:0098657) | 14 | 1 | 1.54E-01 | 1.00E+00 |
| meiosis I cell cycle process (GO:0061982) | 14 | 1 | 1.54E-01 | 1.00E+00 |
| immune system process (GO:0002376) | 43 | 3 | 1.39E-02 | 1.00E+00 |
| ion transmembrane transport (GO:0034220) | 15 | 1 | 1.64E-01 | 1.00E+00 |
| protein N-linked glycosylation (GO:0006487) | 15 | 1 | 1.64E-01 | 1.00E+00 |
| defense response (GO:0006952) | 15 | 1 | 1.64E-01 | 1.00E+00 |
| inorganic cation transmembrane transport (GO:0098662) | 15 | 1 | 1.64E-01 | 1.00E+00 |
| inorganic ion transmembrane transport (GO:0098660) | 15 | 1 | 1.64E-01 | 1.00E+00 |
| movement of cell or subcellular component (GO:0006928) | 15 | 1 | 1.64E-01 | 1.00E+00 |
| stress-activated protein kinase signaling cascade (GO:0031098) | 15 | 1 | 1.64E-01 | 1.00E+00 |
| peptidyl-amino acid modification (GO:0018193) | 48 | 3 | 1.83E-02 | 1.00E+00 |
| mitotic sister chromatid segregation (GO:0000070) | 16 | 1 | 1.73E-01 | 1.00E+00 |
| Wnt signaling pathway (GO:0016055) | 16 | 1 | 1.73E-01 | 1.00E+00 |
| positive regulation of protein kinase activity (GO:0045860) | 16 | 1 | 1.73E-01 | 1.00E+00 |
| activation of protein kinase activity (GO:0032147) | 16 | 1 | 1.73E-01 | 1.00E+00 |
| cellular catabolic process (GO:0044248) | 17 | 1 | 1.82E-01 | 1.00E+00 |
| positive regulation of cell proliferation (GO:0008284) | 17 | 1 | 1.82E-01 | 1.00E+00 |
| cell-cell adhesion (GO:0098609) | 17 | 1 | 1.82E-01 | 1.00E+00 |
| mitotic cell cycle phase transition (GO:0044772) | 17 | 1 | 1.82E-01 | 1.00E+00 |
| cell-cell signaling by wnt (GO:0198738) | 17 | 1 | 1.82E-01 | 1.00E+00 |
| organonitrogen compound catabolic process (GO:1901565) | 18 | 1 | 1.91E-01 | 1.00E+00 |
| nucleobase-containing small molecule metabolic process (GO:0055086) | 37 | 2 | 6.87E-02 | 1.00E+00 |
| glycerophospholipid metabolic process (GO:0006650) | 19 | 1 | 2.00E-01 | 1.00E+00 |
| proteasome-mediated ubiquitin-dependent protein catabolic process (GO:0043161) | 40 | 2 | 7.82E-02 | 1.00E+00 |
| positive regulation of signal transduction (GO:0009967) | 20 | 1 | 2.09E-01 | 1.00E+00 |
| protein localization (GO:0008104) | 20 | 1 | 2.09E-01 | 1.00E+00 |
| protein polyubiquitination (GO:0000209) | 21 | 1 | 2.18E-01 | 1.00E+00 |
| cell proliferation (GO:0008283) | 21 | 1 | 2.18E-01 | 1.00E+00 |
| positive regulation of response to stimulus (GO:0048584) | 21 | 1 | 2.18E-01 | 1.00E+00 |
| peptidyl-lysine modification (GO:0018205) | 21 | 1 | 2.18E-01 | 1.00E+00 |
| regulation of cell cycle process (GO:0010564) | 22 | 1 | 2.27E-01 | 1.00E+00 |
| mitotic nuclear division (GO:0140014) | 66 | 3 | 4.02E-02 | 1.00E+00 |
| regulation of multicellular organismal process (GO:0051239) | 22 | 1 | 2.27E-01 | 1.00E+00 |
| proteasomal protein catabolic process (GO:0010498) | 45 | 2 | 9.49E-02 | 1.00E+00 |
| mitotic cell cycle (GO:0000278) | 68 | 3 | 4.31E-02 | 1.00E+00 |
| mitotic cell cycle process (GO:1903047) | 68 | 3 | 4.31E-02 | 1.00E+00 |
| protein metabolic process (GO:0019538) | 68 | 3 | 4.31E-02 | 1.00E+00 |
| regulation of cell proliferation (GO:0042127) | 23 | 1 | 2.35E-01 | 1.00E+00 |
| signal transduction by protein phosphorylation (GO:0023014) | 47 | 2 | 1.02E-01 | 1.00E+00 |
| endomembrane system organization (GO:0010256) | 24 | 1 | 2.44E-01 | 1.00E+00 |
| drug metabolic process (GO:0017144) | 24 | 1 | 2.44E-01 | 1.00E+00 |
| regulation of intracellular signal transduction (GO:1902531) | 51 | 2 | 1.16E-01 | 1.00E+00 |
| positive regulation of protein phosphorylation (GO:0001934) | 26 | 1 | 2.60E-01 | 1.00E+00 |
| positive regulation of phosphorylation (GO:0042327) | 26 | 1 | 2.60E-01 | 1.00E+00 |
| lipid metabolic process (GO:0006629) | 26 | 1 | 2.60E-01 | 1.00E+00 |
| protein modification by small protein removal (GO:0070646) | 26 | 1 | 2.60E-01 | 1.00E+00 |
| cell cycle (GO:0007049) | 156 | 6 | 8.31E-03 | 1.00E+00 |
| tissue development (GO:0009888) | 26 | 1 | 2.60E-01 | 1.00E+00 |
| response to organic substance (GO:0010033) | 52 | 2 | 1.20E-01 | 1.00E+00 |
| response to stress (GO:0006950) | 53 | 2 | 1.23E-01 | 1.00E+00 |
| apoptotic process (GO:0006915) | 53 | 2 | 1.23E-01 | 1.00E+00 |
| programmed cell death (GO:0012501) | 54 | 2 | 1.27E-01 | 1.00E+00 |
| glycerolipid metabolic process (GO:0046486) | 27 | 1 | 2.69E-01 | 1.00E+00 |
| small molecule metabolic process (GO:0044281) | 55 | 2 | 1.31E-01 | 1.00E+00 |
| meiotic cell cycle process (GO:1903046) | 28 | 1 | 2.77E-01 | 1.00E+00 |
| meiotic cell cycle (GO:0051321) | 28 | 1 | 2.77E-01 | 1.00E+00 |
| cell death (GO:0008219) | 57 | 2 | 1.38E-01 | 1.00E+00 |
| reproductive process (GO:0022414) | 29 | 1 | 2.85E-01 | 1.00E+00 |
| positive regulation of biological process (GO:0048518) | 59 | 2 | 1.46E-01 | 1.00E+00 |
| cellular component morphogenesis (GO:0032989) | 30 | 1 | 2.93E-01 | 1.00E+00 |
| organelle assembly (GO:0070925) | 30 | 1 | 2.93E-01 | 1.00E+00 |
| regulation of molecular function (GO:0065009) | 60 | 2 | 1.50E-01 | 1.00E+00 |
| cell-cell signaling (GO:0007267) | 92 | 3 | 8.71E-02 | 1.00E+00 |
| cell communication (GO:0007154) | 92 | 3 | 8.71E-02 | 1.00E+00 |
| cellular protein modification process (GO:0006464) | 216 | 7 | 1.05E-02 | 1.00E+00 |
| protein glycosylation (GO:0006486) | 31 | 1 | 3.01E-01 | 1.00E+00 |
| negative regulation of signal transduction (GO:0009968) | 31 | 1 | 3.01E-01 | 1.00E+00 |
| cellular protein-containing complex assembly (GO:0034622) | 31 | 1 | 3.01E-01 | 1.00E+00 |
| nucleoside phosphate metabolic process (GO:0006753) | 32 | 1 | 3.09E-01 | 1.00E+00 |
| regulation of macromolecule metabolic process (GO:0060255) | 32 | 1 | 3.09E-01 | 1.00E+00 |
| organonitrogen compound biosynthetic process (GO:1901566) | 32 | 1 | 3.09E-01 | 1.00E+00 |
| glycoprotein biosynthetic process (GO:0009101) | 33 | 1 | 3.16E-01 | 1.00E+00 |
| regulation of cell cycle (GO:0051726) | 33 | 1 | 3.16E-01 | 1.00E+00 |
| cellular protein metabolic process (GO:0044267) | 231 | 7 | 1.48E-02 | 1.00E+00 |
| cellular macromolecule metabolic process (GO:0044260) | 231 | 7 | 1.48E-02 | 1.00E+00 |
| negative regulation of response to stimulus (GO:0048585) | 33 | 1 | 3.16E-01 | 1.00E+00 |
| intracellular transport (GO:0046907) | 33 | 1 | 3.16E-01 | 1.00E+00 |
| ion transport (GO:0006811) | 33 | 1 | 3.16E-01 | 1.00E+00 |
| regulation of cell communication (GO:0010646) | 68 | 2 | 1.81E-01 | 1.00E+00 |
| regulation of signal transduction (GO:0009966) | 68 | 2 | 1.81E-01 | 1.00E+00 |
| mRNA splicing, via spliceosome (GO:0000398) | 34 | 1 | 3.24E-01 | 1.00E+00 |
| RNA splicing, via transesterification reactions with bulged adenosine as nucleophile (GO:0000377) | 34 | 1 | 3.24E-01 | 1.00E+00 |
| formation of translation initiation ternary complex (GO:0001677) | 69 | 2 | 1.85E-01 | 1.00E+00 |
| translational termination (GO:0006415) | 69 | 2 | 1.85E-01 | 1.00E+00 |
| translational elongation (GO:0006414) | 69 | 2 | 1.85E-01 | 1.00E+00 |
| RNA splicing, via transesterification reactions (GO:0000375) | 35 | 1 | 3.31E-01 | 1.00E+00 |
| intracellular signal transduction (GO:0035556) | 142 | 4 | 7.68E-02 | 1.00E+00 |
| RNA splicing (GO:0008380) | 36 | 1 | 3.39E-01 | 1.00E+00 |
| proteolysis involved in cellular protein catabolic process (GO:0051603) | 72 | 2 | 1.97E-01 | 1.00E+00 |
| cellular protein catabolic process (GO:0044257) | 73 | 2 | 2.01E-01 | 1.00E+00 |
| carbohydrate derivative metabolic process (GO:1901135) | 74 | 2 | 2.05E-01 | 1.00E+00 |
| positive regulation of cellular process (GO:0048522) | 37 | 1 | 3.46E-01 | 1.00E+00 |
| protein phosphorylation (GO:0006468) | 75 | 2 | 2.10E-01 | 1.00E+00 |
| MAPK cascade (GO:0000165) | 39 | 1 | 3.61E-01 | 1.00E+00 |
| primary metabolic process (GO:0044238) | 118 | 3 | 1.49E-01 | 1.00E+00 |
| regulation of cellular metabolic process (GO:0031323) | 119 | 3 | 1.52E-01 | 1.00E+00 |
| macromolecule localization (GO:0033036) | 40 | 1 | 3.68E-01 | 1.00E+00 |
| regulation of phosphorylation (GO:0042325) | 41 | 1 | 3.75E-01 | 1.00E+00 |
| protein catabolic process (GO:0030163) | 83 | 2 | 2.42E-01 | 1.00E+00 |
| translation (GO:0006412) | 83 | 2 | 2.42E-01 | 1.00E+00 |
| carbohydrate derivative biosynthetic process (GO:1901137) | 42 | 1 | 3.82E-01 | 1.00E+00 |
| transmembrane receptor protein tyrosine kinase signaling pathway (GO:0007169) | 42 | 1 | 3.82E-01 | 1.00E+00 |
| organophosphate metabolic process (GO:0019637) | 42 | 1 | 3.82E-01 | 1.00E+00 |
| macromolecule catabolic process (GO:0009057) | 85 | 2 | 2.51E-01 | 1.00E+00 |
| response to stimulus (GO:0050896) | 180 | 4 | 1.45E-01 | 1.00E+00 |
| inorganic ion homeostasis (GO:0098771) | 46 | 1 | 4.09E-01 | 1.00E+00 |
| proteolysis (GO:0006508) | 46 | 1 | 4.09E-01 | 1.00E+00 |
| cellular metabolic process (GO:0044237) | 422 | 9 | 5.04E-02 | 1.00E+00 |
| regulation of phosphorus metabolic process (GO:0051174) | 49 | 1 | 4.29E-01 | 1.00E+00 |
| regulation of phosphate metabolic process (GO:0019220) | 49 | 1 | 4.29E-01 | 1.00E+00 |
| ion homeostasis (GO:0050801) | 49 | 1 | 4.29E-01 | 1.00E+00 |
| reproduction (GO:0000003) | 50 | 1 | 4.35E-01 | 1.00E+00 |
| response to chemical (GO:0042221) | 100 | 2 | 3.13E-01 | 1.00E+00 |
| enzyme linked receptor protein signaling pathway (GO:0007167) | 51 | 1 | 4.42E-01 | 1.00E+00 |
| cellular process (GO:0009987) | 1339 | 26 | 1.44E-03 | 1.00E+00 |
| RNA processing (GO:0006396) | 52 | 1 | 4.48E-01 | 1.00E+00 |
| protein-containing complex assembly (GO:0065003) | 52 | 1 | 4.48E-01 | 1.00E+00 |
| protein ubiquitination (GO:0016567) | 54 | 1 | 4.60E-01 | 1.00E+00 |
| cellular response to stimulus (GO:0051716) | 435 | 8 | 1.47E-01 | 1.00E+00 |
| signal transduction (GO:0007165) | 328 | 6 | 2.66E-01 | 1.00E+00 |
| regulation of response to stimulus (GO:0048583) | 55 | 1 | 4.66E-01 | 1.00E+00 |
| organic substance catabolic process (GO:1901575) | 57 | 1 | 4.78E-01 | 1.00E+00 |
| protein modification by small protein conjugation or removal (GO:0070647) | 59 | 1 | 4.90E-01 | 1.00E+00 |
| cytoskeleton organization (GO:0007010) | 59 | 1 | 4.90E-01 | 1.00E+00 |
| protein modification by small protein conjugation (GO:0032446) | 59 | 1 | 4.90E-01 | 1.00E+00 |
| chemical synaptic transmission (GO:0007268) | 61 | 1 | 5.01E-01 | 1.00E+00 |
| chemical homeostasis (GO:0048878) | 61 | 1 | 5.01E-01 | 1.00E+00 |
| anterograde trans-synaptic signaling (GO:0098916) | 61 | 1 | 5.01E-01 | 1.00E+00 |
| trans-synaptic signaling (GO:0099537) | 62 | 1 | 5.07E-01 | 1.00E+00 |
| synaptic signaling (GO:0099536) | 62 | 1 | 5.07E-01 | 1.00E+00 |
| cellular response to stress (GO:0033554) | 124 | 2 | 6.50E-01 | 1.00E+00 |
| protein-containing complex subunit organization (GO:0043933) | 62 | 1 | 5.07E-01 | 1.00E+00 |
| cellular lipid metabolic process (GO:0044255) | 67 | 1 | 5.34E-01 | 1.00E+00 |
| transcription, DNA-templated (GO:0006351) | 277 | 4 | 5.51E-01 | 1.00E+00 |
| anatomical structure development (GO:0048856) | 70 | 1 | 5.50E-01 | 1.00E+00 |
| regulation of cellular process (GO:0050794) | 211 | 3 | 5.12E-01 | 1.00E+00 |
| cell adhesion (GO:0007155) | 71 | 1 | 5.55E-01 | 1.00E+00 |
| biological adhesion (GO:0022610) | 71 | 1 | 5.55E-01 | 1.00E+00 |
| catabolic process (GO:0009056) | 72 | 1 | 5.60E-01 | 1.00E+00 |
| organic substance metabolic process (GO:0071704) | 866 | 12 | 4.69E-01 | 1.00E+00 |
| cellular component organization (GO:0016043) | 363 | 5 | 5.99E-01 | 1.00E+00 |
| gene expression (GO:0010467) | 441 | 6 | 6.30E-01 | 1.00E+00 |
| metabolic process (GO:0008152) | 1035 | 14 | 4.01E-01 | 1.00E+00 |
| homeostatic process (GO:0042592) | 75 | 1 | 5.74E-01 | 1.00E+00 |
| macromolecule metabolic process (GO:0043170) | 615 | 8 | 6.78E-01 | 1.00E+00 |
| organic substance biosynthetic process (GO:1901576) | 81 | 1 | 6.02E-01 | 1.00E+00 |
| regulation of metabolic process (GO:0019222) | 333 | 4 | 7.86E-01 | 1.00E+00 |
| regulation of biological process (GO:0050789) | 583 | 7 | 8.32E-01 | 1.00E+00 |
| biological regulation (GO:0065007) | 668 | 8 | 8.41E-01 | 1.00E+00 |
| nervous system process (GO:0050877) | 88 | 1 | 1.00E+00 | 1.00E+00 |
| organelle organization (GO:0006996) | 188 | 2 | 1.00E+00 | 1.00E+00 |
| developmental process (GO:0032502) | 99 | 1 | 1.00E+00 | 1.00E+00 |
| cellular protein localization (GO:0034613) | 207 | 2 | 1.00E+00 | 1.00E+00 |
| cellular macromolecule localization (GO:0070727) | 208 | 2 | 1.00E+00 | 1.00E+00 |
| system process (GO:0003008) | 110 | 1 | 1.00E+00 | 1.00E+00 |
| cellular localization (GO:0051641) | 235 | 2 | 1.00E+00 | 1.00E+00 |
| multicellular organismal process (GO:0032501) | 236 | 2 | 1.00E+00 | 1.00E+00 |
| Unclassified (UNCLASSIFIED) | 2019 | 16 | 5.98E-02 | 1.00E+00 |
| cell surface receptor signaling pathway (GO:0007166) | 131 | 1 | 1.00E+00 | 1.00E+00 |
| establishment of localization (GO:0051234) | 262 | 2 | 1.00E+00 | 1.00E+00 |
| transport (GO:0006810) | 262 | 2 | 1.00E+00 | 1.00E+00 |
| regulation of biological quality (GO:0065008) | 137 | 1 | 1.00E+00 | 1.00E+00 |
| localization (GO:0051179) | 457 | 3 | 4.78E-01 | 1.00E+00 |
| intracellular protein transport (GO:0006886) | 169 | 1 | 1.00E+00 | 1.00E+00 |
| regulation of biosynthetic process (GO:0009889) | 181 | 1 | 7.22E-01 | 1.00E+00 |
| regulation of cellular biosynthetic process (GO:0031326) | 181 | 1 | 7.22E-01 | 1.00E+00 |
| transcription by RNA polymerase II (GO:0006366) | 227 | 1 | 5.17E-01 | 1.00E+00 |

Table S26. GO analysis in Struthioniformes in the 2-times hypothesis using PANTHER GO-Slim Cellular Component. P values are calculated by Fisher’s exact test and FDR values are calculated by the Benjamini–Hochberg procedure.

| GO term | Number of genes in reference | Number of genes in query | P-values | FDR values |
| --- | --- | --- | --- | --- |
| neuromuscular junction (GO:0031594) | 2 | 1 | 3.29E-02 | 1.00E+00 |
| basolateral plasma membrane (GO:0016323) | 3 | 1 | 4.36E-02 | 1.00E+00 |
| polymeric cytoskeletal fiber (GO:0099513) | 3 | 1 | 4.36E-02 | 1.00E+00 |
| oligosaccharyltransferase complex (GO:0008250) | 3 | 1 | 4.36E-02 | 1.00E+00 |
| transcription factor TFIIIC complex (GO:0000127) | 3 | 1 | 4.36E-02 | 1.00E+00 |
| synapse (GO:0045202) | 3 | 1 | 4.36E-02 | 1.00E+00 |
| membrane region (GO:0098589) | 4 | 1 | 5.42E-02 | 1.00E+00 |
| membrane raft (GO:0045121) | 4 | 1 | 5.42E-02 | 1.00E+00 |
| membrane microdomain (GO:0098857) | 4 | 1 | 5.42E-02 | 1.00E+00 |
| organelle part (GO:0044422) | 5 | 1 | 6.47E-02 | 1.00E+00 |
| ionotropic glutamate receptor complex (GO:0008328) | 5 | 1 | 6.47E-02 | 1.00E+00 |
| spindle pole (GO:0000922) | 5 | 1 | 6.47E-02 | 1.00E+00 |
| kinetochore (GO:0000776) | 5 | 1 | 6.47E-02 | 1.00E+00 |
| anaphase-promoting complex (GO:0005680) | 5 | 1 | 6.47E-02 | 1.00E+00 |
| chromosome, centromeric region (GO:0000775) | 6 | 1 | 7.51E-02 | 1.00E+00 |
| SCF ubiquitin ligase complex (GO:0019005) | 8 | 1 | 9.55E-02 | 1.00E+00 |
| whole membrane (GO:0098805) | 8 | 1 | 9.55E-02 | 1.00E+00 |
| chromosomal region (GO:0098687) | 8 | 1 | 9.55E-02 | 1.00E+00 |
| cyclin-dependent protein kinase holoenzyme complex (GO:0000307) | 10 | 1 | 1.15E-01 | 1.00E+00 |
| spindle (GO:0005819) | 10 | 1 | 1.15E-01 | 1.00E+00 |
| cytoskeletal part (GO:0044430) | 11 | 1 | 1.25E-01 | 1.00E+00 |
| postsynaptic density (GO:0014069) | 11 | 1 | 1.25E-01 | 1.00E+00 |
| microtubule organizing center part (GO:0044450) | 35 | 3 | 8.20E-03 | 1.00E+00 |
| centriole (GO:0005814) | 35 | 3 | 8.20E-03 | 1.00E+00 |
| cullin-RING ubiquitin ligase complex (GO:0031461) | 13 | 1 | 1.45E-01 | 1.00E+00 |
| postsynaptic membrane (GO:0045211) | 13 | 1 | 1.45E-01 | 1.00E+00 |
| external side of plasma membrane (GO:0009897) | 14 | 1 | 1.54E-01 | 1.00E+00 |
| postsynapse (GO:0098794) | 14 | 1 | 1.54E-01 | 1.00E+00 |
| centrosome (GO:0005813) | 29 | 2 | 4.56E-02 | 1.00E+00 |
| microtubule organizing center (GO:0005815) | 44 | 3 | 1.47E-02 | 1.00E+00 |
| nuclear transcription factor complex (GO:0044798) | 15 | 1 | 1.64E-01 | 1.00E+00 |
| cytosolic ribosome (GO:0022626) | 18 | 1 | 1.91E-01 | 1.00E+00 |
| leaflet of membrane bilayer (GO:0097478) | 24 | 1 | 2.44E-01 | 1.00E+00 |
| ubiquitin ligase complex (GO:0000151) | 24 | 1 | 2.44E-01 | 1.00E+00 |
| cytosolic part (GO:0044445) | 26 | 1 | 2.60E-01 | 1.00E+00 |
| endoplasmic reticulum membrane (GO:0005789) | 28 | 1 | 2.77E-01 | 1.00E+00 |
| endoplasmic reticulum subcompartment (GO:0098827) | 30 | 1 | 2.93E-01 | 1.00E+00 |
| cytoskeleton (GO:0005856) | 154 | 5 | 3.00E-02 | 1.00E+00 |
| microtubule cytoskeleton (GO:0015630) | 93 | 3 | 8.92E-02 | 1.00E+00 |
| cell surface (GO:0009986) | 33 | 1 | 3.16E-01 | 1.00E+00 |
| neuron projection (GO:0043005) | 39 | 1 | 3.61E-01 | 1.00E+00 |
| microtubule (GO:0005874) | 39 | 1 | 3.61E-01 | 1.00E+00 |
| Golgi apparatus (GO:0005794) | 47 | 1 | 4.16E-01 | 1.00E+00 |
| integral component of plasma membrane (GO:0005887) | 144 | 3 | 2.22E-01 | 1.00E+00 |
| receptor complex (GO:0043235) | 53 | 1 | 4.54E-01 | 1.00E+00 |
| membrane part (GO:0044425) | 221 | 4 | 3.13E-01 | 1.00E+00 |
| membrane (GO:0016020) | 225 | 4 | 3.20E-01 | 1.00E+00 |
| intracellular non-membrane-bounded organelle (GO:0043232) | 284 | 5 | 2.49E-01 | 1.00E+00 |
| non-membrane-bounded organelle (GO:0043228) | 284 | 5 | 2.49E-01 | 1.00E+00 |
| actin cytoskeleton (GO:0015629) | 58 | 1 | 4.84E-01 | 1.00E+00 |
| cytosol (GO:0005829) | 176 | 3 | 4.49E-01 | 1.00E+00 |
| integral component of membrane (GO:0016021) | 184 | 3 | 4.62E-01 | 1.00E+00 |
| intrinsic component of membrane (GO:0031224) | 185 | 3 | 4.64E-01 | 1.00E+00 |
| ribonucleoprotein complex (GO:1990904) | 68 | 1 | 5.39E-01 | 1.00E+00 |
| organelle (GO:0043226) | 903 | 13 | 2.91E-01 | 1.00E+00 |
| organelle subcompartment (GO:0031984) | 72 | 1 | 5.60E-01 | 1.00E+00 |
| cell (GO:0005623) | 1225 | 17 | 3.35E-01 | 1.00E+00 |
| intracellular (GO:0005622) | 1013 | 14 | 3.93E-01 | 1.00E+00 |
| nucleus (GO:0005634) | 513 | 7 | 5.07E-01 | 1.00E+00 |
| intracellular membrane-bounded organelle (GO:0043231) | 527 | 7 | 6.57E-01 | 1.00E+00 |
| intracellular organelle (GO:0043229) | 528 | 7 | 6.57E-01 | 1.00E+00 |
| intracellular part (GO:0044424) | 908 | 12 | 4.82E-01 | 1.00E+00 |
| cell part (GO:0044464) | 1219 | 16 | 5.20E-01 | 1.00E+00 |
| neuron part (GO:0097458) | 77 | 1 | 5.84E-01 | 1.00E+00 |
| plasma membrane (GO:0005886) | 483 | 6 | 8.17E-01 | 1.00E+00 |
| cell periphery (GO:0071944) | 493 | 6 | 8.19E-01 | 1.00E+00 |
| cytoplasm (GO:0005737) | 853 | 10 | 8.56E-01 | 1.00E+00 |
| endomembrane system (GO:0012505) | 177 | 2 | 1.00E+00 | 1.00E+00 |
| cytoplasmic part (GO:0044444) | 540 | 6 | 1.00E+00 | 1.00E+00 |
| vacuole (GO:0005773) | 282 | 3 | 1.00E+00 | 1.00E+00 |
| plasma membrane region (GO:0098590) | 98 | 1 | 1.00E+00 | 1.00E+00 |
| Unclassified (UNCLASSIFIED) | 2289 | 22 | 3.11E-01 | 1.00E+00 |
| chromosomal part (GO:0044427) | 107 | 1 | 1.00E+00 | 1.00E+00 |
| nuclear part (GO:0044428) | 227 | 2 | 1.00E+00 | 1.00E+00 |
| plasma membrane part (GO:0044459) | 115 | 1 | 1.00E+00 | 1.00E+00 |
| chromosome (GO:0005694) | 117 | 1 | 1.00E+00 | 1.00E+00 |
| membrane-bounded organelle (GO:0043227) | 147 | 1 | 1.00E+00 | 1.00E+00 |
| protein-containing complex (GO:0032991) | 378 | 2 | 4.36E-01 | 1.00E+00 |

Table S27. GO analysis in Struthioniformes in the 2-times hypothesis using PANTHER GO-Slim Molecular Function. P values are calculated by Fisher’s exact test and FDR values are calculated by the Benjamini–Hochberg procedure.

| GO term | Number of genes in reference | Number of genes in query | P-values | FDR values |
| --- | --- | --- | --- | --- |
| oxidoreductase activity, acting on the CH-CH group of donors, NAD or NADP as acceptor (GO:0016628) | 1 | 1 | 2.20E-02 | 1.00E+00 |
| protein tyrosine kinase binding (GO:1990782) | 1 | 1 | 2.20E-02 | 1.00E+00 |
| microtubule plus-end binding (GO:0051010) | 2 | 1 | 3.29E-02 | 1.00E+00 |
| oligosaccharyl transferase activity (GO:0004576) | 4 | 1 | 5.42E-02 | 1.00E+00 |
| protein binding, bridging (GO:0030674) | 4 | 1 | 5.42E-02 | 1.00E+00 |
| mRNA 3'-UTR binding (GO:0003730) | 7 | 1 | 8.53E-02 | 1.00E+00 |
| antiporter activity (GO:0015297) | 7 | 1 | 8.53E-02 | 1.00E+00 |
| active transmembrane transporter activity (GO:0022804) | 8 | 1 | 9.55E-02 | 1.00E+00 |
| cadherin binding (GO:0045296) | 8 | 1 | 9.55E-02 | 1.00E+00 |
| secondary active transmembrane transporter activity (GO:0015291) | 8 | 1 | 9.55E-02 | 1.00E+00 |
| oxidoreductase activity, acting on the CH-CH group of donors (GO:0016627) | 10 | 1 | 1.15E-01 | 1.00E+00 |
| protein serine/threonine phosphatase activity (GO:0004722) | 12 | 1 | 1.35E-01 | 1.00E+00 |
| translation regulator activity (GO:0045182) | 12 | 1 | 1.35E-01 | 1.00E+00 |
| protein homodimerization activity (GO:0042803) | 12 | 1 | 1.35E-01 | 1.00E+00 |
| protein dimerization activity (GO:0046983) | 13 | 1 | 1.45E-01 | 1.00E+00 |
| cell adhesion molecule binding (GO:0050839) | 13 | 1 | 1.45E-01 | 1.00E+00 |
| cofactor binding (GO:0048037) | 13 | 1 | 1.45E-01 | 1.00E+00 |
| ribonucleoprotein complex binding (GO:0043021) | 15 | 1 | 1.64E-01 | 1.00E+00 |
| cytokine activity (GO:0005125) | 15 | 1 | 1.64E-01 | 1.00E+00 |
| drug binding (GO:0008144) | 15 | 1 | 1.64E-01 | 1.00E+00 |
| protein kinase binding (GO:0019901) | 34 | 2 | 5.96E-02 | 1.00E+00 |
| oxidoreductase activity, acting on the CH-OH group of donors, NAD or NADP as acceptor (GO:0016616) | 17 | 1 | 1.82E-01 | 1.00E+00 |
| transmembrane receptor protein kinase activity (GO:0019199) | 17 | 1 | 1.82E-01 | 1.00E+00 |
| cyclin-dependent protein serine/threonine kinase activity (GO:0004693) | 17 | 1 | 1.82E-01 | 1.00E+00 |
| transmembrane receptor protein tyrosine kinase activity (GO:0004714) | 17 | 1 | 1.82E-01 | 1.00E+00 |
| protein kinase regulator activity (GO:0019887) | 17 | 1 | 1.82E-01 | 1.00E+00 |
| kinase binding (GO:0019900) | 36 | 2 | 6.56E-02 | 1.00E+00 |
| oxidoreductase activity, acting on CH-OH group of donors (GO:0016614) | 18 | 1 | 1.91E-01 | 1.00E+00 |
| sodium ion transmembrane transporter activity (GO:0015081) | 19 | 1 | 2.00E-01 | 1.00E+00 |
| ATPase activity, coupled to transmembrane movement of substances (GO:0042626) | 19 | 1 | 2.00E-01 | 1.00E+00 |
| mRNA binding (GO:0003729) | 41 | 2 | 8.15E-02 | 1.00E+00 |
| ATPase activity, coupled to movement of substances (GO:0043492) | 22 | 1 | 2.27E-01 | 1.00E+00 |
| signaling receptor binding (GO:0005102) | 68 | 3 | 4.31E-02 | 1.00E+00 |
| transferase activity, transferring hexosyl groups (GO:0016758) | 23 | 1 | 2.35E-01 | 1.00E+00 |
| protein serine/threonine kinase activity (GO:0004674) | 96 | 4 | 2.39E-02 | 1.00E+00 |
| kinase regulator activity (GO:0019207) | 24 | 1 | 2.44E-01 | 1.00E+00 |
| protein kinase activity (GO:0004672) | 135 | 5 | 1.84E-02 | 1.00E+00 |
| potassium ion transmembrane transporter activity (GO:0015079) | 28 | 1 | 2.77E-01 | 1.00E+00 |
| anion transmembrane transporter activity (GO:0008509) | 30 | 1 | 2.93E-01 | 1.00E+00 |
| phosphoprotein phosphatase activity (GO:0004721) | 31 | 1 | 3.01E-01 | 1.00E+00 |
| phosphotransferase activity, alcohol group as acceptor (GO:0016773) | 156 | 5 | 3.14E-02 | 1.00E+00 |
| actin binding (GO:0003779) | 33 | 1 | 3.16E-01 | 1.00E+00 |
| ubiquitin-protein transferase activity (GO:0004842) | 67 | 2 | 1.77E-01 | 1.00E+00 |
| monovalent inorganic cation transmembrane transporter activity (GO:0015077) | 35 | 1 | 3.31E-01 | 1.00E+00 |
| receptor ligand activity (GO:0048018) | 35 | 1 | 3.31E-01 | 1.00E+00 |
| GTPase activator activity (GO:0005096) | 36 | 1 | 3.39E-01 | 1.00E+00 |
| receptor regulator activity (GO:0030545) | 37 | 1 | 3.46E-01 | 1.00E+00 |
| GTPase regulator activity (GO:0030695) | 37 | 1 | 3.46E-01 | 1.00E+00 |
| protein-containing complex binding (GO:0044877) | 38 | 1 | 3.54E-01 | 1.00E+00 |
| ubiquitin protein ligase activity (GO:0061630) | 38 | 1 | 3.54E-01 | 1.00E+00 |
| nucleoside-triphosphatase regulator activity (GO:0060589) | 38 | 1 | 3.54E-01 | 1.00E+00 |
| calcium ion binding (GO:0005509) | 39 | 1 | 3.61E-01 | 1.00E+00 |
| microtubule binding (GO:0008017) | 40 | 1 | 3.68E-01 | 1.00E+00 |
| metal ion transmembrane transporter activity (GO:0046873) | 40 | 1 | 3.68E-01 | 1.00E+00 |
| nucleotide binding (GO:0000166) | 40 | 1 | 3.68E-01 | 1.00E+00 |
| transferase activity, transferring phosphorus-containing groups (GO:0016772) | 205 | 5 | 8.07E-02 | 1.00E+00 |
| ubiquitin-like protein transferase activity (GO:0019787) | 86 | 2 | 2.55E-01 | 1.00E+00 |
| tubulin binding (GO:0015631) | 44 | 1 | 3.96E-01 | 1.00E+00 |
| transferase activity, transferring glycosyl groups (GO:0016757) | 44 | 1 | 3.96E-01 | 1.00E+00 |
| cytoskeletal protein binding (GO:0008092) | 95 | 2 | 2.92E-01 | 1.00E+00 |
| transferase activity (GO:0016740) | 451 | 9 | 9.23E-02 | 1.00E+00 |
| ion transmembrane transporter activity (GO:0015075) | 110 | 2 | 3.54E-01 | 1.00E+00 |
| phosphatase activity (GO:0016791) | 56 | 1 | 4.72E-01 | 1.00E+00 |
| ATPase activity, coupled (GO:0042623) | 60 | 1 | 4.96E-01 | 1.00E+00 |
| metal ion binding (GO:0046872) | 61 | 1 | 5.01E-01 | 1.00E+00 |
| oxidoreductase activity (GO:0016491) | 122 | 2 | 4.02E-01 | 1.00E+00 |
| protein binding (GO:0005515) | 563 | 9 | 2.79E-01 | 1.00E+00 |
| small molecule binding (GO:0036094) | 63 | 1 | 5.12E-01 | 1.00E+00 |
| molecular function regulator (GO:0098772) | 197 | 3 | 4.85E-01 | 1.00E+00 |
| transcription regulator activity (GO:0140110) | 201 | 3 | 4.93E-01 | 1.00E+00 |
| RNA binding (GO:0003723) | 135 | 2 | 6.64E-01 | 1.00E+00 |
| cation binding (GO:0043169) | 72 | 1 | 5.60E-01 | 1.00E+00 |
| molecular transducer activity (GO:0060089) | 152 | 2 | 6.88E-01 | 1.00E+00 |
| ATPase activity (GO:0016887) | 76 | 1 | 5.79E-01 | 1.00E+00 |
| inorganic cation transmembrane transporter activity (GO:0022890) | 76 | 1 | 5.79E-01 | 1.00E+00 |
| enzyme regulator activity (GO:0030234) | 153 | 2 | 6.90E-01 | 1.00E+00 |
| transmembrane transporter activity (GO:0022857) | 153 | 2 | 6.90E-01 | 1.00E+00 |
| catalytic activity (GO:0003824) | 1153 | 15 | 5.14E-01 | 1.00E+00 |
| enzyme binding (GO:0019899) | 158 | 2 | 6.97E-01 | 1.00E+00 |
| GTPase activity (GO:0003924) | 81 | 1 | 6.02E-01 | 1.00E+00 |
| phosphoric ester hydrolase activity (GO:0042578) | 82 | 1 | 6.07E-01 | 1.00E+00 |
| cation transmembrane transporter activity (GO:0008324) | 83 | 1 | 6.11E-01 | 1.00E+00 |
| DNA-binding transcription factor activity (GO:0003700) | 168 | 2 | 7.13E-01 | 1.00E+00 |
| binding (GO:0005488) | 1109 | 13 | 8.69E-01 | 1.00E+00 |
| nucleoside-triphosphatase activity (GO:0017111) | 172 | 2 | 7.19E-01 | 1.00E+00 |
| pyrophosphatase activity (GO:0016462) | 176 | 2 | 1.00E+00 | 1.00E+00 |
| hydrolase activity, acting on acid anhydrides, in phosphorus-containing anhydrides (GO:0016818) | 176 | 2 | 1.00E+00 | 1.00E+00 |
| hydrolase activity, acting on acid anhydrides (GO:0016817) | 176 | 2 | 1.00E+00 | 1.00E+00 |
| structural molecule activity (GO:0005198) | 94 | 1 | 1.00E+00 | 1.00E+00 |
| transporter activity (GO:0005215) | 196 | 2 | 1.00E+00 | 1.00E+00 |
| Unclassified (UNCLASSIFIED) | 2114 | 21 | 4.71E-01 | 1.00E+00 |
| ion binding (GO:0043167) | 121 | 1 | 1.00E+00 | 1.00E+00 |
| signaling receptor activity (GO:0038023) | 134 | 1 | 1.00E+00 | 1.00E+00 |
| hydrolase activity, acting on ester bonds (GO:0016788) | 146 | 1 | 1.00E+00 | 1.00E+00 |
| hydrolase activity (GO:0016787) | 490 | 3 | 3.61E-01 | 1.00E+00 |
| nucleic acid binding (GO:0003676) | 406 | 2 | 3.17E-01 | 1.00E+00 |
| heterocyclic compound binding (GO:1901363) | 417 | 2 | 3.20E-01 | 1.00E+00 |

Table S28. Pathway analysis for Struthioniformes in the 2-times hypothesis using PANTHER pathway. P values are calculated by Fisher’s exact test and FDR values are calculated by the Benjamini–Hochberg procedure.

| Pathway | Number of genes in reference | Number of genes in query | P-values | FDR values |
| --- | --- | --- | --- | --- |
| Pyrimidine Metabolism (P02771) | 5 | 1 | 6.47E-02 | 1.00E+00 |
| Toll receptor signaling pathway (P00054) | 14 | 2 | 1.31E-02 | 1.00E+00 |
| Cell cycle (P00013) | 7 | 1 | 8.53E-02 | 1.00E+00 |
| FAS signaling pathway (P00020) | 9 | 1 | 1.06E-01 | 1.00E+00 |
| p53 pathway feedback loops 2 (P04398) | 17 | 1 | 1.82E-01 | 1.00E+00 |
| Parkinson disease (P00049) | 18 | 1 | 1.91E-01 | 1.00E+00 |
| B cell activation (P00010) | 19 | 1 | 2.00E-01 | 1.00E+00 |
| Integrin signalling pathway (P00034) | 40 | 2 | 7.82E-02 | 1.00E+00 |
| p53 pathway (P00059) | 26 | 1 | 2.60E-01 | 1.00E+00 |
| FGF signaling pathway (P00021) | 26 | 1 | 2.60E-01 | 1.00E+00 |
| EGF receptor signaling pathway (P00018) | 35 | 1 | 3.31E-01 | 1.00E+00 |
| Wnt signaling pathway (P00057) | 56 | 1 | 4.72E-01 | 1.00E+00 |
| Gonadotropin-releasing hormone receptor pathway (P06664) | 61 | 1 | 5.01E-01 | 1.00E+00 |
| Unclassified (UNCLASSIFIED) | 3681 | 40 | 5.37E-01 | 1.00E+00 |

Table S29. GO analysis in ratites excluding Struthioniformes in the 2-times hypothesis using PANTHER GO-Slim Biological Process. P values are calculated by Fisher’s exact test and FDR values are calculated by the Benjamini–Hochberg procedure.

| GO term | Number of genes in reference | Number of genes in query | P-values | FDR values |
| --- | --- | --- | --- | --- |
| aromatic amino acid family catabolic process (GO:0009074) | 3 | 1 | 1.57E-02 | 1.00E+00 |
| cellular divalent inorganic cation homeostasis (GO:0072503) | 3 | 1 | 1.57E-02 | 1.00E+00 |
| divalent metal ion transport (GO:0070838) | 7 | 1 | 3.12E-02 | 1.00E+00 |
| divalent inorganic cation transport (GO:0072511) | 7 | 1 | 3.12E-02 | 1.00E+00 |
| cellular metal ion homeostasis (GO:0006875) | 7 | 1 | 3.12E-02 | 1.00E+00 |
| carboxylic acid metabolic process (GO:0019752) | 8 | 1 | 3.50E-02 | 1.00E+00 |
| monocarboxylic acid metabolic process (GO:0032787) | 8 | 1 | 3.50E-02 | 1.00E+00 |
| transition metal ion transport (GO:0000041) | 9 | 1 | 3.89E-02 | 1.00E+00 |
| transition metal ion homeostasis (GO:0055076) | 10 | 1 | 4.27E-02 | 1.00E+00 |
| metal ion transport (GO:0030001) | 10 | 1 | 4.27E-02 | 1.00E+00 |
| organic hydroxy compound metabolic process (GO:1901615) | 10 | 1 | 4.27E-02 | 1.00E+00 |
| oxoacid metabolic process (GO:0043436) | 11 | 1 | 4.65E-02 | 1.00E+00 |
| organic acid metabolic process (GO:0006082) | 12 | 1 | 5.02E-02 | 1.00E+00 |
| DNA recombination (GO:0006310) | 12 | 1 | 5.02E-02 | 1.00E+00 |
| mRNA processing (GO:0006397) | 13 | 1 | 5.40E-02 | 1.00E+00 |
| RNA 3'-end processing (GO:0031123) | 13 | 1 | 5.40E-02 | 1.00E+00 |
| metal ion homeostasis (GO:0055065) | 13 | 1 | 5.40E-02 | 1.00E+00 |
| import into cell (GO:0098657) | 14 | 1 | 5.78E-02 | 1.00E+00 |
| ion transmembrane transport (GO:0034220) | 15 | 1 | 6.15E-02 | 1.00E+00 |
| inorganic cation transmembrane transport (GO:0098662) | 15 | 1 | 6.15E-02 | 1.00E+00 |
| inorganic ion transmembrane transport (GO:0098660) | 15 | 1 | 6.15E-02 | 1.00E+00 |
| mRNA metabolic process (GO:0016071) | 15 | 1 | 6.15E-02 | 1.00E+00 |
| cellular amino acid catabolic process (GO:0009063) | 16 | 1 | 6.52E-02 | 1.00E+00 |
| membrane invagination (GO:0010324) | 19 | 1 | 7.63E-02 | 1.00E+00 |
| vesicle budding from membrane (GO:0006900) | 19 | 1 | 7.63E-02 | 1.00E+00 |
| positive regulation of nucleic acid-templated transcription (GO:1903508) | 20 | 1 | 8.00E-02 | 1.00E+00 |
| positive regulation of transcription, DNA-templated (GO:0045893) | 20 | 1 | 8.00E-02 | 1.00E+00 |
| positive regulation of RNA biosynthetic process (GO:1902680) | 20 | 1 | 8.00E-02 | 1.00E+00 |
| cation transport (GO:0006812) | 21 | 1 | 8.36E-02 | 1.00E+00 |
| drug metabolic process (GO:0017144) | 24 | 1 | 9.45E-02 | 1.00E+00 |
| tRNA metabolic process (GO:0006399) | 25 | 1 | 9.81E-02 | 1.00E+00 |
| divalent inorganic cation homeostasis (GO:0072507) | 30 | 1 | 1.16E-01 | 1.00E+00 |
| carboxylic acid catabolic process (GO:0046395) | 33 | 1 | 1.26E-01 | 1.00E+00 |
| organic acid catabolic process (GO:0016054) | 33 | 1 | 1.26E-01 | 1.00E+00 |
| ion transport (GO:0006811) | 33 | 1 | 1.26E-01 | 1.00E+00 |
| formation of translation initiation ternary complex (GO:0001677) | 69 | 2 | 3.11E-02 | 1.00E+00 |
| translational termination (GO:0006415) | 69 | 2 | 3.11E-02 | 1.00E+00 |
| translational elongation (GO:0006414) | 69 | 2 | 3.11E-02 | 1.00E+00 |
| catabolic process (GO:0009056) | 72 | 2 | 3.36E-02 | 1.00E+00 |
| cellular amino acid metabolic process (GO:0006520) | 40 | 1 | 1.50E-01 | 1.00E+00 |
| translation (GO:0006412) | 83 | 2 | 4.33E-02 | 1.00E+00 |
| DNA metabolic process (GO:0006259) | 42 | 1 | 1.57E-01 | 1.00E+00 |
| nucleic acid metabolic process (GO:0090304) | 131 | 3 | 1.46E-02 | 1.00E+00 |
| endocytosis (GO:0006897) | 44 | 1 | 1.64E-01 | 1.00E+00 |
| inorganic ion homeostasis (GO:0098771) | 46 | 1 | 1.71E-01 | 1.00E+00 |
| RNA metabolic process (GO:0016070) | 92 | 2 | 5.19E-02 | 1.00E+00 |
| proteolysis (GO:0006508) | 46 | 1 | 1.71E-01 | 1.00E+00 |
| ion homeostasis (GO:0050801) | 49 | 1 | 1.81E-01 | 1.00E+00 |
| positive regulation of transcription by RNA polymerase II (GO:0045944) | 51 | 1 | 1.87E-01 | 1.00E+00 |
| RNA processing (GO:0006396) | 52 | 1 | 1.90E-01 | 1.00E+00 |
| ncRNA metabolic process (GO:0034660) | 53 | 1 | 1.94E-01 | 1.00E+00 |
| organic substance catabolic process (GO:1901575) | 57 | 1 | 2.06E-01 | 1.00E+00 |
| membrane organization (GO:0061024) | 58 | 1 | 2.10E-01 | 1.00E+00 |
| chemical homeostasis (GO:0048878) | 61 | 1 | 2.19E-01 | 1.00E+00 |
| organonitrogen compound metabolic process (GO:1901564) | 65 | 1 | 2.32E-01 | 1.00E+00 |
| protein metabolic process (GO:0019538) | 68 | 1 | 2.41E-01 | 1.00E+00 |
| gene expression (GO:0010467) | 441 | 6 | 5.54E-03 | 1.00E+00 |
| homeostatic process (GO:0042592) | 75 | 1 | 2.62E-01 | 1.00E+00 |
| macromolecule metabolic process (GO:0043170) | 615 | 7 | 6.64E-03 | 1.00E+00 |
| regulation of RNA biosynthetic process (GO:2001141) | 179 | 2 | 1.59E-01 | 1.00E+00 |
| regulation of biosynthetic process (GO:0009889) | 181 | 2 | 1.61E-01 | 1.00E+00 |
| regulation of cellular biosynthetic process (GO:0031326) | 181 | 2 | 1.61E-01 | 1.00E+00 |
| transcription, DNA-templated (GO:0006351) | 277 | 3 | 9.42E-02 | 1.00E+00 |
| organic substance metabolic process (GO:0071704) | 866 | 8 | 1.19E-02 | 1.00E+00 |
| transcription by RNA polymerase II (GO:0006366) | 227 | 2 | 2.28E-01 | 1.00E+00 |
| metabolic process (GO:0008152) | 1035 | 9 | 9.99E-03 | 1.00E+00 |
| primary metabolic process (GO:0044238) | 118 | 1 | 3.80E-01 | 1.00E+00 |
| establishment of localization (GO:0051234) | 262 | 2 | 2.81E-01 | 1.00E+00 |
| transport (GO:0006810) | 262 | 2 | 2.81E-01 | 1.00E+00 |
| vesicle-mediated transport (GO:0016192) | 133 | 1 | 4.17E-01 | 1.00E+00 |
| regulation of biological quality (GO:0065008) | 137 | 1 | 4.26E-01 | 1.00E+00 |
| localization (GO:0051179) | 457 | 3 | 4.17E-01 | 1.00E+00 |
| regulation of transcription by RNA polymerase II (GO:0006357) | 156 | 1 | 4.69E-01 | 1.00E+00 |
| regulation of transcription, DNA-templated (GO:0006355) | 163 | 1 | 4.84E-01 | 1.00E+00 |
| regulation of nucleic acid-templated transcription (GO:1903506) | 163 | 1 | 4.84E-01 | 1.00E+00 |
| regulation of metabolic process (GO:0019222) | 333 | 2 | 3.86E-01 | 1.00E+00 |
| biological regulation (GO:0065007) | 668 | 4 | 3.24E-01 | 1.00E+00 |
| intracellular protein transport (GO:0006886) | 169 | 1 | 4.97E-01 | 1.00E+00 |
| response to stimulus (GO:0050896) | 180 | 1 | 5.19E-01 | 1.00E+00 |
| regulation of biological process (GO:0050789) | 583 | 3 | 4.97E-01 | 1.00E+00 |
| cellular protein localization (GO:0034613) | 207 | 1 | 5.70E-01 | 1.00E+00 |
| cellular macromolecule localization (GO:0070727) | 208 | 1 | 5.72E-01 | 1.00E+00 |
| cellular metabolic process (GO:0044237) | 422 | 2 | 6.81E-01 | 1.00E+00 |
| cellular protein modification process (GO:0006464) | 216 | 1 | 5.86E-01 | 1.00E+00 |
| cellular protein metabolic process (GO:0044267) | 231 | 1 | 6.11E-01 | 1.00E+00 |
| cellular macromolecule metabolic process (GO:0044260) | 231 | 1 | 6.11E-01 | 1.00E+00 |
| cellular localization (GO:0051641) | 235 | 1 | 6.17E-01 | 1.00E+00 |
| cellular component organization (GO:0016043) | 363 | 1 | 1.00E+00 | 1.00E+00 |
| Unclassified (UNCLASSIFIED) | 2019 | 5 | 2.23E-01 | 1.00E+00 |
| cellular process (GO:0009987) | 1339 | 3 | 2.99E-01 | 1.00E+00 |

Table S30. GO analysis in ratites excluding Struthioniformes in the 2-times hypothesis using PANTHER GO-Slim Cellular Component. P values are calculated by Fisher’s exact test and FDR values are calculated by the Benjamini–Hochberg procedure.

| GO term | Number of genes in reference | Number of genes in query | P-values | FDR values |
| --- | --- | --- | --- | --- |
| transcription factor TFIID complex (GO:0005669) | 4 | 1 | 1.96E-02 | 1.00E+00 |
| mRNA cleavage factor complex (GO:0005849) | 4 | 1 | 1.96E-02 | 1.00E+00 |
| DNA-directed RNA polymerase II, holoenzyme (GO:0016591) | 24 | 1 | 9.45E-02 | 1.00E+00 |
| nuclear DNA-directed RNA polymerase complex (GO:0055029) | 32 | 1 | 1.23E-01 | 1.00E+00 |
| nuclear chromatin (GO:0000790) | 76 | 1 | 2.65E-01 | 1.00E+00 |
| mitochondrion (GO:0005739) | 77 | 1 | 2.68E-01 | 1.00E+00 |
| chromatin (GO:0000785) | 86 | 1 | 2.94E-01 | 1.00E+00 |
| chromosomal part (GO:0044427) | 107 | 1 | 3.51E-01 | 1.00E+00 |
| chromosome (GO:0005694) | 117 | 1 | 3.77E-01 | 1.00E+00 |
| integral component of plasma membrane (GO:0005887) | 144 | 1 | 4.42E-01 | 1.00E+00 |
| intracellular (GO:0005622) | 1013 | 7 | 1.47E-01 | 1.00E+00 |
| cytoplasm (GO:0005737) | 853 | 5 | 3.58E-01 | 1.00E+00 |
| nucleus (GO:0005634) | 513 | 3 | 4.48E-01 | 1.00E+00 |
| cell part (GO:0044464) | 1219 | 7 | 2.82E-01 | 1.00E+00 |
| cell (GO:0005623) | 1225 | 7 | 2.83E-01 | 1.00E+00 |
| intracellular membrane-bounded organelle (GO:0043231) | 527 | 3 | 4.57E-01 | 1.00E+00 |
| intracellular organelle (GO:0043229) | 528 | 3 | 4.57E-01 | 1.00E+00 |
| intracellular part (GO:0044424) | 908 | 5 | 3.81E-01 | 1.00E+00 |
| integral component of membrane (GO:0016021) | 184 | 1 | 5.27E-01 | 1.00E+00 |
| intrinsic component of membrane (GO:0031224) | 185 | 1 | 5.29E-01 | 1.00E+00 |
| protein-containing complex (GO:0032991) | 378 | 2 | 6.58E-01 | 1.00E+00 |
| membrane part (GO:0044425) | 221 | 1 | 5.94E-01 | 1.00E+00 |
| membrane (GO:0016020) | 225 | 1 | 6.01E-01 | 1.00E+00 |
| organelle (GO:0043226) | 903 | 4 | 7.68E-01 | 1.00E+00 |
| nuclear part (GO:0044428) | 227 | 1 | 6.04E-01 | 1.00E+00 |
| cytoplasmic part (GO:0044444) | 540 | 2 | 1.00E+00 | 1.00E+00 |
| intracellular non-membrane-bounded organelle (GO:0043232) | 284 | 1 | 1.00E+00 | 1.00E+00 |
| non-membrane-bounded organelle (GO:0043228) | 284 | 1 | 1.00E+00 | 1.00E+00 |
| Unclassified (UNCLASSIFIED) | 2289 | 6 | 1.50E-01 | 1.00E+00 |

Table S31. GO analysis in ratites excluding Struthioniformes in the 2-times hypothesis using PANTHER GO-Slim Molecular Function. P values are calculated by Fisher’s exact test and FDR values are calculated by the Benjamini–Hochberg procedure.

| GO term | Number of genes in reference | Number of genes in query | P-values | FDR values |
| --- | --- | --- | --- | --- |
| translation initiation factor binding (GO:0031369) | 3 | 1 | 1.57E-02 | 1.00E+00 |
| translation initiation factor activity (GO:0003743) | 11 | 1 | 4.65E-02 | 1.00E+00 |
| endodeoxyribonuclease activity (GO:0004520) | 12 | 1 | 5.02E-02 | 1.00E+00 |
| endonuclease activity (GO:0004519) | 12 | 1 | 5.02E-02 | 1.00E+00 |
| ribonuclease activity (GO:0004540) | 13 | 1 | 5.40E-02 | 1.00E+00 |
| endoribonuclease activity (GO:0004521) | 13 | 1 | 5.40E-02 | 1.00E+00 |
| GDP binding (GO:0019003) | 14 | 1 | 5.78E-02 | 1.00E+00 |
| Rab GTPase binding (GO:0017137) | 23 | 1 | 9.09E-02 | 1.00E+00 |
| GTP binding (GO:0005525) | 24 | 1 | 9.45E-02 | 1.00E+00 |
| purine ribonucleoside binding (GO:0032550) | 24 | 1 | 9.45E-02 | 1.00E+00 |
| ribonucleoside binding (GO:0032549) | 24 | 1 | 9.45E-02 | 1.00E+00 |
| nucleoside binding (GO:0001882) | 24 | 1 | 9.45E-02 | 1.00E+00 |
| catalytic activity, acting on RNA (GO:0140098) | 58 | 2 | 2.27E-02 | 1.00E+00 |
| phosphoprotein phosphatase activity (GO:0004721) | 31 | 1 | 1.19E-01 | 1.00E+00 |
| GTPase binding (GO:0051020) | 64 | 2 | 2.72E-02 | 1.00E+00 |
| guanyl-nucleotide exchange factor activity (GO:0005085) | 33 | 1 | 1.26E-01 | 1.00E+00 |
| Ras GTPase binding (GO:0017016) | 34 | 1 | 1.30E-01 | 1.00E+00 |
| small GTPase binding (GO:0031267) | 35 | 1 | 1.33E-01 | 1.00E+00 |
| GTPase activator activity (GO:0005096) | 36 | 1 | 1.37E-01 | 1.00E+00 |
| nuclease activity (GO:0004518) | 37 | 1 | 1.40E-01 | 1.00E+00 |
| GTPase regulator activity (GO:0030695) | 37 | 1 | 1.40E-01 | 1.00E+00 |
| nucleoside-triphosphatase regulator activity (GO:0060589) | 38 | 1 | 1.44E-01 | 1.00E+00 |
| metal ion transmembrane transporter activity (GO:0046873) | 40 | 1 | 1.50E-01 | 1.00E+00 |
| mRNA binding (GO:0003729) | 41 | 1 | 1.54E-01 | 1.00E+00 |
| organic cyclic compound binding (GO:0097159) | 42 | 1 | 1.57E-01 | 1.00E+00 |
| transcription factor binding (GO:0008134) | 42 | 1 | 1.57E-01 | 1.00E+00 |
| RNA polymerase II transcription factor activity, sequence-specific DNA binding (GO:0000981) | 47 | 1 | 1.74E-01 | 1.00E+00 |
| phosphatase activity (GO:0016791) | 56 | 1 | 2.03E-01 | 1.00E+00 |
| transcription regulatory region sequence-specific DNA binding (GO:0000976) | 61 | 1 | 2.19E-01 | 1.00E+00 |
| anion binding (GO:0043168) | 67 | 1 | 2.38E-01 | 1.00E+00 |
| RNA binding (GO:0003723) | 135 | 2 | 1.00E-01 | 1.00E+00 |
| hydrolase activity, acting on ester bonds (GO:0016788) | 146 | 2 | 1.14E-01 | 1.00E+00 |
| inorganic cation transmembrane transporter activity (GO:0022890) | 76 | 1 | 2.65E-01 | 1.00E+00 |
| transcription regulatory region DNA binding (GO:0044212) | 76 | 1 | 2.65E-01 | 1.00E+00 |
| enzyme binding (GO:0019899) | 158 | 2 | 1.30E-01 | 1.00E+00 |
| GTPase activity (GO:0003924) | 81 | 1 | 2.79E-01 | 1.00E+00 |
| nucleic acid binding (GO:0003676) | 406 | 5 | 1.83E-02 | 1.00E+00 |
| phosphoric ester hydrolase activity (GO:0042578) | 82 | 1 | 2.82E-01 | 1.00E+00 |
| cation transmembrane transporter activity (GO:0008324) | 83 | 1 | 2.85E-01 | 1.00E+00 |
| heterocyclic compound binding (GO:1901363) | 417 | 5 | 2.03E-02 | 1.00E+00 |
| DNA-binding transcription factor activity (GO:0003700) | 168 | 2 | 1.43E-01 | 1.00E+00 |
| ligase activity (GO:0016874) | 90 | 1 | 3.05E-01 | 1.00E+00 |
| transcription regulator activity (GO:0140110) | 201 | 2 | 1.90E-01 | 1.00E+00 |
| ion transmembrane transporter activity (GO:0015075) | 110 | 1 | 3.59E-01 | 1.00E+00 |
| DNA binding (GO:0003677) | 240 | 2 | 2.48E-01 | 1.00E+00 |
| ion binding (GO:0043167) | 121 | 1 | 3.87E-01 | 1.00E+00 |
| hydrolase activity (GO:0016787) | 490 | 4 | 1.22E-01 | 1.00E+00 |
| protein binding (GO:0005515) | 563 | 4 | 2.67E-01 | 1.00E+00 |
| enzyme regulator activity (GO:0030234) | 153 | 1 | 4.63E-01 | 1.00E+00 |
| transmembrane transporter activity (GO:0022857) | 153 | 1 | 4.63E-01 | 1.00E+00 |
| binding (GO:0005488) | 1109 | 7 | 1.67E-01 | 1.00E+00 |
| nucleoside-triphosphatase activity (GO:0017111) | 172 | 1 | 5.03E-01 | 1.00E+00 |
| pyrophosphatase activity (GO:0016462) | 176 | 1 | 5.11E-01 | 1.00E+00 |
| hydrolase activity, acting on acid anhydrides, in phosphorus-containing anhydrides (GO:0016818) | 176 | 1 | 5.11E-01 | 1.00E+00 |
| hydrolase activity, acting on acid anhydrides (GO:0016817) | 176 | 1 | 5.11E-01 | 1.00E+00 |
| catalytic activity (GO:0003824) | 1153 | 6 | 4.20E-01 | 1.00E+00 |
| transporter activity (GO:0005215) | 196 | 1 | 5.50E-01 | 1.00E+00 |
| molecular function regulator (GO:0098772) | 197 | 1 | 5.52E-01 | 1.00E+00 |
| Unclassified (UNCLASSIFIED) | 2114 | 5 | 1.43E-01 | 1.00E+00 |

Table S32. Pathway analysis for ratites excluding Struthioniformes in the 2-times hypothesis using PANTHER pathway. P values are calculated by Fisher’s exact test and FDR values are calculated by the Benjamini–Hochberg procedure.

| GO term | Number of genes in reference | Number of genes in query | P-values | FDR values |
| --- | --- | --- | --- | --- |
| Circadian clock system (P00015) | 4 | 1 | 1.96E-02 | 1.00E+00 |
| JAK/STAT signaling pathway (P00038) | 6 | 1 | 2.74E-02 | 1.00E+00 |
| Hedgehog signaling pathway (P00025) | 8 | 1 | 3.50E-02 | 1.00E+00 |
| Parkinson disease (P00049) | 18 | 1 | 7.26E-02 | 1.00E+00 |
| B cell activation (P00010) | 19 | 1 | 7.63E-02 | 1.00E+00 |
| CCKR signaling map (P06959) | 42 | 2 | 1.26E-02 | 1.00E+00 |
| T cell activation (P00053) | 21 | 1 | 8.36E-02 | 1.00E+00 |
| Wnt signaling pathway (P00057) | 56 | 1 | 2.03E-01 | 1.00E+00 |
| Unclassified (UNCLASSIFIED) | 3681 | 14 | 7.23E-01 | 1.00E+00 |

Table S33. GO analysis in Sphenisciformes in the 4-times hypothesis using PANTHER GO-Slim Biological Process. P values are calculated by Fisher’s exact test and FDR values are calculated by the Benjamini–Hochberg procedure.

| GO term | Number of genes in reference | Number of genes in query | P-values | FDR values |
| --- | --- | --- | --- | --- |
| regulation of exit from mitosis (GO:0007096) | 1 | 1 | 6.46E-03 | 1.00E+00 |
| glycosaminoglycan biosynthetic process (GO:0006024) | 2 | 1 | 9.68E-03 | 1.00E+00 |
| cellular response to light stimulus (GO:0071482) | 3 | 1 | 1.29E-02 | 1.00E+00 |
| cell cycle arrest (GO:0007050) | 3 | 1 | 1.29E-02 | 1.00E+00 |
| regulation of cytokinesis (GO:0032465) | 3 | 1 | 1.29E-02 | 1.00E+00 |
| regulation of cell division (GO:0051302) | 4 | 1 | 1.61E-02 | 1.00E+00 |
| detection of stimulus (GO:0051606) | 4 | 1 | 1.61E-02 | 1.00E+00 |
| response to metal ion (GO:0010038) | 9 | 2 | 5.35E-04 | 8.80E-01 |
| aminoglycan biosynthetic process (GO:0006023) | 5 | 1 | 1.93E-02 | 1.00E+00 |
| cellular divalent inorganic cation homeostasis (GO:0072503) | 5 | 1 | 1.93E-02 | 1.00E+00 |
| response to light stimulus (GO:0009416) | 6 | 1 | 2.25E-02 | 1.00E+00 |
| cellular polysaccharide biosynthetic process (GO:0033692) | 6 | 1 | 2.25E-02 | 1.00E+00 |
| protein O-linked glycosylation (GO:0006493) | 7 | 1 | 2.56E-02 | 1.00E+00 |
| response to calcium ion (GO:0051592) | 7 | 1 | 2.56E-02 | 1.00E+00 |
| membrane fission (GO:0090148) | 7 | 1 | 2.56E-02 | 1.00E+00 |
| response to inorganic substance (GO:0010035) | 14 | 2 | 1.16E-03 | 9.51E-01 |
| nuclear transport (GO:0051169) | 8 | 1 | 2.88E-02 | 1.00E+00 |
| regulation of mitotic nuclear division (GO:0007088) | 9 | 1 | 3.19E-02 | 1.00E+00 |
| divalent metal ion transport (GO:0070838) | 10 | 1 | 3.51E-02 | 1.00E+00 |
| divalent inorganic cation transport (GO:0072511) | 10 | 1 | 3.51E-02 | 1.00E+00 |
| response to radiation (GO:0009314) | 12 | 1 | 4.13E-02 | 1.00E+00 |
| maintenance of location (GO:0051235) | 12 | 1 | 4.13E-02 | 1.00E+00 |
| cellular metal ion homeostasis (GO:0006875) | 12 | 1 | 4.13E-02 | 1.00E+00 |
| extracellular matrix organization (GO:0030198) | 13 | 1 | 4.44E-02 | 1.00E+00 |
| negative regulation of mitotic cell cycle (GO:0045930) | 13 | 1 | 4.44E-02 | 1.00E+00 |
| response to abiotic stimulus (GO:0009628) | 13 | 1 | 4.44E-02 | 1.00E+00 |
| transition metal ion homeostasis (GO:0055076) | 13 | 1 | 4.44E-02 | 1.00E+00 |
| response to external stimulus (GO:0009605) | 14 | 1 | 4.75E-02 | 1.00E+00 |
| extracellular structure organization (GO:0043062) | 14 | 1 | 4.75E-02 | 1.00E+00 |
| transition metal ion transport (GO:0000041) | 14 | 1 | 4.75E-02 | 1.00E+00 |
| cellular component assembly (GO:0022607) | 16 | 1 | 5.37E-02 | 1.00E+00 |
| positive regulation of cell cycle (GO:0045787) | 17 | 1 | 5.68E-02 | 1.00E+00 |
| negative regulation of cell cycle (GO:0045786) | 17 | 1 | 5.68E-02 | 1.00E+00 |
| metal ion transport (GO:0030001) | 17 | 1 | 5.68E-02 | 1.00E+00 |
| macromolecule biosynthetic process (GO:0009059) | 18 | 1 | 5.98E-02 | 1.00E+00 |
| metal ion homeostasis (GO:0055065) | 20 | 1 | 6.59E-02 | 1.00E+00 |
| cell cycle process (GO:0022402) | 20 | 1 | 6.59E-02 | 1.00E+00 |
| mitotic cell cycle phase transition (GO:0044772) | 20 | 1 | 6.59E-02 | 1.00E+00 |
| cytokinesis (GO:0000910) | 20 | 1 | 6.59E-02 | 1.00E+00 |
| ion transmembrane transport (GO:0034220) | 21 | 1 | 6.90E-02 | 1.00E+00 |
| inorganic cation transmembrane transport (GO:0098662) | 21 | 1 | 6.90E-02 | 1.00E+00 |
| inorganic ion transmembrane transport (GO:0098660) | 21 | 1 | 6.90E-02 | 1.00E+00 |
| cell division (GO:0051301) | 22 | 1 | 7.20E-02 | 1.00E+00 |
| regulation of cell cycle process (GO:0010564) | 23 | 1 | 7.50E-02 | 1.00E+00 |
| cellular amino acid catabolic process (GO:0009063) | 27 | 1 | 8.70E-02 | 1.00E+00 |
| tRNA metabolic process (GO:0006399) | 30 | 1 | 9.59E-02 | 1.00E+00 |
| cation transport (GO:0006812) | 31 | 1 | 9.88E-02 | 1.00E+00 |
| carboxylic acid biosynthetic process (GO:0046394) | 35 | 1 | 1.11E-01 | 1.00E+00 |
| organic acid biosynthetic process (GO:0016053) | 35 | 1 | 1.11E-01 | 1.00E+00 |
| intracellular transport (GO:0046907) | 36 | 1 | 1.13E-01 | 1.00E+00 |
| negative regulation of cellular process (GO:0048523) | 37 | 1 | 1.16E-01 | 1.00E+00 |
| protein glycosylation (GO:0006486) | 39 | 1 | 1.22E-01 | 1.00E+00 |
| regulation of cell cycle (GO:0051726) | 39 | 1 | 1.22E-01 | 1.00E+00 |
| lipid metabolic process (GO:0006629) | 39 | 1 | 1.22E-01 | 1.00E+00 |
| divalent inorganic cation homeostasis (GO:0072507) | 39 | 1 | 1.22E-01 | 1.00E+00 |
| cellular response to chemical stimulus (GO:0070887) | 40 | 1 | 1.25E-01 | 1.00E+00 |
| glycoprotein biosynthetic process (GO:0009101) | 43 | 1 | 1.33E-01 | 1.00E+00 |
| positive regulation of cellular process (GO:0048522) | 45 | 1 | 1.39E-01 | 1.00E+00 |
| microtubule cytoskeleton organization (GO:0000226) | 47 | 1 | 1.45E-01 | 1.00E+00 |
| ion transport (GO:0006811) | 48 | 1 | 1.47E-01 | 1.00E+00 |
| carboxylic acid catabolic process (GO:0046395) | 49 | 1 | 1.50E-01 | 1.00E+00 |
| organic acid catabolic process (GO:0016054) | 49 | 1 | 1.50E-01 | 1.00E+00 |
| transmembrane receptor protein tyrosine kinase signaling pathway (GO:0007169) | 52 | 1 | 1.59E-01 | 1.00E+00 |
| carbohydrate derivative biosynthetic process (GO:1901137) | 53 | 1 | 1.61E-01 | 1.00E+00 |
| cellular macromolecule biosynthetic process (GO:0034645) | 53 | 1 | 1.61E-01 | 1.00E+00 |
| mRNA splicing, via spliceosome (GO:0000398) | 55 | 1 | 1.67E-01 | 1.00E+00 |
| RNA splicing, via transesterification reactions with bulged adenosine as nucleophile (GO:0000377) | 55 | 1 | 1.67E-01 | 1.00E+00 |
| RNA splicing, via transesterification reactions (GO:0000375) | 56 | 1 | 1.70E-01 | 1.00E+00 |
| response to stimulus (GO:0050896) | 230 | 4 | 5.87E-03 | 1.00E+00 |
| RNA splicing (GO:0008380) | 58 | 1 | 1.75E-01 | 1.00E+00 |
| inorganic ion homeostasis (GO:0098771) | 63 | 1 | 1.88E-01 | 1.00E+00 |
| enzyme linked receptor protein signaling pathway (GO:0007167) | 65 | 1 | 1.94E-01 | 1.00E+00 |
| regulation of localization (GO:0032879) | 65 | 1 | 1.94E-01 | 1.00E+00 |
| response to stress (GO:0006950) | 65 | 1 | 1.94E-01 | 1.00E+00 |
| ncRNA metabolic process (GO:0034660) | 65 | 1 | 1.94E-01 | 1.00E+00 |
| ion homeostasis (GO:0050801) | 66 | 1 | 1.96E-01 | 1.00E+00 |
| response to chemical (GO:0042221) | 134 | 2 | 7.04E-02 | 1.00E+00 |
| apoptotic process (GO:0006915) | 69 | 1 | 2.04E-01 | 1.00E+00 |
| programmed cell death (GO:0012501) | 70 | 1 | 2.07E-01 | 1.00E+00 |
| microtubule-based process (GO:0007017) | 70 | 1 | 2.07E-01 | 1.00E+00 |
| cell death (GO:0008219) | 74 | 1 | 2.17E-01 | 1.00E+00 |
| positive regulation of biological process (GO:0048518) | 75 | 1 | 2.20E-01 | 1.00E+00 |
| mitotic nuclear division (GO:0140014) | 78 | 1 | 2.27E-01 | 1.00E+00 |
| membrane organization (GO:0061024) | 78 | 1 | 2.27E-01 | 1.00E+00 |
| RNA processing (GO:0006396) | 79 | 1 | 2.30E-01 | 1.00E+00 |
| organic substance catabolic process (GO:1901575) | 80 | 1 | 2.32E-01 | 1.00E+00 |
| mitotic cell cycle (GO:0000278) | 81 | 1 | 2.35E-01 | 1.00E+00 |
| mitotic cell cycle process (GO:1903047) | 81 | 1 | 2.35E-01 | 1.00E+00 |
| cellular component organization or biogenesis (GO:0071840) | 81 | 1 | 2.35E-01 | 1.00E+00 |
| cellular component biogenesis (GO:0044085) | 81 | 1 | 2.35E-01 | 1.00E+00 |
| chemical homeostasis (GO:0048878) | 82 | 1 | 2.37E-01 | 1.00E+00 |
| cellular biosynthetic process (GO:0044249) | 84 | 1 | 2.43E-01 | 1.00E+00 |
| protein metabolic process (GO:0019538) | 88 | 1 | 2.52E-01 | 1.00E+00 |
| carbohydrate derivative metabolic process (GO:1901135) | 93 | 1 | 2.65E-01 | 1.00E+00 |
| cell cycle (GO:0007049) | 188 | 2 | 1.24E-01 | 1.00E+00 |
| catabolic process (GO:0009056) | 96 | 1 | 2.72E-01 | 1.00E+00 |
| biosynthetic process (GO:0009058) | 101 | 1 | 2.84E-01 | 1.00E+00 |
| homeostatic process (GO:0042592) | 101 | 1 | 2.84E-01 | 1.00E+00 |
| organic substance biosynthetic process (GO:1901576) | 111 | 1 | 3.07E-01 | 1.00E+00 |
| nervous system process (GO:0050877) | 112 | 1 | 3.09E-01 | 1.00E+00 |
| RNA metabolic process (GO:0016070) | 117 | 1 | 3.21E-01 | 1.00E+00 |
| system process (GO:0003008) | 137 | 1 | 3.64E-01 | 1.00E+00 |
| regulation of cellular process (GO:0050794) | 275 | 2 | 2.25E-01 | 1.00E+00 |
| signal transduction (GO:0007165) | 438 | 3 | 1.67E-01 | 1.00E+00 |
| transcription by RNA polymerase II (GO:0006366) | 292 | 2 | 2.46E-01 | 1.00E+00 |
| primary metabolic process (GO:0044238) | 151 | 1 | 3.93E-01 | 1.00E+00 |
| nucleic acid metabolic process (GO:0090304) | 169 | 1 | 4.29E-01 | 1.00E+00 |
| establishment of localization (GO:0051234) | 346 | 2 | 3.12E-01 | 1.00E+00 |
| transport (GO:0006810) | 346 | 2 | 3.12E-01 | 1.00E+00 |
| cell surface receptor signaling pathway (GO:0007166) | 176 | 1 | 4.42E-01 | 1.00E+00 |
| transcription, DNA-templated (GO:0006351) | 359 | 2 | 3.28E-01 | 1.00E+00 |
| cellular response to stimulus (GO:0051716) | 559 | 3 | 4.18E-01 | 1.00E+00 |
| regulation of biological quality (GO:0065008) | 195 | 1 | 4.76E-01 | 1.00E+00 |
| macromolecule metabolic process (GO:0043170) | 818 | 4 | 3.26E-01 | 1.00E+00 |
| regulation of transcription by RNA polymerase II (GO:0006357) | 207 | 1 | 4.97E-01 | 1.00E+00 |
| intracellular protein transport (GO:0006886) | 208 | 1 | 4.99E-01 | 1.00E+00 |
| regulation of transcription, DNA-templated (GO:0006355) | 219 | 1 | 5.17E-01 | 1.00E+00 |
| regulation of nucleic acid-templated transcription (GO:1903506) | 219 | 1 | 5.17E-01 | 1.00E+00 |
| metabolic process (GO:0008152) | 1369 | 6 | 4.14E-01 | 1.00E+00 |
| organic substance metabolic process (GO:0071704) | 1145 | 5 | 3.97E-01 | 1.00E+00 |
| regulation of RNA biosynthetic process (GO:2001141) | 233 | 1 | 5.39E-01 | 1.00E+00 |
| cellular component organization (GO:0016043) | 474 | 2 | 6.63E-01 | 1.00E+00 |
| regulation of biosynthetic process (GO:0009889) | 238 | 1 | 5.47E-01 | 1.00E+00 |
| regulation of cellular biosynthetic process (GO:0031326) | 238 | 1 | 5.47E-01 | 1.00E+00 |
| regulation of biological process (GO:0050789) | 755 | 3 | 7.27E-01 | 1.00E+00 |
| cellular protein localization (GO:0034613) | 256 | 1 | 5.74E-01 | 1.00E+00 |
| cellular macromolecule localization (GO:0070727) | 258 | 1 | 5.77E-01 | 1.00E+00 |
| gene expression (GO:0010467) | 581 | 2 | 7.13E-01 | 1.00E+00 |
| multicellular organismal process (GO:0032501) | 291 | 1 | 1.00E+00 | 1.00E+00 |
| cellular process (GO:0009987) | 1754 | 6 | 1.00E+00 | 1.00E+00 |
| biological regulation (GO:0065007) | 881 | 3 | 1.00E+00 | 1.00E+00 |
| cellular localization (GO:0051641) | 294 | 1 | 1.00E+00 | 1.00E+00 |
| localization (GO:0051179) | 597 | 2 | 1.00E+00 | 1.00E+00 |
| Unclassified (UNCLASSIFIED) | 2556 | 7 | 6.39E-01 | 1.00E+00 |
| regulation of metabolic process (GO:0019222) | 432 | 1 | 1.00E+00 | 1.00E+00 |

Table S34. GO analysis in Sphenisciformes in the 4-times hypothesis using PANTHER GO-Slim Cellular Component. P values are calculated by Fisher’s exact test and FDR values are calculated by the Benjamini–Hochberg procedure.

| GO term | Number of genes in reference | Number of genes in query | P-values | FDR values |
| --- | --- | --- | --- | --- |
| photoreceptor outer segment (GO:0001750) | 3 | 1 | 1.29E-02 | 1.00E+00 |
| mitotic spindle (GO:0072686) | 3 | 1 | 1.29E-02 | 1.00E+00 |
| spindle pole (GO:0000922) | 7 | 1 | 2.56E-02 | 1.00E+00 |
| spindle (GO:0005819) | 17 | 1 | 5.68E-02 | 1.00E+00 |
| centrosome (GO:0005813) | 39 | 1 | 1.22E-01 | 1.00E+00 |
| microtubule organizing center part (GO:0044450) | 47 | 1 | 1.45E-01 | 1.00E+00 |
| centriole (GO:0005814) | 47 | 1 | 1.45E-01 | 1.00E+00 |
| intraciliary transport particle (GO:0030990) | 52 | 1 | 1.59E-01 | 1.00E+00 |
| neuron projection (GO:0043005) | 54 | 1 | 1.64E-01 | 1.00E+00 |
| microtubule organizing center (GO:0005815) | 57 | 1 | 1.72E-01 | 1.00E+00 |
| nucleolus (GO:0005730) | 58 | 1 | 1.75E-01 | 1.00E+00 |
| microtubule cytoskeleton (GO:0015630) | 105 | 1 | 2.93E-01 | 1.00E+00 |
| integral component of plasma membrane (GO:0005887) | 213 | 2 | 1.52E-01 | 1.00E+00 |
| neuron part (GO:0097458) | 110 | 1 | 3.05E-01 | 1.00E+00 |
| plasma membrane region (GO:0098590) | 128 | 1 | 3.45E-01 | 1.00E+00 |
| integral component of membrane (GO:0016021) | 268 | 2 | 2.17E-01 | 1.00E+00 |
| intrinsic component of membrane (GO:0031224) | 269 | 2 | 2.18E-01 | 1.00E+00 |
| plasma membrane part (GO:0044459) | 149 | 1 | 3.89E-01 | 1.00E+00 |
| membrane part (GO:0044425) | 314 | 2 | 2.73E-01 | 1.00E+00 |
| membrane (GO:0016020) | 318 | 2 | 2.78E-01 | 1.00E+00 |
| cytoskeleton (GO:0005856) | 178 | 1 | 4.46E-01 | 1.00E+00 |
| nuclear lumen (GO:0031981) | 186 | 1 | 4.60E-01 | 1.00E+00 |
| plasma membrane (GO:0005886) | 623 | 3 | 4.47E-01 | 1.00E+00 |
| cell periphery (GO:0071944) | 635 | 3 | 4.53E-01 | 1.00E+00 |
| Unclassified (UNCLASSIFIED) | 2890 | 12 | 2.45E-01 | 1.00E+00 |
| nuclear part (GO:0044428) | 303 | 1 | 1.00E+00 | 1.00E+00 |
| intracellular non-membrane-bounded organelle (GO:0043232) | 342 | 1 | 1.00E+00 | 1.00E+00 |
| non-membrane-bounded organelle (GO:0043228) | 342 | 1 | 1.00E+00 | 1.00E+00 |
| nucleus (GO:0005634) | 696 | 2 | 1.00E+00 | 1.00E+00 |
| intracellular membrane-bounded organelle (GO:0043231) | 716 | 2 | 1.00E+00 | 1.00E+00 |
| intracellular organelle (GO:0043229) | 717 | 2 | 1.00E+00 | 1.00E+00 |
| protein-containing complex (GO:0032991) | 501 | 1 | 1.00E+00 | 1.00E+00 |
| cell part (GO:0044464) | 1620 | 3 | 3.06E-01 | 1.00E+00 |
| cell (GO:0005623) | 1627 | 3 | 3.06E-01 | 1.00E+00 |
| organelle (GO:0043226) | 1177 | 2 | 3.95E-01 | 1.00E+00 |

Table S35. GO analysis in Sphenisciformes in the 4-times hypothesis using PANTHER GO-Slim Molecular Function. P values are calculated by Fisher’s exact test and FDR values are calculated by the Benjamini–Hochberg procedure.

| GO term | Number of genes in reference | Number of genes in query | P-values | FDR values |
| --- | --- | --- | --- | --- |
| receptor tyrosine kinase binding (GO:0030971) | 2 | 1 | 9.68E-03 | 1.00E+00 |
| galactosyltransferase activity (GO:0008378) | 3 | 1 | 1.29E-02 | 1.00E+00 |
| UDP-glycosyltransferase activity (GO:0008194) | 7 | 1 | 2.56E-02 | 1.00E+00 |
| protein serine/threonine phosphatase activity (GO:0004722) | 15 | 1 | 5.06E-02 | 1.00E+00 |
| transferase activity, transferring hexosyl groups (GO:0016758) | 32 | 2 | 5.22E-03 | 1.00E+00 |
| calcium-dependent phospholipid binding (GO:0005544) | 18 | 1 | 5.98E-02 | 1.00E+00 |
| transferase activity, transferring glycosyl groups (GO:0016757) | 58 | 2 | 1.57E-02 | 1.00E+00 |
| phosphoprotein phosphatase activity (GO:0004721) | 38 | 1 | 1.19E-01 | 1.00E+00 |
| phospholipid binding (GO:0005543) | 55 | 1 | 1.67E-01 | 1.00E+00 |
| metal ion transmembrane transporter activity (GO:0046873) | 56 | 1 | 1.70E-01 | 1.00E+00 |
| mRNA binding (GO:0003729) | 56 | 1 | 1.70E-01 | 1.00E+00 |
| catalytic activity, acting on a protein (GO:0140096) | 60 | 1 | 1.80E-01 | 1.00E+00 |
| phosphatase activity (GO:0016791) | 66 | 1 | 1.96E-01 | 1.00E+00 |
| anion binding (GO:0043168) | 91 | 1 | 2.60E-01 | 1.00E+00 |
| signaling receptor binding (GO:0005102) | 94 | 1 | 2.67E-01 | 1.00E+00 |
| RNA binding (GO:0003723) | 195 | 2 | 1.32E-01 | 1.00E+00 |
| phosphoric ester hydrolase activity (GO:0042578) | 101 | 1 | 2.84E-01 | 1.00E+00 |
| G-protein coupled receptor activity (GO:0004930) | 107 | 1 | 2.98E-01 | 1.00E+00 |
| inorganic cation transmembrane transporter activity (GO:0022890) | 109 | 1 | 3.02E-01 | 1.00E+00 |
| cation transmembrane transporter activity (GO:0008324) | 119 | 1 | 3.25E-01 | 1.00E+00 |
| transmembrane signaling receptor activity (GO:0004888) | 157 | 1 | 4.05E-01 | 1.00E+00 |
| ion transmembrane transporter activity (GO:0015075) | 158 | 1 | 4.07E-01 | 1.00E+00 |
| protein kinase activity (GO:0004672) | 162 | 1 | 4.15E-01 | 1.00E+00 |
| ion binding (GO:0043167) | 166 | 1 | 4.23E-01 | 1.00E+00 |
| hydrolase activity, acting on ester bonds (GO:0016788) | 174 | 1 | 4.38E-01 | 1.00E+00 |
| nucleic acid binding (GO:0003676) | 532 | 3 | 2.47E-01 | 1.00E+00 |
| heterocyclic compound binding (GO:1901363) | 556 | 3 | 4.17E-01 | 1.00E+00 |
| transferase activity (GO:0016740) | 590 | 3 | 4.31E-01 | 1.00E+00 |
| phosphotransferase activity, alcohol group as acceptor (GO:0016773) | 199 | 1 | 4.83E-01 | 1.00E+00 |
| signaling receptor activity (GO:0038023) | 201 | 1 | 4.87E-01 | 1.00E+00 |
| DNA-binding transcription factor activity (GO:0003700) | 212 | 1 | 5.06E-01 | 1.00E+00 |
| molecular transducer activity (GO:0060089) | 225 | 1 | 5.27E-01 | 1.00E+00 |
| transmembrane transporter activity (GO:0022857) | 238 | 1 | 5.47E-01 | 1.00E+00 |
| transcription regulator activity (GO:0140110) | 257 | 1 | 5.75E-01 | 1.00E+00 |
| transferase activity, transferring phosphorus-containing groups (GO:0016772) | 266 | 1 | 5.88E-01 | 1.00E+00 |
| transporter activity (GO:0005215) | 292 | 1 | 1.00E+00 | 1.00E+00 |
| catalytic activity (GO:0003824) | 1504 | 5 | 1.00E+00 | 1.00E+00 |
| DNA binding (GO:0003677) | 302 | 1 | 1.00E+00 | 1.00E+00 |
| Unclassified (UNCLASSIFIED) | 2686 | 8 | 8.16E-01 | 1.00E+00 |
| protein binding (GO:0005515) | 690 | 2 | 1.00E+00 | 1.00E+00 |
| binding (GO:0005488) | 1436 | 4 | 1.00E+00 | 1.00E+00 |
| hydrolase activity (GO:0016787) | 607 | 1 | 7.13E-01 | 1.00E+00 |

Table S36. Pathway analysis in Sphenisciformes in the 4-times hypothesis using PANTHER Pathways. P values are calculated by Fisher’s exact test and FDR values are calculated by the Benjamini–Hochberg procedure.

| GO term | Number of genes in reference | Number of genes in query | P-values | FDR values |
| --- | --- | --- | --- | --- |
| Unclassified (UNCLASSIFIED) | 4758 | 18 | 9.63E-02 | 1.00E+00 |

Table S37. GO analysis in Struthioniformes in the 4-times hypothesis using PANTHER GO-Slim Biological Process. P values are calculated by Fisher’s exact test and FDR values are calculated by the Benjamini–Hochberg procedure.

| GO term | Number of genes in reference | Number of genes in query | P-values | FDR values |
| --- | --- | --- | --- | --- |
| protein demethylation (GO:0006482) | 1 | 1 | 2.17E-02 | 1.00E+00 |
| interleukin-6 production (GO:0032635) | 1 | 1 | 2.17E-02 | 1.00E+00 |
| regulation of cytokine biosynthetic process (GO:0042035) | 1 | 1 | 2.17E-02 | 1.00E+00 |
| protein dealkylation (GO:0008214) | 1 | 1 | 2.17E-02 | 1.00E+00 |
| positive regulation of protein metabolic process (GO:0051247) | 1 | 1 | 2.17E-02 | 1.00E+00 |
| defense response to virus (GO:0051607) | 2 | 1 | 3.23E-02 | 1.00E+00 |
| establishment or maintenance of epithelial cell apical/basal polarity (GO:0045197) | 2 | 1 | 3.23E-02 | 1.00E+00 |
| cation homeostasis (GO:0055080) | 5 | 2 | 2.36E-03 | 1.00E+00 |
| cellular monovalent inorganic cation homeostasis (GO:0030004) | 5 | 2 | 2.36E-03 | 1.00E+00 |
| cellular cation homeostasis (GO:0030003) | 5 | 2 | 2.36E-03 | 1.00E+00 |
| centrosome duplication (GO:0051298) | 5 | 2 | 2.36E-03 | 9.72E-01 |
| glycerolipid catabolic process (GO:0046503) | 3 | 1 | 4.29E-02 | 1.00E+00 |
| regulation of translational initiation (GO:0006446) | 3 | 1 | 4.29E-02 | 1.00E+00 |
| protein-containing complex localization (GO:0031503) | 3 | 1 | 4.29E-02 | 1.00E+00 |
| positive regulation of canonical Wnt signaling pathway (GO:0090263) | 3 | 1 | 4.29E-02 | 1.00E+00 |
| protein localization to synapse (GO:0035418) | 3 | 1 | 4.29E-02 | 1.00E+00 |
| receptor localization to synapse (GO:0097120) | 3 | 1 | 4.29E-02 | 1.00E+00 |
| T cell activation (GO:0042110) | 4 | 1 | 5.33E-02 | 1.00E+00 |
| synapsis (GO:0007129) | 4 | 1 | 5.33E-02 | 1.00E+00 |
| toll-like receptor signaling pathway (GO:0002224) | 4 | 1 | 5.33E-02 | 1.00E+00 |
| protein K11-linked ubiquitination (GO:0070979) | 4 | 1 | 5.33E-02 | 1.00E+00 |
| positive regulation of NF-kappaB transcription factor activity (GO:0051092) | 9 | 2 | 6.02E-03 | 1.00E+00 |
| centrosome cycle (GO:0007098) | 9 | 2 | 6.02E-03 | 1.00E+00 |
| positive regulation of Wnt signaling pathway (GO:0030177) | 5 | 1 | 6.36E-02 | 1.00E+00 |
| integrin-mediated signaling pathway (GO:0007229) | 5 | 1 | 6.36E-02 | 1.00E+00 |
| positive regulation of I-kappaB kinase/NF-kappaB signaling (GO:0043123) | 5 | 1 | 6.36E-02 | 1.00E+00 |
| regulation of I-kappaB kinase/NF-kappaB signaling (GO:0043122) | 5 | 1 | 6.36E-02 | 1.00E+00 |
| cytokine production (GO:0001816) | 5 | 1 | 6.36E-02 | 1.00E+00 |
| activation of immune response (GO:0002253) | 5 | 1 | 6.36E-02 | 1.00E+00 |
| immune effector process (GO:0002252) | 5 | 1 | 6.36E-02 | 1.00E+00 |
| positive regulation of ERK1 and ERK2 cascade (GO:0070374) | 5 | 1 | 6.36E-02 | 1.00E+00 |
| activation of innate immune response (GO:0002218) | 5 | 1 | 6.36E-02 | 1.00E+00 |
| pyrimidine nucleobase metabolic process (GO:0006206) | 5 | 1 | 6.36E-02 | 1.00E+00 |
| positive regulation of cellular biosynthetic process (GO:0031328) | 5 | 1 | 6.36E-02 | 1.00E+00 |
| homologous chromosome segregation (GO:0045143) | 5 | 1 | 6.36E-02 | 1.00E+00 |
| cytokine-mediated signaling pathway (GO:0019221) | 11 | 2 | 8.42E-03 | 1.00E+00 |
| nucleobase metabolic process (GO:0009112) | 6 | 1 | 7.38E-02 | 1.00E+00 |
| positive regulation of DNA-binding transcription factor activity (GO:0051091) | 12 | 2 | 9.75E-03 | 1.00E+00 |
| organophosphate catabolic process (GO:0046434) | 6 | 1 | 7.38E-02 | 1.00E+00 |
| negative regulation of cell adhesion (GO:0007162) | 6 | 1 | 7.38E-02 | 1.00E+00 |
| mitotic sister chromatid separation (GO:0051306) | 6 | 1 | 7.38E-02 | 1.00E+00 |
| transcription by RNA polymerase III (GO:0006383) | 6 | 1 | 7.38E-02 | 1.00E+00 |
| cell adhesion mediated by integrin (GO:0033627) | 6 | 1 | 7.38E-02 | 1.00E+00 |
| lymphocyte activation (GO:0046649) | 6 | 1 | 7.38E-02 | 1.00E+00 |
| metaphase/anaphase transition of mitotic cell cycle (GO:0007091) | 6 | 1 | 7.38E-02 | 1.00E+00 |
| cellular metal ion homeostasis (GO:0006875) | 12 | 2 | 9.75E-03 | 1.00E+00 |
| cellular response to cytokine stimulus (GO:0071345) | 12 | 2 | 9.75E-03 | 1.00E+00 |
| histone modification (GO:0016570) | 7 | 1 | 8.39E-02 | 1.00E+00 |
| innate immune response (GO:0045087) | 7 | 1 | 8.39E-02 | 1.00E+00 |
| cellular nitrogen compound catabolic process (GO:0044270) | 7 | 1 | 8.39E-02 | 1.00E+00 |
| leukocyte activation (GO:0045321) | 7 | 1 | 8.39E-02 | 1.00E+00 |
| positive regulation of axonogenesis (GO:0050772) | 7 | 1 | 8.39E-02 | 1.00E+00 |
| I-kappaB kinase/NF-kappaB signaling (GO:0007249) | 15 | 2 | 1.43E-02 | 1.00E+00 |
| nuclear transport (GO:0051169) | 8 | 1 | 9.39E-02 | 1.00E+00 |
| regulation of cell adhesion (GO:0030155) | 8 | 1 | 9.39E-02 | 1.00E+00 |
| microtubule polymerization (GO:0046785) | 8 | 1 | 9.39E-02 | 1.00E+00 |
| wound healing (GO:0042060) | 8 | 1 | 9.39E-02 | 1.00E+00 |
| positive regulation of macromolecule biosynthetic process (GO:0010557) | 8 | 1 | 9.39E-02 | 1.00E+00 |
| heterocycle catabolic process (GO:0046700) | 8 | 1 | 9.39E-02 | 1.00E+00 |
| response to wounding (GO:0009611) | 8 | 1 | 9.39E-02 | 1.00E+00 |
| spindle organization (GO:0007051) | 8 | 1 | 9.39E-02 | 1.00E+00 |
| microtubule nucleation (GO:0007020) | 8 | 1 | 9.39E-02 | 1.00E+00 |
| establishment or maintenance of cell polarity (GO:0007163) | 17 | 2 | 1.77E-02 | 1.00E+00 |
| positive regulation of MAPK cascade (GO:0043410) | 9 | 1 | 1.04E-01 | 1.00E+00 |
| organic cyclic compound catabolic process (GO:1901361) | 9 | 1 | 1.04E-01 | 1.00E+00 |
| regulation of GTPase activity (GO:0043087) | 9 | 1 | 1.04E-01 | 1.00E+00 |
| protein polymerization (GO:0051258) | 9 | 1 | 1.04E-01 | 1.00E+00 |
| regulation of mitotic nuclear division (GO:0007088) | 9 | 1 | 1.04E-01 | 1.00E+00 |
| epithelial cell differentiation (GO:0030855) | 9 | 1 | 1.04E-01 | 1.00E+00 |
| regulation of translation (GO:0006417) | 19 | 2 | 2.14E-02 | 1.00E+00 |
| regulation of cellular amide metabolic process (GO:0034248) | 20 | 2 | 2.34E-02 | 1.00E+00 |
| positive regulation of neuron projection development (GO:0010976) | 10 | 1 | 1.14E-01 | 1.00E+00 |
| metal ion homeostasis (GO:0055065) | 20 | 2 | 2.34E-02 | 1.00E+00 |
| cell cycle process (GO:0022402) | 20 | 2 | 2.34E-02 | 1.00E+00 |
| response to cytokine (GO:0034097) | 20 | 2 | 2.34E-02 | 1.00E+00 |
| positive regulation of macromolecule metabolic process (GO:0010604) | 11 | 1 | 1.23E-01 | 1.00E+00 |
| SCF-dependent proteasomal ubiquitin-dependent protein catabolic process (GO:0031146) | 11 | 1 | 1.23E-01 | 1.00E+00 |
| import into cell (GO:0098657) | 22 | 2 | 2.76E-02 | 1.00E+00 |
| positive regulation of biosynthetic process (GO:0009891) | 11 | 1 | 1.23E-01 | 1.00E+00 |
| regulation of cell shape (GO:0008360) | 12 | 1 | 1.33E-01 | 1.00E+00 |
| protein localization to membrane (GO:0072657) | 12 | 1 | 1.33E-01 | 1.00E+00 |
| proton transmembrane transport (GO:1902600) | 12 | 1 | 1.33E-01 | 1.00E+00 |
| positive regulation of metabolic process (GO:0009893) | 13 | 1 | 1.42E-01 | 1.00E+00 |
| cellular lipid catabolic process (GO:0044242) | 14 | 1 | 1.52E-01 | 1.00E+00 |
| positive regulation of neuron differentiation (GO:0045666) | 14 | 1 | 1.52E-01 | 1.00E+00 |
| protein N-linked glycosylation (GO:0006487) | 15 | 1 | 1.61E-01 | 1.00E+00 |
| heterocycle metabolic process (GO:0046483) | 15 | 1 | 1.61E-01 | 1.00E+00 |
| meiosis I cell cycle phase (GO:0098764) | 15 | 1 | 1.61E-01 | 1.00E+00 |
| meiotic cell cycle phase (GO:0098762) | 15 | 1 | 1.61E-01 | 1.00E+00 |
| meiotic telophase I (GO:0007134) | 15 | 1 | 1.61E-01 | 1.00E+00 |
| biological phase (GO:0044848) | 15 | 1 | 1.61E-01 | 1.00E+00 |
| meiosis I (GO:0007127) | 15 | 1 | 1.61E-01 | 1.00E+00 |
| cell cycle phase (GO:0022403) | 15 | 1 | 1.61E-01 | 1.00E+00 |
| positive regulation of neurogenesis (GO:0050769) | 15 | 1 | 1.61E-01 | 1.00E+00 |
| canonical Wnt signaling pathway (GO:0060070) | 16 | 1 | 1.70E-01 | 1.00E+00 |
| regulation of hydrolase activity (GO:0051336) | 16 | 1 | 1.70E-01 | 1.00E+00 |
| regulation of MAPK cascade (GO:0043408) | 16 | 1 | 1.70E-01 | 1.00E+00 |
| regulation of cyclin-dependent protein serine/threonine kinase activity (GO:0000079) | 16 | 1 | 1.70E-01 | 1.00E+00 |
| positive regulation of multicellular organismal process (GO:0051240) | 16 | 1 | 1.70E-01 | 1.00E+00 |
| meiosis I cell cycle process (GO:0061982) | 16 | 1 | 1.70E-01 | 1.00E+00 |
| positive regulation of molecular function (GO:0044093) | 32 | 2 | 5.23E-02 | 1.00E+00 |
| positive regulation of cell cycle (GO:0045787) | 17 | 1 | 1.79E-01 | 1.00E+00 |
| epithelium development (GO:0060429) | 17 | 1 | 1.79E-01 | 1.00E+00 |
| mitotic sister chromatid segregation (GO:0000070) | 17 | 1 | 1.79E-01 | 1.00E+00 |
| movement of cell or subcellular component (GO:0006928) | 17 | 1 | 1.79E-01 | 1.00E+00 |
| stress-activated protein kinase signaling cascade (GO:0031098) | 17 | 1 | 1.79E-01 | 1.00E+00 |
| positive regulation of cell development (GO:0010720) | 17 | 1 | 1.79E-01 | 1.00E+00 |
| axon guidance (GO:0007411) | 18 | 1 | 1.88E-01 | 1.00E+00 |
| peptidyl-amino acid modification (GO:0018193) | 55 | 3 | 2.48E-02 | 1.00E+00 |
| positive regulation of cell proliferation (GO:0008284) | 19 | 1 | 1.97E-01 | 1.00E+00 |
| positive regulation of cell differentiation (GO:0045597) | 19 | 1 | 1.97E-01 | 1.00E+00 |
| immune system process (GO:0002376) | 59 | 3 | 2.95E-02 | 1.00E+00 |
| regulation of cell migration (GO:0030334) | 20 | 1 | 2.06E-01 | 1.00E+00 |
| cell-cell adhesion (GO:0098609) | 20 | 1 | 2.06E-01 | 1.00E+00 |
| positive regulation of protein kinase activity (GO:0045860) | 20 | 1 | 2.06E-01 | 1.00E+00 |
| mitotic cell cycle phase transition (GO:0044772) | 20 | 1 | 2.06E-01 | 1.00E+00 |
| Golgi organization (GO:0007030) | 20 | 1 | 2.06E-01 | 1.00E+00 |
| activation of protein kinase activity (GO:0032147) | 20 | 1 | 2.06E-01 | 1.00E+00 |
| ion transmembrane transport (GO:0034220) | 21 | 1 | 2.14E-01 | 1.00E+00 |
| cell-substrate adhesion (GO:0031589) | 21 | 1 | 2.14E-01 | 1.00E+00 |
| cell-matrix adhesion (GO:0007160) | 21 | 1 | 2.14E-01 | 1.00E+00 |
| defense response (GO:0006952) | 21 | 1 | 2.14E-01 | 1.00E+00 |
| inorganic cation transmembrane transport (GO:0098662) | 21 | 1 | 2.14E-01 | 1.00E+00 |
| inorganic ion transmembrane transport (GO:0098660) | 21 | 1 | 2.14E-01 | 1.00E+00 |
| cell migration (GO:0016477) | 44 | 2 | 8.90E-02 | 1.00E+00 |
| regulation of cell motility (GO:2000145) | 22 | 1 | 2.23E-01 | 1.00E+00 |
| negative regulation of intracellular signal transduction (GO:1902532) | 23 | 1 | 2.31E-01 | 1.00E+00 |
| regulation of cell cycle process (GO:0010564) | 23 | 1 | 2.31E-01 | 1.00E+00 |
| cell proliferation (GO:0008283) | 23 | 1 | 2.31E-01 | 1.00E+00 |
| peptidyl-lysine modification (GO:0018205) | 23 | 1 | 2.31E-01 | 1.00E+00 |
| organonitrogen compound catabolic process (GO:1901565) | 23 | 1 | 2.31E-01 | 1.00E+00 |
| regulation of cellular component movement (GO:0051270) | 24 | 1 | 2.40E-01 | 1.00E+00 |
| Wnt signaling pathway (GO:0016055) | 25 | 1 | 2.48E-01 | 1.00E+00 |
| regulation of cell proliferation (GO:0042127) | 26 | 1 | 2.56E-01 | 1.00E+00 |
| localization of cell (GO:0051674) | 52 | 2 | 1.17E-01 | 1.00E+00 |
| cell motility (GO:0048870) | 52 | 2 | 1.17E-01 | 1.00E+00 |
| cell-cell signaling by wnt (GO:0198738) | 26 | 1 | 2.56E-01 | 1.00E+00 |
| mitotic nuclear division (GO:0140014) | 78 | 3 | 5.76E-02 | 1.00E+00 |
| mitotic cell cycle (GO:0000278) | 81 | 3 | 6.29E-02 | 1.00E+00 |
| mitotic cell cycle process (GO:1903047) | 81 | 3 | 6.29E-02 | 1.00E+00 |
| protein polyubiquitination (GO:0000209) | 27 | 1 | 2.65E-01 | 1.00E+00 |
| proteasome-mediated ubiquitin-dependent protein catabolic process (GO:0043161) | 54 | 2 | 1.24E-01 | 1.00E+00 |
| protein localization (GO:0008104) | 27 | 1 | 2.65E-01 | 1.00E+00 |
| regulation of cell differentiation (GO:0045595) | 28 | 1 | 2.73E-01 | 1.00E+00 |
| axonogenesis (GO:0007409) | 29 | 1 | 2.81E-01 | 1.00E+00 |
| regulation of multicellular organismal process (GO:0051239) | 29 | 1 | 2.81E-01 | 1.00E+00 |
| protein metabolic process (GO:0019538) | 88 | 3 | 7.61E-02 | 1.00E+00 |
| cell adhesion (GO:0007155) | 88 | 3 | 7.61E-02 | 1.00E+00 |
| biological adhesion (GO:0022610) | 88 | 3 | 7.61E-02 | 1.00E+00 |
| proteasomal protein catabolic process (GO:0010498) | 59 | 2 | 1.42E-01 | 1.00E+00 |
| positive regulation of signal transduction (GO:0009967) | 30 | 1 | 2.88E-01 | 1.00E+00 |
| signal transduction by protein phosphorylation (GO:0023014) | 61 | 2 | 1.50E-01 | 1.00E+00 |
| regulation of molecular function (GO:0065009) | 92 | 3 | 8.41E-02 | 1.00E+00 |
| formation of translation initiation ternary complex (GO:0001677) | 93 | 3 | 8.62E-02 | 1.00E+00 |
| translational termination (GO:0006415) | 93 | 3 | 8.62E-02 | 1.00E+00 |
| translational elongation (GO:0006414) | 93 | 3 | 8.62E-02 | 1.00E+00 |
| meiotic cell cycle process (GO:1903046) | 31 | 1 | 2.96E-01 | 1.00E+00 |
| cellular catabolic process (GO:0044248) | 31 | 1 | 2.96E-01 | 1.00E+00 |
| meiotic cell cycle (GO:0051321) | 31 | 1 | 2.96E-01 | 1.00E+00 |
| positive regulation of response to stimulus (GO:0048584) | 31 | 1 | 2.96E-01 | 1.00E+00 |
| glycerophospholipid metabolic process (GO:0006650) | 31 | 1 | 2.96E-01 | 1.00E+00 |
| inorganic ion homeostasis (GO:0098771) | 63 | 2 | 1.57E-01 | 1.00E+00 |
| endomembrane system organization (GO:0010256) | 32 | 1 | 3.04E-01 | 1.00E+00 |
| reproductive process (GO:0022414) | 32 | 1 | 3.04E-01 | 1.00E+00 |
| response to organic substance (GO:0010033) | 64 | 2 | 1.61E-01 | 1.00E+00 |
| response to stress (GO:0006950) | 65 | 2 | 1.65E-01 | 1.00E+00 |
| positive regulation of protein phosphorylation (GO:0001934) | 33 | 1 | 3.12E-01 | 1.00E+00 |
| cell morphogenesis involved in neuron differentiation (GO:0048667) | 33 | 1 | 3.12E-01 | 1.00E+00 |
| ion homeostasis (GO:0050801) | 66 | 2 | 1.69E-01 | 1.00E+00 |
| regulation of macromolecule metabolic process (GO:0060255) | 33 | 1 | 3.12E-01 | 1.00E+00 |
| tissue development (GO:0009888) | 33 | 1 | 3.12E-01 | 1.00E+00 |
| positive regulation of phosphorylation (GO:0042327) | 34 | 1 | 3.19E-01 | 1.00E+00 |
| apoptotic process (GO:0006915) | 69 | 2 | 1.81E-01 | 1.00E+00 |
| programmed cell death (GO:0012501) | 70 | 2 | 1.84E-01 | 1.00E+00 |
| drug metabolic process (GO:0017144) | 35 | 1 | 3.27E-01 | 1.00E+00 |
| protein modification by small protein removal (GO:0070646) | 35 | 1 | 3.27E-01 | 1.00E+00 |
| translation (GO:0006412) | 108 | 3 | 1.20E-01 | 1.00E+00 |
| intracellular transport (GO:0046907) | 36 | 1 | 3.34E-01 | 1.00E+00 |
| regulation of intracellular signal transduction (GO:1902531) | 74 | 2 | 2.00E-01 | 1.00E+00 |
| cell death (GO:0008219) | 74 | 2 | 2.00E-01 | 1.00E+00 |
| positive regulation of biological process (GO:0048518) | 75 | 2 | 2.04E-01 | 1.00E+00 |
| cell cycle (GO:0007049) | 188 | 5 | 5.79E-02 | 1.00E+00 |
| RNA catabolic process (GO:0006401) | 38 | 1 | 3.48E-01 | 1.00E+00 |
| organelle assembly (GO:0070925) | 38 | 1 | 3.48E-01 | 1.00E+00 |
| regulation of developmental process (GO:0050793) | 38 | 1 | 3.48E-01 | 1.00E+00 |
| protein glycosylation (GO:0006486) | 39 | 1 | 3.56E-01 | 1.00E+00 |
| regulation of cell cycle (GO:0051726) | 39 | 1 | 3.56E-01 | 1.00E+00 |
| lipid metabolic process (GO:0006629) | 39 | 1 | 3.56E-01 | 1.00E+00 |
| cellular protein modification process (GO:0006464) | 274 | 7 | 3.18E-02 | 1.00E+00 |
| glycerolipid metabolic process (GO:0046486) | 40 | 1 | 3.63E-01 | 1.00E+00 |
| cellular protein-containing complex assembly (GO:0034622) | 40 | 1 | 3.63E-01 | 1.00E+00 |
| cellular component morphogenesis (GO:0032989) | 41 | 1 | 3.70E-01 | 1.00E+00 |
| negative regulation of signal transduction (GO:0009968) | 41 | 1 | 3.70E-01 | 1.00E+00 |
| chemical homeostasis (GO:0048878) | 82 | 2 | 2.32E-01 | 1.00E+00 |
| organonitrogen compound biosynthetic process (GO:1901566) | 42 | 1 | 3.77E-01 | 1.00E+00 |
| cellular protein metabolic process (GO:0044267) | 297 | 7 | 4.57E-02 | 1.00E+00 |
| cellular macromolecule metabolic process (GO:0044260) | 297 | 7 | 4.57E-02 | 1.00E+00 |
| glycoprotein biosynthetic process (GO:0009101) | 43 | 1 | 3.83E-01 | 1.00E+00 |
| negative regulation of response to stimulus (GO:0048585) | 44 | 1 | 3.90E-01 | 1.00E+00 |
| positive regulation of cellular process (GO:0048522) | 45 | 1 | 3.97E-01 | 1.00E+00 |
| regulation of cell communication (GO:0010646) | 95 | 2 | 2.85E-01 | 1.00E+00 |
| regulation of signal transduction (GO:0009966) | 95 | 2 | 2.85E-01 | 1.00E+00 |
| proteolysis involved in cellular protein catabolic process (GO:0051603) | 96 | 2 | 2.89E-01 | 1.00E+00 |
| ion transport (GO:0006811) | 48 | 1 | 4.17E-01 | 1.00E+00 |
| cellular protein catabolic process (GO:0044257) | 97 | 2 | 2.93E-01 | 1.00E+00 |
| cell-cell signaling (GO:0007267) | 146 | 3 | 2.20E-01 | 1.00E+00 |
| cell communication (GO:0007154) | 146 | 3 | 2.20E-01 | 1.00E+00 |
| protein phosphorylation (GO:0006468) | 98 | 2 | 2.97E-01 | 1.00E+00 |
| intracellular signal transduction (GO:0035556) | 198 | 4 | 2.82E-01 | 1.00E+00 |
| neuron development (GO:0048666) | 50 | 1 | 4.29E-01 | 1.00E+00 |
| nucleobase-containing small molecule metabolic process (GO:0055086) | 50 | 1 | 4.29E-01 | 1.00E+00 |
| organophosphate metabolic process (GO:0019637) | 50 | 1 | 4.29E-01 | 1.00E+00 |
| regulation of cellular metabolic process (GO:0031323) | 150 | 3 | 2.32E-01 | 1.00E+00 |
| primary metabolic process (GO:0044238) | 151 | 3 | 2.35E-01 | 1.00E+00 |
| homeostatic process (GO:0042592) | 101 | 2 | 3.09E-01 | 1.00E+00 |
| MAPK cascade (GO:0000165) | 51 | 1 | 4.36E-01 | 1.00E+00 |
| transmembrane receptor protein tyrosine kinase signaling pathway (GO:0007169) | 52 | 1 | 4.42E-01 | 1.00E+00 |
| carbohydrate derivative biosynthetic process (GO:1901137) | 53 | 1 | 4.48E-01 | 1.00E+00 |
| signal transduction (GO:0007165) | 438 | 8 | 1.48E-01 | 1.00E+00 |
| mRNA splicing, via spliceosome (GO:0000398) | 55 | 1 | 4.60E-01 | 1.00E+00 |
| RNA splicing, via transesterification reactions with bulged adenosine as nucleophile (GO:0000377) | 55 | 1 | 4.60E-01 | 1.00E+00 |
| protein catabolic process (GO:0030163) | 111 | 2 | 3.50E-01 | 1.00E+00 |
| cellular response to stimulus (GO:0051716) | 559 | 10 | 1.30E-01 | 1.00E+00 |
| RNA splicing, via transesterification reactions (GO:0000375) | 56 | 1 | 4.66E-01 | 1.00E+00 |
| regulation of catalytic activity (GO:0050790) | 56 | 1 | 4.66E-01 | 1.00E+00 |
| macromolecule catabolic process (GO:0009057) | 114 | 2 | 3.61E-01 | 1.00E+00 |
| macromolecule localization (GO:0033036) | 57 | 1 | 4.72E-01 | 1.00E+00 |
| response to stimulus (GO:0050896) | 230 | 4 | 3.23E-01 | 1.00E+00 |
| RNA splicing (GO:0008380) | 58 | 1 | 4.78E-01 | 1.00E+00 |
| neuron differentiation (GO:0030182) | 58 | 1 | 4.78E-01 | 1.00E+00 |
| cell surface receptor signaling pathway (GO:0007166) | 176 | 3 | 4.46E-01 | 1.00E+00 |
| regulation of phosphorylation (GO:0042325) | 60 | 1 | 4.89E-01 | 1.00E+00 |
| reproduction (GO:0000003) | 60 | 1 | 4.89E-01 | 1.00E+00 |
| cellular metabolic process (GO:0044237) | 567 | 9 | 2.84E-01 | 1.00E+00 |
| cellular process (GO:0009987) | 1754 | 27 | 3.86E-02 | 1.00E+00 |
| generation of neurons (GO:0048699) | 65 | 1 | 5.17E-01 | 1.00E+00 |
| enzyme linked receptor protein signaling pathway (GO:0007167) | 65 | 1 | 5.17E-01 | 1.00E+00 |
| regulation of localization (GO:0032879) | 65 | 1 | 5.17E-01 | 1.00E+00 |
| regulation of biological quality (GO:0065008) | 195 | 3 | 4.76E-01 | 1.00E+00 |
| proteolysis (GO:0006508) | 65 | 1 | 5.17E-01 | 1.00E+00 |
| protein-containing complex assembly (GO:0065003) | 66 | 1 | 5.22E-01 | 1.00E+00 |
| response to chemical (GO:0042221) | 134 | 2 | 6.60E-01 | 1.00E+00 |
| regulation of phosphorus metabolic process (GO:0051174) | 68 | 1 | 5.32E-01 | 1.00E+00 |
| regulation of phosphate metabolic process (GO:0019220) | 68 | 1 | 5.32E-01 | 1.00E+00 |
| regulation of response to stimulus (GO:0048583) | 68 | 1 | 5.32E-01 | 1.00E+00 |
| cellular response to stress (GO:0033554) | 143 | 2 | 6.72E-01 | 1.00E+00 |
| small molecule metabolic process (GO:0044281) | 72 | 1 | 5.53E-01 | 1.00E+00 |
| neurogenesis (GO:0022008) | 72 | 1 | 5.53E-01 | 1.00E+00 |
| protein ubiquitination (GO:0016567) | 75 | 1 | 5.67E-01 | 1.00E+00 |
| anatomical structure development (GO:0048856) | 75 | 1 | 5.67E-01 | 1.00E+00 |
| cytoskeleton organization (GO:0007010) | 75 | 1 | 5.67E-01 | 1.00E+00 |
| RNA processing (GO:0006396) | 79 | 1 | 5.86E-01 | 1.00E+00 |
| protein-containing complex subunit organization (GO:0043933) | 80 | 1 | 5.91E-01 | 1.00E+00 |
| organic substance catabolic process (GO:1901575) | 80 | 1 | 5.91E-01 | 1.00E+00 |
| protein modification by small protein conjugation or removal (GO:0070647) | 81 | 1 | 5.95E-01 | 1.00E+00 |
| protein modification by small protein conjugation (GO:0032446) | 81 | 1 | 5.95E-01 | 1.00E+00 |
| macromolecule metabolic process (GO:0043170) | 818 | 10 | 7.16E-01 | 1.00E+00 |
| gene expression (GO:0010467) | 581 | 7 | 8.32E-01 | 1.00E+00 |
| nervous system development (GO:0007399) | 88 | 1 | 6.25E-01 | 1.00E+00 |
| organic substance metabolic process (GO:0071704) | 1145 | 13 | 8.74E-01 | 1.00E+00 |
| biological regulation (GO:0065007) | 881 | 10 | 8.61E-01 | 1.00E+00 |
| transcription, DNA-templated (GO:0006351) | 359 | 4 | 1.00E+00 | 1.00E+00 |
| metabolic process (GO:0008152) | 1369 | 15 | 1.00E+00 | 1.00E+00 |
| regulation of cellular process (GO:0050794) | 275 | 3 | 1.00E+00 | 1.00E+00 |
| cellular lipid metabolic process (GO:0044255) | 92 | 1 | 1.00E+00 | 1.00E+00 |
| carbohydrate derivative metabolic process (GO:1901135) | 93 | 1 | 1.00E+00 | 1.00E+00 |
| regulation of biological process (GO:0050789) | 755 | 8 | 1.00E+00 | 1.00E+00 |
| cellular component organization (GO:0016043) | 474 | 5 | 1.00E+00 | 1.00E+00 |
| catabolic process (GO:0009056) | 96 | 1 | 1.00E+00 | 1.00E+00 |
| multicellular organismal process (GO:0032501) | 291 | 3 | 1.00E+00 | 1.00E+00 |
| chemical synaptic transmission (GO:0007268) | 98 | 1 | 1.00E+00 | 1.00E+00 |
| anterograde trans-synaptic signaling (GO:0098916) | 98 | 1 | 1.00E+00 | 1.00E+00 |
| Unclassified (UNCLASSIFIED) | 2556 | 26 | 6.08E-01 | 1.00E+00 |
| trans-synaptic signaling (GO:0099537) | 99 | 1 | 1.00E+00 | 1.00E+00 |
| synaptic signaling (GO:0099536) | 99 | 1 | 1.00E+00 | 1.00E+00 |
| localization (GO:0051179) | 597 | 6 | 1.00E+00 | 1.00E+00 |
| regulation of metabolic process (GO:0019222) | 432 | 4 | 1.00E+00 | 1.00E+00 |
| developmental process (GO:0032502) | 110 | 1 | 1.00E+00 | 1.00E+00 |
| organic substance biosynthetic process (GO:1901576) | 111 | 1 | 1.00E+00 | 1.00E+00 |
| nervous system process (GO:0050877) | 112 | 1 | 1.00E+00 | 1.00E+00 |
| establishment of localization (GO:0051234) | 346 | 3 | 1.00E+00 | 1.00E+00 |
| transport (GO:0006810) | 346 | 3 | 1.00E+00 | 1.00E+00 |
| RNA metabolic process (GO:0016070) | 117 | 1 | 1.00E+00 | 1.00E+00 |
| organelle organization (GO:0006996) | 242 | 2 | 1.00E+00 | 1.00E+00 |
| system development (GO:0048731) | 124 | 1 | 1.00E+00 | 1.00E+00 |
| cellular protein localization (GO:0034613) | 256 | 2 | 1.00E+00 | 1.00E+00 |
| cellular macromolecule localization (GO:0070727) | 258 | 2 | 1.00E+00 | 1.00E+00 |
| system process (GO:0003008) | 137 | 1 | 1.00E+00 | 1.00E+00 |
| cellular localization (GO:0051641) | 294 | 2 | 7.71E-01 | 1.00E+00 |
| multicellular organism development (GO:0007275) | 151 | 1 | 1.00E+00 | 1.00E+00 |
| nucleic acid metabolic process (GO:0090304) | 169 | 1 | 1.00E+00 | 1.00E+00 |
| intracellular protein transport (GO:0006886) | 208 | 1 | 7.28E-01 | 1.00E+00 |
| regulation of biosynthetic process (GO:0009889) | 238 | 1 | 5.21E-01 | 1.00E+00 |
| regulation of cellular biosynthetic process (GO:0031326) | 238 | 1 | 5.21E-01 | 1.00E+00 |
| transcription by RNA polymerase II (GO:0006366) | 292 | 1 | 3.76E-01 | 1.00E+00 |

Table S38. GO analysis in Struthioniformes in the 4-times hypothesis using PANTHER GO-Slim Cellular Component. P values are calculated by Fisher’s exact test and FDR values are calculated by the Benjamini–Hochberg procedure.

| GO term | Number of genes in reference | Number of genes in query | P-values | FDR values |
| --- | --- | --- | --- | --- |
| neuromuscular junction (GO:0031594) | 2 | 1 | 3.23E-02 | 1.00E+00 |
| polymeric cytoskeletal fiber (GO:0099513) | 3 | 1 | 4.29E-02 | 1.00E+00 |
| oligosaccharyltransferase complex (GO:0008250) | 3 | 1 | 4.29E-02 | 1.00E+00 |
| synapse (GO:0045202) | 3 | 1 | 4.29E-02 | 1.00E+00 |
| basolateral plasma membrane (GO:0016323) | 4 | 1 | 5.33E-02 | 1.00E+00 |
| membrane region (GO:0098589) | 4 | 1 | 5.33E-02 | 1.00E+00 |
| membrane raft (GO:0045121) | 4 | 1 | 5.33E-02 | 1.00E+00 |
| membrane microdomain (GO:0098857) | 4 | 1 | 5.33E-02 | 1.00E+00 |
| transcription factor TFIIIC complex (GO:0000127) | 4 | 1 | 5.33E-02 | 1.00E+00 |
| organelle part (GO:0044422) | 5 | 1 | 6.36E-02 | 1.00E+00 |
| anaphase-promoting complex (GO:0005680) | 5 | 1 | 6.36E-02 | 1.00E+00 |
| kinetochore (GO:0000776) | 6 | 1 | 7.38E-02 | 1.00E+00 |
| spindle pole (GO:0000922) | 7 | 1 | 8.39E-02 | 1.00E+00 |
| chromosome, centromeric region (GO:0000775) | 7 | 1 | 8.39E-02 | 1.00E+00 |
| focal adhesion (GO:0005925) | 8 | 1 | 9.39E-02 | 1.00E+00 |
| intraciliary transport particle B (GO:0030992) | 9 | 1 | 1.04E-01 | 1.00E+00 |
| whole membrane (GO:0098805) | 9 | 1 | 1.04E-01 | 1.00E+00 |
| anchoring junction (GO:0070161) | 10 | 1 | 1.14E-01 | 1.00E+00 |
| ionotropic glutamate receptor complex (GO:0008328) | 10 | 1 | 1.14E-01 | 1.00E+00 |
| adherens junction (GO:0005912) | 10 | 1 | 1.14E-01 | 1.00E+00 |
| cytosolic small ribosomal subunit (GO:0022627) | 10 | 1 | 1.14E-01 | 1.00E+00 |
| cyclin-dependent protein kinase holoenzyme complex (GO:0000307) | 11 | 1 | 1.23E-01 | 1.00E+00 |
| chromosomal region (GO:0098687) | 11 | 1 | 1.23E-01 | 1.00E+00 |
| microtubule organizing center part (GO:0044450) | 47 | 4 | 2.17E-03 | 9.26E-01 |
| centriole (GO:0005814) | 47 | 4 | 2.17E-03 | 4.63E-01 |
| cytoskeletal part (GO:0044430) | 12 | 1 | 1.33E-01 | 1.00E+00 |
| SCF ubiquitin ligase complex (GO:0019005) | 13 | 1 | 1.42E-01 | 1.00E+00 |
| centrosome (GO:0005813) | 39 | 3 | 1.04E-02 | 1.00E+00 |
| cytosolic ribosome (GO:0022626) | 27 | 2 | 3.92E-02 | 1.00E+00 |
| postsynaptic density (GO:0014069) | 14 | 1 | 1.52E-01 | 1.00E+00 |
| microtubule organizing center (GO:0005815) | 57 | 4 | 4.18E-03 | 5.95E-01 |
| external side of plasma membrane (GO:0009897) | 15 | 1 | 1.61E-01 | 1.00E+00 |
| spindle (GO:0005819) | 17 | 1 | 1.79E-01 | 1.00E+00 |
| nuclear transcription factor complex (GO:0044798) | 19 | 1 | 1.97E-01 | 1.00E+00 |
| cytosolic part (GO:0044445) | 40 | 2 | 7.61E-02 | 1.00E+00 |
| cell surface (GO:0009986) | 41 | 2 | 7.92E-02 | 1.00E+00 |
| cullin-RING ubiquitin ligase complex (GO:0031461) | 21 | 1 | 2.14E-01 | 1.00E+00 |
| postsynaptic membrane (GO:0045211) | 21 | 1 | 2.14E-01 | 1.00E+00 |
| receptor complex (GO:0043235) | 64 | 3 | 3.60E-02 | 1.00E+00 |
| postsynapse (GO:0098794) | 25 | 1 | 2.48E-01 | 1.00E+00 |
| plasma membrane protein complex (GO:0098797) | 52 | 2 | 1.17E-01 | 1.00E+00 |
| microtubule cytoskeleton (GO:0015630) | 105 | 4 | 3.03E-02 | 1.00E+00 |
| leaflet of membrane bilayer (GO:0097478) | 29 | 1 | 2.81E-01 | 1.00E+00 |
| cytoskeleton (GO:0005856) | 178 | 6 | 1.44E-02 | 1.00E+00 |
| transmembrane transporter complex (GO:1902495) | 32 | 1 | 3.04E-01 | 1.00E+00 |
| cell junction (GO:0030054) | 32 | 1 | 3.04E-01 | 1.00E+00 |
| ubiquitin ligase complex (GO:0000151) | 35 | 1 | 3.27E-01 | 1.00E+00 |
| integral component of plasma membrane (GO:0005887) | 213 | 6 | 3.09E-02 | 1.00E+00 |
| endoplasmic reticulum membrane (GO:0005789) | 37 | 1 | 3.41E-01 | 1.00E+00 |
| endoplasmic reticulum subcompartment (GO:0098827) | 39 | 1 | 3.56E-01 | 1.00E+00 |
| microtubule (GO:0005874) | 43 | 1 | 3.83E-01 | 1.00E+00 |
| membrane protein complex (GO:0098796) | 87 | 2 | 2.53E-01 | 1.00E+00 |
| cilium (GO:0005929) | 44 | 1 | 3.90E-01 | 1.00E+00 |
| integral component of membrane (GO:0016021) | 268 | 6 | 1.22E-01 | 1.00E+00 |
| intrinsic component of membrane (GO:0031224) | 269 | 6 | 1.23E-01 | 1.00E+00 |
| membrane part (GO:0044425) | 314 | 7 | 8.50E-02 | 1.00E+00 |
| membrane (GO:0016020) | 318 | 7 | 8.70E-02 | 1.00E+00 |
| intraciliary transport particle (GO:0030990) | 52 | 1 | 4.42E-01 | 1.00E+00 |
| neuron projection (GO:0043005) | 54 | 1 | 4.54E-01 | 1.00E+00 |
| intracellular non-membrane-bounded organelle (GO:0043232) | 342 | 6 | 2.75E-01 | 1.00E+00 |
| non-membrane-bounded organelle (GO:0043228) | 342 | 6 | 2.75E-01 | 1.00E+00 |
| cytosol (GO:0005829) | 234 | 4 | 3.29E-01 | 1.00E+00 |
| plasma membrane region (GO:0098590) | 128 | 2 | 6.53E-01 | 1.00E+00 |
| Golgi apparatus (GO:0005794) | 65 | 1 | 5.17E-01 | 1.00E+00 |
| actin cytoskeleton (GO:0015629) | 67 | 1 | 5.27E-01 | 1.00E+00 |
| cell (GO:0005623) | 1627 | 22 | 2.60E-01 | 1.00E+00 |
| plasma membrane part (GO:0044459) | 149 | 2 | 6.80E-01 | 1.00E+00 |
| cell part (GO:0044464) | 1620 | 21 | 3.96E-01 | 1.00E+00 |
| intracellular (GO:0005622) | 1347 | 17 | 5.48E-01 | 1.00E+00 |
| intracellular part (GO:0044424) | 1199 | 15 | 5.36E-01 | 1.00E+00 |
| protein-containing complex (GO:0032991) | 501 | 6 | 8.21E-01 | 1.00E+00 |
| organelle (GO:0043226) | 1177 | 14 | 7.53E-01 | 1.00E+00 |
| cytoplasm (GO:0005737) | 1127 | 13 | 8.73E-01 | 1.00E+00 |
| plasma membrane (GO:0005886) | 623 | 7 | 8.41E-01 | 1.00E+00 |
| cell periphery (GO:0071944) | 635 | 7 | 1.00E+00 | 1.00E+00 |
| organelle subcompartment (GO:0031984) | 95 | 1 | 1.00E+00 | 1.00E+00 |
| nucleus (GO:0005634) | 696 | 7 | 1.00E+00 | 1.00E+00 |
| catalytic complex (GO:1902494) | 100 | 1 | 1.00E+00 | 1.00E+00 |
| cytoplasmic part (GO:0044444) | 715 | 7 | 1.00E+00 | 1.00E+00 |
| intracellular membrane-bounded organelle (GO:0043231) | 716 | 7 | 8.50E-01 | 1.00E+00 |
| intracellular organelle (GO:0043229) | 717 | 7 | 8.50E-01 | 1.00E+00 |
| Unclassified (UNCLASSIFIED) | 2890 | 28 | 3.68E-01 | 1.00E+00 |
| ribonucleoprotein complex (GO:1990904) | 104 | 1 | 1.00E+00 | 1.00E+00 |
| neuron part (GO:0097458) | 110 | 1 | 1.00E+00 | 1.00E+00 |
| endomembrane system (GO:0012505) | 226 | 2 | 1.00E+00 | 1.00E+00 |
| vacuole (GO:0005773) | 358 | 3 | 7.97E-01 | 1.00E+00 |
| chromosomal part (GO:0044427) | 133 | 1 | 1.00E+00 | 1.00E+00 |
| chromosome (GO:0005694) | 146 | 1 | 1.00E+00 | 1.00E+00 |
| nuclear part (GO:0044428) | 303 | 2 | 7.73E-01 | 1.00E+00 |
| membrane-bounded organelle (GO:0043227) | 192 | 1 | 7.24E-01 | 1.00E+00 |

Table S39. GO analysis in Struthioniformes in the 4-times hypothesis using PANTHER GO-Slim Molecular Function. P values are calculated by Fisher’s exact test and FDR values are calculated by the Benjamini–Hochberg procedure.

| GO term | Number of genes in reference | Number of genes in query | P-values | FDR values |
| --- | --- | --- | --- | --- |
| protein tyrosine kinase binding (GO:1990782) | 1 | 1 | 2.17E-02 | 1.00E+00 |
| neutral amino acid transmembrane transporter activity (GO:0015175) | 2 | 1 | 3.23E-02 | 1.00E+00 |
| oxidoreductase activity, acting on the CH-CH group of donors, NAD or NADP as acceptor (GO:0016628) | 2 | 1 | 3.23E-02 | 1.00E+00 |
| ATPase activator activity (GO:0001671) | 3 | 1 | 4.29E-02 | 1.00E+00 |
| microtubule plus-end binding (GO:0051010) | 3 | 1 | 4.29E-02 | 1.00E+00 |
| oligosaccharyl transferase activity (GO:0004576) | 4 | 1 | 5.33E-02 | 1.00E+00 |
| protein binding, bridging (GO:0030674) | 6 | 1 | 7.38E-02 | 1.00E+00 |
| mRNA 3'-UTR binding (GO:0003730) | 7 | 1 | 8.39E-02 | 1.00E+00 |
| tRNA binding (GO:0000049) | 7 | 1 | 8.39E-02 | 1.00E+00 |
| integrin binding (GO:0005178) | 8 | 1 | 9.39E-02 | 1.00E+00 |
| L-amino acid transmembrane transporter activity (GO:0015179) | 9 | 1 | 1.04E-01 | 1.00E+00 |
| cyclic-nucleotide phosphodiesterase activity (GO:0004112) | 9 | 1 | 1.04E-01 | 1.00E+00 |
| antiporter activity (GO:0015297) | 9 | 1 | 1.04E-01 | 1.00E+00 |
| ribonucleoprotein complex binding (GO:0043021) | 21 | 2 | 2.55E-02 | 1.00E+00 |
| ribosome binding (GO:0043022) | 11 | 1 | 1.23E-01 | 1.00E+00 |
| cadherin binding (GO:0045296) | 11 | 1 | 1.23E-01 | 1.00E+00 |
| active transmembrane transporter activity (GO:0022804) | 13 | 1 | 1.42E-01 | 1.00E+00 |
| secondary active transmembrane transporter activity (GO:0015291) | 13 | 1 | 1.42E-01 | 1.00E+00 |
| sodium ion transmembrane transporter activity (GO:0015081) | 27 | 2 | 3.92E-02 | 1.00E+00 |
| ATPase activity, coupled to transmembrane movement of substances (GO:0042626) | 27 | 2 | 3.92E-02 | 1.00E+00 |
| protein serine/threonine phosphatase activity (GO:0004722) | 15 | 1 | 1.61E-01 | 1.00E+00 |
| oxidoreductase activity, acting on the CH-CH group of donors (GO:0016627) | 16 | 1 | 1.70E-01 | 1.00E+00 |
| ATPase activity, coupled to movement of substances (GO:0043492) | 32 | 2 | 5.23E-02 | 1.00E+00 |
| translation regulator activity (GO:0045182) | 17 | 1 | 1.79E-01 | 1.00E+00 |
| protein-containing complex binding (GO:0044877) | 51 | 3 | 2.06E-02 | 1.00E+00 |
| cell adhesion molecule binding (GO:0050839) | 18 | 1 | 1.88E-01 | 1.00E+00 |
| transmembrane receptor protein kinase activity (GO:0019199) | 18 | 1 | 1.88E-01 | 1.00E+00 |
| transmembrane receptor protein tyrosine kinase activity (GO:0004714) | 18 | 1 | 1.88E-01 | 1.00E+00 |
| mRNA binding (GO:0003729) | 56 | 3 | 2.59E-02 | 1.00E+00 |
| oxidoreductase activity, acting on the CH-OH group of donors, NAD or NADP as acceptor (GO:0016616) | 19 | 1 | 1.97E-01 | 1.00E+00 |
| protein kinase regulator activity (GO:0019887) | 19 | 1 | 1.97E-01 | 1.00E+00 |
| protein homodimerization activity (GO:0042803) | 19 | 1 | 1.97E-01 | 1.00E+00 |
| potassium ion transmembrane transporter activity (GO:0015079) | 39 | 2 | 7.29E-02 | 1.00E+00 |
| protein dimerization activity (GO:0046983) | 20 | 1 | 2.06E-01 | 1.00E+00 |
| oxidoreductase activity, acting on CH-OH group of donors (GO:0016614) | 20 | 1 | 2.06E-01 | 1.00E+00 |
| translation initiation factor activity (GO:0003743) | 20 | 1 | 2.06E-01 | 1.00E+00 |
| protein kinase binding (GO:0019901) | 42 | 2 | 8.24E-02 | 1.00E+00 |
| amino acid transmembrane transporter activity (GO:0015171) | 21 | 1 | 2.14E-01 | 1.00E+00 |
| cytokine activity (GO:0005125) | 21 | 1 | 2.14E-01 | 1.00E+00 |
| anion transmembrane transporter activity (GO:0008509) | 43 | 2 | 8.57E-02 | 1.00E+00 |
| cyclin-dependent protein serine/threonine kinase activity (GO:0004693) | 22 | 1 | 2.23E-01 | 1.00E+00 |
| cofactor binding (GO:0048037) | 22 | 1 | 2.23E-01 | 1.00E+00 |
| drug binding (GO:0008144) | 23 | 1 | 2.31E-01 | 1.00E+00 |
| nucleoside-triphosphatase regulator activity (GO:0060589) | 46 | 2 | 9.57E-02 | 1.00E+00 |
| kinase binding (GO:0019900) | 48 | 2 | 1.02E-01 | 1.00E+00 |
| tubulin binding (GO:0015631) | 49 | 2 | 1.06E-01 | 1.00E+00 |
| monovalent inorganic cation transmembrane transporter activity (GO:0015077) | 51 | 2 | 1.13E-01 | 1.00E+00 |
| metal ion transmembrane transporter activity (GO:0046873) | 56 | 2 | 1.31E-01 | 1.00E+00 |
| kinase regulator activity (GO:0019207) | 30 | 1 | 2.88E-01 | 1.00E+00 |
| protein serine/threonine kinase activity (GO:0004674) | 124 | 4 | 4.99E-02 | 1.00E+00 |
| signaling receptor binding (GO:0005102) | 94 | 3 | 8.83E-02 | 1.00E+00 |
| transferase activity, transferring hexosyl groups (GO:0016758) | 32 | 1 | 3.04E-01 | 1.00E+00 |
| protein kinase activity (GO:0004672) | 162 | 5 | 3.44E-02 | 1.00E+00 |
| phosphoric diester hydrolase activity (GO:0008081) | 34 | 1 | 3.19E-01 | 1.00E+00 |
| carboxylic acid transmembrane transporter activity (GO:0046943) | 36 | 1 | 3.34E-01 | 1.00E+00 |
| cytoskeletal protein binding (GO:0008092) | 114 | 3 | 1.34E-01 | 1.00E+00 |
| phosphoprotein phosphatase activity (GO:0004721) | 38 | 1 | 3.48E-01 | 1.00E+00 |
| ATPase activity, coupled (GO:0042623) | 77 | 2 | 2.12E-01 | 1.00E+00 |
| GTPase activator activity (GO:0005096) | 39 | 1 | 3.56E-01 | 1.00E+00 |
| ion transmembrane transporter activity (GO:0015075) | 158 | 4 | 9.90E-02 | 1.00E+00 |
| phosphotransferase activity, alcohol group as acceptor (GO:0016773) | 199 | 5 | 7.01E-02 | 1.00E+00 |
| GTPase regulator activity (GO:0030695) | 41 | 1 | 3.70E-01 | 1.00E+00 |
| actin binding (GO:0003779) | 42 | 1 | 3.77E-01 | 1.00E+00 |
| organic anion transmembrane transporter activity (GO:0008514) | 42 | 1 | 3.77E-01 | 1.00E+00 |
| microtubule binding (GO:0008017) | 43 | 1 | 3.83E-01 | 1.00E+00 |
| receptor ligand activity (GO:0048018) | 45 | 1 | 3.97E-01 | 1.00E+00 |
| calcium ion binding (GO:0005509) | 46 | 1 | 4.03E-01 | 1.00E+00 |
| ubiquitin-protein transferase activity (GO:0004842) | 93 | 2 | 2.77E-01 | 1.00E+00 |
| receptor regulator activity (GO:0030545) | 47 | 1 | 4.10E-01 | 1.00E+00 |
| ubiquitin protein ligase activity (GO:0061630) | 48 | 1 | 4.17E-01 | 1.00E+00 |
| ATPase activity (GO:0016887) | 96 | 2 | 2.89E-01 | 1.00E+00 |
| GTPase activity (GO:0003924) | 97 | 2 | 2.93E-01 | 1.00E+00 |
| RNA binding (GO:0003723) | 195 | 4 | 1.70E-01 | 1.00E+00 |
| phosphoric ester hydrolase activity (GO:0042578) | 101 | 2 | 3.09E-01 | 1.00E+00 |
| nucleotide binding (GO:0000166) | 51 | 1 | 4.36E-01 | 1.00E+00 |
| nucleoside-triphosphatase activity (GO:0017111) | 210 | 4 | 2.95E-01 | 1.00E+00 |
| transferase activity, transferring phosphorus-containing groups (GO:0016772) | 266 | 5 | 2.19E-01 | 1.00E+00 |
| pyrophosphatase activity (GO:0016462) | 216 | 4 | 3.03E-01 | 1.00E+00 |
| hydrolase activity, acting on acid anhydrides, in phosphorus-containing anhydrides (GO:0016818) | 216 | 4 | 3.03E-01 | 1.00E+00 |
| hydrolase activity, acting on acid anhydrides (GO:0016817) | 216 | 4 | 3.03E-01 | 1.00E+00 |
| inorganic cation transmembrane transporter activity (GO:0022890) | 109 | 2 | 3.42E-01 | 1.00E+00 |
| ubiquitin-like protein transferase activity (GO:0019787) | 115 | 2 | 3.65E-01 | 1.00E+00 |
| transferase activity, transferring glycosyl groups (GO:0016757) | 58 | 1 | 4.78E-01 | 1.00E+00 |
| cation transmembrane transporter activity (GO:0008324) | 119 | 2 | 3.81E-01 | 1.00E+00 |
| transmembrane transporter activity (GO:0022857) | 238 | 4 | 3.36E-01 | 1.00E+00 |
| molecular function regulator (GO:0098772) | 243 | 4 | 3.44E-01 | 1.00E+00 |
| enzyme regulator activity (GO:0030234) | 190 | 3 | 4.68E-01 | 1.00E+00 |
| transferase activity (GO:0016740) | 590 | 9 | 2.96E-01 | 1.00E+00 |
| phosphatase activity (GO:0016791) | 66 | 1 | 5.22E-01 | 1.00E+00 |
| protein binding (GO:0005515) | 690 | 10 | 3.31E-01 | 1.00E+00 |
| transporter activity (GO:0005215) | 292 | 4 | 5.63E-01 | 1.00E+00 |
| molecular transducer activity (GO:0060089) | 225 | 3 | 7.38E-01 | 1.00E+00 |
| metal ion binding (GO:0046872) | 75 | 1 | 5.67E-01 | 1.00E+00 |
| catalytic activity (GO:0003824) | 1504 | 18 | 6.66E-01 | 1.00E+00 |
| binding (GO:0005488) | 1436 | 17 | 7.69E-01 | 1.00E+00 |
| transcription regulator activity (GO:0140110) | 257 | 3 | 7.61E-01 | 1.00E+00 |
| hydrolase activity, acting on ester bonds (GO:0016788) | 174 | 2 | 7.18E-01 | 1.00E+00 |
| oxidoreductase activity (GO:0016491) | 177 | 2 | 7.22E-01 | 1.00E+00 |
| small molecule binding (GO:0036094) | 91 | 1 | 1.00E+00 | 1.00E+00 |
| enzyme binding (GO:0019899) | 187 | 2 | 1.00E+00 | 1.00E+00 |
| cation binding (GO:0043169) | 95 | 1 | 1.00E+00 | 1.00E+00 |
| signaling receptor activity (GO:0038023) | 201 | 2 | 1.00E+00 | 1.00E+00 |
| hydrolase activity (GO:0016787) | 607 | 6 | 1.00E+00 | 1.00E+00 |
| Unclassified (UNCLASSIFIED) | 2686 | 26 | 3.71E-01 | 1.00E+00 |
| DNA-binding transcription factor activity (GO:0003700) | 212 | 2 | 1.00E+00 | 1.00E+00 |
| structural molecule activity (GO:0005198) | 120 | 1 | 1.00E+00 | 1.00E+00 |
| nucleic acid binding (GO:0003676) | 532 | 4 | 6.59E-01 | 1.00E+00 |
| heterocyclic compound binding (GO:1901363) | 556 | 4 | 5.18E-01 | 1.00E+00 |
| transmembrane signaling receptor activity (GO:0004888) | 157 | 1 | 1.00E+00 | 1.00E+00 |
| ion binding (GO:0043167) | 166 | 1 | 1.00E+00 | 1.00E+00 |

Table S40. Pathway analysis for Struthioniformes in the 4-times hypothesis using PANTHER pathway. P values are calculated by Fisher’s exact test and FDR values are calculated by the Benjamini–Hochberg procedure.

| GO term | Number of genes in reference | Number of genes in query | P-values | FDR values |
| --- | --- | --- | --- | --- |
| Pyrimidine Metabolism (P02771) | 6 | 1 | 7.38E-02 | 1.00E+00 |
| Cell cycle (P00013) | 9 | 1 | 1.04E-01 | 1.00E+00 |
| Toll receptor signaling pathway (P00054) | 20 | 2 | 2.34E-02 | 1.00E+00 |
| FAS signaling pathway (P00020) | 10 | 1 | 1.14E-01 | 1.00E+00 |
| Integrin signalling pathway (P00034) | 44 | 3 | 1.42E-02 | 1.00E+00 |
| B cell activation (P00010) | 21 | 1 | 2.14E-01 | 1.00E+00 |
| Parkinson disease (P00049) | 22 | 1 | 2.23E-01 | 1.00E+00 |
| p53 pathway feedback loops 2 (P04398) | 22 | 1 | 2.23E-01 | 1.00E+00 |
| FGF signaling pathway (P00021) | 30 | 1 | 2.88E-01 | 1.00E+00 |
| p53 pathway (P00059) | 35 | 1 | 3.27E-01 | 1.00E+00 |
| Gonadotropin-releasing hormone receptor pathway (P06664) | 71 | 2 | 1.88E-01 | 1.00E+00 |
| EGF receptor signaling pathway (P00018) | 42 | 1 | 3.77E-01 | 1.00E+00 |
| Wnt signaling pathway (P00057) | 72 | 1 | 5.53E-01 | 1.00E+00 |
| Unclassified (UNCLASSIFIED) | 4758 | 51 | 5.81E-01 | 1.00E+00 |

Table S41. GO analysis in Apterygiformes in the 4-times hypothesis using PANTHER GO-Slim Biological Process. P values are calculated by Fisher’s exact test and FDR values are calculated by the Benjamini–Hochberg procedure.

| GO term | Number of genes in reference | Number of genes in query | P-values | FDR values |
| --- | --- | --- | --- | --- |
| peptidyl-proline hydroxylation (GO:0019511) | 3 | 1 | 5.30E-02 | 1.00E+00 |
| regulation of translational initiation (GO:0006446) | 3 | 1 | 5.30E-02 | 1.00E+00 |
| vesicle transport along microtubule (GO:0047496) | 3 | 1 | 5.30E-02 | 1.00E+00 |
| heart morphogenesis (GO:0003007) | 3 | 1 | 5.30E-02 | 1.00E+00 |
| muscle organ development (GO:0007517) | 4 | 1 | 6.59E-02 | 1.00E+00 |
| reactive oxygen species metabolic process (GO:0072593) | 14 | 3 | 1.41E-03 | 1.00E+00 |
| positive regulation of actin filament polymerization (GO:0030838) | 5 | 1 | 7.85E-02 | 1.00E+00 |
| integrin-mediated signaling pathway (GO:0007229) | 5 | 1 | 7.85E-02 | 1.00E+00 |
| organelle transport along microtubule (GO:0072384) | 5 | 1 | 7.85E-02 | 1.00E+00 |
| regulation of synaptic transmission, glutamatergic (GO:0051966) | 5 | 1 | 7.85E-02 | 1.00E+00 |
| endoplasmic reticulum unfolded protein response (GO:0030968) | 5 | 1 | 7.85E-02 | 1.00E+00 |
| microtubule depolymerization (GO:0007019) | 5 | 1 | 7.85E-02 | 1.00E+00 |
| nucleobase-containing compound catabolic process (GO:0034655) | 5 | 1 | 7.85E-02 | 1.00E+00 |
| actomyosin structure organization (GO:0031032) | 5 | 1 | 7.85E-02 | 1.00E+00 |
| myofibril assembly (GO:0030239) | 5 | 1 | 7.85E-02 | 1.00E+00 |
| purine nucleobase biosynthetic process (GO:0009113) | 6 | 1 | 9.10E-02 | 1.00E+00 |
| maturation of LSU-rRNA from tricistronic rRNA transcript (SSU-rRNA, 5.8S rRNA, LSU-rRNA) (GO:0000463) | 6 | 1 | 9.10E-02 | 1.00E+00 |
| nucleobase-containing small molecule biosynthetic process (GO:0034404) | 6 | 1 | 9.10E-02 | 1.00E+00 |
| cell adhesion mediated by integrin (GO:0033627) | 6 | 1 | 9.10E-02 | 1.00E+00 |
| response to unfolded protein (GO:0006986) | 6 | 1 | 9.10E-02 | 1.00E+00 |
| protein depolymerization (GO:0051261) | 6 | 1 | 9.10E-02 | 1.00E+00 |
| response to reactive oxygen species (GO:0000302) | 6 | 1 | 9.10E-02 | 1.00E+00 |
| nucleobase-containing compound biosynthetic process (GO:0034654) | 6 | 1 | 9.10E-02 | 1.00E+00 |
| cellular protein complex disassembly (GO:0043624) | 6 | 1 | 9.10E-02 | 1.00E+00 |
| cellular response to unfolded protein (GO:0034620) | 6 | 1 | 9.10E-02 | 1.00E+00 |
| histone lysine methylation (GO:0034968) | 6 | 1 | 9.10E-02 | 1.00E+00 |
| positive regulation of cytoskeleton organization (GO:0051495) | 7 | 1 | 1.03E-01 | 1.00E+00 |
| cellular response to oxidative stress (GO:0034599) | 7 | 1 | 1.03E-01 | 1.00E+00 |
| histone deacetylation (GO:0016575) | 7 | 1 | 1.03E-01 | 1.00E+00 |
| protein deacetylation (GO:0006476) | 7 | 1 | 1.03E-01 | 1.00E+00 |
| actin cytoskeleton reorganization (GO:0031532) | 7 | 1 | 1.03E-01 | 1.00E+00 |
| maturation of LSU-rRNA (GO:0000470) | 7 | 1 | 1.03E-01 | 1.00E+00 |
| positive regulation of cellular protein metabolic process (GO:0032270) | 7 | 1 | 1.03E-01 | 1.00E+00 |
| positive regulation of protein modification process (GO:0031401) | 7 | 1 | 1.03E-01 | 1.00E+00 |
| ribosomal large subunit assembly (GO:0000027) | 7 | 1 | 1.03E-01 | 1.00E+00 |
| peptidyl-lysine methylation (GO:0018022) | 8 | 1 | 1.15E-01 | 1.00E+00 |
| adenylate cyclase-inhibiting G-protein coupled receptor signaling pathway (GO:0007193) | 8 | 1 | 1.15E-01 | 1.00E+00 |
| muscle cell differentiation (GO:0042692) | 8 | 1 | 1.15E-01 | 1.00E+00 |
| establishment of organelle localization (GO:0051656) | 8 | 1 | 1.15E-01 | 1.00E+00 |
| muscle structure development (GO:0061061) | 8 | 1 | 1.15E-01 | 1.00E+00 |
| response to oxidative stress (GO:0006979) | 8 | 1 | 1.15E-01 | 1.00E+00 |
| negative regulation of catalytic activity (GO:0043086) | 8 | 1 | 1.15E-01 | 1.00E+00 |
| tissue morphogenesis (GO:0048729) | 9 | 1 | 1.27E-01 | 1.00E+00 |
| regulation of chromatin organization (GO:1902275) | 9 | 1 | 1.27E-01 | 1.00E+00 |
| protein deacylation (GO:0035601) | 9 | 1 | 1.27E-01 | 1.00E+00 |
| heart development (GO:0007507) | 9 | 1 | 1.27E-01 | 1.00E+00 |
| protein-containing complex disassembly (GO:0032984) | 9 | 1 | 1.27E-01 | 1.00E+00 |
| actin filament bundle assembly (GO:0051017) | 9 | 1 | 1.27E-01 | 1.00E+00 |
| lysosomal transport (GO:0007041) | 9 | 1 | 1.27E-01 | 1.00E+00 |
| intrinsic apoptotic signaling pathway (GO:0097193) | 9 | 1 | 1.27E-01 | 1.00E+00 |
| actin filament bundle organization (GO:0061572) | 10 | 1 | 1.39E-01 | 1.00E+00 |
| positive regulation of organelle organization (GO:0010638) | 10 | 1 | 1.39E-01 | 1.00E+00 |
| response to topologically incorrect protein (GO:0035966) | 10 | 1 | 1.39E-01 | 1.00E+00 |
| cell-substrate adhesion (GO:0031589) | 21 | 2 | 3.80E-02 | 1.00E+00 |
| cell-matrix adhesion (GO:0007160) | 21 | 2 | 3.80E-02 | 1.00E+00 |
| defense response (GO:0006952) | 21 | 2 | 3.80E-02 | 1.00E+00 |
| aromatic compound catabolic process (GO:0019439) | 11 | 1 | 1.51E-01 | 1.00E+00 |
| cellular component disassembly (GO:0022411) | 11 | 1 | 1.51E-01 | 1.00E+00 |
| cAMP-mediated signaling (GO:0019933) | 11 | 1 | 1.51E-01 | 1.00E+00 |
| small molecule catabolic process (GO:0044282) | 12 | 1 | 1.62E-01 | 1.00E+00 |
| synaptic transmission, glutamatergic (GO:0035249) | 12 | 1 | 1.62E-01 | 1.00E+00 |
| cellular response to oxygen-containing compound (GO:1901701) | 13 | 1 | 1.74E-01 | 1.00E+00 |
| cytoplasmic microtubule organization (GO:0031122) | 13 | 1 | 1.74E-01 | 1.00E+00 |
| negative regulation of molecular function (GO:0044092) | 13 | 1 | 1.74E-01 | 1.00E+00 |
| cellular amino acid catabolic process (GO:0009063) | 27 | 2 | 5.80E-02 | 1.00E+00 |
| regulation of cytoskeleton organization (GO:0051493) | 14 | 1 | 1.85E-01 | 1.00E+00 |
| pigment metabolic process (GO:0042440) | 14 | 1 | 1.85E-01 | 1.00E+00 |
| pigment biosynthetic process (GO:0046148) | 14 | 1 | 1.85E-01 | 1.00E+00 |
| regulation of chromosome organization (GO:0033044) | 14 | 1 | 1.85E-01 | 1.00E+00 |
| regulation of cellular protein metabolic process (GO:0032268) | 14 | 1 | 1.85E-01 | 1.00E+00 |
| ribosomal large subunit biogenesis (GO:0042273) | 14 | 1 | 1.85E-01 | 1.00E+00 |
| vacuolar transport (GO:0007034) | 14 | 1 | 1.85E-01 | 1.00E+00 |
| response to inorganic substance (GO:0010035) | 14 | 1 | 1.85E-01 | 1.00E+00 |
| glutamate receptor signaling pathway (GO:0007215) | 16 | 1 | 2.07E-01 | 1.00E+00 |
| response to stress (GO:0006950) | 65 | 4 | 1.38E-02 | 1.00E+00 |
| carboxylic acid catabolic process (GO:0046395) | 49 | 3 | 3.27E-02 | 1.00E+00 |
| organic acid catabolic process (GO:0016054) | 49 | 3 | 3.27E-02 | 1.00E+00 |
| regulation of adenylate cyclase activity (GO:0045761) | 33 | 2 | 8.07E-02 | 1.00E+00 |
| adenylate cyclase-modulating G-protein coupled receptor signaling pathway (GO:0007188) | 33 | 2 | 8.07E-02 | 1.00E+00 |
| regulation of cyclase activity (GO:0031279) | 34 | 2 | 8.47E-02 | 1.00E+00 |
| organic cyclic compound biosynthetic process (GO:1901362) | 17 | 1 | 2.18E-01 | 1.00E+00 |
| mitotic sister chromatid segregation (GO:0000070) | 17 | 1 | 2.18E-01 | 1.00E+00 |
| movement of cell or subcellular component (GO:0006928) | 17 | 1 | 2.18E-01 | 1.00E+00 |
| regulation of cAMP-mediated signaling (GO:0043949) | 35 | 2 | 8.88E-02 | 1.00E+00 |
| cellular modified amino acid metabolic process (GO:0006575) | 18 | 1 | 2.28E-01 | 1.00E+00 |
| regulation of organelle organization (GO:0033043) | 37 | 2 | 9.71E-02 | 1.00E+00 |
| positive regulation of cellular component organization (GO:0051130) | 19 | 1 | 2.39E-01 | 1.00E+00 |
| regulation of translation (GO:0006417) | 19 | 1 | 2.39E-01 | 1.00E+00 |
| circulatory system development (GO:0072359) | 19 | 1 | 2.39E-01 | 1.00E+00 |
| actin cytoskeleton organization (GO:0030036) | 58 | 3 | 4.90E-02 | 1.00E+00 |
| actin filament organization (GO:0007015) | 39 | 2 | 1.06E-01 | 1.00E+00 |
| regulation of cellular amide metabolic process (GO:0034248) | 20 | 1 | 2.49E-01 | 1.00E+00 |
| fatty acid catabolic process (GO:0009062) | 20 | 1 | 2.49E-01 | 1.00E+00 |
| G-protein coupled receptor signaling pathway, coupled to cyclic nucleotide second messenger (GO:0007187) | 40 | 2 | 1.10E-01 | 1.00E+00 |
| response to toxic substance (GO:0009636) | 20 | 1 | 2.49E-01 | 1.00E+00 |
| actin filament polymerization (GO:0030041) | 20 | 1 | 2.49E-01 | 1.00E+00 |
| regulation of trans-synaptic signaling (GO:0099177) | 20 | 1 | 2.49E-01 | 1.00E+00 |
| modulation of chemical synaptic transmission (GO:0050804) | 20 | 1 | 2.49E-01 | 1.00E+00 |
| cyclic-nucleotide-mediated signaling (GO:0019935) | 20 | 1 | 2.49E-01 | 1.00E+00 |
| organic substance catabolic process (GO:1901575) | 80 | 4 | 2.64E-02 | 1.00E+00 |
| developmental process (GO:0032502) | 110 | 5 | 1.92E-02 | 1.00E+00 |
| regulation of signaling (GO:0023051) | 22 | 1 | 2.69E-01 | 1.00E+00 |
| muscle contraction (GO:0006936) | 22 | 1 | 2.69E-01 | 1.00E+00 |
| negative regulation of intracellular signal transduction (GO:1902532) | 23 | 1 | 2.79E-01 | 1.00E+00 |
| organelle localization (GO:0051640) | 23 | 1 | 2.79E-01 | 1.00E+00 |
| recombinational repair (GO:0000725) | 23 | 1 | 2.79E-01 | 1.00E+00 |
| double-strand break repair via homologous recombination (GO:0000724) | 23 | 1 | 2.79E-01 | 1.00E+00 |
| muscle system process (GO:0003012) | 23 | 1 | 2.79E-01 | 1.00E+00 |
| peptidyl-lysine modification (GO:0018205) | 23 | 1 | 2.79E-01 | 1.00E+00 |
| organonitrogen compound catabolic process (GO:1901565) | 23 | 1 | 2.79E-01 | 1.00E+00 |
| catabolic process (GO:0009056) | 96 | 4 | 4.58E-02 | 1.00E+00 |
| activation of adenylate cyclase activity (GO:0007190) | 24 | 1 | 2.89E-01 | 1.00E+00 |
| adenylate cyclase-activating G-protein coupled receptor signaling pathway (GO:0007189) | 24 | 1 | 2.89E-01 | 1.00E+00 |
| actin polymerization or depolymerization (GO:0008154) | 24 | 1 | 2.89E-01 | 1.00E+00 |
| microtubule-based movement (GO:0007018) | 24 | 1 | 2.89E-01 | 1.00E+00 |
| cellular amino acid biosynthetic process (GO:0008652) | 25 | 1 | 2.99E-01 | 1.00E+00 |
| cytoskeleton organization (GO:0007010) | 75 | 3 | 8.82E-02 | 1.00E+00 |
| peptidyl-amino acid modification (GO:0018193) | 55 | 2 | 1.80E-01 | 1.00E+00 |
| regulation of catalytic activity (GO:0050790) | 56 | 2 | 1.85E-01 | 1.00E+00 |
| cellular aromatic compound metabolic process (GO:0006725) | 29 | 1 | 3.36E-01 | 1.00E+00 |
| cell adhesion (GO:0007155) | 88 | 3 | 1.25E-01 | 1.00E+00 |
| biological adhesion (GO:0022610) | 88 | 3 | 1.25E-01 | 1.00E+00 |
| animal organ development (GO:0048513) | 31 | 1 | 3.54E-01 | 1.00E+00 |
| regulation of cellular component organization (GO:0051128) | 64 | 2 | 2.24E-01 | 1.00E+00 |
| phosphate-containing compound metabolic process (GO:0006796) | 32 | 1 | 3.63E-01 | 1.00E+00 |
| sensory perception (GO:0007600) | 32 | 1 | 3.63E-01 | 1.00E+00 |
| proteolysis (GO:0006508) | 65 | 2 | 2.29E-01 | 1.00E+00 |
| nucleobase-containing compound metabolic process (GO:0006139) | 33 | 1 | 3.72E-01 | 1.00E+00 |
| tissue development (GO:0009888) | 33 | 1 | 3.72E-01 | 1.00E+00 |
| anatomical structure morphogenesis (GO:0009653) | 34 | 1 | 3.80E-01 | 1.00E+00 |
| carboxylic acid biosynthetic process (GO:0046394) | 35 | 1 | 3.89E-01 | 1.00E+00 |
| drug metabolic process (GO:0017144) | 35 | 1 | 3.89E-01 | 1.00E+00 |
| organic acid biosynthetic process (GO:0016053) | 35 | 1 | 3.89E-01 | 1.00E+00 |
| microtubule-based process (GO:0007017) | 70 | 2 | 2.55E-01 | 1.00E+00 |
| regulation of intracellular signal transduction (GO:1902531) | 74 | 2 | 2.75E-01 | 1.00E+00 |
| anatomical structure development (GO:0048856) | 75 | 2 | 2.80E-01 | 1.00E+00 |
| organelle assembly (GO:0070925) | 38 | 1 | 4.13E-01 | 1.00E+00 |
| bleb assembly (GO:0032060) | 39 | 1 | 4.21E-01 | 1.00E+00 |
| phosphorus metabolic process (GO:0006793) | 39 | 1 | 4.21E-01 | 1.00E+00 |
| organic cyclic compound metabolic process (GO:1901360) | 39 | 1 | 4.21E-01 | 1.00E+00 |
| execution phase of apoptosis (GO:0097194) | 39 | 1 | 4.21E-01 | 1.00E+00 |
| apoptotic signaling pathway (GO:0097190) | 39 | 1 | 4.21E-01 | 1.00E+00 |
| G-protein coupled receptor signaling pathway (GO:0007186) | 79 | 2 | 3.00E-01 | 1.00E+00 |
| cellular response to chemical stimulus (GO:0070887) | 40 | 1 | 4.29E-01 | 1.00E+00 |
| cellular protein-containing complex assembly (GO:0034622) | 40 | 1 | 4.29E-01 | 1.00E+00 |
| negative regulation of signal transduction (GO:0009968) | 41 | 1 | 4.37E-01 | 1.00E+00 |
| second-messenger-mediated signaling (GO:0019932) | 42 | 1 | 4.45E-01 | 1.00E+00 |
| protein metabolic process (GO:0019538) | 88 | 2 | 3.45E-01 | 1.00E+00 |
| oxidation-reduction process (GO:0055114) | 44 | 1 | 4.60E-01 | 1.00E+00 |
| cell surface receptor signaling pathway (GO:0007166) | 176 | 4 | 3.09E-01 | 1.00E+00 |
| cell migration (GO:0016477) | 44 | 1 | 4.60E-01 | 1.00E+00 |
| cell projection organization (GO:0030030) | 44 | 1 | 4.60E-01 | 1.00E+00 |
| negative regulation of response to stimulus (GO:0048585) | 44 | 1 | 4.60E-01 | 1.00E+00 |
| plasma membrane bounded cell projection organization (GO:0120036) | 44 | 1 | 4.60E-01 | 1.00E+00 |
| plasma membrane bounded cell projection assembly (GO:0120031) | 44 | 1 | 4.60E-01 | 1.00E+00 |
| positive regulation of cellular process (GO:0048522) | 45 | 1 | 4.67E-01 | 1.00E+00 |
| system process (GO:0003008) | 137 | 3 | 4.39E-01 | 1.00E+00 |
| response to stimulus (GO:0050896) | 230 | 5 | 2.49E-01 | 1.00E+00 |
| regulation of molecular function (GO:0065009) | 92 | 2 | 3.65E-01 | 1.00E+00 |
| microtubule cytoskeleton organization (GO:0000226) | 47 | 1 | 4.81E-01 | 1.00E+00 |
| regulation of cell communication (GO:0010646) | 95 | 2 | 3.79E-01 | 1.00E+00 |
| regulation of signal transduction (GO:0009966) | 95 | 2 | 3.79E-01 | 1.00E+00 |
| cellular amino acid metabolic process (GO:0006520) | 48 | 1 | 4.88E-01 | 1.00E+00 |
| ribosome biogenesis (GO:0042254) | 50 | 1 | 5.02E-01 | 1.00E+00 |
| localization of cell (GO:0051674) | 52 | 1 | 5.16E-01 | 1.00E+00 |
| cell motility (GO:0048870) | 52 | 1 | 5.16E-01 | 1.00E+00 |
| carbohydrate derivative biosynthetic process (GO:1901137) | 53 | 1 | 5.22E-01 | 1.00E+00 |
| proteasome-mediated ubiquitin-dependent protein catabolic process (GO:0043161) | 54 | 1 | 5.29E-01 | 1.00E+00 |
| cellular protein modification process (GO:0006464) | 274 | 5 | 4.28E-01 | 1.00E+00 |
| organic substance biosynthetic process (GO:1901576) | 111 | 2 | 6.66E-01 | 1.00E+00 |
| nervous system process (GO:0050877) | 112 | 2 | 6.68E-01 | 1.00E+00 |
| cellular metabolic process (GO:0044237) | 567 | 10 | 4.43E-01 | 1.00E+00 |
| proteasomal protein catabolic process (GO:0010498) | 59 | 1 | 5.60E-01 | 1.00E+00 |
| ribonucleoprotein complex biogenesis (GO:0022613) | 59 | 1 | 5.60E-01 | 1.00E+00 |
| cellular protein metabolic process (GO:0044267) | 297 | 5 | 6.04E-01 | 1.00E+00 |
| cellular macromolecule metabolic process (GO:0044260) | 297 | 5 | 6.04E-01 | 1.00E+00 |
| organelle organization (GO:0006996) | 242 | 4 | 5.75E-01 | 1.00E+00 |
| system development (GO:0048731) | 124 | 2 | 6.88E-01 | 1.00E+00 |
| cellular response to stimulus (GO:0051716) | 559 | 9 | 5.66E-01 | 1.00E+00 |
| signal transduction (GO:0007165) | 438 | 7 | 6.66E-01 | 1.00E+00 |
| cellular process (GO:0009987) | 1754 | 27 | 4.59E-01 | 1.00E+00 |
| Unclassified (UNCLASSIFIED) | 2556 | 39 | 4.18E-01 | 1.00E+00 |
| protein-containing complex assembly (GO:0065003) | 66 | 1 | 6.01E-01 | 1.00E+00 |
| regulation of response to stimulus (GO:0048583) | 68 | 1 | 6.12E-01 | 1.00E+00 |
| regulation of cellular process (GO:0050794) | 275 | 4 | 7.90E-01 | 1.00E+00 |
| apoptotic process (GO:0006915) | 69 | 1 | 6.17E-01 | 1.00E+00 |
| programmed cell death (GO:0012501) | 70 | 1 | 6.22E-01 | 1.00E+00 |
| cellular response to stress (GO:0033554) | 143 | 2 | 1.00E+00 | 1.00E+00 |
| neurogenesis (GO:0022008) | 72 | 1 | 1.00E+00 | 1.00E+00 |
| multicellular organismal process (GO:0032501) | 291 | 4 | 1.00E+00 | 1.00E+00 |
| cell death (GO:0008219) | 74 | 1 | 1.00E+00 | 1.00E+00 |
| protein ubiquitination (GO:0016567) | 75 | 1 | 1.00E+00 | 1.00E+00 |
| positive regulation of biological process (GO:0048518) | 75 | 1 | 1.00E+00 | 1.00E+00 |
| regulation of cellular metabolic process (GO:0031323) | 150 | 2 | 1.00E+00 | 1.00E+00 |
| multicellular organism development (GO:0007275) | 151 | 2 | 1.00E+00 | 1.00E+00 |
| primary metabolic process (GO:0044238) | 151 | 2 | 1.00E+00 | 1.00E+00 |
| DNA repair (GO:0006281) | 76 | 1 | 1.00E+00 | 1.00E+00 |
| mitotic nuclear division (GO:0140014) | 78 | 1 | 1.00E+00 | 1.00E+00 |
| cellular component organization (GO:0016043) | 474 | 6 | 1.00E+00 | 1.00E+00 |
| organonitrogen compound metabolic process (GO:1901564) | 79 | 1 | 1.00E+00 | 1.00E+00 |
| protein-containing complex subunit organization (GO:0043933) | 80 | 1 | 1.00E+00 | 1.00E+00 |
| mitotic cell cycle (GO:0000278) | 81 | 1 | 1.00E+00 | 1.00E+00 |
| mitotic cell cycle process (GO:1903047) | 81 | 1 | 1.00E+00 | 1.00E+00 |
| cellular component organization or biogenesis (GO:0071840) | 81 | 1 | 1.00E+00 | 1.00E+00 |
| protein modification by small protein conjugation or removal (GO:0070647) | 81 | 1 | 1.00E+00 | 1.00E+00 |
| protein modification by small protein conjugation (GO:0032446) | 81 | 1 | 1.00E+00 | 1.00E+00 |
| cellular component biogenesis (GO:0044085) | 81 | 1 | 1.00E+00 | 1.00E+00 |
| nervous system development (GO:0007399) | 88 | 1 | 1.00E+00 | 1.00E+00 |
| formation of translation initiation ternary complex (GO:0001677) | 93 | 1 | 1.00E+00 | 1.00E+00 |
| carbohydrate derivative metabolic process (GO:1901135) | 93 | 1 | 1.00E+00 | 1.00E+00 |
| translational termination (GO:0006415) | 93 | 1 | 1.00E+00 | 1.00E+00 |
| translational elongation (GO:0006414) | 93 | 1 | 1.00E+00 | 1.00E+00 |
| cellular response to DNA damage stimulus (GO:0006974) | 95 | 1 | 1.00E+00 | 1.00E+00 |
| proteolysis involved in cellular protein catabolic process (GO:0051603) | 96 | 1 | 1.00E+00 | 1.00E+00 |
| cellular protein catabolic process (GO:0044257) | 97 | 1 | 1.00E+00 | 1.00E+00 |
| chemical synaptic transmission (GO:0007268) | 98 | 1 | 1.00E+00 | 1.00E+00 |
| cellular localization (GO:0051641) | 294 | 3 | 7.98E-01 | 1.00E+00 |
| anterograde trans-synaptic signaling (GO:0098916) | 98 | 1 | 1.00E+00 | 1.00E+00 |
| trans-synaptic signaling (GO:0099537) | 99 | 1 | 1.00E+00 | 1.00E+00 |
| synaptic signaling (GO:0099536) | 99 | 1 | 1.00E+00 | 1.00E+00 |
| intracellular protein transport (GO:0006886) | 208 | 2 | 1.00E+00 | 1.00E+00 |
| translation (GO:0006412) | 108 | 1 | 1.00E+00 | 1.00E+00 |
| protein catabolic process (GO:0030163) | 111 | 1 | 1.00E+00 | 1.00E+00 |
| macromolecule catabolic process (GO:0009057) | 114 | 1 | 1.00E+00 | 1.00E+00 |
| localization (GO:0051179) | 597 | 5 | 3.47E-01 | 1.00E+00 |
| metabolic process (GO:0008152) | 1369 | 11 | 4.34E-02 | 1.00E+00 |
| cellular protein localization (GO:0034613) | 256 | 2 | 5.84E-01 | 1.00E+00 |
| cellular macromolecule localization (GO:0070727) | 258 | 2 | 5.84E-01 | 1.00E+00 |
| response to chemical (GO:0042221) | 134 | 1 | 1.00E+00 | 1.00E+00 |
| cell-cell signaling (GO:0007267) | 146 | 1 | 7.24E-01 | 1.00E+00 |
| cell communication (GO:0007154) | 146 | 1 | 7.24E-01 | 1.00E+00 |
| regulation of biological process (GO:0050789) | 755 | 5 | 8.93E-02 | 1.00E+00 |
| organic substance metabolic process (GO:0071704) | 1145 | 7 | 1.41E-02 | 1.00E+00 |
| establishment of localization (GO:0051234) | 346 | 2 | 3.31E-01 | 1.00E+00 |
| transport (GO:0006810) | 346 | 2 | 3.31E-01 | 1.00E+00 |
| biological regulation (GO:0065007) | 881 | 5 | 2.54E-02 | 1.00E+00 |
| cell cycle (GO:0007049) | 188 | 1 | 5.21E-01 | 1.00E+00 |
| intracellular signal transduction (GO:0035556) | 198 | 1 | 5.25E-01 | 1.00E+00 |
| regulation of metabolic process (GO:0019222) | 432 | 2 | 1.25E-01 | 1.00E+00 |
| macromolecule metabolic process (GO:0043170) | 818 | 3 | 4.86E-03 | 1.00E+00 |
| gene expression (GO:0010467) | 581 | 2 | 2.14E-02 | 1.00E+00 |
| transcription, DNA-templated (GO:0006351) | 359 | 1 | 9.20E-02 | 1.00E+00 |

Table S42. GO analysis in Apterygiformes in the 4-times hypothesis using PANTHER GO-Slim Cellular Component. P values are calculated by Fisher’s exact test and FDR values are calculated by the Benjamini–Hochberg procedure.

| GO term | Number of genes in reference | Number of genes in query | P-values | FDR values |
| --- | --- | --- | --- | --- |
| A band (GO:0031672) | 3 | 1 | 5.30E-02 | 1.00E+00 |
| mitotic spindle (GO:0072686) | 3 | 1 | 5.30E-02 | 1.00E+00 |
| spindle midzone (GO:0051233) | 4 | 1 | 6.59E-02 | 1.00E+00 |
| oxidoreductase complex (GO:1990204) | 8 | 2 | 7.58E-03 | 1.00E+00 |
| cytoplasmic microtubule (GO:0005881) | 5 | 1 | 7.85E-02 | 1.00E+00 |
| spindle microtubule (GO:0005876) | 5 | 1 | 7.85E-02 | 1.00E+00 |
| cytoplasmic dynein complex (GO:0005868) | 6 | 1 | 9.10E-02 | 1.00E+00 |
| proteasome regulatory particle, base subcomplex (GO:0008540) | 6 | 1 | 9.10E-02 | 1.00E+00 |
| focal adhesion (GO:0005925) | 8 | 1 | 1.15E-01 | 1.00E+00 |
| proteasome accessory complex (GO:0022624) | 8 | 1 | 1.15E-01 | 1.00E+00 |
| proteasome regulatory particle (GO:0005838) | 8 | 1 | 1.15E-01 | 1.00E+00 |
| anchoring junction (GO:0070161) | 10 | 1 | 1.39E-01 | 1.00E+00 |
| adherens junction (GO:0005912) | 10 | 1 | 1.39E-01 | 1.00E+00 |
| Z disc (GO:0030018) | 10 | 1 | 1.39E-01 | 1.00E+00 |
| I band (GO:0031674) | 11 | 1 | 1.51E-01 | 1.00E+00 |
| cytoskeletal part (GO:0044430) | 12 | 1 | 1.62E-01 | 1.00E+00 |
| dynein complex (GO:0030286) | 13 | 1 | 1.74E-01 | 1.00E+00 |
| sarcomere (GO:0030017) | 15 | 1 | 1.96E-01 | 1.00E+00 |
| myofibril (GO:0030016) | 15 | 1 | 1.96E-01 | 1.00E+00 |
| contractile fiber (GO:0043292) | 16 | 1 | 2.07E-01 | 1.00E+00 |
| supramolecular polymer (GO:0099081) | 17 | 1 | 2.18E-01 | 1.00E+00 |
| supramolecular complex (GO:0099080) | 17 | 1 | 2.18E-01 | 1.00E+00 |
| supramolecular fiber (GO:0099512) | 17 | 1 | 2.18E-01 | 1.00E+00 |
| spindle (GO:0005819) | 17 | 1 | 2.18E-01 | 1.00E+00 |
| proteasome complex (GO:0000502) | 17 | 1 | 2.18E-01 | 1.00E+00 |
| plasma membrane protein complex (GO:0098797) | 52 | 3 | 3.78E-02 | 1.00E+00 |
| microtubule associated complex (GO:0005875) | 18 | 1 | 2.28E-01 | 1.00E+00 |
| small-subunit processome (GO:0032040) | 19 | 1 | 2.39E-01 | 1.00E+00 |
| cytosolic part (GO:0044445) | 40 | 2 | 1.10E-01 | 1.00E+00 |
| membrane protein complex (GO:0098796) | 87 | 4 | 3.41E-02 | 1.00E+00 |
| actin cytoskeleton (GO:0015629) | 67 | 3 | 6.84E-02 | 1.00E+00 |
| t-UTP complex (GO:0034455) | 27 | 1 | 3.18E-01 | 1.00E+00 |
| cytosolic ribosome (GO:0022626) | 27 | 1 | 3.18E-01 | 1.00E+00 |
| nucleolus (GO:0005730) | 58 | 2 | 1.95E-01 | 1.00E+00 |
| integral component of plasma membrane (GO:0005887) | 213 | 7 | 2.84E-02 | 1.00E+00 |
| nucleolar part (GO:0044452) | 31 | 1 | 3.54E-01 | 1.00E+00 |
| cell junction (GO:0030054) | 32 | 1 | 3.63E-01 | 1.00E+00 |
| preribosome (GO:0030684) | 35 | 1 | 3.89E-01 | 1.00E+00 |
| endoplasmic reticulum membrane (GO:0005789) | 37 | 1 | 4.05E-01 | 1.00E+00 |
| integral component of membrane (GO:0016021) | 268 | 7 | 9.86E-02 | 1.00E+00 |
| intrinsic component of membrane (GO:0031224) | 269 | 7 | 9.95E-02 | 1.00E+00 |
| endoplasmic reticulum subcompartment (GO:0098827) | 39 | 1 | 4.21E-01 | 1.00E+00 |
| cell surface (GO:0009986) | 41 | 1 | 4.37E-01 | 1.00E+00 |
| microtubule (GO:0005874) | 43 | 1 | 4.52E-01 | 1.00E+00 |
| cytoskeleton (GO:0005856) | 178 | 4 | 3.12E-01 | 1.00E+00 |
| membrane part (GO:0044425) | 314 | 7 | 2.05E-01 | 1.00E+00 |
| membrane (GO:0016020) | 318 | 7 | 2.08E-01 | 1.00E+00 |
| extracellular matrix (GO:0031012) | 49 | 1 | 4.95E-01 | 1.00E+00 |
| mitochondrion (GO:0005739) | 107 | 2 | 6.60E-01 | 1.00E+00 |
| cytosol (GO:0005829) | 234 | 4 | 5.64E-01 | 1.00E+00 |
| Unclassified (UNCLASSIFIED) | 2890 | 48 | 6.41E-02 | 1.00E+00 |
| nuclear lumen (GO:0031981) | 186 | 3 | 7.42E-01 | 1.00E+00 |
| extracellular region part (GO:0044421) | 124 | 2 | 6.88E-01 | 1.00E+00 |
| receptor complex (GO:0043235) | 64 | 1 | 5.90E-01 | 1.00E+00 |
| extracellular region (GO:0005576) | 151 | 2 | 1.00E+00 | 1.00E+00 |
| endoplasmic reticulum (GO:0005783) | 80 | 1 | 1.00E+00 | 1.00E+00 |
| protein-containing complex (GO:0032991) | 501 | 6 | 1.00E+00 | 1.00E+00 |
| intracellular non-membrane-bounded organelle (GO:0043232) | 342 | 4 | 1.00E+00 | 1.00E+00 |
| non-membrane-bounded organelle (GO:0043228) | 342 | 4 | 1.00E+00 | 1.00E+00 |
| nucleus (GO:0005634) | 696 | 8 | 7.28E-01 | 1.00E+00 |
| cytoplasmic part (GO:0044444) | 715 | 8 | 7.29E-01 | 1.00E+00 |
| intracellular membrane-bounded organelle (GO:0043231) | 716 | 8 | 7.29E-01 | 1.00E+00 |
| intracellular organelle (GO:0043229) | 717 | 8 | 7.29E-01 | 1.00E+00 |
| organelle subcompartment (GO:0031984) | 95 | 1 | 1.00E+00 | 1.00E+00 |
| nucleoplasm (GO:0005654) | 99 | 1 | 1.00E+00 | 1.00E+00 |
| catalytic complex (GO:1902494) | 100 | 1 | 1.00E+00 | 1.00E+00 |
| nuclear part (GO:0044428) | 303 | 3 | 7.98E-01 | 1.00E+00 |
| cell part (GO:0044464) | 1620 | 16 | 1.28E-01 | 1.00E+00 |
| cell (GO:0005623) | 1627 | 16 | 1.28E-01 | 1.00E+00 |
| extracellular space (GO:0005615) | 102 | 1 | 1.00E+00 | 1.00E+00 |
| ribonucleoprotein complex (GO:1990904) | 104 | 1 | 1.00E+00 | 1.00E+00 |
| microtubule cytoskeleton (GO:0015630) | 105 | 1 | 1.00E+00 | 1.00E+00 |
| organelle (GO:0043226) | 1177 | 11 | 2.02E-01 | 1.00E+00 |
| intracellular (GO:0005622) | 1347 | 12 | 1.05E-01 | 1.00E+00 |
| cytoplasm (GO:0005737) | 1127 | 9 | 8.29E-02 | 1.00E+00 |
| intracellular part (GO:0044424) | 1199 | 9 | 4.76E-02 | 1.00E+00 |
| vacuole (GO:0005773) | 358 | 2 | 2.37E-01 | 1.00E+00 |
| membrane-bounded organelle (GO:0043227) | 192 | 1 | 5.22E-01 | 1.00E+00 |
| plasma membrane (GO:0005886) | 623 | 3 | 4.26E-02 | 1.00E+00 |
| cell periphery (GO:0071944) | 635 | 3 | 4.29E-02 | 1.00E+00 |
| endomembrane system (GO:0012505) | 226 | 1 | 3.73E-01 | 1.00E+00 |

Table S43. GO analysis in Apterygiformes in the 4-times hypothesis using PANTHER GO-Slim Molecular Function. P values are calculated by Fisher’s exact test and FDR values are calculated by the Benjamini–Hochberg procedure.

| GO term | Number of genes in reference | Number of genes in query | P-values | FDR values |
| --- | --- | --- | --- | --- |
| copper ion binding (GO:0005507) | 6 | 1 | 9.10E-02 | 1.00E+00 |
| repressing transcription factor binding (GO:0070491) | 8 | 1 | 1.15E-01 | 1.00E+00 |
| integrin binding (GO:0005178) | 8 | 1 | 1.15E-01 | 1.00E+00 |
| purine nucleotide binding (GO:0017076) | 8 | 1 | 1.15E-01 | 1.00E+00 |
| hydrolase activity, acting on carbon-nitrogen (but not peptide) bonds, in linear amides (GO:0016811) | 8 | 1 | 1.15E-01 | 1.00E+00 |
| ammonium transmembrane transporter activity (GO:0008519) | 8 | 1 | 1.15E-01 | 1.00E+00 |
| chromatin DNA binding (GO:0031490) | 9 | 1 | 1.27E-01 | 1.00E+00 |
| hydrolase activity, acting on carbon-nitrogen (but not peptide) bonds (GO:0016810) | 18 | 2 | 2.93E-02 | 1.00E+00 |
| calcium channel activity (GO:0005262) | 10 | 1 | 1.39E-01 | 1.00E+00 |
| ATP-dependent microtubule motor activity, plus-end-directed (GO:0008574) | 10 | 1 | 1.39E-01 | 1.00E+00 |
| oxidoreductase activity, acting on paired donors, with incorporation or reduction of molecular oxygen, 2-oxoglutarate as one donor, and incorporation of one atom each of oxygen into both donors (GO:0016706) | 11 | 1 | 1.51E-01 | 1.00E+00 |
| calcium ion transmembrane transporter activity (GO:0015085) | 12 | 1 | 1.62E-01 | 1.00E+00 |
| acetyltransferase activity (GO:0016407) | 14 | 1 | 1.85E-01 | 1.00E+00 |
| glutamate receptor activity (GO:0008066) | 17 | 1 | 2.18E-01 | 1.00E+00 |
| ribonuclease activity (GO:0004540) | 17 | 1 | 2.18E-01 | 1.00E+00 |
| glutamate binding (GO:0016595) | 17 | 1 | 2.18E-01 | 1.00E+00 |
| endoribonuclease activity (GO:0004521) | 17 | 1 | 2.18E-01 | 1.00E+00 |
| adenylate cyclase activity (GO:0004016) | 34 | 2 | 8.47E-02 | 1.00E+00 |
| translation regulator activity (GO:0045182) | 17 | 1 | 2.18E-01 | 1.00E+00 |
| unfolded protein binding (GO:0051082) | 18 | 1 | 2.28E-01 | 1.00E+00 |
| phosphorus-oxygen lyase activity (GO:0016849) | 37 | 2 | 9.71E-02 | 1.00E+00 |
| oxidoreductase activity, acting on paired donors, with incorporation or reduction of molecular oxygen (GO:0016705) | 19 | 1 | 2.39E-01 | 1.00E+00 |
| cytokine receptor binding (GO:0005126) | 19 | 1 | 2.39E-01 | 1.00E+00 |
| motor activity (GO:0003774) | 20 | 1 | 2.49E-01 | 1.00E+00 |
| cytokine activity (GO:0005125) | 21 | 1 | 2.59E-01 | 1.00E+00 |
| actin filament binding (GO:0051015) | 21 | 1 | 2.59E-01 | 1.00E+00 |
| transferase activity, transferring acyl groups (GO:0016746) | 63 | 3 | 5.94E-02 | 1.00E+00 |
| amino acid binding (GO:0016597) | 22 | 1 | 2.69E-01 | 1.00E+00 |
| lipid transporter activity (GO:0005319) | 23 | 1 | 2.79E-01 | 1.00E+00 |
| Rab GTPase binding (GO:0017137) | 25 | 1 | 2.99E-01 | 1.00E+00 |
| ATPase activity, coupled (GO:0042623) | 77 | 3 | 9.35E-02 | 1.00E+00 |
| transition metal ion binding (GO:0046914) | 26 | 1 | 3.08E-01 | 1.00E+00 |
| ATPase activity, coupled to transmembrane movement of substances (GO:0042626) | 27 | 1 | 3.18E-01 | 1.00E+00 |
| carboxylic acid binding (GO:0031406) | 29 | 1 | 3.36E-01 | 1.00E+00 |
| organic acid binding (GO:0043177) | 31 | 1 | 3.54E-01 | 1.00E+00 |
| ATPase activity (GO:0016887) | 96 | 3 | 1.50E-01 | 1.00E+00 |
| ATPase activity, coupled to movement of substances (GO:0043492) | 32 | 1 | 3.63E-01 | 1.00E+00 |
| oxidoreductase activity (GO:0016491) | 177 | 5 | 9.91E-02 | 1.00E+00 |
| G-protein coupled receptor activity (GO:0004930) | 107 | 3 | 1.86E-01 | 1.00E+00 |
| small GTPase binding (GO:0031267) | 39 | 1 | 4.21E-01 | 1.00E+00 |
| GTPase activator activity (GO:0005096) | 39 | 1 | 4.21E-01 | 1.00E+00 |
| Ras GTPase binding (GO:0017016) | 39 | 1 | 4.21E-01 | 1.00E+00 |
| endopeptidase activity (GO:0004175) | 41 | 1 | 4.37E-01 | 1.00E+00 |
| RNA polymerase II regulatory region sequence-specific DNA binding (GO:0000977) | 41 | 1 | 4.37E-01 | 1.00E+00 |
| ligase activity, forming carbon-sulfur bonds (GO:0016877) | 41 | 1 | 4.37E-01 | 1.00E+00 |
| GTPase regulator activity (GO:0030695) | 41 | 1 | 4.37E-01 | 1.00E+00 |
| actin binding (GO:0003779) | 42 | 1 | 4.45E-01 | 1.00E+00 |
| lyase activity (GO:0016829) | 84 | 2 | 3.25E-01 | 1.00E+00 |
| microtubule binding (GO:0008017) | 43 | 1 | 4.52E-01 | 1.00E+00 |
| receptor ligand activity (GO:0048018) | 45 | 1 | 4.67E-01 | 1.00E+00 |
| small molecule binding (GO:0036094) | 91 | 2 | 3.60E-01 | 1.00E+00 |
| nucleoside-triphosphatase regulator activity (GO:0060589) | 46 | 1 | 4.74E-01 | 1.00E+00 |
| ubiquitin-protein transferase activity (GO:0004842) | 93 | 2 | 3.69E-01 | 1.00E+00 |
| receptor regulator activity (GO:0030545) | 47 | 1 | 4.81E-01 | 1.00E+00 |
| kinase binding (GO:0019900) | 48 | 1 | 4.88E-01 | 1.00E+00 |
| transferase activity, transferring acyl groups other than amino-acyl groups (GO:0016747) | 48 | 1 | 4.88E-01 | 1.00E+00 |
| tubulin binding (GO:0015631) | 49 | 1 | 4.95E-01 | 1.00E+00 |
| protein-containing complex binding (GO:0044877) | 51 | 1 | 5.09E-01 | 1.00E+00 |
| nucleotide binding (GO:0000166) | 51 | 1 | 5.09E-01 | 1.00E+00 |
| transmembrane signaling receptor activity (GO:0004888) | 157 | 3 | 4.78E-01 | 1.00E+00 |
| nucleoside-triphosphatase activity (GO:0017111) | 210 | 4 | 5.36E-01 | 1.00E+00 |
| pyrophosphatase activity (GO:0016462) | 216 | 4 | 5.42E-01 | 1.00E+00 |
| hydrolase activity, acting on acid anhydrides, in phosphorus-containing anhydrides (GO:0016818) | 216 | 4 | 5.42E-01 | 1.00E+00 |
| hydrolase activity, acting on acid anhydrides (GO:0016817) | 216 | 4 | 5.42E-01 | 1.00E+00 |
| transcription factor binding (GO:0008134) | 55 | 1 | 5.35E-01 | 1.00E+00 |
| ligase activity (GO:0016874) | 111 | 2 | 6.66E-01 | 1.00E+00 |
| metal ion transmembrane transporter activity (GO:0046873) | 56 | 1 | 5.42E-01 | 1.00E+00 |
| cytoskeletal protein binding (GO:0008092) | 114 | 2 | 6.71E-01 | 1.00E+00 |
| ubiquitin-like protein transferase activity (GO:0019787) | 115 | 2 | 6.73E-01 | 1.00E+00 |
| chromatin binding (GO:0003682) | 58 | 1 | 5.54E-01 | 1.00E+00 |
| cation transmembrane transporter activity (GO:0008324) | 119 | 2 | 6.79E-01 | 1.00E+00 |
| structural molecule activity (GO:0005198) | 120 | 2 | 6.81E-01 | 1.00E+00 |
| catalytic activity, acting on a protein (GO:0140096) | 60 | 1 | 5.66E-01 | 1.00E+00 |
| Unclassified (UNCLASSIFIED) | 2686 | 44 | 1.07E-01 | 1.00E+00 |
| protein serine/threonine kinase activity (GO:0004674) | 124 | 2 | 6.88E-01 | 1.00E+00 |
| enzyme binding (GO:0019899) | 187 | 3 | 7.43E-01 | 1.00E+00 |
| signaling receptor activity (GO:0038023) | 201 | 3 | 7.56E-01 | 1.00E+00 |
| GTPase binding (GO:0051020) | 68 | 1 | 6.12E-01 | 1.00E+00 |
| catalytic activity (GO:0003824) | 1504 | 22 | 6.99E-01 | 1.00E+00 |
| catalytic activity, acting on RNA (GO:0140098) | 73 | 1 | 1.00E+00 | 1.00E+00 |
| transcription coregulator activity (GO:0003712) | 73 | 1 | 1.00E+00 | 1.00E+00 |
| transferase activity (GO:0016740) | 590 | 8 | 1.00E+00 | 1.00E+00 |
| molecular transducer activity (GO:0060089) | 225 | 3 | 1.00E+00 | 1.00E+00 |
| metal ion binding (GO:0046872) | 75 | 1 | 1.00E+00 | 1.00E+00 |
| hydrolase activity (GO:0016787) | 607 | 8 | 1.00E+00 | 1.00E+00 |
| transcription regulatory region sequence-specific DNA binding (GO:0000976) | 78 | 1 | 1.00E+00 | 1.00E+00 |
| ion transmembrane transporter activity (GO:0015075) | 158 | 2 | 1.00E+00 | 1.00E+00 |
| protein kinase activity (GO:0004672) | 162 | 2 | 1.00E+00 | 1.00E+00 |
| signaling receptor binding (GO:0005102) | 94 | 1 | 1.00E+00 | 1.00E+00 |
| cation binding (GO:0043169) | 95 | 1 | 1.00E+00 | 1.00E+00 |
| GTPase activity (GO:0003924) | 97 | 1 | 1.00E+00 | 1.00E+00 |
| transporter activity (GO:0005215) | 292 | 3 | 7.98E-01 | 1.00E+00 |
| protein binding (GO:0005515) | 690 | 7 | 4.85E-01 | 1.00E+00 |
| phosphotransferase activity, alcohol group as acceptor (GO:0016773) | 199 | 2 | 1.00E+00 | 1.00E+00 |
| transcription regulatory region DNA binding (GO:0044212) | 100 | 1 | 1.00E+00 | 1.00E+00 |
| DNA-binding transcription factor activity (GO:0003700) | 212 | 2 | 1.00E+00 | 1.00E+00 |
| inorganic cation transmembrane transporter activity (GO:0022890) | 109 | 1 | 1.00E+00 | 1.00E+00 |
| transmembrane transporter activity (GO:0022857) | 238 | 2 | 7.72E-01 | 1.00E+00 |
| binding (GO:0005488) | 1436 | 12 | 4.74E-02 | 1.00E+00 |
| molecular function regulator (GO:0098772) | 243 | 2 | 7.74E-01 | 1.00E+00 |
| transcription regulator activity (GO:0140110) | 257 | 2 | 5.84E-01 | 1.00E+00 |
| transferase activity, transferring phosphorus-containing groups (GO:0016772) | 266 | 2 | 5.85E-01 | 1.00E+00 |
| DNA binding (GO:0003677) | 302 | 2 | 4.40E-01 | 1.00E+00 |
| peptidase activity, acting on L-amino acid peptides (GO:0070011) | 156 | 1 | 7.25E-01 | 1.00E+00 |
| peptidase activity (GO:0008233) | 166 | 1 | 7.28E-01 | 1.00E+00 |
| ion binding (GO:0043167) | 166 | 1 | 7.28E-01 | 1.00E+00 |
| enzyme regulator activity (GO:0030234) | 190 | 1 | 5.21E-01 | 1.00E+00 |
| nucleic acid binding (GO:0003676) | 532 | 2 | 4.53E-02 | 1.00E+00 |
| heterocyclic compound binding (GO:1901363) | 556 | 2 | 3.12E-02 | 1.00E+00 |

Table S44. Pathway analysis for Apterygiformes in the 4-times hypothesis using PANTHER pathway. P values are calculated by Fisher’s exact test and FDR values are calculated by the Benjamini–Hochberg procedure.

| GO term | Number of genes in reference | Number of genes in query | P-values | FDR values |
| --- | --- | --- | --- | --- |
| Adenine and hypoxanthine salvage pathway (P02723) | 5 | 1 | 7.85E-02 | 1.00E+00 |
| Blood coagulation (P00011) | 10 | 2 | 1.09E-02 | 1.00E+00 |
| Metabotropic glutamate receptor group II pathway (P00040) | 9 | 1 | 1.27E-01 | 1.00E+00 |
| FAS signaling pathway (P00020) | 10 | 1 | 1.39E-01 | 1.00E+00 |
| Nicotine pharmacodynamics pathway (P06587) | 12 | 1 | 1.62E-01 | 1.00E+00 |
| Cytoskeletal regulation by Rho GTPase (P00016) | 14 | 1 | 1.85E-01 | 1.00E+00 |
| Dopamine receptor mediated signaling pathway (P05912) | 20 | 1 | 2.49E-01 | 1.00E+00 |
| Ubiquitin proteasome pathway (P00060) | 21 | 1 | 2.59E-01 | 1.00E+00 |
| Ionotropic glutamate receptor pathway (P00037) | 21 | 1 | 2.59E-01 | 1.00E+00 |
| Integrin signalling pathway (P00034) | 44 | 2 | 1.28E-01 | 1.00E+00 |
| Cadherin signaling pathway (P00012) | 24 | 1 | 2.89E-01 | 1.00E+00 |
| Alzheimer disease-presenilin pathway (P00004) | 30 | 1 | 3.45E-01 | 1.00E+00 |
| Heterotrimeric G-protein signaling pathway-Gq alpha and Go alpha mediated pathway (P00027) | 34 | 1 | 3.80E-01 | 1.00E+00 |
| Apoptosis signaling pathway (P00006) | 35 | 1 | 3.89E-01 | 1.00E+00 |
| p53 pathway (P00059) | 35 | 1 | 3.89E-01 | 1.00E+00 |
| Wnt signaling pathway (P00057) | 72 | 2 | 2.65E-01 | 1.00E+00 |
| Huntington disease (P00029) | 36 | 1 | 3.97E-01 | 1.00E+00 |
| Heterotrimeric G-protein signaling pathway-Gi alpha and Gs alpha mediated pathway (P00026) | 55 | 1 | 5.35E-01 | 1.00E+00 |
| Inflammation mediated by chemokine and cytokine signaling pathway (P00031) | 60 | 1 | 5.66E-01 | 1.00E+00 |
| Unclassified (UNCLASSIFIED) | 4758 | 61 | 1.84E-01 | 1.00E+00 |

Table S45. GO analysis in Rheiformes in the 4-times hypothesis using PANTHER GO-Slim Biological Process. P values are calculated by Fisher’s exact test and FDR values are calculated by the Benjamini–Hochberg procedure.

| GO term | Number of genes in reference | Number of genes in query | P-values | FDR values |
| --- | --- | --- | --- | --- |
| collagen fibril organization (GO:0030199) | 3 | 1 | 3.74E-02 | 1.00E+00 |
| rRNA modification (GO:0000154) | 3 | 1 | 3.74E-02 | 1.00E+00 |
| negative regulation of DNA-binding transcription factor activity (GO:0043433) | 3 | 1 | 3.74E-02 | 1.00E+00 |
| positive regulation of canonical Wnt signaling pathway (GO:0090263) | 3 | 1 | 3.74E-02 | 1.00E+00 |
| regulation of systemic arterial blood pressure (GO:0003073) | 3 | 1 | 3.74E-02 | 1.00E+00 |
| phagocytosis (GO:0006909) | 3 | 1 | 3.74E-02 | 1.00E+00 |
| regulation of blood pressure (GO:0008217) | 4 | 1 | 4.65E-02 | 1.00E+00 |
| positive regulation of actin filament polymerization (GO:0030838) | 5 | 1 | 5.55E-02 | 1.00E+00 |
| regulation of anatomical structure size (GO:0090066) | 5 | 1 | 5.55E-02 | 1.00E+00 |
| positive regulation of Wnt signaling pathway (GO:0030177) | 5 | 1 | 5.55E-02 | 1.00E+00 |
| organelle transport along microtubule (GO:0072384) | 5 | 1 | 5.55E-02 | 1.00E+00 |
| sterol biosynthetic process (GO:0016126) | 5 | 1 | 5.55E-02 | 1.00E+00 |
| regulation of mitochondrion organization (GO:0010821) | 5 | 1 | 5.55E-02 | 1.00E+00 |
| cytokine production (GO:0001816) | 5 | 1 | 5.55E-02 | 1.00E+00 |
| regulation of synaptic transmission, glutamatergic (GO:0051966) | 5 | 1 | 5.55E-02 | 1.00E+00 |
| protein monoubiquitination (GO:0006513) | 5 | 1 | 5.55E-02 | 1.00E+00 |
| telomere capping (GO:0016233) | 6 | 1 | 6.45E-02 | 1.00E+00 |
| histone ubiquitination (GO:0016574) | 6 | 1 | 6.45E-02 | 1.00E+00 |
| protein acetylation (GO:0006473) | 6 | 1 | 6.45E-02 | 1.00E+00 |
| isoprenoid biosynthetic process (GO:0008299) | 6 | 1 | 6.45E-02 | 1.00E+00 |
| cell fate commitment (GO:0045165) | 6 | 1 | 6.45E-02 | 1.00E+00 |
| negative regulation of molecular function (GO:0044092) | 13 | 2 | 8.54E-03 | 1.00E+00 |
| positive regulation of cytoskeleton organization (GO:0051495) | 7 | 1 | 7.34E-02 | 1.00E+00 |
| actin cytoskeleton reorganization (GO:0031532) | 7 | 1 | 7.34E-02 | 1.00E+00 |
| GPI anchor biosynthetic process (GO:0006506) | 7 | 1 | 7.34E-02 | 1.00E+00 |
| RNA modification (GO:0009451) | 8 | 1 | 8.22E-02 | 1.00E+00 |
| adenylate cyclase-inhibiting G-protein coupled receptor signaling pathway (GO:0007193) | 8 | 1 | 8.22E-02 | 1.00E+00 |
| establishment of organelle localization (GO:0051656) | 8 | 1 | 8.22E-02 | 1.00E+00 |
| chromatin silencing (GO:0006342) | 8 | 1 | 8.22E-02 | 1.00E+00 |
| negative regulation of catalytic activity (GO:0043086) | 8 | 1 | 8.22E-02 | 1.00E+00 |
| actin filament bundle assembly (GO:0051017) | 9 | 1 | 9.09E-02 | 1.00E+00 |
| positive regulation of nucleobase-containing compound metabolic process (GO:0045935) | 9 | 1 | 9.09E-02 | 1.00E+00 |
| negative regulation of nucleic acid-templated transcription (GO:1903507) | 19 | 2 | 1.65E-02 | 1.00E+00 |
| negative regulation of transcription, DNA-templated (GO:0045892) | 19 | 2 | 1.65E-02 | 1.00E+00 |
| negative regulation of RNA biosynthetic process (GO:1902679) | 19 | 2 | 1.65E-02 | 1.00E+00 |
| actin filament bundle organization (GO:0061572) | 10 | 1 | 9.95E-02 | 1.00E+00 |
| positive regulation of organelle organization (GO:0010638) | 10 | 1 | 9.95E-02 | 1.00E+00 |
| sterol metabolic process (GO:0016125) | 10 | 1 | 9.95E-02 | 1.00E+00 |
| negative regulation of cellular biosynthetic process (GO:0031327) | 20 | 2 | 1.80E-02 | 1.00E+00 |
| blood circulation (GO:0008015) | 11 | 1 | 1.08E-01 | 1.00E+00 |
| positive regulation of macromolecule metabolic process (GO:0010604) | 11 | 1 | 1.08E-01 | 1.00E+00 |
| lipid biosynthetic process (GO:0008610) | 11 | 1 | 1.08E-01 | 1.00E+00 |
| cAMP-mediated signaling (GO:0019933) | 11 | 1 | 1.08E-01 | 1.00E+00 |
| regulation of system process (GO:0044057) | 11 | 1 | 1.08E-01 | 1.00E+00 |
| synaptic transmission, glutamatergic (GO:0035249) | 12 | 1 | 1.17E-01 | 1.00E+00 |
| circulatory system process (GO:0003013) | 12 | 1 | 1.17E-01 | 1.00E+00 |
| regulation of organelle organization (GO:0033043) | 37 | 3 | 6.19E-03 | 1.00E+00 |
| protein acylation (GO:0043543) | 13 | 1 | 1.25E-01 | 1.00E+00 |
| maturation of SSU-rRNA (GO:0030490) | 13 | 1 | 1.25E-01 | 1.00E+00 |
| calcium-mediated signaling (GO:0019722) | 13 | 1 | 1.25E-01 | 1.00E+00 |
| organic hydroxy compound metabolic process (GO:1901615) | 13 | 1 | 1.25E-01 | 1.00E+00 |
| regulation of cytoskeleton organization (GO:0051493) | 14 | 1 | 1.33E-01 | 1.00E+00 |
| Rho protein signal transduction (GO:0007266) | 14 | 1 | 1.33E-01 | 1.00E+00 |
| inositol phosphate-mediated signaling (GO:0048016) | 14 | 1 | 1.33E-01 | 1.00E+00 |
| glycogen metabolic process (GO:0005977) | 14 | 1 | 1.33E-01 | 1.00E+00 |
| regulation of chromosome organization (GO:0033044) | 14 | 1 | 1.33E-01 | 1.00E+00 |
| cellular glucan metabolic process (GO:0006073) | 14 | 1 | 1.33E-01 | 1.00E+00 |
| negative regulation of metabolic process (GO:0009892) | 29 | 2 | 3.44E-02 | 1.00E+00 |
| negative regulation of biosynthetic process (GO:0009890) | 29 | 2 | 3.44E-02 | 1.00E+00 |
| cellular polysaccharide metabolic process (GO:0044264) | 15 | 1 | 1.41E-01 | 1.00E+00 |
| telomere maintenance (GO:0000723) | 15 | 1 | 1.41E-01 | 1.00E+00 |
| glutamate receptor signaling pathway (GO:0007215) | 16 | 1 | 1.50E-01 | 1.00E+00 |
| canonical Wnt signaling pathway (GO:0060070) | 16 | 1 | 1.50E-01 | 1.00E+00 |
| anatomical structure homeostasis (GO:0060249) | 16 | 1 | 1.50E-01 | 1.00E+00 |
| positive regulation of multicellular organismal process (GO:0051240) | 16 | 1 | 1.50E-01 | 1.00E+00 |
| regulation of adenylate cyclase activity (GO:0045761) | 33 | 2 | 4.29E-02 | 1.00E+00 |
| adenylate cyclase-modulating G-protein coupled receptor signaling pathway (GO:0007188) | 33 | 2 | 4.29E-02 | 1.00E+00 |
| regulation of cyclase activity (GO:0031279) | 34 | 2 | 4.52E-02 | 1.00E+00 |
| regulation of cAMP-mediated signaling (GO:0043949) | 35 | 2 | 4.75E-02 | 1.00E+00 |
| cellular response to hormone stimulus (GO:0032870) | 18 | 1 | 1.66E-01 | 1.00E+00 |
| ribosomal small subunit biogenesis (GO:0042274) | 18 | 1 | 1.66E-01 | 1.00E+00 |
| cellular modified amino acid metabolic process (GO:0006575) | 18 | 1 | 1.66E-01 | 1.00E+00 |
| negative regulation of biological process (GO:0048519) | 37 | 2 | 5.22E-02 | 1.00E+00 |
| positive regulation of cellular component organization (GO:0051130) | 19 | 1 | 1.74E-01 | 1.00E+00 |
| endocytosis (GO:0006897) | 58 | 3 | 1.96E-02 | 1.00E+00 |
| lipid metabolic process (GO:0006629) | 39 | 2 | 5.71E-02 | 1.00E+00 |
| neurotransmitter secretion (GO:0007269) | 20 | 1 | 1.81E-01 | 1.00E+00 |
| G-protein coupled receptor signaling pathway, coupled to cyclic nucleotide second messenger (GO:0007187) | 40 | 2 | 5.96E-02 | 1.00E+00 |
| actin filament polymerization (GO:0030041) | 20 | 1 | 1.81E-01 | 1.00E+00 |
| regulation of trans-synaptic signaling (GO:0099177) | 20 | 1 | 1.81E-01 | 1.00E+00 |
| modulation of chemical synaptic transmission (GO:0050804) | 20 | 1 | 1.81E-01 | 1.00E+00 |
| cyclic-nucleotide-mediated signaling (GO:0019935) | 20 | 1 | 1.81E-01 | 1.00E+00 |
| receptor-mediated endocytosis (GO:0006898) | 20 | 1 | 1.81E-01 | 1.00E+00 |
| lipoprotein biosynthetic process (GO:0042158) | 21 | 1 | 1.89E-01 | 1.00E+00 |
| protein lipidation (GO:0006497) | 21 | 1 | 1.89E-01 | 1.00E+00 |
| regulation of DNA metabolic process (GO:0051052) | 21 | 1 | 1.89E-01 | 1.00E+00 |
| second-messenger-mediated signaling (GO:0019932) | 42 | 2 | 6.47E-02 | 1.00E+00 |
| regulation of cellular component organization (GO:0051128) | 64 | 3 | 2.51E-02 | 1.00E+00 |
| proteolysis (GO:0006508) | 65 | 3 | 2.61E-02 | 1.00E+00 |
| regulation of signaling (GO:0023051) | 22 | 1 | 1.97E-01 | 1.00E+00 |
| response to hormone (GO:0009725) | 22 | 1 | 1.97E-01 | 1.00E+00 |
| negative regulation of intracellular signal transduction (GO:1902532) | 23 | 1 | 2.05E-01 | 1.00E+00 |
| organelle localization (GO:0051640) | 23 | 1 | 2.05E-01 | 1.00E+00 |
| protein targeting (GO:0006605) | 23 | 1 | 2.05E-01 | 1.00E+00 |
| peptidyl-lysine modification (GO:0018205) | 23 | 1 | 2.05E-01 | 1.00E+00 |
| activation of adenylate cyclase activity (GO:0007190) | 24 | 1 | 2.12E-01 | 1.00E+00 |
| adenylate cyclase-activating G-protein coupled receptor signaling pathway (GO:0007189) | 24 | 1 | 2.12E-01 | 1.00E+00 |
| actin polymerization or depolymerization (GO:0008154) | 24 | 1 | 2.12E-01 | 1.00E+00 |
| Ras protein signal transduction (GO:0007265) | 25 | 1 | 2.20E-01 | 1.00E+00 |
| cellular amino acid biosynthetic process (GO:0008652) | 25 | 1 | 2.20E-01 | 1.00E+00 |
| polysaccharide metabolic process (GO:0005976) | 25 | 1 | 2.20E-01 | 1.00E+00 |
| Wnt signaling pathway (GO:0016055) | 25 | 1 | 2.20E-01 | 1.00E+00 |
| lipid transport (GO:0006869) | 26 | 1 | 2.27E-01 | 1.00E+00 |
| cell-cell signaling by wnt (GO:0198738) | 26 | 1 | 2.27E-01 | 1.00E+00 |
| lipid localization (GO:0010876) | 27 | 1 | 2.34E-01 | 1.00E+00 |
| mitochondrion organization (GO:0007005) | 27 | 1 | 2.34E-01 | 1.00E+00 |
| membrane invagination (GO:0010324) | 28 | 1 | 2.42E-01 | 1.00E+00 |
| vesicle budding from membrane (GO:0006900) | 28 | 1 | 2.42E-01 | 1.00E+00 |
| regulation of catalytic activity (GO:0050790) | 56 | 2 | 1.04E-01 | 1.00E+00 |
| regulation of nucleobase-containing compound metabolic process (GO:0019219) | 58 | 2 | 1.10E-01 | 1.00E+00 |
| regulation of multicellular organismal process (GO:0051239) | 29 | 1 | 2.49E-01 | 1.00E+00 |
| protein metabolic process (GO:0019538) | 88 | 3 | 5.44E-02 | 1.00E+00 |
| small GTPase mediated signal transduction (GO:0007264) | 30 | 1 | 2.56E-01 | 1.00E+00 |
| tRNA metabolic process (GO:0006399) | 30 | 1 | 2.56E-01 | 1.00E+00 |
| positive regulation of signal transduction (GO:0009967) | 30 | 1 | 2.56E-01 | 1.00E+00 |
| regulation of molecular function (GO:0065009) | 92 | 3 | 6.04E-02 | 1.00E+00 |
| chromatin organization (GO:0006325) | 31 | 1 | 2.63E-01 | 1.00E+00 |
| positive regulation of response to stimulus (GO:0048584) | 31 | 1 | 2.63E-01 | 1.00E+00 |
| sensory perception (GO:0007600) | 32 | 1 | 2.70E-01 | 1.00E+00 |
| chemical synaptic transmission (GO:0007268) | 98 | 3 | 7.00E-02 | 1.00E+00 |
| anterograde trans-synaptic signaling (GO:0098916) | 98 | 3 | 7.00E-02 | 1.00E+00 |
| trans-synaptic signaling (GO:0099537) | 99 | 3 | 7.16E-02 | 1.00E+00 |
| synaptic signaling (GO:0099536) | 99 | 3 | 7.16E-02 | 1.00E+00 |
| nucleobase-containing compound metabolic process (GO:0006139) | 33 | 1 | 2.77E-01 | 1.00E+00 |
| regulation of macromolecule metabolic process (GO:0060255) | 33 | 1 | 2.77E-01 | 1.00E+00 |
| system process (GO:0003008) | 137 | 4 | 4.37E-02 | 1.00E+00 |
| cell differentiation (GO:0030154) | 70 | 2 | 1.48E-01 | 1.00E+00 |
| carboxylic acid biosynthetic process (GO:0046394) | 35 | 1 | 2.91E-01 | 1.00E+00 |
| cellular developmental process (GO:0048869) | 70 | 2 | 1.48E-01 | 1.00E+00 |
| organic acid biosynthetic process (GO:0016053) | 35 | 1 | 2.91E-01 | 1.00E+00 |
| positive regulation of transcription by RNA polymerase II (GO:0045944) | 72 | 2 | 1.55E-01 | 1.00E+00 |
| cell-cell signaling (GO:0007267) | 146 | 4 | 5.28E-02 | 1.00E+00 |
| cell communication (GO:0007154) | 146 | 4 | 5.28E-02 | 1.00E+00 |
| regulation of intracellular signal transduction (GO:1902531) | 74 | 2 | 1.62E-01 | 1.00E+00 |
| negative regulation of transcription by RNA polymerase II (GO:0000122) | 37 | 1 | 3.04E-01 | 1.00E+00 |
| organic substance biosynthetic process (GO:1901576) | 111 | 3 | 9.28E-02 | 1.00E+00 |
| nervous system process (GO:0050877) | 112 | 3 | 9.47E-02 | 1.00E+00 |
| primary metabolic process (GO:0044238) | 151 | 4 | 5.82E-02 | 1.00E+00 |
| organic cyclic compound metabolic process (GO:1901360) | 39 | 1 | 3.18E-01 | 1.00E+00 |
| actin filament organization (GO:0007015) | 39 | 1 | 3.18E-01 | 1.00E+00 |
| G-protein coupled receptor signaling pathway (GO:0007186) | 79 | 2 | 1.78E-01 | 1.00E+00 |
| organonitrogen compound metabolic process (GO:1901564) | 79 | 2 | 1.78E-01 | 1.00E+00 |
| negative regulation of signal transduction (GO:0009968) | 41 | 1 | 3.31E-01 | 1.00E+00 |
| regulation of transcription by RNA polymerase II (GO:0006357) | 207 | 5 | 4.91E-02 | 1.00E+00 |
| organonitrogen compound biosynthetic process (GO:1901566) | 42 | 1 | 3.37E-01 | 1.00E+00 |
| regulation of transcription, DNA-templated (GO:0006355) | 219 | 5 | 5.95E-02 | 1.00E+00 |
| regulation of nucleic acid-templated transcription (GO:1903506) | 219 | 5 | 5.95E-02 | 1.00E+00 |
| cell migration (GO:0016477) | 44 | 1 | 3.49E-01 | 1.00E+00 |
| negative regulation of response to stimulus (GO:0048585) | 44 | 1 | 3.49E-01 | 1.00E+00 |
| positive regulation of cellular process (GO:0048522) | 45 | 1 | 3.56E-01 | 1.00E+00 |
| regulation of RNA biosynthetic process (GO:2001141) | 233 | 5 | 7.33E-02 | 1.00E+00 |
| regulation of cell communication (GO:0010646) | 95 | 2 | 2.34E-01 | 1.00E+00 |
| regulation of signal transduction (GO:0009966) | 95 | 2 | 2.34E-01 | 1.00E+00 |
| regulation of biosynthetic process (GO:0009889) | 238 | 5 | 7.86E-02 | 1.00E+00 |
| regulation of cellular biosynthetic process (GO:0031326) | 238 | 5 | 7.86E-02 | 1.00E+00 |
| cellular amino acid metabolic process (GO:0006520) | 48 | 1 | 3.74E-01 | 1.00E+00 |
| multicellular organismal process (GO:0032501) | 291 | 6 | 6.01E-02 | 1.00E+00 |
| transcription by RNA polymerase II (GO:0006366) | 292 | 6 | 6.09E-02 | 1.00E+00 |
| ribosome biogenesis (GO:0042254) | 50 | 1 | 3.86E-01 | 1.00E+00 |
| transcription, DNA-templated (GO:0006351) | 359 | 7 | 8.27E-02 | 1.00E+00 |
| transmembrane receptor protein tyrosine kinase signaling pathway (GO:0007169) | 52 | 1 | 3.98E-01 | 1.00E+00 |
| protein localization to organelle (GO:0033365) | 52 | 1 | 3.98E-01 | 1.00E+00 |
| localization of cell (GO:0051674) | 52 | 1 | 3.98E-01 | 1.00E+00 |
| cell motility (GO:0048870) | 52 | 1 | 3.98E-01 | 1.00E+00 |
| vesicle-mediated transport (GO:0016192) | 161 | 3 | 2.02E-01 | 1.00E+00 |
| regulation of cellular process (GO:0050794) | 275 | 5 | 1.89E-01 | 1.00E+00 |
| peptidyl-amino acid modification (GO:0018193) | 55 | 1 | 4.15E-01 | 1.00E+00 |
| macromolecule localization (GO:0033036) | 57 | 1 | 4.26E-01 | 1.00E+00 |
| actin cytoskeleton organization (GO:0030036) | 58 | 1 | 4.31E-01 | 1.00E+00 |
| chromosome organization (GO:0051276) | 58 | 1 | 4.31E-01 | 1.00E+00 |
| RNA metabolic process (GO:0016070) | 117 | 2 | 3.12E-01 | 1.00E+00 |
| cellular localization (GO:0051641) | 294 | 5 | 2.05E-01 | 1.00E+00 |
| ribonucleoprotein complex biogenesis (GO:0022613) | 59 | 1 | 4.37E-01 | 1.00E+00 |
| regulation of metabolic process (GO:0019222) | 432 | 7 | 1.90E-01 | 1.00E+00 |
| signal transduction (GO:0007165) | 438 | 7 | 1.93E-01 | 1.00E+00 |
| regulation of biological process (GO:0050789) | 755 | 12 | 6.89E-02 | 1.00E+00 |
| carbohydrate metabolic process (GO:0005975) | 63 | 1 | 4.58E-01 | 1.00E+00 |
| cellular protein localization (GO:0034613) | 256 | 4 | 3.10E-01 | 1.00E+00 |
| response to organic substance (GO:0010033) | 64 | 1 | 4.63E-01 | 1.00E+00 |
| cellular macromolecule localization (GO:0070727) | 258 | 4 | 3.13E-01 | 1.00E+00 |
| enzyme linked receptor protein signaling pathway (GO:0007167) | 65 | 1 | 4.68E-01 | 1.00E+00 |
| ncRNA metabolic process (GO:0034660) | 65 | 1 | 4.68E-01 | 1.00E+00 |
| intracellular signal transduction (GO:0035556) | 198 | 3 | 4.39E-01 | 1.00E+00 |
| biological regulation (GO:0065007) | 881 | 13 | 9.13E-02 | 1.00E+00 |
| regulation of response to stimulus (GO:0048583) | 68 | 1 | 4.84E-01 | 1.00E+00 |
| cellular protein modification process (GO:0006464) | 274 | 4 | 3.35E-01 | 1.00E+00 |
| intracellular protein transport (GO:0006886) | 208 | 3 | 4.52E-01 | 1.00E+00 |
| metabolic process (GO:0008152) | 1369 | 19 | 7.72E-02 | 1.00E+00 |
| cellular protein metabolic process (GO:0044267) | 297 | 4 | 5.31E-01 | 1.00E+00 |
| cellular macromolecule metabolic process (GO:0044260) | 297 | 4 | 5.31E-01 | 1.00E+00 |
| protein ubiquitination (GO:0016567) | 75 | 1 | 5.17E-01 | 1.00E+00 |
| positive regulation of biological process (GO:0048518) | 75 | 1 | 5.17E-01 | 1.00E+00 |
| cytoskeleton organization (GO:0007010) | 75 | 1 | 5.17E-01 | 1.00E+00 |
| regulation of cellular metabolic process (GO:0031323) | 150 | 2 | 6.55E-01 | 1.00E+00 |
| organic substance metabolic process (GO:0071704) | 1145 | 15 | 1.74E-01 | 1.00E+00 |
| response to stimulus (GO:0050896) | 230 | 3 | 4.84E-01 | 1.00E+00 |
| membrane organization (GO:0061024) | 78 | 1 | 5.31E-01 | 1.00E+00 |
| cellular component organization (GO:0016043) | 474 | 6 | 4.56E-01 | 1.00E+00 |
| cellular response to stimulus (GO:0051716) | 559 | 7 | 4.88E-01 | 1.00E+00 |
| protein-containing complex subunit organization (GO:0043933) | 80 | 1 | 5.40E-01 | 1.00E+00 |
| organelle organization (GO:0006996) | 242 | 3 | 5.04E-01 | 1.00E+00 |
| cellular component organization or biogenesis (GO:0071840) | 81 | 1 | 5.44E-01 | 1.00E+00 |
| protein modification by small protein conjugation or removal (GO:0070647) | 81 | 1 | 5.44E-01 | 1.00E+00 |
| protein modification by small protein conjugation (GO:0032446) | 81 | 1 | 5.44E-01 | 1.00E+00 |
| cellular component biogenesis (GO:0044085) | 81 | 1 | 5.44E-01 | 1.00E+00 |
| gene expression (GO:0010467) | 581 | 7 | 4.98E-01 | 1.00E+00 |
| nucleic acid metabolic process (GO:0090304) | 169 | 2 | 6.77E-01 | 1.00E+00 |
| cell surface receptor signaling pathway (GO:0007166) | 176 | 2 | 6.86E-01 | 1.00E+00 |
| nervous system development (GO:0007399) | 88 | 1 | 5.74E-01 | 1.00E+00 |
| macromolecule metabolic process (GO:0043170) | 818 | 9 | 6.96E-01 | 1.00E+00 |
| cellular process (GO:0009987) | 1754 | 18 | 7.67E-01 | 1.00E+00 |
| regulation of biological quality (GO:0065008) | 195 | 2 | 7.10E-01 | 1.00E+00 |
| localization (GO:0051179) | 597 | 6 | 8.24E-01 | 1.00E+00 |
| homeostatic process (GO:0042592) | 101 | 1 | 6.25E-01 | 1.00E+00 |
| Unclassified (UNCLASSIFIED) | 2556 | 23 | 7.82E-01 | 1.00E+00 |
| establishment of localization (GO:0051234) | 346 | 3 | 1.00E+00 | 1.00E+00 |
| transport (GO:0006810) | 346 | 3 | 1.00E+00 | 1.00E+00 |
| system development (GO:0048731) | 124 | 1 | 1.00E+00 | 1.00E+00 |
| response to chemical (GO:0042221) | 134 | 1 | 1.00E+00 | 1.00E+00 |
| cellular metabolic process (GO:0044237) | 567 | 4 | 6.53E-01 | 1.00E+00 |
| multicellular organism development (GO:0007275) | 151 | 1 | 1.00E+00 | 1.00E+00 |
| cell cycle (GO:0007049) | 188 | 1 | 1.00E+00 | 1.00E+00 |

Table S46. GO analysis in Rheiformes in the 4-times hypothesis using PANTHER GO-Slim Cellular Component. P values are calculated by Fisher’s exact test and FDR values are calculated by the Benjamini–Hochberg procedure.

| GO term | Number of genes in reference | Number of genes in query | P-values | FDR values |
| --- | --- | --- | --- | --- |
| integral component of mitochondrial outer membrane (GO:0031307) | 2 | 1 | 2.82E-02 | 1.00E+00 |
| DNA-directed RNA polymerase III complex (GO:0005666) | 7 | 1 | 7.34E-02 | 1.00E+00 |
| PcG protein complex (GO:0031519) | 7 | 1 | 7.34E-02 | 1.00E+00 |
| mitochondrial outer membrane (GO:0005741) | 10 | 1 | 9.95E-02 | 1.00E+00 |
| organelle outer membrane (GO:0031968) | 11 | 1 | 1.08E-01 | 1.00E+00 |
| integral component of Golgi membrane (GO:0030173) | 17 | 1 | 1.58E-01 | 1.00E+00 |
| nuclear transcription factor complex (GO:0044798) | 19 | 1 | 1.74E-01 | 1.00E+00 |
| ubiquitin ligase complex (GO:0000151) | 35 | 1 | 2.91E-01 | 1.00E+00 |
| Golgi membrane (GO:0000139) | 41 | 1 | 3.31E-01 | 1.00E+00 |
| nuclear DNA-directed RNA polymerase complex (GO:0055029) | 44 | 1 | 3.49E-01 | 1.00E+00 |
| organelle envelope (GO:0031967) | 46 | 1 | 3.62E-01 | 1.00E+00 |
| nuclear chromatin (GO:0000790) | 98 | 2 | 2.45E-01 | 1.00E+00 |
| nucleoplasm (GO:0005654) | 99 | 2 | 2.48E-01 | 1.00E+00 |
| integral component of plasma membrane (GO:0005887) | 213 | 4 | 1.49E-01 | 1.00E+00 |
| chromatin (GO:0000785) | 109 | 2 | 2.83E-01 | 1.00E+00 |
| Golgi subcompartment (GO:0098791) | 58 | 1 | 4.31E-01 | 1.00E+00 |
| nuclear part (GO:0044428) | 303 | 5 | 2.14E-01 | 1.00E+00 |
| receptor complex (GO:0043235) | 64 | 1 | 4.63E-01 | 1.00E+00 |
| chromosomal part (GO:0044427) | 133 | 2 | 3.67E-01 | 1.00E+00 |
| actin cytoskeleton (GO:0015629) | 67 | 1 | 4.79E-01 | 1.00E+00 |
| integral component of membrane (GO:0016021) | 268 | 4 | 3.26E-01 | 1.00E+00 |
| intrinsic component of membrane (GO:0031224) | 269 | 4 | 3.28E-01 | 1.00E+00 |
| chromosome (GO:0005694) | 146 | 2 | 6.51E-01 | 1.00E+00 |
| cytosol (GO:0005829) | 234 | 3 | 4.91E-01 | 1.00E+00 |
| Unclassified (UNCLASSIFIED) | 2890 | 37 | 1.23E-02 | 1.00E+00 |
| membrane part (GO:0044425) | 314 | 4 | 5.43E-01 | 1.00E+00 |
| membrane (GO:0016020) | 318 | 4 | 5.46E-01 | 1.00E+00 |
| endoplasmic reticulum (GO:0005783) | 80 | 1 | 5.40E-01 | 1.00E+00 |
| nuclear lumen (GO:0031981) | 186 | 2 | 6.99E-01 | 1.00E+00 |
| organelle subcompartment (GO:0031984) | 95 | 1 | 6.02E-01 | 1.00E+00 |
| membrane-bounded organelle (GO:0043227) | 192 | 2 | 7.06E-01 | 1.00E+00 |
| nucleus (GO:0005634) | 696 | 7 | 8.35E-01 | 1.00E+00 |
| intracellular membrane-bounded organelle (GO:0043231) | 716 | 7 | 8.39E-01 | 1.00E+00 |
| intracellular organelle (GO:0043229) | 717 | 7 | 8.39E-01 | 1.00E+00 |
| intracellular non-membrane-bounded organelle (GO:0043232) | 342 | 3 | 1.00E+00 | 1.00E+00 |
| non-membrane-bounded organelle (GO:0043228) | 342 | 3 | 1.00E+00 | 1.00E+00 |
| organelle (GO:0043226) | 1177 | 10 | 8.66E-01 | 1.00E+00 |
| cytoplasmic part (GO:0044444) | 715 | 5 | 6.78E-01 | 1.00E+00 |
| intracellular (GO:0005622) | 1347 | 9 | 2.60E-01 | 1.00E+00 |
| intracellular part (GO:0044424) | 1199 | 7 | 1.78E-01 | 1.00E+00 |
| cytoskeleton (GO:0005856) | 178 | 1 | 1.00E+00 | 1.00E+00 |
| vacuole (GO:0005773) | 358 | 2 | 5.81E-01 | 1.00E+00 |
| cell part (GO:0044464) | 1620 | 9 | 6.68E-02 | 1.00E+00 |
| cell (GO:0005623) | 1627 | 9 | 4.91E-02 | 1.00E+00 |
| cytoplasm (GO:0005737) | 1127 | 6 | 1.22E-01 | 1.00E+00 |
| endomembrane system (GO:0012505) | 226 | 1 | 7.24E-01 | 1.00E+00 |
| protein-containing complex (GO:0032991) | 501 | 2 | 2.31E-01 | 1.00E+00 |
| plasma membrane (GO:0005886) | 623 | 2 | 1.20E-01 | 1.00E+00 |
| cell periphery (GO:0071944) | 635 | 2 | 8.33E-02 | 1.00E+00 |

Table S47. GO analysis in Rheiformes in the 4-times hypothesis using PANTHER GO-Slim Molecular Function. P values are calculated by Fisher’s exact test and FDR values are calculated by the Benjamini–Hochberg procedure.

| GO term | Number of genes in reference | Number of genes in query | P-values | FDR values |
| --- | --- | --- | --- | --- |
| transferase activity, transferring pentosyl groups (GO:0016763) | 4 | 1 | 4.65E-02 | 1.00E+00 |
| carbohydrate derivative transmembrane transporter activity (GO:1901505) | 6 | 1 | 6.45E-02 | 1.00E+00 |
| RNA polymerase III activity (GO:0001056) | 7 | 1 | 7.34E-02 | 1.00E+00 |
| ATP-dependent helicase activity (GO:0008026) | 9 | 1 | 9.09E-02 | 1.00E+00 |
| nucleobase-containing compound transmembrane transporter activity (GO:0015932) | 9 | 1 | 9.09E-02 | 1.00E+00 |
| purine NTP-dependent helicase activity (GO:0070035) | 9 | 1 | 9.09E-02 | 1.00E+00 |
| lipid binding (GO:0008289) | 9 | 1 | 9.09E-02 | 1.00E+00 |
| oxidoreductase activity, acting on the CH-OH group of donors, NAD or NADP as acceptor (GO:0016616) | 19 | 2 | 1.65E-02 | 1.00E+00 |
| monocarboxylic acid transmembrane transporter activity (GO:0008028) | 10 | 1 | 9.95E-02 | 1.00E+00 |
| oxidoreductase activity, acting on CH-OH group of donors (GO:0016614) | 20 | 2 | 1.80E-02 | 1.00E+00 |
| helicase activity (GO:0004386) | 10 | 1 | 9.95E-02 | 1.00E+00 |
| oxidoreductase activity, acting on paired donors, with incorporation or reduction of molecular oxygen, 2-oxoglutarate as one donor, and incorporation of one atom each of oxygen into both donors (GO:0016706) | 11 | 1 | 1.08E-01 | 1.00E+00 |
| carboxylic acid transmembrane transporter activity (GO:0046943) | 36 | 3 | 5.77E-03 | 1.00E+00 |
| G-protein coupled peptide receptor activity (GO:0008528) | 14 | 1 | 1.33E-01 | 1.00E+00 |
| organic anion transmembrane transporter activity (GO:0008514) | 42 | 3 | 8.60E-03 | 1.00E+00 |
| anion transmembrane transporter activity (GO:0008509) | 43 | 3 | 9.14E-03 | 1.00E+00 |
| phosphatase regulator activity (GO:0019208) | 15 | 1 | 1.41E-01 | 1.00E+00 |
| glutamate receptor activity (GO:0008066) | 17 | 1 | 1.58E-01 | 1.00E+00 |
| DNA-directed 5'-3' RNA polymerase activity (GO:0003899) | 17 | 1 | 1.58E-01 | 1.00E+00 |
| 5'-3' RNA polymerase activity (GO:0034062) | 17 | 1 | 1.58E-01 | 1.00E+00 |
| glutamate binding (GO:0016595) | 17 | 1 | 1.58E-01 | 1.00E+00 |
| RNA polymerase activity (GO:0097747) | 17 | 1 | 1.58E-01 | 1.00E+00 |
| adenylate cyclase activity (GO:0004016) | 34 | 2 | 4.52E-02 | 1.00E+00 |
| transmembrane receptor protein kinase activity (GO:0019199) | 18 | 1 | 1.66E-01 | 1.00E+00 |
| transmembrane receptor protein tyrosine kinase activity (GO:0004714) | 18 | 1 | 1.66E-01 | 1.00E+00 |
| N-acetyltransferase activity (GO:0008080) | 18 | 1 | 1.66E-01 | 1.00E+00 |
| phosphorus-oxygen lyase activity (GO:0016849) | 37 | 2 | 5.22E-02 | 1.00E+00 |
| oxidoreductase activity, acting on paired donors, with incorporation or reduction of molecular oxygen (GO:0016705) | 19 | 1 | 1.74E-01 | 1.00E+00 |
| amino acid transmembrane transporter activity (GO:0015171) | 21 | 1 | 1.89E-01 | 1.00E+00 |
| cytokine activity (GO:0005125) | 21 | 1 | 1.89E-01 | 1.00E+00 |
| peptidase inhibitor activity (GO:0030414) | 21 | 1 | 1.89E-01 | 1.00E+00 |
| N-acyltransferase activity (GO:0016410) | 21 | 1 | 1.89E-01 | 1.00E+00 |
| amino acid binding (GO:0016597) | 22 | 1 | 1.97E-01 | 1.00E+00 |
| peptidase regulator activity (GO:0061134) | 22 | 1 | 1.97E-01 | 1.00E+00 |
| GTP binding (GO:0005525) | 29 | 1 | 2.49E-01 | 1.00E+00 |
| chromatin binding (GO:0003682) | 58 | 2 | 1.10E-01 | 1.00E+00 |
| purine ribonucleoside binding (GO:0032550) | 29 | 1 | 2.49E-01 | 1.00E+00 |
| ribonucleoside binding (GO:0032549) | 29 | 1 | 2.49E-01 | 1.00E+00 |
| nucleoside binding (GO:0001882) | 29 | 1 | 2.49E-01 | 1.00E+00 |
| carboxylic acid binding (GO:0031406) | 29 | 1 | 2.49E-01 | 1.00E+00 |
| proximal promoter sequence-specific DNA binding (GO:0000987) | 30 | 1 | 2.56E-01 | 1.00E+00 |
| RNA polymerase II proximal promoter sequence-specific DNA binding (GO:0000978) | 30 | 1 | 2.56E-01 | 1.00E+00 |
| organic acid binding (GO:0043177) | 31 | 1 | 2.63E-01 | 1.00E+00 |
| guanyl-nucleotide exchange factor activity (GO:0005085) | 33 | 1 | 2.77E-01 | 1.00E+00 |
| G-protein coupled receptor activity (GO:0004930) | 107 | 3 | 8.55E-02 | 1.00E+00 |
| peptide binding (GO:0042277) | 36 | 1 | 2.98E-01 | 1.00E+00 |
| catalytic activity, acting on RNA (GO:0140098) | 73 | 2 | 1.58E-01 | 1.00E+00 |
| amide binding (GO:0033218) | 41 | 1 | 3.31E-01 | 1.00E+00 |
| lyase activity (GO:0016829) | 84 | 2 | 1.96E-01 | 1.00E+00 |
| receptor ligand activity (GO:0048018) | 45 | 1 | 3.56E-01 | 1.00E+00 |
| calcium ion binding (GO:0005509) | 46 | 1 | 3.62E-01 | 1.00E+00 |
| ubiquitin-protein transferase activity (GO:0004842) | 93 | 2 | 2.27E-01 | 1.00E+00 |
| receptor regulator activity (GO:0030545) | 47 | 1 | 3.68E-01 | 1.00E+00 |
| enzyme regulator activity (GO:0030234) | 190 | 4 | 1.10E-01 | 1.00E+00 |
| transferase activity, transferring acyl groups other than amino-acyl groups (GO:0016747) | 48 | 1 | 3.74E-01 | 1.00E+00 |
| GTPase activity (GO:0003924) | 97 | 2 | 2.41E-01 | 1.00E+00 |
| transcription regulator activity (GO:0140110) | 257 | 5 | 1.01E-01 | 1.00E+00 |
| transmembrane signaling receptor activity (GO:0004888) | 157 | 3 | 1.93E-01 | 1.00E+00 |
| ion transmembrane transporter activity (GO:0015075) | 158 | 3 | 1.95E-01 | 1.00E+00 |
| DNA-binding transcription factor activity (GO:0003700) | 212 | 4 | 1.47E-01 | 1.00E+00 |
| transcription factor binding (GO:0008134) | 55 | 1 | 4.15E-01 | 1.00E+00 |
| ligase activity (GO:0016874) | 111 | 2 | 2.90E-01 | 1.00E+00 |
| organic cyclic compound binding (GO:0097159) | 57 | 1 | 4.26E-01 | 1.00E+00 |
| ubiquitin-like protein transferase activity (GO:0019787) | 115 | 2 | 3.05E-01 | 1.00E+00 |
| transferase activity, transferring glycosyl groups (GO:0016757) | 58 | 1 | 4.31E-01 | 1.00E+00 |
| RNA polymerase II transcription factor activity, sequence-specific DNA binding (GO:0000981) | 59 | 1 | 4.37E-01 | 1.00E+00 |
| oxidoreductase activity (GO:0016491) | 177 | 3 | 2.42E-01 | 1.00E+00 |
| transmembrane transporter activity (GO:0022857) | 238 | 4 | 2.90E-01 | 1.00E+00 |
| catalytic activity, acting on a protein (GO:0140096) | 60 | 1 | 4.42E-01 | 1.00E+00 |
| molecular function regulator (GO:0098772) | 243 | 4 | 2.95E-01 | 1.00E+00 |
| transferase activity, transferring acyl groups (GO:0016746) | 63 | 1 | 4.58E-01 | 1.00E+00 |
| signaling receptor activity (GO:0038023) | 201 | 3 | 4.43E-01 | 1.00E+00 |
| GTPase binding (GO:0051020) | 68 | 1 | 4.84E-01 | 1.00E+00 |
| nucleoside-triphosphatase activity (GO:0017111) | 210 | 3 | 4.55E-01 | 1.00E+00 |
| pyrophosphatase activity (GO:0016462) | 216 | 3 | 4.63E-01 | 1.00E+00 |
| hydrolase activity, acting on acid anhydrides, in phosphorus-containing anhydrides (GO:0016818) | 216 | 3 | 4.63E-01 | 1.00E+00 |
| hydrolase activity, acting on acid anhydrides (GO:0016817) | 216 | 3 | 4.63E-01 | 1.00E+00 |
| transporter activity (GO:0005215) | 292 | 4 | 3.63E-01 | 1.00E+00 |
| transcription coregulator activity (GO:0003712) | 73 | 1 | 5.08E-01 | 1.00E+00 |
| molecular transducer activity (GO:0060089) | 225 | 3 | 4.77E-01 | 1.00E+00 |
| metal ion binding (GO:0046872) | 75 | 1 | 5.17E-01 | 1.00E+00 |
| transcription regulatory region sequence-specific DNA binding (GO:0000976) | 78 | 1 | 5.31E-01 | 1.00E+00 |
| small molecule binding (GO:0036094) | 91 | 1 | 5.86E-01 | 1.00E+00 |
| cation binding (GO:0043169) | 95 | 1 | 6.02E-01 | 1.00E+00 |
| binding (GO:0005488) | 1436 | 15 | 7.53E-01 | 1.00E+00 |
| RNA binding (GO:0003723) | 195 | 2 | 7.10E-01 | 1.00E+00 |
| protein binding (GO:0005515) | 690 | 7 | 8.34E-01 | 1.00E+00 |
| transcription regulatory region DNA binding (GO:0044212) | 100 | 1 | 6.21E-01 | 1.00E+00 |
| catalytic activity (GO:0003824) | 1504 | 14 | 1.00E+00 | 1.00E+00 |
| cytoskeletal protein binding (GO:0008092) | 114 | 1 | 1.00E+00 | 1.00E+00 |
| transferase activity (GO:0016740) | 590 | 5 | 1.00E+00 | 1.00E+00 |
| Unclassified (UNCLASSIFIED) | 2686 | 22 | 3.36E-01 | 1.00E+00 |
| nucleic acid binding (GO:0003676) | 532 | 4 | 8.15E-01 | 1.00E+00 |
| heterocyclic compound binding (GO:1901363) | 556 | 4 | 8.17E-01 | 1.00E+00 |
| DNA binding (GO:0003677) | 302 | 2 | 1.00E+00 | 1.00E+00 |
| protein kinase activity (GO:0004672) | 162 | 1 | 1.00E+00 | 1.00E+00 |
| ion binding (GO:0043167) | 166 | 1 | 1.00E+00 | 1.00E+00 |
| enzyme binding (GO:0019899) | 187 | 1 | 1.00E+00 | 1.00E+00 |
| phosphotransferase activity, alcohol group as acceptor (GO:0016773) | 199 | 1 | 1.00E+00 | 1.00E+00 |
| hydrolase activity (GO:0016787) | 607 | 3 | 2.72E-01 | 1.00E+00 |
| transferase activity, transferring phosphorus-containing groups (GO:0016772) | 266 | 1 | 5.18E-01 | 1.00E+00 |

Table S48. Pathway analysis for Rheiformes in the 4-times hypothesis using PANTHER pathway. P values are calculated by Fisher’s exact test and FDR values are calculated by the Benjamini–Hochberg procedure.

| GO term | Number of genes in reference | Number of genes in query | P-values | FDR values |
| --- | --- | --- | --- | --- |
| Cholesterol biosynthesis (P00014) | 7 | 1 | 7.34E-02 | 1.00E+00 |
| Metabotropic glutamate receptor group II pathway (P00040) | 9 | 1 | 9.09E-02 | 1.00E+00 |
| Axon guidance mediated by netrin (P00009) | 13 | 1 | 1.25E-01 | 1.00E+00 |
| Ionotropic glutamate receptor pathway (P00037) | 21 | 1 | 1.89E-01 | 1.00E+00 |
| B cell activation (P00010) | 21 | 1 | 1.89E-01 | 1.00E+00 |
| T cell activation (P00053) | 24 | 1 | 2.12E-01 | 1.00E+00 |
| Interleukin signaling pathway (P00036) | 29 | 1 | 2.49E-01 | 1.00E+00 |
| Heterotrimeric G-protein signaling pathway-Gq alpha and Go alpha mediated pathway (P00027) | 34 | 1 | 2.84E-01 | 1.00E+00 |
| Heterotrimeric G-protein signaling pathway-Gi alpha and Gs alpha mediated pathway (P00026) | 55 | 1 | 4.15E-01 | 1.00E+00 |
| Inflammation mediated by chemokine and cytokine signaling pathway (P00031) | 60 | 1 | 4.42E-01 | 1.00E+00 |
| Gonadotropin-releasing hormone receptor pathway (P06664) | 71 | 1 | 4.98E-01 | 1.00E+00 |
| Wnt signaling pathway (P00057) | 72 | 1 | 5.03E-01 | 1.00E+00 |
| Unclassified (UNCLASSIFIED) | 4758 | 49 | 2.32E-01 | 1.00E+00 |

Table S49. GO analysis in Casuariiformes in the 4-times hypothesis using PANTHER GO-Slim Biological Process. P values are calculated by Fisher’s exact test and FDR values are calculated by the Benjamini–Hochberg procedure.

| GO term | Number of genes in reference | Number of genes in query | P-values | FDR values |
| --- | --- | --- | --- | --- |
| regulation of leukocyte migration (GO:0002685) | 3 | 1 | 1.78E-02 | 1.00E+00 |
| negative regulation of cell migration (GO:0030336) | 3 | 1 | 1.78E-02 | 1.00E+00 |
| regulation of immune system process (GO:0002682) | 5 | 1 | 2.67E-02 | 1.00E+00 |
| positive regulation of MAP kinase activity (GO:0043406) | 5 | 1 | 2.67E-02 | 1.00E+00 |
| positive regulation of ERK1 and ERK2 cascade (GO:0070374) | 5 | 1 | 2.67E-02 | 1.00E+00 |
| leukocyte migration (GO:0050900) | 6 | 1 | 3.10E-02 | 1.00E+00 |
| cellular protein-containing complex localization (GO:0034629) | 6 | 1 | 3.10E-02 | 1.00E+00 |
| cellular response to cytokine stimulus (GO:0071345) | 12 | 2 | 1.70E-03 | 1.00E+00 |
| histone modification (GO:0016570) | 7 | 1 | 3.54E-02 | 1.00E+00 |
| protein transport (GO:0015031) | 7 | 1 | 3.54E-02 | 1.00E+00 |
| GPI anchor biosynthetic process (GO:0006506) | 7 | 1 | 3.54E-02 | 1.00E+00 |
| establishment of protein localization (GO:0045184) | 8 | 1 | 3.97E-02 | 1.00E+00 |
| protein autophosphorylation (GO:0046777) | 9 | 1 | 4.40E-02 | 1.00E+00 |
| positive regulation of MAPK cascade (GO:0043410) | 9 | 1 | 4.40E-02 | 1.00E+00 |
| positive regulation of cell migration (GO:0030335) | 10 | 1 | 4.83E-02 | 1.00E+00 |
| regulation of cell migration (GO:0030334) | 20 | 2 | 4.23E-03 | 1.00E+00 |
| response to cytokine (GO:0034097) | 20 | 2 | 4.23E-03 | 1.00E+00 |
| cytokine-mediated signaling pathway (GO:0019221) | 11 | 1 | 5.26E-02 | 1.00E+00 |
| positive regulation of protein serine/threonine kinase activity (GO:0071902) | 11 | 1 | 5.26E-02 | 1.00E+00 |
| regulation of cell motility (GO:2000145) | 22 | 2 | 5.03E-03 | 1.00E+00 |
| regulation of cellular component movement (GO:0051270) | 24 | 2 | 5.89E-03 | 1.00E+00 |
| extracellular matrix organization (GO:0030198) | 13 | 1 | 6.11E-02 | 1.00E+00 |
| positive regulation of neuron differentiation (GO:0045666) | 14 | 1 | 6.54E-02 | 1.00E+00 |
| extracellular structure organization (GO:0043062) | 14 | 1 | 6.54E-02 | 1.00E+00 |
| positive regulation of neurogenesis (GO:0050769) | 15 | 1 | 6.96E-02 | 1.00E+00 |
| regulation of MAPK cascade (GO:0043408) | 16 | 1 | 7.38E-02 | 1.00E+00 |
| positive regulation of cell development (GO:0010720) | 17 | 1 | 7.79E-02 | 1.00E+00 |
| regulation of protein serine/threonine kinase activity (GO:0071900) | 18 | 1 | 8.21E-02 | 1.00E+00 |
| positive regulation of cell proliferation (GO:0008284) | 19 | 1 | 8.62E-02 | 1.00E+00 |
| positive regulation of cell differentiation (GO:0045597) | 19 | 1 | 8.62E-02 | 1.00E+00 |
| regulation of protein kinase activity (GO:0045859) | 19 | 1 | 8.62E-02 | 1.00E+00 |
| cytoplasmic translation (GO:0002181) | 19 | 1 | 8.62E-02 | 1.00E+00 |
| regulation of protein phosphorylation (GO:0001932) | 20 | 1 | 9.03E-02 | 1.00E+00 |
| lipoprotein biosynthetic process (GO:0042158) | 21 | 1 | 9.44E-02 | 1.00E+00 |
| protein lipidation (GO:0006497) | 21 | 1 | 9.44E-02 | 1.00E+00 |
| cell migration (GO:0016477) | 44 | 2 | 1.78E-02 | 1.00E+00 |
| cell proliferation (GO:0008283) | 23 | 1 | 1.03E-01 | 1.00E+00 |
| recombinational repair (GO:0000725) | 23 | 1 | 1.03E-01 | 1.00E+00 |
| double-strand break repair via homologous recombination (GO:0000724) | 23 | 1 | 1.03E-01 | 1.00E+00 |
| microtubule-based movement (GO:0007018) | 24 | 1 | 1.07E-01 | 1.00E+00 |
| regulation of cell proliferation (GO:0042127) | 26 | 1 | 1.15E-01 | 1.00E+00 |
| localization of cell (GO:0051674) | 52 | 2 | 2.40E-02 | 1.00E+00 |
| cell motility (GO:0048870) | 52 | 2 | 2.40E-02 | 1.00E+00 |
| protein localization (GO:0008104) | 27 | 1 | 1.19E-01 | 1.00E+00 |
| regulation of cell differentiation (GO:0045595) | 28 | 1 | 1.23E-01 | 1.00E+00 |
| cation transport (GO:0006812) | 31 | 1 | 1.34E-01 | 1.00E+00 |
| vesicle fusion to plasma membrane (GO:0099500) | 32 | 1 | 1.38E-01 | 1.00E+00 |
| response to organic substance (GO:0010033) | 64 | 2 | 3.49E-02 | 1.00E+00 |
| regulation of localization (GO:0032879) | 65 | 2 | 3.58E-02 | 1.00E+00 |
| plasma membrane fusion (GO:0045026) | 33 | 1 | 1.42E-01 | 1.00E+00 |
| cell development (GO:0048468) | 34 | 1 | 1.46E-01 | 1.00E+00 |
| organic substance transport (GO:0071702) | 36 | 1 | 1.54E-01 | 1.00E+00 |
| regulation of developmental process (GO:0050793) | 38 | 1 | 1.61E-01 | 1.00E+00 |
| membrane fusion (GO:0061025) | 39 | 1 | 1.65E-01 | 1.00E+00 |
| organonitrogen compound biosynthetic process (GO:1901566) | 42 | 1 | 1.77E-01 | 1.00E+00 |
| ion transport (GO:0006811) | 48 | 1 | 1.99E-01 | 1.00E+00 |
| transmembrane receptor protein tyrosine kinase signaling pathway (GO:0007169) | 52 | 1 | 2.13E-01 | 1.00E+00 |
| peptidyl-amino acid modification (GO:0018193) | 55 | 1 | 2.24E-01 | 1.00E+00 |
| macromolecule localization (GO:0033036) | 57 | 1 | 2.31E-01 | 1.00E+00 |
| neuron differentiation (GO:0030182) | 58 | 1 | 2.34E-01 | 1.00E+00 |
| regulation of phosphorylation (GO:0042325) | 60 | 1 | 2.41E-01 | 1.00E+00 |
| generation of neurons (GO:0048699) | 65 | 1 | 2.58E-01 | 1.00E+00 |
| enzyme linked receptor protein signaling pathway (GO:0007167) | 65 | 1 | 2.58E-01 | 1.00E+00 |
| response to chemical (GO:0042221) | 134 | 2 | 1.24E-01 | 1.00E+00 |
| regulation of phosphorus metabolic process (GO:0051174) | 68 | 1 | 2.68E-01 | 1.00E+00 |
| regulation of phosphate metabolic process (GO:0019220) | 68 | 1 | 2.68E-01 | 1.00E+00 |
| cell differentiation (GO:0030154) | 70 | 1 | 2.75E-01 | 1.00E+00 |
| cellular developmental process (GO:0048869) | 70 | 1 | 2.75E-01 | 1.00E+00 |
| microtubule-based process (GO:0007017) | 70 | 1 | 2.75E-01 | 1.00E+00 |
| positive regulation of transcription by RNA polymerase II (GO:0045944) | 72 | 1 | 2.82E-01 | 1.00E+00 |
| neurogenesis (GO:0022008) | 72 | 1 | 2.82E-01 | 1.00E+00 |
| regulation of intracellular signal transduction (GO:1902531) | 74 | 1 | 2.88E-01 | 1.00E+00 |
| DNA repair (GO:0006281) | 76 | 1 | 2.95E-01 | 1.00E+00 |
| mitotic nuclear division (GO:0140014) | 78 | 1 | 3.01E-01 | 1.00E+00 |
| membrane organization (GO:0061024) | 78 | 1 | 3.01E-01 | 1.00E+00 |
| mitotic cell cycle (GO:0000278) | 81 | 1 | 3.10E-01 | 1.00E+00 |
| mitotic cell cycle process (GO:1903047) | 81 | 1 | 3.10E-01 | 1.00E+00 |
| nervous system development (GO:0007399) | 88 | 1 | 3.32E-01 | 1.00E+00 |
| formation of translation initiation ternary complex (GO:0001677) | 93 | 1 | 3.47E-01 | 1.00E+00 |
| translational termination (GO:0006415) | 93 | 1 | 3.47E-01 | 1.00E+00 |
| translational elongation (GO:0006414) | 93 | 1 | 3.47E-01 | 1.00E+00 |
| cell cycle (GO:0007049) | 188 | 2 | 2.09E-01 | 1.00E+00 |
| regulation of cell communication (GO:0010646) | 95 | 1 | 3.53E-01 | 1.00E+00 |
| regulation of signal transduction (GO:0009966) | 95 | 1 | 3.53E-01 | 1.00E+00 |
| cellular response to DNA damage stimulus (GO:0006974) | 95 | 1 | 3.53E-01 | 1.00E+00 |
| protein phosphorylation (GO:0006468) | 98 | 1 | 3.62E-01 | 1.00E+00 |
| localization (GO:0051179) | 597 | 6 | 4.64E-02 | 1.00E+00 |
| regulation of transcription by RNA polymerase II (GO:0006357) | 207 | 2 | 2.41E-01 | 1.00E+00 |
| translation (GO:0006412) | 108 | 1 | 3.91E-01 | 1.00E+00 |
| regulation of transcription, DNA-templated (GO:0006355) | 219 | 2 | 2.61E-01 | 1.00E+00 |
| regulation of nucleic acid-templated transcription (GO:1903506) | 219 | 2 | 2.61E-01 | 1.00E+00 |
| organic substance biosynthetic process (GO:1901576) | 111 | 1 | 3.99E-01 | 1.00E+00 |
| response to stimulus (GO:0050896) | 230 | 2 | 2.80E-01 | 1.00E+00 |
| regulation of RNA biosynthetic process (GO:2001141) | 233 | 2 | 2.85E-01 | 1.00E+00 |
| regulation of biosynthetic process (GO:0009889) | 238 | 2 | 2.93E-01 | 1.00E+00 |
| regulation of cellular biosynthetic process (GO:0031326) | 238 | 2 | 2.93E-01 | 1.00E+00 |
| system development (GO:0048731) | 124 | 1 | 4.34E-01 | 1.00E+00 |
| cellular protein modification process (GO:0006464) | 274 | 2 | 3.54E-01 | 1.00E+00 |
| cellular response to stress (GO:0033554) | 143 | 1 | 4.81E-01 | 1.00E+00 |
| regulation of metabolic process (GO:0019222) | 432 | 3 | 4.40E-01 | 1.00E+00 |
| cell-cell signaling (GO:0007267) | 146 | 1 | 4.89E-01 | 1.00E+00 |
| cell communication (GO:0007154) | 146 | 1 | 4.89E-01 | 1.00E+00 |
| transcription by RNA polymerase II (GO:0006366) | 292 | 2 | 3.84E-01 | 1.00E+00 |
| cellular protein metabolic process (GO:0044267) | 297 | 2 | 3.92E-01 | 1.00E+00 |
| cellular macromolecule metabolic process (GO:0044260) | 297 | 2 | 3.92E-01 | 1.00E+00 |
| regulation of cellular metabolic process (GO:0031323) | 150 | 1 | 4.98E-01 | 1.00E+00 |
| multicellular organism development (GO:0007275) | 151 | 1 | 5.00E-01 | 1.00E+00 |
| establishment of localization (GO:0051234) | 346 | 2 | 6.68E-01 | 1.00E+00 |
| transport (GO:0006810) | 346 | 2 | 6.68E-01 | 1.00E+00 |
| cell surface receptor signaling pathway (GO:0007166) | 176 | 1 | 5.55E-01 | 1.00E+00 |
| transcription, DNA-templated (GO:0006351) | 359 | 2 | 6.75E-01 | 1.00E+00 |
| cellular response to stimulus (GO:0051716) | 559 | 3 | 7.35E-01 | 1.00E+00 |
| regulation of biological process (GO:0050789) | 755 | 4 | 7.68E-01 | 1.00E+00 |
| gene expression (GO:0010467) | 581 | 3 | 7.42E-01 | 1.00E+00 |
| cellular process (GO:0009987) | 1754 | 9 | 6.68E-01 | 1.00E+00 |
| intracellular signal transduction (GO:0035556) | 198 | 1 | 5.98E-01 | 1.00E+00 |
| signal transduction (GO:0007165) | 438 | 2 | 1.00E+00 | 1.00E+00 |
| biological regulation (GO:0065007) | 881 | 4 | 1.00E+00 | 1.00E+00 |
| Unclassified (UNCLASSIFIED) | 2556 | 11 | 1.00E+00 | 1.00E+00 |
| cellular component organization (GO:0016043) | 474 | 2 | 1.00E+00 | 1.00E+00 |
| cellular protein localization (GO:0034613) | 256 | 1 | 1.00E+00 | 1.00E+00 |
| cellular macromolecule localization (GO:0070727) | 258 | 1 | 1.00E+00 | 1.00E+00 |
| macromolecule metabolic process (GO:0043170) | 818 | 3 | 1.00E+00 | 1.00E+00 |
| regulation of cellular process (GO:0050794) | 275 | 1 | 1.00E+00 | 1.00E+00 |
| cellular metabolic process (GO:0044237) | 567 | 2 | 1.00E+00 | 1.00E+00 |
| organic substance metabolic process (GO:0071704) | 1145 | 4 | 8.04E-01 | 1.00E+00 |
| multicellular organismal process (GO:0032501) | 291 | 1 | 1.00E+00 | 1.00E+00 |
| cellular localization (GO:0051641) | 294 | 1 | 1.00E+00 | 1.00E+00 |
| metabolic process (GO:0008152) | 1369 | 4 | 4.84E-01 | 1.00E+00 |

Table S50. GO analysis in Casuariiformes in the 4-times hypothesis using PANTHER GO-Slim Cellular Component. P values are calculated by Fisher’s exact test and FDR values are calculated by the Benjamini–Hochberg procedure.

| GO term | Number of genes in reference | Number of genes in query | P-values | FDR values |
| --- | --- | --- | --- | --- |
| recycling endosome (GO:0055037) | 6 | 1 | 3.10E-02 | 1.00E+00 |
| polysome (GO:0005844) | 6 | 1 | 3.10E-02 | 1.00E+00 |
| exocyst (GO:0000145) | 7 | 1 | 3.54E-02 | 1.00E+00 |
| cell cortex part (GO:0044448) | 9 | 1 | 4.40E-02 | 1.00E+00 |
| endosome membrane (GO:0010008) | 13 | 1 | 6.11E-02 | 1.00E+00 |
| transcription factor complex (GO:0005667) | 17 | 1 | 7.79E-02 | 1.00E+00 |
| microtubule associated complex (GO:0005875) | 18 | 1 | 8.21E-02 | 1.00E+00 |
| cell cortex (GO:0005938) | 19 | 1 | 8.62E-02 | 1.00E+00 |
| trans-Golgi network (GO:0005802) | 22 | 1 | 9.85E-02 | 1.00E+00 |
| Golgi membrane (GO:0000139) | 41 | 1 | 1.73E-01 | 1.00E+00 |
| microtubule (GO:0005874) | 43 | 1 | 1.80E-01 | 1.00E+00 |
| extracellular matrix (GO:0031012) | 49 | 1 | 2.02E-01 | 1.00E+00 |
| endosome (GO:0005768) | 50 | 1 | 2.06E-01 | 1.00E+00 |
| extracellular space (GO:0005615) | 102 | 2 | 7.85E-02 | 1.00E+00 |
| Golgi subcompartment (GO:0098791) | 58 | 1 | 2.34E-01 | 1.00E+00 |
| extracellular region part (GO:0044421) | 124 | 2 | 1.09E-01 | 1.00E+00 |
| extracellular region (GO:0005576) | 151 | 2 | 1.50E-01 | 1.00E+00 |
| endoplasmic reticulum (GO:0005783) | 80 | 1 | 3.07E-01 | 1.00E+00 |
| organelle subcompartment (GO:0031984) | 95 | 1 | 3.53E-01 | 1.00E+00 |
| ribonucleoprotein complex (GO:1990904) | 104 | 1 | 3.79E-01 | 1.00E+00 |
| microtubule cytoskeleton (GO:0015630) | 105 | 1 | 3.82E-01 | 1.00E+00 |
| endomembrane system (GO:0012505) | 226 | 2 | 2.73E-01 | 1.00E+00 |
| cell periphery (GO:0071944) | 635 | 5 | 1.99E-01 | 1.00E+00 |
| plasma membrane (GO:0005886) | 623 | 4 | 5.18E-01 | 1.00E+00 |
| cytoskeleton (GO:0005856) | 178 | 1 | 5.59E-01 | 1.00E+00 |
| vacuole (GO:0005773) | 358 | 2 | 6.75E-01 | 1.00E+00 |
| cell part (GO:0044464) | 1620 | 9 | 5.09E-01 | 1.00E+00 |
| cell (GO:0005623) | 1627 | 9 | 5.10E-01 | 1.00E+00 |
| membrane-bounded organelle (GO:0043227) | 192 | 1 | 5.87E-01 | 1.00E+00 |
| intracellular (GO:0005622) | 1347 | 7 | 6.44E-01 | 1.00E+00 |
| intracellular part (GO:0044424) | 1199 | 6 | 8.08E-01 | 1.00E+00 |
| Unclassified (UNCLASSIFIED) | 2890 | 13 | 1.00E+00 | 1.00E+00 |
| cytoplasm (GO:0005737) | 1127 | 5 | 1.00E+00 | 1.00E+00 |
| cytosol (GO:0005829) | 234 | 1 | 1.00E+00 | 1.00E+00 |
| cytoplasmic part (GO:0044444) | 715 | 3 | 1.00E+00 | 1.00E+00 |
| organelle (GO:0043226) | 1177 | 4 | 6.31E-01 | 1.00E+00 |
| intracellular non-membrane-bounded organelle (GO:0043232) | 342 | 1 | 1.00E+00 | 1.00E+00 |
| non-membrane-bounded organelle (GO:0043228) | 342 | 1 | 1.00E+00 | 1.00E+00 |
| nucleus (GO:0005634) | 696 | 2 | 7.62E-01 | 1.00E+00 |
| intracellular membrane-bounded organelle (GO:0043231) | 716 | 2 | 7.63E-01 | 1.00E+00 |
| intracellular organelle (GO:0043229) | 717 | 2 | 7.63E-01 | 1.00E+00 |
| protein-containing complex (GO:0032991) | 501 | 1 | 7.22E-01 | 1.00E+00 |

Table S51. GO analysis in Casuariiformes in the 4-times hypothesis using PANTHER GO-Slim Molecular Function. P values are calculated by Fisher’s exact test and FDR values are calculated by the Benjamini–Hochberg procedure.

| GO term | Number of genes in reference | Number of genes in query | P-values | FDR values |
| --- | --- | --- | --- | --- |
| double-stranded RNA binding (GO:0003725) | 5 | 1 | 2.67E-02 | 1.00E+00 |
| growth factor receptor binding (GO:0070851) | 6 | 1 | 3.10E-02 | 1.00E+00 |
| single-stranded RNA binding (GO:0003727) | 6 | 1 | 3.10E-02 | 1.00E+00 |
| protein heterodimerization activity (GO:0046982) | 7 | 1 | 3.54E-02 | 1.00E+00 |
| amide transmembrane transporter activity (GO:0042887) | 7 | 1 | 3.54E-02 | 1.00E+00 |
| hormone binding (GO:0042562) | 8 | 1 | 3.97E-02 | 1.00E+00 |
| peptide hormone binding (GO:0017046) | 8 | 1 | 3.97E-02 | 1.00E+00 |
| extracellular matrix structural constituent (GO:0005201) | 10 | 1 | 4.83E-02 | 1.00E+00 |
| G-protein coupled peptide receptor activity (GO:0008528) | 14 | 1 | 6.54E-02 | 1.00E+00 |
| SNARE binding (GO:0000149) | 18 | 1 | 8.21E-02 | 1.00E+00 |
| protein homodimerization activity (GO:0042803) | 19 | 1 | 8.62E-02 | 1.00E+00 |
| protein dimerization activity (GO:0046983) | 20 | 1 | 9.03E-02 | 1.00E+00 |
| motor activity (GO:0003774) | 20 | 1 | 9.03E-02 | 1.00E+00 |
| translation initiation factor activity (GO:0003743) | 20 | 1 | 9.03E-02 | 1.00E+00 |
| ribonucleoprotein complex binding (GO:0043021) | 21 | 1 | 9.44E-02 | 1.00E+00 |
| MAP kinase activity (GO:0004707) | 22 | 1 | 9.85E-02 | 1.00E+00 |
| ATPase activity, coupled to transmembrane movement of substances (GO:0042626) | 27 | 1 | 1.19E-01 | 1.00E+00 |
| proximal promoter sequence-specific DNA binding (GO:0000987) | 30 | 1 | 1.31E-01 | 1.00E+00 |
| RNA polymerase II proximal promoter sequence-specific DNA binding (GO:0000978) | 30 | 1 | 1.31E-01 | 1.00E+00 |
| ATPase activity, coupled to movement of substances (GO:0043492) | 32 | 1 | 1.38E-01 | 1.00E+00 |
| microtubule binding (GO:0008017) | 43 | 1 | 1.80E-01 | 1.00E+00 |
| ATPase activity (GO:0016887) | 96 | 2 | 7.08E-02 | 1.00E+00 |
| tubulin binding (GO:0015631) | 49 | 1 | 2.02E-01 | 1.00E+00 |
| protein-containing complex binding (GO:0044877) | 51 | 1 | 2.10E-01 | 1.00E+00 |
| passive transmembrane transporter activity (GO:0022803) | 54 | 1 | 2.20E-01 | 1.00E+00 |
| channel activity (GO:0015267) | 54 | 1 | 2.20E-01 | 1.00E+00 |
| transcription factor binding (GO:0008134) | 55 | 1 | 2.24E-01 | 1.00E+00 |
| chromatin binding (GO:0003682) | 58 | 1 | 2.34E-01 | 1.00E+00 |
| protein serine/threonine kinase activity (GO:0004674) | 124 | 2 | 1.09E-01 | 1.00E+00 |
| ATPase activity, coupled (GO:0042623) | 77 | 1 | 2.98E-01 | 1.00E+00 |
| transcription regulatory region sequence-specific DNA binding (GO:0000976) | 78 | 1 | 3.01E-01 | 1.00E+00 |
| transmembrane signaling receptor activity (GO:0004888) | 157 | 2 | 1.59E-01 | 1.00E+00 |
| protein kinase activity (GO:0004672) | 162 | 2 | 1.67E-01 | 1.00E+00 |
| signaling receptor binding (GO:0005102) | 94 | 1 | 3.50E-01 | 1.00E+00 |
| phosphotransferase activity, alcohol group as acceptor (GO:0016773) | 199 | 2 | 2.28E-01 | 1.00E+00 |
| transcription regulatory region DNA binding (GO:0044212) | 100 | 1 | 3.68E-01 | 1.00E+00 |
| signaling receptor activity (GO:0038023) | 201 | 2 | 2.31E-01 | 1.00E+00 |
| nucleoside-triphosphatase activity (GO:0017111) | 210 | 2 | 2.46E-01 | 1.00E+00 |
| DNA-binding transcription factor activity (GO:0003700) | 212 | 2 | 2.50E-01 | 1.00E+00 |
| G-protein coupled receptor activity (GO:0004930) | 107 | 1 | 3.88E-01 | 1.00E+00 |
| pyrophosphatase activity (GO:0016462) | 216 | 2 | 2.56E-01 | 1.00E+00 |
| hydrolase activity, acting on acid anhydrides, in phosphorus-containing anhydrides (GO:0016818) | 216 | 2 | 2.56E-01 | 1.00E+00 |
| hydrolase activity, acting on acid anhydrides (GO:0016817) | 216 | 2 | 2.56E-01 | 1.00E+00 |
| molecular transducer activity (GO:0060089) | 225 | 2 | 2.71E-01 | 1.00E+00 |
| cytoskeletal protein binding (GO:0008092) | 114 | 1 | 4.07E-01 | 1.00E+00 |
| transmembrane transporter activity (GO:0022857) | 238 | 2 | 2.93E-01 | 1.00E+00 |
| structural molecule activity (GO:0005198) | 120 | 1 | 4.23E-01 | 1.00E+00 |
| transcription regulator activity (GO:0140110) | 257 | 2 | 3.25E-01 | 1.00E+00 |
| transferase activity, transferring phosphorus-containing groups (GO:0016772) | 266 | 2 | 3.41E-01 | 1.00E+00 |
| transporter activity (GO:0005215) | 292 | 2 | 3.84E-01 | 1.00E+00 |
| DNA binding (GO:0003677) | 302 | 2 | 6.45E-01 | 1.00E+00 |
| protein binding (GO:0005515) | 690 | 4 | 5.43E-01 | 1.00E+00 |
| nucleic acid binding (GO:0003676) | 532 | 3 | 7.28E-01 | 1.00E+00 |
| heterocyclic compound binding (GO:1901363) | 556 | 3 | 7.34E-01 | 1.00E+00 |
| RNA binding (GO:0003723) | 195 | 1 | 5.93E-01 | 1.00E+00 |
| binding (GO:0005488) | 1436 | 7 | 8.20E-01 | 1.00E+00 |
| Unclassified (UNCLASSIFIED) | 2686 | 12 | 1.00E+00 | 1.00E+00 |
| transferase activity (GO:0016740) | 590 | 2 | 1.00E+00 | 1.00E+00 |
| hydrolase activity (GO:0016787) | 607 | 2 | 1.00E+00 | 1.00E+00 |
| catalytic activity (GO:0003824) | 1504 | 4 | 2.64E-01 | 1.00E+00 |

Table S52. Pathway analysis for Casuariiformes in the 4-times hypothesis using PANTHER pathway. P values are calculated by Fisher’s exact test and FDR values are calculated by the Benjamini–Hochberg procedure.

| GO term | Number of genes in reference | Number of genes in query | P-values | FDR values |
| --- | --- | --- | --- | --- |
| Integrin signalling pathway (P00034) | 44 | 1 | 1.84E-01 | 1.00E+00 |
| PDGF signaling pathway (P00047) | 53 | 1 | 2.17E-01 | 1.00E+00 |
| Angiogenesis (P00005) | 62 | 1 | 2.48E-01 | 1.00E+00 |
| Unclassified (UNCLASSIFIED) | 4758 | 23 | 5.66E-01 | 1.00E+00 |
